# CPT1A-mediated IDO1 succinylation shapes EGFRvIII-driven resistance to tumor electric field therapy in glioblastoma

**DOI:** 10.1038/s41467-026-77927-w

**Published:** 2026-09-23

**Authors:** Bowen Feng, Boyan Li, Qinran Zhang, Daiyan Wang, Weiyang Ma, Qingtong Wang, Huize Xia, Rongrong Zhao, Hongyu Zhao, Kailiang Zhang, Shaobo Wang, Xinglong Zhang, Chuanzheng Wang, Leyang Hao, Yanhua Qi, Zijie Gao, Ziwen Pan, Jiawei Qiu, Zhe Han, Junzhi Liu, Fangyong Wang, Gang Li, Hao Xue

**Affiliations:** 1 Shandong Key Laboratory of Brain Health and Function Remodeling, Department of Neurosurgery, Qilu Hospital of Shandong University, Jinan, China; 2 Institute of Brain and Brain-Inspired Science, Shandong University, Jinan, China; 3 Glioma Comprehensive Diagnosis and Treatment Center, Qilu Hospital of Shandong University, Jinan, China; 4 School of Rehabilitation, Capital Medical University, Beijing Bo’ai Hospital, China Rehabilitation Research Center, Beijing, China; 5 School of Biological Science and Medical Engineering, Beihang University, Beijing, China; 6 University of Health and Rehabilitation Sciences, Qingdao, China; 7 Department of Orthopaedics, Qilu Hospital, Shandong University, Jinan, China

**Keywords:** CNS cancer, Cancer therapeutic resistance, Cancer metabolism

## Abstract

Tumor Electric Field Therapy (TEFT) disrupts mitosis in glioblastoma (GBM), but responses vary markedly among patients. In a retrospective cohort of TEFT-treated GBM, *EGFR* variant III (EGFRvIII) alteration is associated with shorter progression-free survival, prompting us to investigate a genotype-linked resistance mechanism. TEFT triggers a broadly shared bioenergetic stress response marked by activation of the AMPK-PPARα-CPT1A axis, whereas EGFRvIII primes *IDO1* transcription through NF-κB. CPT1A further stabilizes IDO1 by promoting succinylation at lysine 377 through non-canonical LSTase-related activity, thereby limiting TRIM21-dependent ubiquitination and proteasomal degradation. Accumulated IDO1 increases kynurenine production and activates AhR, which upregulates *DCLK1* and *ARHGEF2* to preserve spindle organization and microtubule dynamics during electric-field exposure. Thus, EGFRvIII converts a general stress-adaptation pathway into a selective cytoprotective program. Genetic or pharmacological disruption of this pathway restores TEFT sensitivity in established and patient-derived GBM cells, organoids, and orthotopic models. Osimertinib suppresses the EGFRvIII-NF-κB-IDO1 arm and enhances TEFT efficacy, while exploratory clinical cases provide preliminary mechanism-informed support for the combination in recurrent EGFR-driven GBM. These findings define a genotype–field convergence mechanism linking metabolic adaptation to mitotic protection and support prospective evaluation of osimertinib plus TEFT.

## Abstract (web summary)

Tumour electric field therapy (TEFT) can improve survival in glioblastoma, but responses vary. Here, the authors show that EGFRvIII turns TEFT-induced bioenergetic stress into cytoskeletal protection via IDO1 upregulation and CPT1A-mediated IDO1 stabilisation which can be therapeutically targeted with EGFR inhibition.

## INTRODUCTION

Glioblastoma (GBM) remains the most invasive and lethal primary malignancy of the central nervous system^1,2^. Despite an aggressive standard-of-care regimen comprising maximal surgical resection, radiotherapy, and temozolomide (TMZ) chemotherapy, the prognosis is dismal, with a median overall survival of approximately 14.6 months^3,4^. The nearly inevitable recurrence of GBM reflects profound intratumoral heterogeneity, adaptive cellular plasticity, and therapy-induced selection of resistant subclones^5^. Among the diverse molecular landscapes of GBM, the amplification of the Epidermal Growth Factor Receptor (*EGFR*) gene is the most common alteration, observed in approximately 40–60% of patients^6,7^. Notably, about half of these cases harbor a specific intragenic deletion of exons 2–7, resulting in the constitutively active mutant variant, EGFRvIII^8^. Clinically, EGFRvIII-positive GBM represents a distinct, highly aggressive subtype characterized by enhanced tumorigenicity and intrinsic resistance to conventional genotoxic therapies, including radiation and alkylating agents^6,9,10^. Thus, these features make EGFRvIII an important model for investigating how oncogenic receptor signaling rewires therapeutic vulnerability and resistance in GBM.

In this context of limited therapeutic options, Tumor Electric Field Therapy (TEFT) has emerged as an important non-pharmacological treatment modality. TEFT delivers low-intensity, intermediate-frequency alternating electric fields that exert physical forces on polar and polarizable intracellular structures, thereby disrupting mitotic spindle assembly and triggering mitotic catastrophe^11^. Although large-scale phase III trials have demonstrated that electric field therapy can extend survival, clinical benefit remains heterogeneous across patients^12–15^. A critical unmet need is to identify which molecular subtypes are sensitive or resistant to this biophysical treatment^16–18^. Through a retrospective analysis of a single-center clinical cohort, we observed that patients harboring EGFRvIII alterations had shorter progression-free survival compared with their EGFRvIII-wild-type counterparts when treated with electric field therapy. Although this observation is limited by the retrospective design and modest cohort size, it is consistent with emerging clinical evidence suggesting that EGFR-related alterations may influence the therapeutic benefit of electric field therapy, including reports that EGFR wild-type status or *EGFR* amplification may stratify progression-free survival in TEFT-treated GBM patients^19^. These clinical observations motivated us to investigate whether and how EGFRvIII mechanistically contributes to TEFT resistance in GBM. Emerging evidence suggests that specific metabolic phenotypes adopted by tumor cells under stress are functionally intertwined with their biological outcomes^20–22^. We hypothesized that electric field therapy, by disrupting dipole alignment, might impose a broadly inducible bioenergetic and cytoskeletal stress on GBM cells, while EGFRvIII-mutant cells may exploit this general stress response through genotype-specific signaling programs that confer a survival advantage.

Here, we combine clinical observations, multi-omics profiling, biochemical assays, patient-derived models, and functional validation to elucidate the mechanism of electric field therapy resistance in EGFRvIII-mutant GBM. We identify Indoleamine 2,3-dioxygenase 1 (IDO1) as a critical effector that bridges EGFRvIII signaling, metabolic stress and mitotic survival. While IDO1 is a canonical tryptophan-catabolic enzyme, its abundance is tightly constrained by ubiquitin-dependent proteasomal degradation in tumors^23^. We show that EGFRvIII primes *IDO1* transcription through NF-κB signaling, whereas TEFT triggers a bioenergetic crisis that upregulates the mitochondrial outer-membrane protein CPT1A via the AMPK-PPARα axis. Unexpectedly, we find that CPT1A functions beyond its canonical metabolic role: it promotes IDO1 stabilization through non-canonical lysine succinylation, thereby reducing its ubiquitin-mediated proteasomal degradation. The stabilized IDO1 then enhances kynurenine production and activates an AhR-mediated transcriptional program that supports mitotic spindle and microtubule homeostasis under electric-field stress. Finally, preclinical models and preliminary exploratory compassionate-use cases suggest that targeting this resistance program with the third-generation EGFR-TKI osimertinib can resensitize EGFR-driven GBM to TEFT. Together, these findings provide a rationale for combining osimertinib with TEFT in EGFR-driven GBM and warrant prospective clinical validation.

## RESULTS

### EGFRvIII confers resistance to tumor electric fields through IDO1 upregulation

**Fig. 1:**
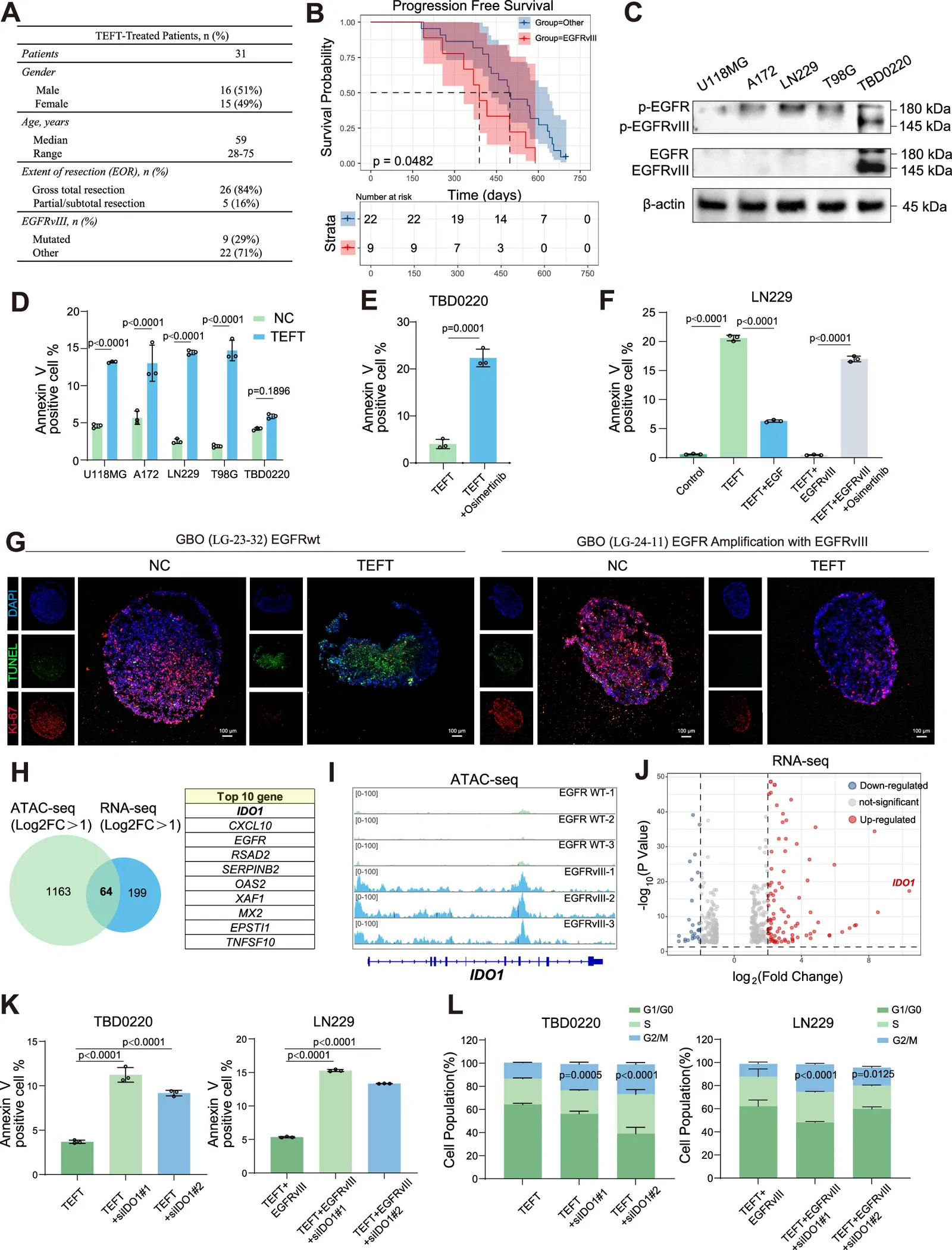
EGFRvIII confers resistance to tumor electric field therapy in GBM through *IDO1* upregulation. **A** Clinical characteristics of the retrospective GBM cohort treated with TEFT (*n* = 31), **B** Kaplan-Meier analysis of progression-free survival (PFS) in GBM patients stratified by EGFRvIII mutation status (*P* = 0.0482, log-rank test), **C** Western blot analysis of basal EGFR and EGFRvIII phosphorylation and expression levels in a panel of GBM cell lines (U118MG, A172, LN229, T98G) and a primary EGFRvIII-mutant line (TBD0220). β-actin served as a loading control, **D** Flow cytometric analysis of apoptosis (Annexin V positive cells) in the indicated cell lines following TEFT treatment (*n* = 3 biological replicates per group), **E** Flow cytometric quantification of apoptosis in TBD0220 cells treated with TEFT alone or TEFT plus osimertinib (*n* = 3 biological replicates per group), **F** Apoptosis rates in LN229 cells under different conditions: control, TEFT alone, TEFT with EGF stimulation (50 ng/mL), TEFT with EGFRvIII transduction, and combination with osimertinib (*n* = 3 biological replicates per group), **G** Representative immunofluorescence images of TUNEL staining (green) in patient-derived glioblastoma organoids (GBOs) with or without EGFRvIII, treated with TEFT. Scale bars, 100 μm, **H** Venn diagram showing the overlap of significantly upregulated genes (RNA-seq, Log₂FC > 1) and increased chromatin accessibility regions (ATAC-seq, Log₂FC > 1) in TEFT-treated EGFRvIII cells compared to wild-type. *IDO1* is identified as a top candidate, **I** ATAC-seq signal tracks showing chromatin accessibility at the *IDO1* promoter region, **J** Volcano plot of RNA**-**seq data highlighting *IDO1* as the most significantly upregulated gene in the EGFRvIII group under TEFT, **K** Rescue effects of *IDO1* knockdown (siRNA) on apoptosis in TEFT-treated EGFRvIII-driven TBD0220 and LN229 cells (*n* = 3 biological replicates per group), **L** Cell cycle distribution analysis showing that IDO1 knockdown restores sensitivity to TEFT-induced mitotic perturbation (G2/M arrest) in EGFRvIII cells (*n* = 3 biological replicates per group). Data are presented as mean ± SD. The exact n values and definitions of biological replicates are specified for the relevant panels. Statistical tests were as follows: two-tailed unpaired Student’s *t*test for panel **E**; ordinary one-way ANOVA followed by two-sided Šídák-adjusted multiple-comparisons tests for **F**,**K**,**L**; and two-way repeated-measures ANOVA followed by two-sided Šídák-adjusted multiple-comparisons tests for (**D**). Exact P values, adjusted for multiple comparisons where applicable, are shown in the corresponding panels. Source data are provided as a Source Data file.

Since 2021, the Department of Neurosurgery at Qilu Hospital of Shandong University has established a real-world “TEFT-GBM” cohort. We consecutively and retrospectively enrolled patients with histologically confirmed glioblastoma who received TEFT after standard Stupp protocol. By the data-cutoff date, 31 patients with complete clinical and molecular profiles were included (median age 59 years, range 28-75; 16 males and 15 females). All patients received TEFT at a field intensity of 2 V cm^−1^ and a frequency of 200 kHz, with treatment compliance of ≥18 h day^−1^. Central neuropathology review identified EGFRvIII alterations in 9 cases (29.0%). Survival analysis showed that the EGFRvIII-altered group had a shorter median progression-free survival than the EGFRvIII-wild-type group (12.97 versus 16.60 months; HR, 3.144; 95% CI, 1.136-8.700; *P* = 0.0482) (Fig. 1A, B). Given the retrospective nature and limited size of this cohort, this association should be interpreted as hypothesis-generating rather than definitive evidence that EGFRvIII independently predicts TEFT resistance. Nevertheless, these clinical findings warranted further mechanistic investigation.

While TEFT induced apoptosis in several standard GBM cell lines (U118MG, A172, LN229, T98G), the primary EGFRvIII-mutant GBM cell line TBD0220 exhibited a relatively limited apoptotic response (Fig. 1C, D and Fig. S1A). Strikingly, activation of EGFR signaling, either through EGF stimulation (50 ng/ml) in parental cells or ectopic EGFRvIII expression, reduced TEFT-induced apoptosis. In TBD0220 and EGFRvIII-expressing LN229 cells, osimertinib increased TEFT-induced apoptosis and restored TEFT sensitivity (Fig. 1E, F and Fig. S1B). Similar effects were observed in T98G-EGFRvIII cells (Fig. S1C–E). Dose-response analysis further showed that TEFT shifted the osimertinib response curve toward lower concentrations, and Bliss analysis supported a synergistic interaction between osimertinib and TEFT in EGFRvIII-driven models (Fig. S1F, G). Furthermore, in patient-derived glioblastoma organoid models, the EGFR-wild-type GBO exhibited prominent TUNEL positivity after TEFT, whereas the *EGFR*-amplified/EGFRvIII-positive GBO showed a more limited apoptotic response (Fig. 1G). These findings support a model in which aberrant EGFR signaling, particularly EGFRvIII-driven signaling, functionally contributes to TEFT resistance in GBM.

To elucidate the molecular drivers of this resistance, we performed a multi-omics analysis integrating transcriptomic and ATAC-seq (Assay for Transposase-Accessible Chromatin using sequencing) data from EGFRwt and EGFRvIII cells under TEFT. Both datasets converged on *IDO1* as a top candidate: overlap analysis identified *IDO1* among the genes showing both transcriptional upregulation and increased chromatin accessibility (Fig. 1H), ATAC-seq revealed a marked increase in chromatin accessibility at the *IDO1* locus (Fig. 1I), and RNA-seq showed that *IDO1* was markedly upregulated in EGFRvIII cells under TEFT treatment (Fig. 1J). Interrogation of TCGA^24^, GTEx^25^, and CGGA^26^ datasets showed that *IDO1* expression was elevated in GBM compared with non-tumor tissue and increased with malignant histology, while high *IDO1* expression was associated with a trend toward poorer disease-free survival (Fig. S2A–D), supporting its clinical relevance.

To determine whether IDO1 is a functional mediator of EGFRvIII-driven resistance rather than a bystander, we performed loss-of-function assays. We first validated the knockdown efficiency of two independent small interfering RNAs in TBD0220, LN229-EGFRvIII, and T98G-EGFRvIII cells, confirming robust depletion at both the mRNA and protein levels (Fig. S2E, F). We then assessed the impact of *IDO1* silencing on cell survival under electric field exposure. Flow cytometric analysis of apoptosis showed that while EGFRvIII expression reduced TEFT-induced cell death, silencing *IDO1* attenuated this protective phenotype, resulting in a marked increase in apoptosis following TEFT treatment (Fig. 1K and Fig. S2G, H). Consistently, cell-cycle analysis revealed that *IDO1* knockdown resensitized EGFRvIII-driven cells to TEFT-induced mitotic perturbation. Whereas EGFRvIII-driven cells displayed less pronounced TEFT-induced G2/M accumulation, IDO1-depleted cells exhibited restored G2/M accumulation and mitotic arrest (Fig. 1L and Fig. S2I–L). These data support a functional requirement for IDO1 in sustaining the TEFT-resistant phenotype in EGFRvIII-driven GBM cells.

To complement these genetic loss-of-function data, we next tested whether pharmacological inhibition of IDO1 catalytic activity could similarly restore TEFT sensitivity. Epacadostat significantly reduced kynurenine concentrations in the culture supernatants of TEFT-treated cells, consistent with inhibition of IDO1 catalytic output (Fig. S3A). Consistent with this biochemical effect, epacadostat significantly increased TEFT-induced apoptosis in all three cell models (Fig. S3B, C). We then extended this analysis to independent low-passage patient-derived GBM cultures. Molecular characterization showed that LG-260 exhibited *EGFR* amplification and EGFRvIII expression, whereas LG-252 lacked the corresponding EGFR-driven signaling profile (Fig. S3D). LG-252 cells remained sensitive to TEFT-induced apoptosis (Fig. S3E, F). In contrast, LG-260 cells showed a limited apoptotic response to TEFT alone, whereas either osimertinib or epacadostat markedly increased TEFT-induced apoptosis (Fig. S3G, H). Thus, direct pharmacological inhibition of IDO1 enzymatic activity phenocopied the resensitizing effect of upstream EGFR blockade in an independent patient-derived EGFR-driven model. Together with the genetic depletion experiments, these findings establish IDO1 as a functional downstream effector of EGFR-driven TEFT resistance.

### EGFRvIII up-regulates *IDO1* transcription in an NF-κB-dependent manner

**Fig. 2:**
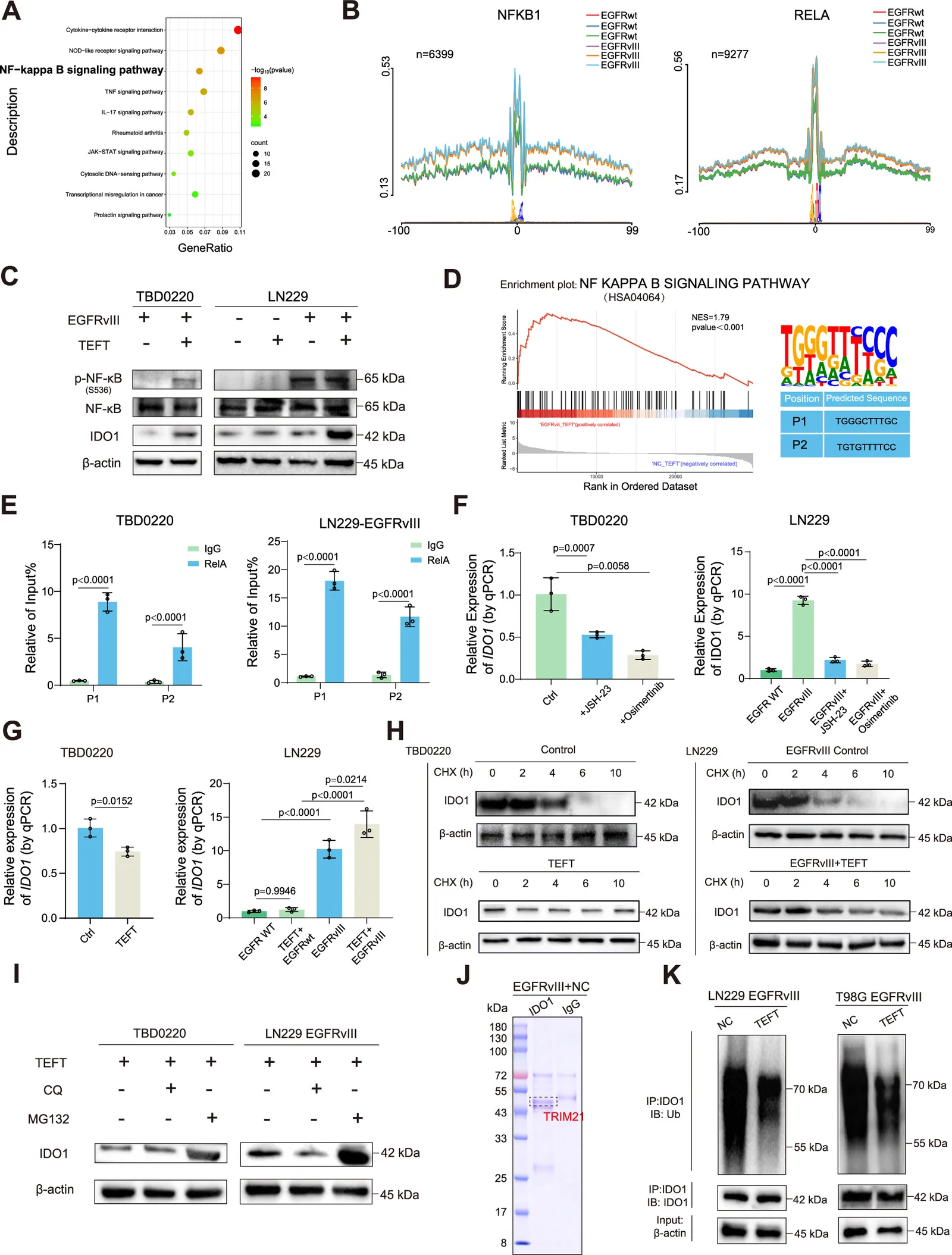
EGFRvIII upregulates *IDO1* via the NF-κB pathway, and TEFT further stabilizes IDO1 protein. **A** KEGG pathway enrichment analysis showing upregulation of the “NF-kappa B signaling pathway” in EGFRvIII-mutant cells compared to wild-type, **B** ATAC-seq footprinting analysis of NFKB1 and RELA (p65) transcription factor binding motifs, showing increased accessibility in EGFRvIII cells versus wild-type, **C** Western blot analysis of phosphorylated NF-κB (p-p65), total NF-κB (p65), showing IDO1 in TBD0220 cells with or without TEFT treatment, and in LN229 cells with or without EGFRvIII expression and TEFT treatment. **D** GSEA enrichment plot confirming the activation of the NF-κB signaling pathway in EGFRvIII cells. Right: Motif logo and predicted binding sequences (P1, P2) of NF-κB on the *IDO1* promoter, **E** ChIP-qPCR analysis of RelA (p65) occupancy at the predicted *IDO1* promoter regions (P1 and P2) in TBD0220 and LN229 cells. Data are presented as percent of input (*n* = 3 biological replicates per group), **F** RT-qPCR analysis of *IDO1* mRNA levels in EGFRvIII-expressing cells treated with the NF-κB inhibitor JSH-23 or osimertinib (*n* = 3 biological replicates per group), **G** RT-qPCR analysis of *IDO1* mRNA expression under TEFT treatment. Note that while EGFRvIII upregulates *IDO1* baseline transcription, TEFT treatment does not further significantly increase *IDO1* mRNA levels (*n* = 3 biological replicates per group), **H** Protein degradation assay using cycloheximide (CHX) chase. TBD0220 and LN229 cells (transduced with EGFRvIII) were treated with TEFT and harvested at the indicated time points. TEFT treatment extends the protein half-life of IDO1, **I** Rescue of IDO1 degradation using proteasome inhibitor (MG132) and lysosome inhibitor (CQ) in TEFT-treated cells. IDO1 accumulation with MG132 indicates proteasomal degradation, **J** Co-immunoprecipitation (Co-IP) of IDO1 followed by Coomassie brilliant blue staining, identifying TRIM21 as an interacting partner (highlighted in red box), **K** Ubiquitination assay in LN229 and T98G cells. TEFT treatment reduces the level of poly-ubiquitinated IDO1 (smear in the IP: IDO1/ IB: Ub Lane) compared to the non-treated control (NC). Data are presented as mean ± SD. The exact n values and definitions of biological replicates are specified for the relevant panels. Statistical tests were as follows: two-tailed unpaired Student’s *t*test for **E** and **G** (TBD0220); ordinary one-way ANOVA followed by two-sided Šídák-adjusted multiple-comparisons tests for panel **F** (LN229). Exact P values, adjusted for multiple comparisons where applicable, are shown in the corresponding panels. Source data are provided as a Source Data file.

To precisely dissect the molecular basis of EGFRvIII-driven IDO1 induction, we first focused on the upstream transcriptional regulatory mechanisms. KEGG pathway analysis revealed enrichment of the NF-κB signaling pathway in EGFRvIII-mutant GBM cells compared with their wild-type counterparts (Fig. 2A). Consistent with this transcriptional signature, ATAC-seq footprinting analysis showed increased accessibility around NFKB1 and RELA binding motifs in EGFRvIII-expressing cells (Fig. 2B). Western blot analysis further supported activation of this pathway, showing elevated phosphorylation of NF-κB p65 together with IDO1 accumulation in TBD0220 and LN229-EGFRvIII cells (Fig. 2C). Additional immunoblotting in an independent EGFRvIII-positive primary model and T98G cells further showed that EGFRvIII expression was associated with enhanced NF-κB phosphorylation and IDO1 abundance, with further IDO1 accumulation following TEFT exposure (Fig. S3I). GSEA analysis further revealed that the NF-κB pathway was enriched in EGFRvIII-mutant GBM cells under TEFT treatment (Fig. 2D). Focusing specifically on the *IDO1* locus, we identified two predicted NF-κB-binding sequences, P1 and P2, within the *IDO1* promoter region. ChIP-qPCR assays showed enhanced RelA occupancy at these predicted *IDO1* promoter regions in TBD0220 and LN229 cells (Fig. 2E), with additional validation in T98G-EGFRvIII cells (Fig. S3J). Functionally, pharmacological inhibition of NF-κB signaling with JSH-23, or upstream blockade of EGFR signaling with osimertinib, reduced *IDO1* mRNA levels (Fig. 2F and Fig. S3K, L). These data indicate that EGFRvIII provides NF-κB-dependent transcriptional priming of *IDO1*.

While EGFRvIII established a high basal level of *IDO1* transcription, we observed discordant regulation of *IDO1* mRNA and protein upon TEFT exposure. TEFT treatment increased IDO1 protein abundance but did not result in a corresponding further elevation of *IDO1* mRNA levels (Fig. 2G and Fig. S3M). This suggested an additional layer of post-translational regulation^27,28^. To investigate protein stability, we employed cycloheximide (CHX) chase assays, which measure protein degradation kinetics. These assays showed that TEFT treatment extended the half-life of IDO1 protein in TBD0220 and LN229-EGFRvIII cells (Fig. 2H), with a consistent effect in T98G-EGFRvIII cells (Fig. S3N). Degradation pathway analysis using the lysosome inhibitor chloroquine (CQ) and the proteasome inhibitor MG132 showed that MG132 increased IDO1 abundance, supporting a major contribution of proteasome-dependent turnover to IDO1 degradation (Fig. 2I and Fig. S4A). To identify the E3 ligase potentially involved in IDO1 proteasomal turnover, we performed IDO1 Co-IP followed by mass spectrometry. This analysis identified the E3 ubiquitin ligase TRIM21 as an IDO1-binding partner (Fig. 2J), consistent with previous evidence that TRIM21 mediates IDO1 ubiquitination and degradation^29^. Importantly, TEFT treatment reduced polyubiquitinated IDO1 in LN229-EGFRvIII and T98G-EGFRvIII cells (Fig. 2K). Furthermore, siRNA-mediated TRIM21 knockdown reduced IDO1 ubiquitination (Fig. S4B-D). Together, these findings suggest that EGFRvIII transcriptionally primes *IDO1* through NF-κB signaling, whereas TEFT attenuates TRIM21-associated proteasomal turnover and promotes post-translational stabilization of IDO1. This dual regulation provides a mechanistic basis for IDO1 accumulation in EGFRvIII-driven cells exposed to electric-field stress.

### TEFT-induced bioenergetic stress activates a broad AMPK-PPARα-CPT1A response and promotes mitochondrial enrichment of IDO1

**Fig. 3:**
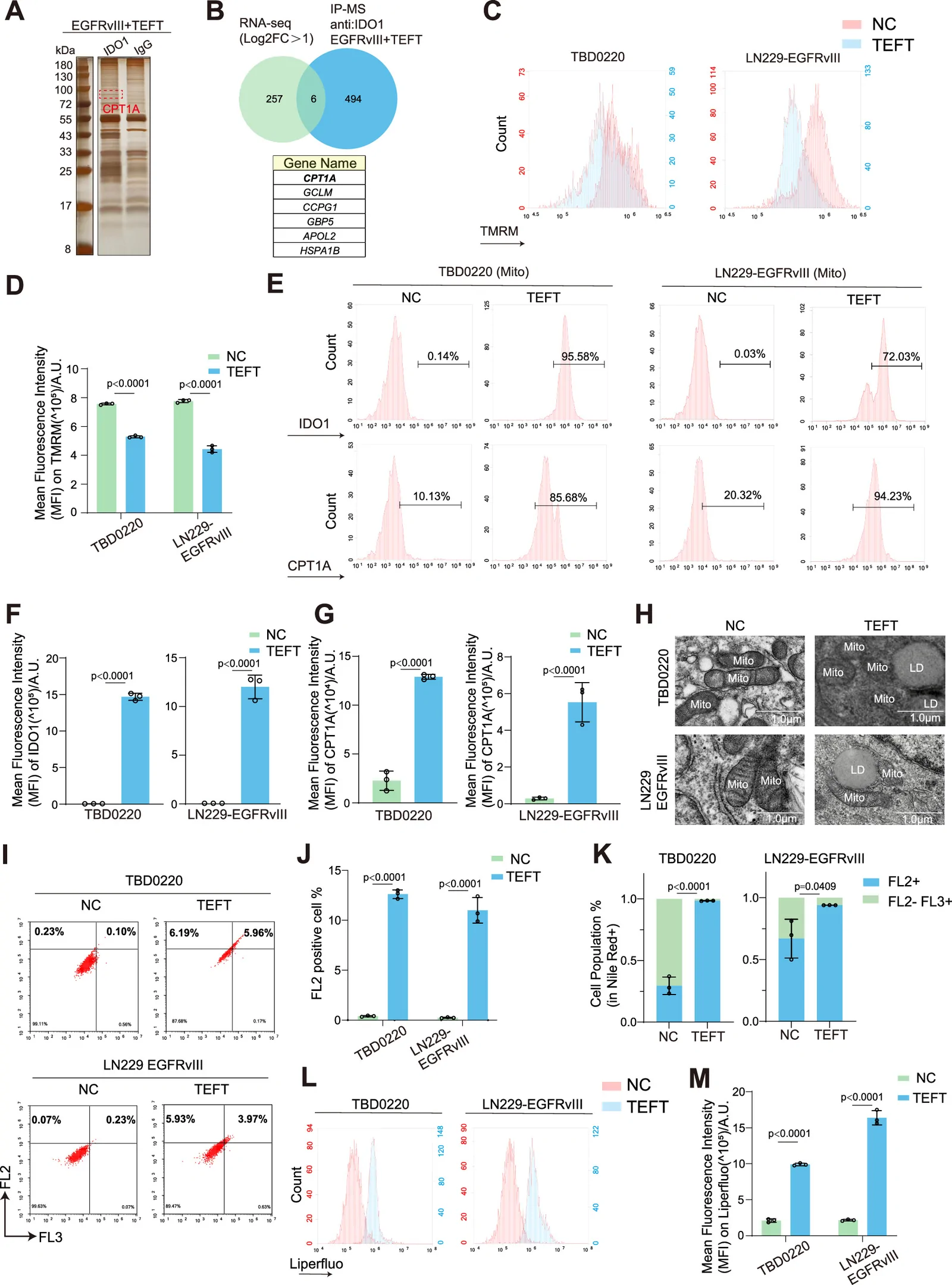
TEFT triggers a bioenergetic crisis that upregulates CPT1A and promotes mitochondrial enrichment of IDO1. **A** Co-immunoprecipitation (Co-IP) of IDO1 in EGFRvIII-expressing LN229 cells treated with TEFT. Silver staining shows the specific interaction with CPT1A (red box), **B** Venn diagram integrating RNA-seq (upregulated genes, Log₂FC > 1) and IP-MS (IDO1-interacting proteins) data, identifying CPT1A as a top candidate, **C** Representative flow cytometry histograms of mitochondrial membrane potential (ΔΨm, TMRM staining) in TBD0220 and LN229-EGFRvIII cells. TEFT treatment causes a leftward shift, indicating depolarization, **D** Quantification of mean fluorescence intensity (MFI) for TMRM (*n* = 3 biological replicates per group), **E** Flow cytometric analysis of isolated mitochondrial fractions stained for IDO1 (top row) and CPT1A (bottom row). TEFT treatment dramatically increases the accumulation of both proteins in the mitochondria, **F**,**G** Quantification of mitochondrial IDO1 (**F**) and CPT1A (**G**) positivity (*n* = 3 biological replicates per group), **H** Representative Transmission Electron Microscopy (TEM) images showing the formation of physical contact sites between mitochondria (Mito) and lipid droplets (LD) under TEFT treatment. **I** Representative flow cytometry dot plots of Nile Red staining showing neutral lipid accumulation (FL2 channel) in cells treated with TEFT, **J** Quantification of the percentage of FL2-positive cells (lipid-rich) (*n* = 3 biological replicates per group), **K** Analysis of lipid droplet subpopulations (FL2+ vs. FL3+ ) in Nile Red-stained cells (*n* = 3 biological replicates per group), **L** Flow cytometric analysis of lipid peroxidation using the Liperfluo probe (*n* = 3 biological replicates per group), **M** Quantification of Liperfluo mean fluorescence intensity in TBD0220 and LN229-EGFRvIII cells (*n* = 3 biological replicates per group). Data are presented as mean ± SD. The exact n values and definitions of biological replicates are specified for the relevant panels. Statistical tests were as follows: two-tailed unpaired Student’s *t*test for panels **F**,**G**,**J**,**K**; ordinary one-way ANOVA followed by two-sided Šídák-adjusted multiple-comparisons tests for **D** and **M**. Exact P values, adjusted for multiple comparisons where applicable, are shown in the corresponding panels. Source data are provided as a Source Data file. Abbreviations: A.U., arbitrary units; Mito, mitochondria; LD, lipid droplet.

To uncover the mechanism underlying this stabilization, we integrated IDO1 Co-IP/MS data with RNA-seq analysis under TEFT conditions. This approach identified the mitochondrial outer-membrane enzyme CPT1A as a candidate IDO1-interacting protein and a transcriptionally upregulated metabolic factor in EGFRvIII cells exposed to TEFT (Fig. 3A, B). We next sought to determine the driver of this upregulation. Metabolic analysis revealed TEFT-induced bioenergetic stress, evidenced by a decrease in mitochondrial membrane potential (ΔΨm), as indicated by tetramethylrhodamine (TMRM) staining in EGFRvIII cells (Fig. 3C, D and Fig. S4E, F). Concurrently, Rhod-2 staining showed mitochondrial Ca²⁺ accumulation after TEFT exposure, further supporting disruption of mitochondrial homeostasis (Fig. S4G, H). Flow cytometric analysis of isolated mitochondrial fractions further showed increased mitochondria-associated IDO1 and CPT1A signals after TEFT treatment (Fig. 3E–G), with consistent findings in T98G-EGFRvIII cells (Fig. S4I, J). The gated mitochondrial population was supported by ATP5A-positive and Calnexin-negative controls (Fig. S4K). Independent subcellular fractionation followed by immunoblotting further demonstrated TEFT-induced enrichment of both IDO1 and CPT1A in the mitochondria-enriched fractions of TBD0220, LN229-EGFRvIII, T98G-EGFRvIII, and patient-derived LG-260 cells (Fig. S4L).

To compensate for this mitochondrial stress, tumor cells may engage adaptive metabolic mechanisms to sustain viability. Consistent with reports that bioenergetic stress drives fatty-acid oxidation to maintain tumor survival, we hypothesized that this TEFT-induced energy crisis triggers a coordinated metabolic reprogramming^30,31^. To determine whether this stress response was restricted to EGFRvIII-driven cells, we examined parental LN229 and T98G cells. Additional JC-1 analyses confirmed mitochondrial depolarization in EGFRvIII-driven models (Fig. S5A, B). In parental LN229 and T98G cells, TEFT induced mitochondrial depolarization and mitochondrial calcium accumulation, as shown by JC-1, TMRM, and Rhod-2 staining (Fig. S5C–G). These results indicate that TEFT-induced mitochondrial and redox stress is broadly inducible across GBM cells rather than being restricted to EGFRvIII-driven models. GSEA analysis of the transcriptome provided mechanistic insight, showing negative enrichment of oxidative phosphorylation signatures but positive enrichment of the PPARα pathway (Fig. S6A). Validating this cellular energy deficit, Western blotting showed activation of the energy sensor AMPK, evidenced by increased AMPKα phosphorylation in EGFRvIII-driven models (Fig. S6B). TEFT also increased AMPKα phosphorylation and CPT1A expression in parental LN229 and T98G cells, further supporting a broadly induced metabolic-stress response (Fig. S6C). Furthermore, Co-IP assays showed that TEFT enhanced the interaction between PPARα and its co-activator PGC1α in both EGFRvIII-driven and parental GBM cells (Fig. S6D, E). ChIP-qPCR further showed increased PPARα occupancy at predicted PPRE motifs within the *CPT1A* promoter (Fig. S6F). Together, these findings indicate that TEFT activates a broadly inducible AMPK-PPARα-CPT1A metabolic-stress program. However, this upstream response alone is insufficient to establish the complete IDO1-dependent resistance cascade, which also requires EGFRvIII-mediated *IDO1* transcriptional priming. Consistent with this distinction, osimertinib did not abolish TEFT-induced AMPKα phosphorylation or CPT1A upregulation (Fig. S6G), suggesting that EGFR inhibition primarily disrupts the IDO1 supply arm rather than the upstream metabolic-stress response.

Previous studies have established that CPT1A functions beyond its enzymatic role, acting as a structural molecular bridge by interacting with the lipid droplet (LD)-associated proteins Rab14 and PLIN2 to facilitate organelle tethering and fatty-acid flux^32^. Motivated by this established structural function, we sought to visualize these contact sites. Transmission electron microscopy showed that TEFT treatment increased the physical proximity between mitochondria and lipid droplets in EGFRvIII-driven cells (Fig. 3H and Fig. S6H). Consequently, this metabolic remodeling resulted in increased neutral lipid accumulation and altered lipid-droplet subpopulations, as shown by Nile Red staining (Fig. 3I–K and Fig. S6I–K), together with elevated lipid oxidative stress detected by Liperfluo staining (Fig. 3L, M and Fig. S7A–D). To assess whether redox stress contributes to this adaptive response, we treated cells with the ROS scavenger NAC. NAC co-treatment increased TEFT-induced apoptosis and attenuated CPT1A protein accumulation. In contrast, total IDO1 abundance was only modestly affected and did not show a consistent reduction across the tested models (Fig. S7E–G). These findings suggest that redox stress contributes primarily to the upstream CPT1A-associated metabolic adaptation, but is not the sole determinant of IDO1 abundance or the TEFT-resistant phenotype. Together, these data support a model in which TEFT induces a broad mitochondrial metabolic stress response that activates the AMPK-PPARα-CPT1A axis and promotes mitochondrial-fraction enrichment of CPT1A and IDO1. However, whether this CPT1A-associated mitochondrial enrichment directly regulates IDO1 protein fate remained to be elucidated.

### CPT1A Stabilizes IDO1 via LSTase activity to antagonize ubiquitination

**Fig. 4:**
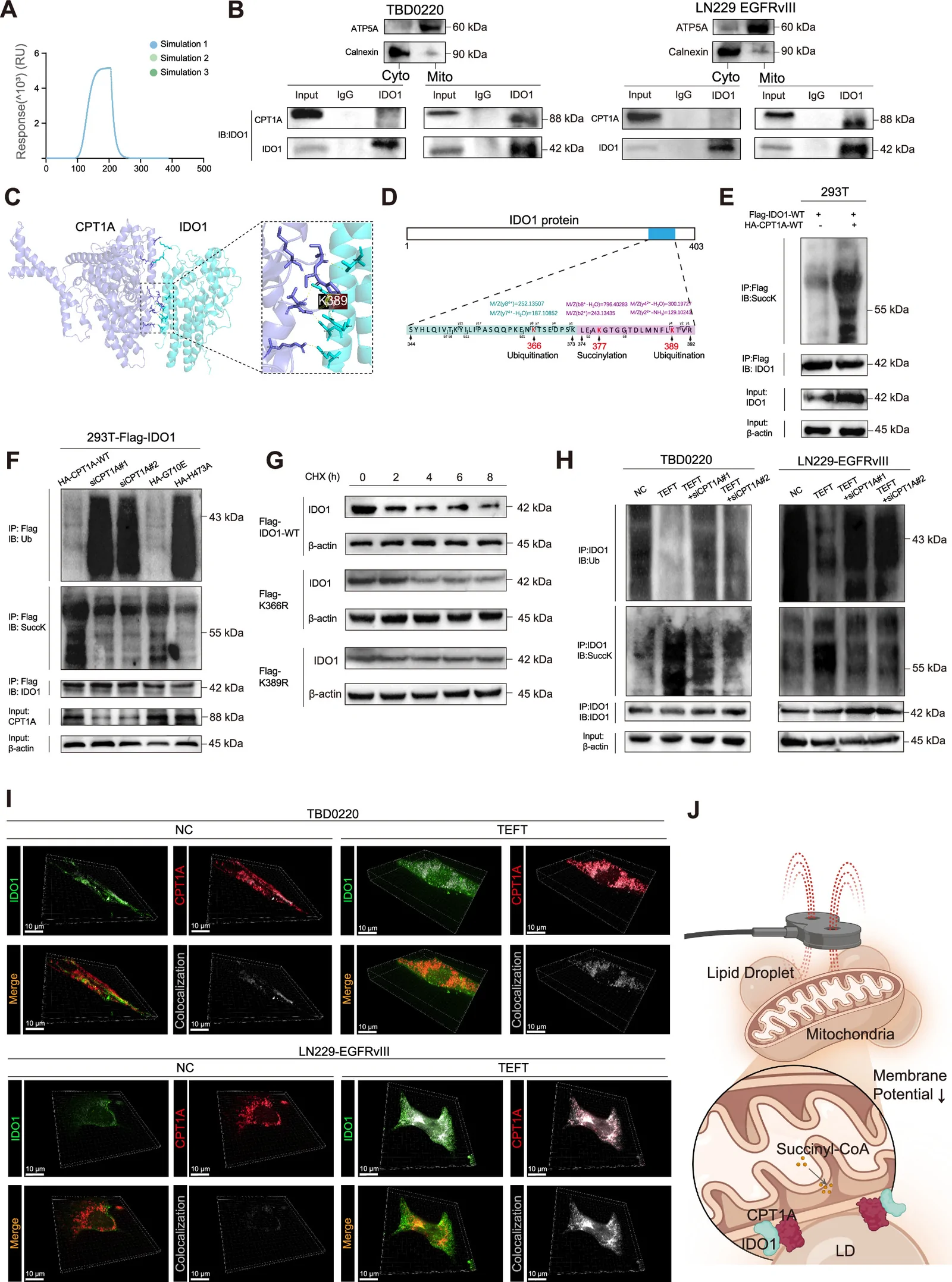
CPT1A stabilizes IDO1 via LSTase activity, antagonizing ubiquitin-mediated degradation. **A** Surface plasmon resonance (SPR) analysis showing the direct physical interaction between purified CPT1A and IDO1 proteins, **B** Subcellular fractionation followed by IDO1 co-immunoprecipitation in TBD0220 and LN229-EGFRvIII cells. CPT1A-IDO1 complex formation was enriched in the mitochondrial fraction. ATP5A and Calnexin were used to assess mitochondrial and non-mitochondrial fraction quality, respectively, **C** AlphaFold3-predicted protein-protein docking model of the CPT1A-IDO1 complex. The inset highlights the interaction interface covering the key lysine residues (e.g., K389), **D** Schematic map of IDO1 protein showing the identified post-translational modification sites: ubiquitination at K366/K389 and succinylation at K377, **E** Immunoprecipitation (IP) assay in 293T cells showing that CPT1A overexpression increases IDO1 succinylation (IB: SuccK) while decreasing IDO1 ubiquitination (IB: Ub), **F** Rescue experiment in CPT1A-knockdown cells. Re-expression of wild-type (WT) CPT1A or the LSTase-active mutant (G710E) restores IDO1 succinylation and stability, whereas the LSTase-dead mutant (H473A) fails to do so, **G** Cycloheximide (CHX) chase assay in 293T cells transfected with IDO1-WT or mutants (K366R, K389R). The K389R mutant exhibits a significantly extended half-life, mimicking the stabilized state, **H** IP analysis of endogenous IDO1 ubiquitination and succinylation in EGFRvIII-GBM cells. TEFT promotes IDO1 succinylation and reduces ubiquitination; this effect is reversed by CPT1A knockdown, **I** High-resolution 3D confocal imaging showing TEFT-enhanced colocalization between IDO1 and CPT1A-positive mitochondrial structures in TBD0220 and LN229-EGFRvIII cells. CPT1A was used as the OMM-resident mechanistic interactor defining the CPT1A-associated mitochondrial interface. Scale bars, 10 μm. **J** Proposed topological model of CPT1A-mediated IDO1 succinylation under TEFT-induced mitochondrial stress. TEFT induces mitochondrial depolarization and permeability stress, which may provide local access to succinyl-CoA or succinyl-CoA-related acyl equivalents at the CPT1A-associated OMM interface. IDO1 is recruited to CPT1A-positive mitochondrial structures, where CPT1A-mediated K377 succinylation antagonizes ubiquitination at the adjacent K389 degron and stabilizes IDO1. Created in BioRender. Feng, B. (2026) https://BioRender.com/643b38a. Source data are provided as a Source Data file. Abbreviations: RU, response units; Cyto, cytoplasm; Mito, mitochondria; LD, lipid droplet.

Motivated by the finding that TEFT drives mitochondrial-fraction enrichment of CPT1A and IDO1, we next investigated whether this spatial proximity facilitates a direct regulatory interaction. We hypothesized that CPT1A stabilizes IDO1 through physical binding and enzymatic modification. Surface plasmon resonance (SPR) assays supported a direct interaction between purified recombinant CPT1A and IDO1 proteins (Fig. 4A). To define the subcellular context of this interaction, we performed subcellular fractionation followed by IDO1 Co-IP in TBD0220 and LN229-EGFRvIII cells. TEFT enhanced the CPT1A-IDO1 interaction, and the complex was enriched in the mitochondrial fraction, with ATP5A and Calnexin used to assess fraction quality (Fig. 4B). Given that CPT1A is an established outer mitochondrial membrane-resident enzyme, these findings support recruitment of IDO1 to a CPT1A-associated OMM interface rather than import of IDO1 into the mitochondrial matrix. Additional Co-IP assays in EGFRvIII-driven models, including patient-derived LG-260 cells, showed that TEFT increased IDO1-CPT1A association while reducing IDO1-TRIM21 association (Fig. S8A, B). Structural modeling using AlphaFold3 predicted a CPT1A-IDO1 docking interface near the IDO1 regulatory region containing the K377 succinylation site and the adjacent K389 ubiquitination-associated degron (Fig. 4C)^33^.

CPT1A possesses carnitine palmitoyl transferase (CPTase) activity and has also been reported to exhibit non-canonical lysine succinyltransferase-related (LSTase) activity^34,35^. We next investigated whether CPT1A exerts a post-translational modification on IDO1. Given CPT1A’s reported moonlighting function as a lysine succinyltransferase, we performed LC-MS/MS analysis on IDO1. This identified lysine 377 (K377) as a candidate succinylation site and lysine 366/389 (K366/K389) as ubiquitination-associated sites (Fig. 4D and Fig. S8C). Functional validation demonstrated that CPT1A overexpression increased IDO1 succinylation (Fig. 4E). Conversely, CPT1A depletion reduced IDO1 succinylation and increased IDO1 ubiquitination, whereas re-expression of wild-type CPT1A restored succinylation and reduced ubiquitination (Fig. 4F). To distinguish this effect of the LSTase-related activity of CPT1A from its canonical carnitine palmitoyl transferase (CPTase) function^36,37^, we utilized specific point mutants: CPT1A-G710E, which retains LSTase activity but lacks CPTase activity, and CPT1A-H473A, a catalytically inactive mutant. In CPT1A-depleted cells, re-expression of wild-type CPT1A or the G710E mutant restored IDO1 succinylation and reduced IDO1 ubiquitination, whereas the H473A mutant failed to do so (Fig. 4F). These mutant-rescue data support the requirement of CPT1A LSTase-related activity, rather than classical CPTase activity alone, for the post-translational regulation of IDO1.

To obtain direct biochemical evidence for CPT1A-mediated IDO1 succinylation, we performed cell-free assays using purified recombinant proteins. CPT1A-WT promoted IDO1 succinylation in the presence of succinyl-CoA, whereas the H473A mutant showed reduced activity toward IDO1 (Fig. S8D). IDO1 succinylation also increased with increasing succinyl-CoA concentrations in the presence of CPT1A (Fig. S8E). Moreover, TEFT did not significantly increase total cellular succinyl-CoA concentrations in TBD0220, LN229-EGFRvIII, or T98G-EGFRvIII cells, arguing against a mechanism driven solely by a global increase in succinyl-CoA availability or nonspecific non-enzymatic succinylation (Fig. S8F).

To determine the precise mechanism of stabilization, we generated IDO1 lysine mutants. Cycloheximide (CHX) chase assays revealed that the IDO1-K389R mutant exhibited a significantly extended half-life compared with wild-type IDO1, implicating K389 as an important ubiquitination-associated degron that contributes to IDO1 turnover (Fig. 4G). Consistent with this, the K389R mutation reduced IDO1 ubiquitination (Fig. S8G). Evolutionary analysis showed that the K366, K377, and K389 sites are highly conserved across species, supporting the potential functional importance of this regulatory region (Fig. S8H). Finally, we verified this axis in the context of TEFT. In TBD0220 and LN229-EGFRvIII cells, TEFT treatment increased IDO1 succinylation and reduced its ubiquitination; importantly, CPT1A knockdown attenuated this TEFT-induced post-translational switch (Fig. 4H). Consistent results were obtained in T98G-EGFRvIII and patient-derived EGFR-driven cells (Fig. S8I–L). Thus, TEFT promotes a CPT1A-dependent shift in IDO1 post-translational regulation from ubiquitination toward succinylation, thereby supporting IDO1 stabilization.

To visually corroborate this interaction in situ, we performed immunofluorescence colocalization assays. High-resolution three-dimensional z-stack imaging revealed that TEFT increased the overlap between IDO1 and CPT1A-positive structures in TBD0220 and LN229-EGFRvIII cells (Fig. 4I and Fig. S9A). Conventional confocal imaging and line-scan analysis in TBD0220, LN229-EGFRvIII, and T98G-EGFRvIII cells provided additional evidence of TEFT-enhanced spatial proximity between IDO1 and CPT1A-positive mitochondrial structures (Fig. S9B, C). Together with the mitochondrial fractionation Co-IP data, these imaging results support recruitment of IDO1 to a CPT1A-associated OMM interface. Collectively, these data support a model in which TEFT-induced bioenergetic stress upregulates CPT1A and promotes its interaction with IDO1 at CPT1A-positive mitochondrial structures. CPT1A-dependent succinylation of IDO1 at K377 functionally antagonizes ubiquitination at the adjacent K389 degron, thereby preserving the IDO1 pool for downstream signaling (Fig. 4J).

### IDO1-AhR axis drives TEFT resistance by rewiring mitotic progression

**Fig. 5:**
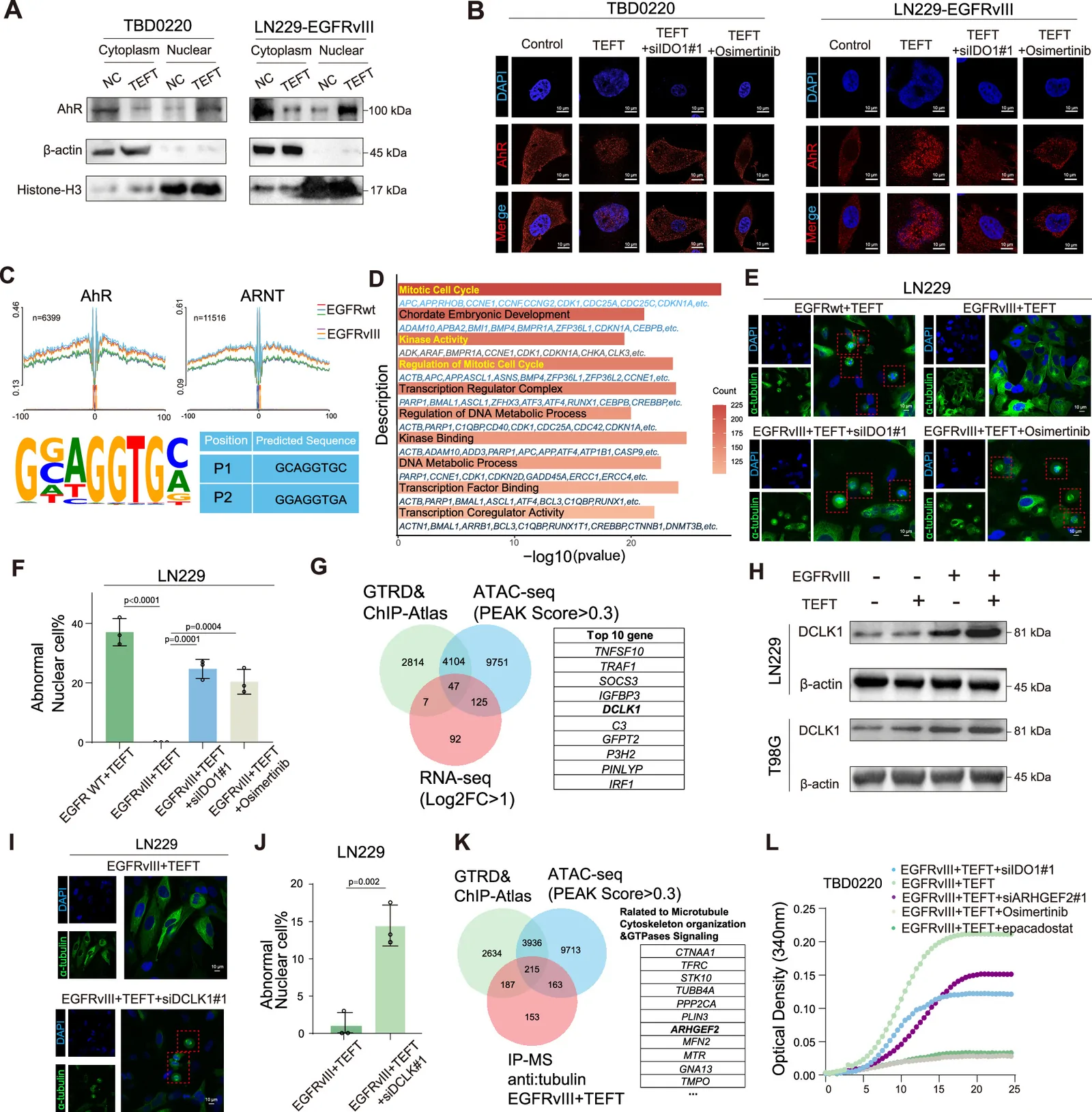
The IDO1-AhR axis confers resistance to TEFT by upregulating mitotic regulators and stabilizing the cytoskeleton. **A** Western blot analysis of AhR in cytoplasmic and nuclear fractions of TBD0220 and LN229-EGFRvIII cells. TEFT treatment promotes AhR nuclear translocation, **B** Immunofluorescence staining of AhR (red) and DAPI (blue). TEFT induces AhR nuclear accumulation, which is reversed by IDO1 knockdown or osimertinib. Scale bars, 10 μm, **C** ATAC-seq footprinting showing increased chromatin accessibility at the AhR binding motif in EGFRvIII cells versus wild-type, **D** GO enrichment analysis of AhR target genes (identified by intersecting ATAC-seq and ChIP-Atlas), highlighting “Mitotic Cell Cycle” and “Microtubule Cytoskeleton” as top pathways, **E** Representative immunofluorescence images of mitotic spindles (α-tubulin, green) and chromosomes (DAPI, blue) in LN229 cells. TEFT causes severe spindle disruption and multipolar mitosis in wild-type cells, whereas EGFRvIII cells maintain bipolar spindles. This protection is lost upon IDO1 knockdown or osimertinib treatment, **F** Quantification of cells with abnormal nuclei following TEFT treatment (*n* = 3 biological replicates per group), **G** Venn diagram intersecting RNA-seq (upregulated), ATAC-seq (accessible peaks), and ChIP-Atlas (AhR binding targets) data, identifying *DCLK1* as a key downstream target, **H** Western blot confirming the upregulation of DCLK1 protein in EGFRvIII cells upon TEFT, **I** Immunofluorescence images of mitotic spindles in *DCLK1*-knockdown cells. Silencing *DCLK1* sensitizes EGFRvIII cells to TEFT-induced spindle defects, **J** Quantification of abnormal nuclear morphology in DCLK1-depleted cells (*n* = 3 biological replicates per group), **K** Venn diagram integrating multi-omics data with microtubule/GTPase-related genes, identifying *ARHGEF2* (GEF-H1) as another target, **L** In vitro tubulin polymerization assay in TBD0220 cells under the indicated conditions. IDO1 knockdown, ARHGEF2 knockdown, osimertinib, or epacadostat reduced the microtubule-polymerizing activity associated with EGFRvIII-mediated TEFT resistance. Data are presented as mean ± SD. The exact n values and definitions of biological replicates are specified for the relevant panels. Statistical tests were as follows: two-tailed unpaired Student’s *t*test for panel **J**; ordinary one-way ANOVA followed by two-sided Šídák-adjusted multiple-comparisons tests for panel **F**. Exact P values, adjusted for multiple comparisons where applicable, are shown in the corresponding panels. Source data are provided as a Source Data file.

The stabilization of IDO1 initiates a crucial downstream signaling cascade by converting tryptophan into kynurenine, a high-affinity endogenous ligand for the aryl hydrocarbon receptor (AhR)^38,39^. To determine whether TEFT-stabilized IDO1 remained enzymatically active, we measured kynurenine levels in culture supernatants. Kynurenine ELISA showed that TEFT increased extracellular kynurenine concentrations in EGFRvIII-driven GBM models, including TBD0220, LN229-EGFRvIII, and T98G-EGFRvIII cells (Fig. S10D). We found that TEFT treatment in EGFRvIII cells significantly promoted AhR nuclear translocation (Fig. 5A, B), supporting its activation. Similar AhR nuclear accumulation was observed in T98G-EGFRvIII and patient-derived LG-260 cells (Fig. S10A, B). Importantly, IDO1 knockdown or EGFR inhibition with osimertinib reduced TEFT-induced AhR nuclear accumulation, supporting placement of AhR activation downstream of EGFRvIII-dependent IDO1 signaling (Fig. 5B and Fig. S10B).

Further genomic analysis using our ATAC-seq data revealed enrichment of AhR/ARNT motifs in EGFRvIII cells under TEFT exposure (Fig. 5C). Consistent with transcriptionally active AhR signaling, RNA-seq analysis showed induction of the canonical AhR-responsive genes *CYP1A1* and *CYP1B1* after TEFT treatment (Fig. S10C). To identify the physiological targets of this axis, we evaluated AhR target genes in the public ChIP-Atlas and GTRD databases^40,41^ and performed GO enrichment analysis. The results showed that “Mitotic Cell Cycle” and “Microtubule Cytoskeleton” were among the most highly enriched pathways (Fig. 5D), consistent with the core biophysical mechanism of TEFT^11,42,43^. This molecular observation directed us to investigate the phenotypic consequences on mitosis. Phenotypic analysis demonstrated that while TEFT typically induces mitotic defects, characterized by spindle misalignment and abnormal nuclear morphology in control cells, EGFRvIII expression conferred a structural protective effect, maintaining bipolar spindle integrity and reducing the percentage of abnormal nuclei. This protection was impaired upon *IDO1* silencing or inhibition with osimertinib, supporting a functional role for IDO1 signaling in TEFT resistance (Fig. 5E, F). Similar spindle-disruptive effects of IDO1 knockdown, osimertinib, or the IDO1 inhibitor epacadostat were observed in TBD0220 and LN229-EGFRvIII cells (Fig. S10E–J). Together, these findings support an IDO1-kynurenine-AhR cascade that contributes to mitotic and microtubule protection during TEFT resistance.

To identify the downstream structural effectors, we employed a multi-omics intersection strategy. Specifically, we intersected public AhR cistrome data from ChIP-Atlas^40^, ATAC-seq data, and our RNA-seq data to screen for genes regulating spindle assembly, which led to the identification of *DCLK1* (doublecortin-like kinase 1) (Fig. 5G). To validate the direct regulatory link, we performed ChIP-qPCR assays, which detected AhR occupancy at the predicted *DCLK1* and *ARHGEF2* promoter regions, supporting direct AhR-dependent transcriptional regulation of *DCLK1* (Fig. S11A). We confirmed that TEFT treatment upregulated DCLK1 protein levels in EGFRvIII-expressing LN229 and T98G cells (Fig. 5H). Functionally, DCLK1 contributed to the observed structural protection^44,45^; silencing DCLK1 in EGFRvIII cells disrupted spindle organization and increased abnormal nuclear morphology (Fig. 5I, J). These effects were further validated in TBD0220 and T98G-EGFRvIII cells (Fig. S11C–F). Thus, DCLK1 functions as a direct AhR-regulated mitotic effector that contributes to cytoskeletal protection under TEFT stress.

To further dissect the mechanism underlying microtubule stability under physical stress, we sought to identify effectors that directly associate with the cytoskeleton. We performed immunoprecipitation coupled with mass spectrometry (IP-MS) using anti-tubulin antibodies in TEFT-treated EGFRvIII cells to isolate the tubulin interactome. By intersecting these proteomic data with our genomic ATAC-seq and public AhR target datasets, we identified ARHGEF2 (GEF-H1) as a microtubule-associated effector (Fig. 5K)^17,46–50^. Similar to DCLK1, ChIP-qPCR analysis demonstrated increased AhR occupancy at predicted motifs within the *ARHGEF2* promoter in LN229-EGFRvIII and T98G-EGFRvIII cells after TEFT treatment, supporting *ARHGEF2* as a direct transcriptional target of AhR (Fig. S11B). Efficient ARHGEF2 knockdown was confirmed at both the mRNA and protein levels (Fig. S11G, H). To validate its functional role, we performed in vitro tubulin polymerization assays. Lysates from TEFT-treated EGFRvIII cells exhibited a capacity to promote microtubule polymerization. Importantly, this stabilizing effect was attenuated by IDO1 knockdown, *ARHGEF2* knockdown, osimertinib, or epacadostat in TBD0220 cells (Fig. 5L), with consistent effects observed in LN229-EGFRvIII and T98G-EGFRvIII cells (Fig. S11I). These complementary perturbations place IDO1 activity, EGFR signaling, and AhR-regulated ARHGEF2 within the same functional cytoskeletal protection program rather than representing independent correlative events.

### EGFR-TKI enhances TEFT efficacy in EGFR-driven GBM models and exploratory clinical cases

**Fig. 6:**
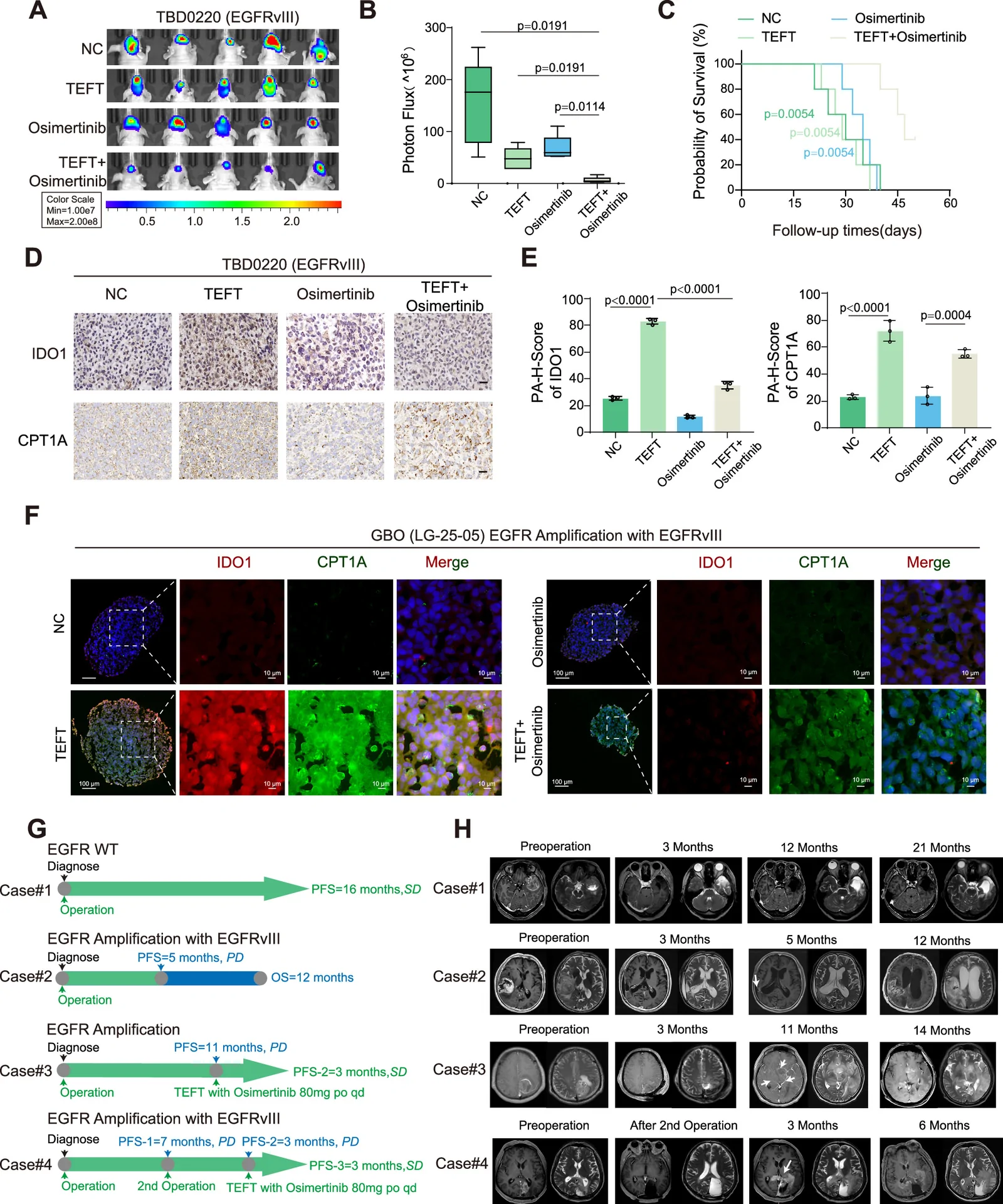
Osimertinib potentiates TEFT efficacy in vivo and reverses adaptive resistance mechanisms in patient-derived samples. **A** Representative bioluminescence images of mice bearing orthotopic EGFRvIII-GBM (TBD0220) xenografts from different treatment groups (NC, TEFT, Osimertinib, combined), showing minimal tumor burden in the combination group, **B** Quantification of tumor photon flux, indicating significant tumor regression in the combination group compared to monotherapies. Data are presented as box-and-whisker plots. The centre line indicates the median, the lower and upper bounds of the box indicate the 25th and 75th percentiles, respectively, and the whiskers extend from the minimum to the maximum value. n = 5 mice per group; each mouse represents an independent biological replicate, **C** Kaplan-Meier survival curves for mice in the NC, TEFT, osimertinib, and TEFT plus osimertinib groups. Three prespecified comparisons of the TEFT plus osimertinib group with the NC, TEFT, and osimertinib groups were performed using two-sided log-rank (Mantel-Cox) tests, with Holm-Šídák adjustment for multiple comparisons. The multiplicity-adjusted P values are shown, **D** Immunohistochemical (IHC) analysis of harvested mouse tumor tissues showing IDO1 (top) and CPT1A (bottom) expression. Scale bars, 50 μm, **E** Quantification of histological scores (H-Score) for IDO1 (left) and CPT1A (right) (*n* = 3 mice per group), **F** Representative immunofluorescence images of IDO1 (red) and CPT1A (green) in patient-derived glioblastoma organoids (GBO, LG-25-05) treated ex vivo. Nuclei were stained with DAPI (blue), **G** Clinical timelines (swimming plots) delineating the disease course, treatment regimens, and therapeutic responses assessed by RANO criteria. CR, complete response; PD, progressive disease; SD, stable disease. Notably, radiological disease stabilization was observed during the available follow-up period for both Case #3 and Case #4, **H** Representative longitudinal MRI scans corresponding to the clinical timelines. Case #1 (EGFR-WT/vIII-negative) shows a sustained response (PFS > 16 months) to standard TEFT, indicating sensitivity in the absence of EGFR activation. Case #2 (EGFRvIII-positive) exhibits rapid recurrence (white arrow) within 5 months despite TEFT treatment, supporting EGFRvIII-driven intrinsic resistance. Case #3 (EGFR-Amplified) presented with recurrence and severe mass effect after standard therapy but showed significant radiological improvement and resolution of midline shift 3 months after initiating TEFT plus osimertinib. Case #4 (EGFRvIII-positive) suffered from rapid recurrence (PFS = 3 months) after a second resection but achieved disease control (SD) and tumor stabilization upon receiving the combination salvage therapy. Data are presented as mean ± SD. The exact n values and definitions of biological replicates are specified for the relevant panels. Statistical tests were as follows: ordinary one-way ANOVA followed by two-sided Šídák-adjusted multiple-comparisons tests for panel E； For panel B, overall group differences were assessed using Brown-Forsythe and Welch one-way ANOVA. Three prespecified comparisons of the TEFT plus osimertinib group with the NC, TEFT, and osimertinib groups were subsequently performed using two-sided unpaired Welch’s t-tests, with Holm adjustment for the three comparisons. Exact P values, adjusted for multiple comparisons where applicable, are shown in the corresponding panels. Source data are provided as a Source Data file.

To translate our mechanistic findings into a therapeutic strategy, we first evaluated the efficacy of combining TEFT with the third-generation EGFR-TKI osimertinib in orthotopic EGFRvIII-driven GBM models. In the TBD0220 orthotopic model, bioluminescence imaging revealed that while TEFT or osimertinib monotherapy only modestly inhibited tumor growth, the combination reduced tumor burden more effectively than either treatment alone (Fig. 6A, B). This enhanced tumor control translated into a significant survival benefit, with the combination group exhibiting extended overall survival compared with either monotherapy (Fig. 6C). A consistent therapeutic pattern was observed in an additional LN229-EGFRvIII orthotopic model (Fig. S12A–C). Immunohistochemical analysis of the harvested TBD0220 tumor tissues provided in vivo support for our mechanistic model: TEFT treatment increased both CPT1A and IDO1 protein staining, whereas concurrent osimertinib administration reduced TEFT-associated IDO1 accumulation and attenuated the resistance-associated IDO1/CPT1A signature (Fig. 6D, E). These in vivo findings support the concept that EGFR inhibition enhances TEFT efficacy in EGFRvIII-driven GBM models without implying complete abrogation of the pathway.

To bridge the gap between animal models and human physiology, we further validated this resistance axis using patient-derived glioblastoma organoids (GBOs). We established GBOs from recurrent GBM patients, including LG-25-05 and LG-24-03, harboring EGFR amplification and EGFRvIII alterations. Consistent with our in vivo findings, immunofluorescence analysis revealed that TEFT treatment increased IDO1 and CPT1A signals in these organoids (Fig. 6F and Fig. S12D). Importantly, the addition of osimertinib to TEFT reduced TEFT-induced IDO1 accumulation and altered the IDO1/CPT1A co-expression pattern. These ex vivo findings support the presence of an EGFR-driven CPT1A-IDO1 resistance program in patient-derived tumor models and its pharmacological disruption by osimertinib, although they do not by themselves establish clinical efficacy.

Motivated by these preclinical and ex vivo data, we evaluated exploratory clinical cases involving GBM patients receiving standard Stupp protocol chemoradiotherapy combined with TEFT. The patient with EGFR wild-type disease maintained durable disease stability, whereas patients harboring EGFR amplification with or without EGFRvIII experienced earlier progression (Fig. 6G). Although these observations were based on a small number of clinically heterogeneous cases, they were consistent with the hypothesis that aberrant EGFR signaling may reduce TEFT responsiveness. Based on this mechanistic rationale, two patients with recurrent EGFR-driven GBM subsequently received osimertinib combined with TEFT as salvage treatment. Case #3, harboring EGFR amplification, presented with recurrence and severe mass effect and showed stable disease with radiological improvement and reduced mass effect after initiation of the combination regimen. Similarly, Case #4, harboring EGFR amplification with EGFRvIII, experienced rapid recurrence following repeat resection but achieved radiological disease stabilization after receiving the salvage combination therapy (Fig. 6G, H).

**Fig. 7:**
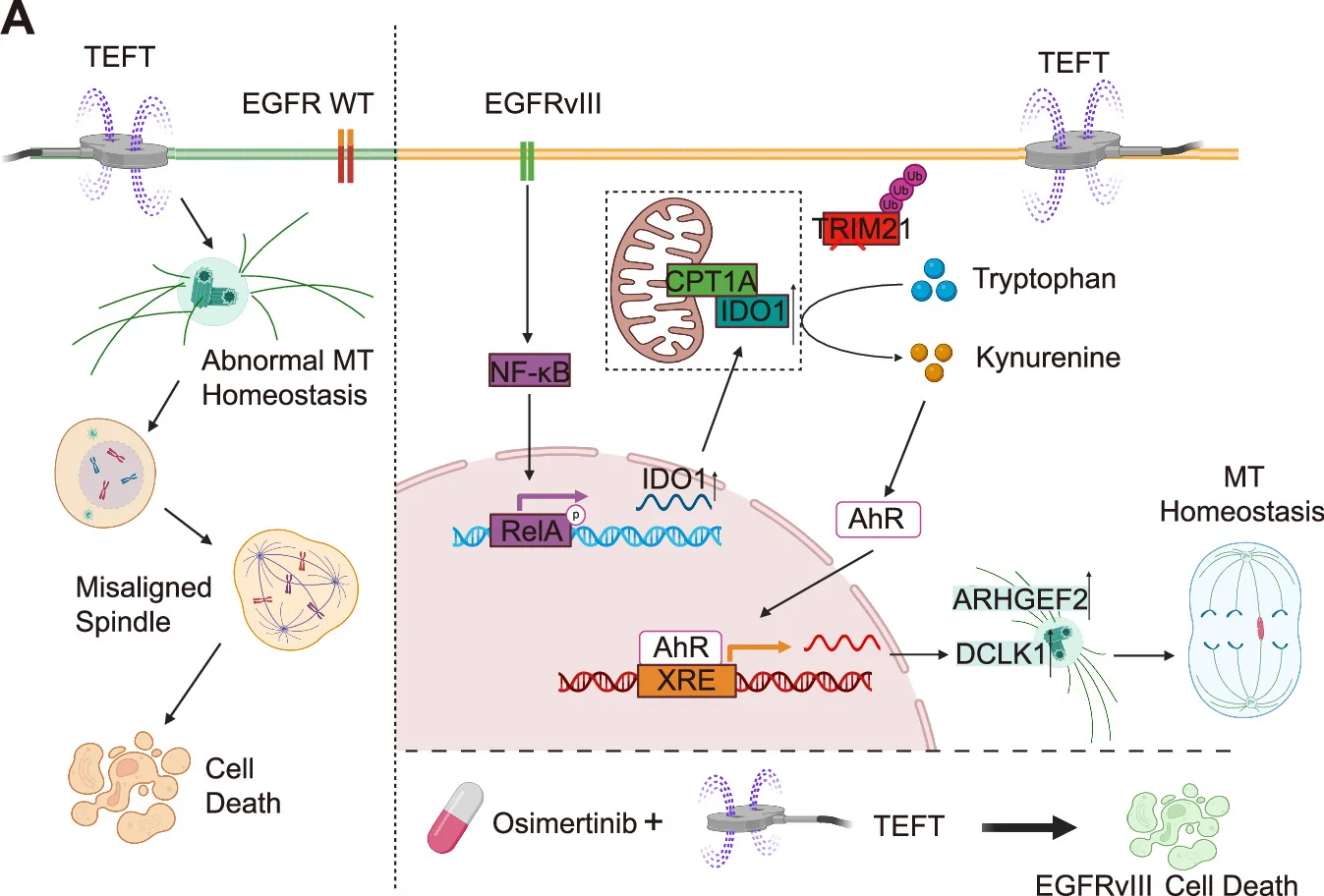
Schematic model of the IDO1-AhR mediated cytoskeletal resistance program. **A** In EGFR wild-type cells, TEFT disrupts microtubule homeostasis, leading to spindle misalignment and cell death. In EGFRvIII cells, NF-κB-dependent *IDO1* transcription and TEFT-induced CPT1A-mediated IDO1 stabilization increase kynurenine production, activate AhR-dependent transcription, and upregulate cytoskeletal regulators, such as DCLK1 and ARHGEF2, thereby preserving microtubule homeostasis and promoting TEFT resistance. Osimertinib blocks the upstream EGFRvIII-NFκB-IDO1 supply axis and resensitizes EGFRvIII-driven cells to TEFT. Created in BioRender. Feng, B. (2026) https://BioRender.com/fvoy6ve.

Collectively, these findings support a resistance mechanism in which EGFRvIII-driven IDO1 stabilization enhances kynurenine production and AhR activation, thereby engaging a cytoskeletal protection program involving DCLK1 and ARHGEF2 (Fig. 7A). Rather than acting as a standalone determinant, the IDO1-AhR axis links EGFRvIII-dependent metabolic and transcriptional adaptation to preservation of mitotic spindle organization and microtubule polymerization under electric-field stress. This mechanism explains how EGFRvIII-driven GBM cells can partially buffer the anti-mitotic effects of TEFT and identifies IDO1/AhR-associated cytoskeletal reinforcement as a therapeutically targetable resistance program. The clinical observations, although limited by the small number of cases and treatment heterogeneity, provide mechanism-informed proof-of-concept support that EGFR inhibition may enhance TEFT responsiveness in EGFR-driven GBM. Together with the orthotopic mouse and patient-derived organoid data, these findings support prospective evaluation of osimertinib plus TEFT as a rational combination strategy for patients with EGFR-driven GBM.

## DISCUSSION

While the updated Stupp regimen combined with TEFT has improved survival in GBM, the heterogeneity of clinical responses remains a critical challenge. Our study identifies EGFRvIII not merely as an oncogenic driver, but as an enabling component within a multi-layered resistance program that couples metabolic plasticity with mitotic fidelity. Rather than indicating that EGFRvIII alone generates all components of this resistance program, our data support a genotype field convergence model. TEFT induces a broadly inducible mitochondrial and bioenergetic stress response that activates the AMPK-PPARα-CPT1A axis, whereas EGFRvIII provides NF-κB-dependent transcriptional priming of *IDO1*. The convergence of these two inputs promotes CPT1A-dependent IDO1 stabilization and subsequent activation of an IDO1-kynurenine-AhR cytoskeletal protection program. This finding bridges the gap between metabolic reprogramming and the physical mechanism of action of electric fields.

The first layer of this resistance is transcriptional. While wild-type EGFR activates NF-κB in a ligand-dependent manner^9,51^, our data indicate that constitutively active EGFRvIII maintains persistent NF-κB/RelA signaling and increases RelA occupancy at the *IDO1* promoter. Mechanistically, this results in increased basal *IDO1* transcription in EGFRvIII-driven cells. Although IDO1 is classically recognized for its role in tryptophan catabolism: depleting essential amino acids to impair T-cell proliferation and generating kynurenine to support immunosuppressive T-cell states^39,52,53^; our findings suggest that, in the context of TEFT, IDO1 also serves an intrinsic tumor-protective role beyond immune evasion. The NF-κB-driven increase in *IDO1* mRNA provides the necessary substrate for subsequent CPT1A-dependent post-translational stabilization, creating a transcriptional supply arm that maintains an IDO1 pool capable of supporting downstream AhR-associated cytoskeletal protection.

Our findings indicate  a counter-intuitive metabolic response triggered by TEFT. We observed that TEFT induces mitochondrial and bioenergetic stress characterized by mitochondrial depolarization, mitochondrial calcium accumulation, and lipid oxidative stress. Instead of succumbing to this stress, tumor cells initiate an active adaptation via the AMPK-PPARα axis to upregulate CPT1A. Crucially, although we initially delineated this pathway in EGFRvIII-mutant cells, the triggering bioenergetic stress is a broadly inducible biophysical consequence of electric-field exposure rather than being specific to the mutation. Parental LN229 and T98G cells also exhibited mitochondrial stress, AMPKα phosphorylation, PPARα-PGC1α engagement, and CPT1A induction following TEFT exposure. This suggests that upregulation of CPT1A-mediated fatty-acid metabolism may represent a common metabolic adaptation employed by diverse GBM cells under TEFT stress, although this upstream response alone is insufficient to establish the complete EGFRvIII–IDO1 resistance cascade. Classically, CPT1A is the rate-limiting enzyme for fatty-acid oxidation (FAO)^54,55^. Under TEFT conditions, CPT1A upregulation may represent a compensatory attempt to maintain mitochondrial substrate flux under reduced energetic efficiency. Consistent with this interpretation, TEFT increased lipid droplet-mitochondrial proximity, neutral lipid accumulation, and lipid oxidative stress. CPT1A is an established outer mitochondrial membrane-resident enzyme, whereas succinyl-CoA is primarily generated within the mitochondrial matrix. Although TEFT did not globally increase total cellular succinyl-CoA, the metabolite pool was not depleted, indicating that donor availability was maintained. We therefore propose a CPT1A-associated mitochondrial microdomain model in which local metabolic organization may permit access to succinyl-CoA or related acyl-donor availability at the outer mitochondrial membrane interface^56,57^. This topological model will require direct submitochondrial metabolite tracing. NAC increased TEFT-induced apoptosis and attenuated CPT1A accumulation, suggesting that redox stress contributes to the upstream metabolic adaptation^58,59^. Crucially, upregulated CPT1A also transcends its canonical metabolic role. Through its LSTase-related activity, CPT1A promotes IDO1 succinylation at K377, which functionally antagonizes ubiquitination at the adjacent K389 degron and limits TRIM21-mediated proteasomal degradation. This reveals a dual-input mechanism: TEFT-induced metabolic stress upregulates CPT1A, whereas EGFRvIII supplies the transcriptionally primed IDO1 substrate. When these inputs converge, the metabolic adaptation induced by TEFT stabilizes the protein that supports downstream resistance, thereby creating a compensatory mechanism that limits therapeutic efficacy.

Our identification of the CPT1A-IDO1 stabilization axis provides an additional framework for considering IDO1 enzymatic inhibitors, including epacadostat^60–62^. While previous strategies have focused on blocking the catalytic pocket^63,64^, our epacadostat experiments indicate that the enzymatic output of IDO1 remains functionally important: pharmacological inhibition increased TEFT-induced apoptosis and impaired microtubule protection, phenocopying key aspects of genetic IDO1 depletion and osimertinib-mediated re-sensitization. At the same time, our findings show that TEFT resistance is also regulated at the level of IDO1 protein abundance through CPT1A-dependent succinylation and stabilization. Therefore, strategies that reduce IDO1 transcription or stability-such as EGFR/NF-κB inhibition or targeting the CPT1A LSTase-related stabilization mechanism- may complement catalytic IDO1 inhibition by limiting both kynurenine production and protein-abundance-dependent downstream signaling.

Downstream of IDO1, our study uncovers a function for the Aryl Hydrocarbon Receptor (AhR). Typically viewed as a sensor for environmental toxins^65^, our data support a role for AhR in linking tryptophan metabolism to cytoskeletal adaptation in EGFRvIII-driven GBM under TEFT stress. Increased kynurenine production, AhR nuclear translocation, and induction of canonical AhR-responsive genes collectively support activation of this pathway. AhR activation is associated with increased transcription of *DCLK1* and *ARHGEF2*, with ChIP-qPCR supporting AhR occupancy at their predicted promoter regions. DCLK1 is a microtubule-associated kinase involved in bipolar spindle formation^44,45^, while ARHGEF2/GEF-H1 regulates RhoA-dependent microtubule dynamics^17,50^. Promoter ChIP-qPCR and functional perturbation experiments support DCLK1 and ARHGEF2 as direct AhR-regulated cytoskeletal effectors in this setting. In the context of TEFT, which physically disrupts dipole alignment and spindle assembly, upregulation of these factors supports spindle organization and microtubule polymerization, thereby helping tumor cells tolerate electric-field-induced mitotic stress. This finding links metabolic inputs, particularly the tryptophan metabolite kynurenine, to physical structural adaptations of the cytoskeleton and provides a mechanistic explanation for how tumor cells can partially buffer the anti-mitotic effects of physical therapy.

Finally, our work provides a strong molecular rationale for combining TEFT with third-generation EGFR-TKIs. Although the upstream AMPK-PPARα-CPT1A response is induced by TEFT independently of EGFRvIII, EGFRvIII-dependent NF-κB signaling supplies the IDO1 substrate required for conversion of this broad metabolic adaptation into cytoskeletal protection. Osimertinib, a third-generation EGFR-TKI with blood-brain barrier penetration^66^, has transformed the management of EGFR-mutant NSCLC^67–69^. However, its application in GBM has been challenging; despite encouraging preclinical data, clinical studies evaluating osimertinib as monotherapy have shown only modest efficacy^70–72^, and have not displaced the standard Stupp regimen. This limited efficacy likely reflects the profound heterogeneity of GBM and rapid activation of compensatory bypass signaling^73–76^. Importantly, our study reframes the potential clinical utility of osimertinib in GBM. Our findings suggest that its primary value in this context may not be as a standalone cytotoxic agent, but as a mechanism-based sensitizer to TEFT. By blocking EGFRvIII signaling, osimertinib suppresses the NF-κB-dependent *IDO1* transcriptional supply, thereby reducing the IDO1 pool available for TEFT-induced CPT1A-mediated stabilization. Osimertinib did not abolish TEFT-induced AMPKα phosphorylation or CPT1A upregulation, indicating that it acts primarily on the EGFR-dependent IDO1 supply arm rather than eliminating the upstream metabolic-stress response. This restores greater biophysical vulnerability of tumor cells to electric fields. Therefore, our findings provide a compelling molecular rationale for combining TEFT with osimertinib. This strategy exploits complementary mechanisms: TEFT exerts physical pressure on the mitotic spindle, whereas osimertinib prevents EGFR-driven tumor cells from efficiently converting metabolic adaptation into IDO1-AhR-dependent cytoskeletal protection. Based on this rationale, our preliminary clinical observations in Cases #3 and #4 provide mechanism-informed proof-of-concept support that osimertinib may enhance TEFT responsiveness in recurrent EGFR-driven GBM. However, these observations remain exploratory because of the small number of patients and treatment heterogeneity and therefore require prospective biomarker-guided validation.

More broadly, we use the term “electric field oncology” to describe a framework in which electric-field-based cancer therapies are investigated through the interaction of biophysical exposure, tumor genotype, adaptive cellular responses and pharmacological sensitization. Our findings illustrate this framework in EGFR-driven GBM, where TEFT-induced biophysical stress converges with oncogenic signaling to generate a targetable resistance program. Whether this framework extends to other tumor types and electric-field modalities will require systematic investigation.

Our study has limitations that point toward critical avenues for future research. First, the association between EGFRvIII status and reduced TEFT benefit was derived from a retrospective single-center cohort, and the clinical observations involving osimertinib plus TEFT were based on a small exploratory case series. These findings are hypothesis-generating and mechanism-informed but cannot establish EGFR alterations as definitive predictive biomarkers or demonstrate clinical efficacy of the combination. Larger prospective, biomarker-guided studies are required. Second, although TEFT-induced mitochondrial stress and AMPK-PPARα-CPT1A activation were observed in both EGFRvIII-driven and parental GBM cells, it remains unknown whether this metabolic program becomes a general mechanism of acquired resistance across molecular subtypes. Longitudinal TEFT exposure models and pan-genotypic patient-derived systems will be needed to determine how broadly this adaptive response applies and whether CPT1A represents a shared therapeutic vulnerability. Third, although we mapped the CPT1A LSTase-related activity, the precise source and submitochondrial accessibility of the succinyl-CoA donor require further biochemical dissection. Although total cellular succinyl-CoA was not depleted under TEFT, direct submitochondrial metabolite tracing will be necessary to test the proposed CPT1A-associated outer mitochondrial membrane microdomain model. Finally, the safety and efficacy of TEFT combined with osimertinib require prospective clinical evaluation, including optimization of osimertinib dose, treatment timing, tolerability, and molecular patient selection.

## METHODS

### Ethics statement

This research complied with all relevant ethical regulations. The retrospective clinical cohort, collection and research use of human glioblastoma specimens, and establishment and use of patient-derived cultures and organoids were approved by the Medical Ethics Committee of Qilu Hospital of Shandong University. Written informed consent was obtained from all participants for the collection and research use of human tissue. Written informed consent for treatment and publication of clinical and imaging information was obtained from the patients who received compassionate-use treatment. All animal experiments were approved by the Ethics Committee On Animal Experiment of Shandong University Qilu Hospital, and were performed in accordance with the approved institutional guidelines. No participant received payment for research participation. The compassionate-use treatment constituted clinical care rather than a clinical trial, and treatment costs were not considered research compensation. Case #3 received humanitarian financial assistance covering the additional drug-related costs; this assistance was independent of research participation and consent for publication.

### Clinical cohorts and patient samples

Retrospective TEFT-GBM Cohort: Patients with histologically confirmed glioblastoma (GBM) treated at the Department of Neurosurgery, Qilu Hospital of Shandong University between 2021 and the data-cutoff date were consecutively and retrospectively enrolled. Inclusion criteria required patients to have completed the standard Stupp protocol followed by tumor electric field therapy with a compliance of ≥18 h/day at a field intensity of 2 V/cm. Clinical data and molecular profiles, including EGFR amplification and EGFRvIII alteration status, were collected and centrally reviewed. Both sexes were eligible, and no participant was selected or excluded on the basis of sex. Sex was extracted from the hospital medical records rather than determined specifically for this study; gender identity was not collected. Sex was reported descriptively but was not included as a stratification factor because the study was not designed or sufficiently powered to assess sex-related differences. Accordingly, no conclusions regarding sex-specific treatment effects are drawn. Two patients with recurrent EGFR-driven GBM received compassionate-use salvage treatment with osimertinib (80 mg, orally once daily) and TEFT. Both patients provided written informed consent for treatment and publication of their clinical data and imaging findings. Treatment response was assessed according to RANO criteria based on longitudinal MRI scans. Human Tissue and Organoids: Primary GBM tissue samples were obtained with written informed consent. Patient-derived organoids (GBOs) were established from recurrent GBM patients. The sex of participants in the exploratory case series is provided in the Supplementary Information.

### Cell lines and cell culture

Human GBM cell lines (U118MG, A172, LN229, and T98G) and HEK293T cells were obtained from the American Type Culture Collection (ATCC). The patient-derived EGFRvIII-positive GBM culture TBD0220 was kindly provided by Professor Chunsheng Kang (Tianjin Medical University). Additional low-passage patient-derived GBM cultures, including LG-260 and LG-252, were established from freshly resected tumor specimens obtained with written informed consent. Briefly, fresh tumor tissues were washed with sterile phosphate-buffered saline, mechanically minced into approximately 0.5–1-mm fragments, and enzymatically dissociated in 3 mL Accutase at 37 °C for approximately 15 min, with gentle trituration during digestion. The resulting cell suspensions were passed through a 70-μm cell strainer, washed with PBS, and collected by centrifugation.

Established GBM cell lines and HEK293T cells were cultured in DMEM supplemented with 10% fetal bovine serum and 1% penicillin-streptomycin. Patient-derived GBM cells were maintained in serum-free NBE medium prepared as an approximately 50-mL formulation containing 48 mL Neurobasal medium (Thermo Fisher Scientific), 1 mL B27 supplement without vitamin A (Thermo Fisher Scientific), 500 μL GlutaMAX supplement (Thermo Fisher Scientific), 500 μL penicillin streptomycin solution (Beyotime; final working concentration, 100 μg/mL), and recombinant human EGF and bFGF (R&D Systems) at final concentrations of 25 ng/mL each. Patient-derived cultures were used between passages 3 and 7 because their viability and growth capacity progressively declined during subsequent passages. The *EGFR* amplification and EGFRvIII statuses of these cultures were determined from the postoperative molecular pathological examination of the corresponding patient tumors. All cells were maintained at 37 °C in a humidified atmosphere containing 5% CO₂.

For EGFR activation, EGFR-wild-type cells were stimulated with EGF (50 ng/mL). EGFRvIII-overexpressing stable cell lines were generated through lentiviral transduction and selected with puromycin.

### Patient-derived glioblastoma specimens and organoids

Fresh GBM specimens were obtained from patients undergoing surgical resection after written informed consent. Tumor tissues not allocated for molecular analysis or histopathological examination were washed with PBS and mechanically dissected into approximately 0.5–1-mm fragments. The tumor fragments were distributed into ultra-low-attachment six-well plates, with each well containing 4 mL of GBO medium.

The GBO medium consisted of 50% DMEM/F12, 50% Neurobasal medium, 1× Glutamax, 1× non-essential amino acids, 1× penicillin-streptomycin, 1× N2 supplement, 1× B27 supplement without vitamin A, 1× 2-mercaptoethanol, and human insulin at a final concentration of 2.5 μg/mL. Plates were maintained on an orbital shaker at 120 rpm in a humidified incubator at 37 °C with 5% CO₂. Approximately 75% of the culture medium was replaced every 48 h by tilting the plates and carefully aspirating the medium above the settled tissue fragments. Tumor fragments generally developed a spherical organoid morphology within 1–2 weeks. Successful GBO establishment was defined by survival for at least 2 weeks, acquisition of a spherical morphology, and continued growth in culture. For prolonged culture exceeding 1 month, organoids were mechanically divided into fragments approximately 200–500 μm in diameter to reduce central necrosis caused by limited nutrient and oxygen diffusion.

### In vitro tumor electric field therapy

The TEFT device used for in vitro experiments was provided by Antai Kangcheng Biotechnology Co., Ltd. GBM cells in the logarithmic growth phase were detached with trypsin, counted, and resuspended in 600 μL of high-glucose DMEM. A total of 6 × 10⁴ cells were seeded onto 20 mm cell culture coverslips and allowed to adhere overnight before being transferred into TEFT culture dishes. For patient-derived glioblastoma organoids (GBOs), established organoids were transferred directly into TEFT culture dishes, which were placed on an orbital shaker at 120 rpm to maintain suspension culture during treatment. TEFT was applied at a frequency of 200 kHz and a field intensity of 1.5 V/cm. The culture temperature was maintained at 37 °C throughout the exposure. TEFT was applied for 72 h unless otherwise indicated. The TEFT culture device contained an integrated active temperature-control module that continuously monitored and regulated the temperature during electric-field exposure. During representative treatment runs, infrared thermography was used to verify that TEFT exposure did not produce focal heat accumulation within the culture region.

### Cell viability and IC_50_ determination

Cell sensitivity to osimertinib was assessed using the Cell Counting Kit-8 (CCK-8) assay. Cells were seeded into 96-well plates at a density of 3 × 10³ cells per well and allowed to adhere overnight. Cells were then treated with serial concentrations of osimertinib in the presence or absence of TEFT applied at 1.5 V/cm and 200 kHz for 72 h. Subsequently, 10 μL of CCK-8 reagent was added to each well, and the plates were incubated at 37 °C for 2 h. Absorbance was measured at 450 nm using a microplate reader, and cell viability was normalized to the untreated control group. Dose-response curves were generated using nonlinear regression, and the half-maximal inhibitory concentration (IC_50_) of osimertinib was calculated separately under control and TEFT conditions using GraphPad Prism software.

Drug-TEFT interactions were further evaluated using the Bliss independence model. For each combination condition, fractional inhibition was calculated from normalized cell viability. The expected Bliss inhibition was calculated as *E*_Bliss_ = *E*_A_ + *E*_B _*- E*_A _× *E*_B_, where *E*_A_ and *E*_B_ represent the fractional inhibition produced by osimertinib and TEFT alone, respectively. The Bliss synergy score was calculated as the observed combination inhibition minus the expected Bliss inhibition. Positive scores indicated synergy, scores close to zero indicated an additive interaction, and negative scores indicated antagonism.

### Apoptosis assay and cell cycle analysis

Treated cells were harvested and washed twice with cold PBS. For apoptosis analysis, cells were resuspended in binding buffer and stained with Annexin V-FITC and propidium iodide (PI) for 15 min in the dark at room temperature according to the manufacturer’s instructions. Early apoptotic cells were defined as Annexin V-FITC-positive/PI-negative, whereas late apoptotic or necrotic cells were defined as Annexin V-FITC-positive/PI-positive. For cell-cycle analysis, cells were harvested and fixed in 70% ice-cold ethanol overnight at 4 °C. The fixed cells were washed with PBS and stained with PI/RNase staining solution for 30 min at 37 °C. Apoptotic populations and cell-cycle distributions, including the G0/G1, S, and G2/M phases, were analyzed using a NovoCyte Quanteon 4020 flow cytometer and Novoexpress software.

### Immunofluorescence and TUNEL assay

Cells, GBOs, or tissue sections were fixed with 4% paraformaldehyde, permeabilized with 0.5% Triton X-100, and blocked with 5% bovine serum albumin (BSA) or normal goat serum, as appropriate. Samples were incubated overnight at 4 °C with primary antibodies against Ki-67, IDO1, CPT1A, AhR, or α-tubulin, followed by incubation with Alexa Fluor-conjugated secondary antibodies. Nuclei were counterstained with DAPI.

For spindle analysis, mitotic spindle morphology was classified as normal bipolar or abnormal, including multipolar or disorganized spindles. At least 25 mitotic cells were randomly evaluated per condition in each independent experiment, and the percentage of abnormal mitotic cells was calculated. For apoptosis assessment, TUNEL staining was performed using the EdUTP TUNEL Apoptosis Assay Kit according to the manufacturer’s instructions, followed by Ki-67 immunofluorescence staining when combined TUNEL/Ki-67 analysis was required.

Conventional confocal images were acquired using a Leica SP8 confocal laser-scanning microscope and analyzed using ImageJ software. For high-resolution IDO1-CPT1A spatial analysis, z-stack images were acquired using an Olympus SpinSR10 super-resolution spinning-disk confocal microscope and reconstructed and analyzed using Imaris software. Colocalization was evaluated qualitatively by three-dimensional spatial overlap and line-scan fluorescence intensity profiles.

### RNA extraction, library construction, and sequencing

Total RNA was extracted from GBM cells (EGFRwt and EGFRvIII) treated with or without TEFT using TRIzol reagent. RNA integrity and concentration were assessed using the Agilent 2100 Bioanalyzer (Agilent Technologies). Messenger RNA (mRNA) was purified from total RNA using poly-T oligo-attached magnetic beads. Fragmentation was carried out using divalent cations under elevated temperature in NEBNext First Strand Synthesis Reaction Buffer (5X). First-strand cDNA was synthesized using random hexamer primers and M-MuLV Reverse Transcriptase, followed by second-strand cDNA synthesis using DNA Polymerase I and RNase H. The library was constructed using the NEBNext® Ultra™ RNA Library Prep Kit for Illumina® (NEB, USA) following the manufacturer’s recommendations. After end repair, A-tailing, and adaptor ligation, the libraries were amplified by PCR and purified using the AMPure XP system (Beckman Coulter). The quality of the libraries was verified by the Agilent 2100 Bioanalyzer and qRT-PCR. Sequencing was performed on an Illumina NovaSeq platform to generate 150 bp paired-end reads.

### ATAC-seq library preparation and sequencing

Chromatin accessibility profiling was performed using the Assay for Transposase-Accessible Chromatin with high-throughput sequencing (ATAC-seq). Briefly, approximately 5 × 10^4^ fresh cells were harvested, washed with cold PBS, and lysed in cold lysis buffer to extract nuclei. The nuclei were resuspended in the Tn5 transposase reaction mixture (Illumina Tagment DNA Enzyme and Buffer Kits) and incubated at 37 °C for 30 min for tagmentation. The fragmented DNA was purified using the MinElute PCR Purification Kit (Qiagen) and amplified by PCR with Nextera primers. The resulting libraries were purified using AMPure XP beads (Beckman Coulter) and quality-controlled using the Agilent 2100 Bioanalyzer. Sequencing was performed on an Illumina platform (PE150 mode).

### Multi-omics analysis

Raw RNA-seq and ATAC-seq reads were processed using fastp for quality control and aligned to the human reference genome (hg38) using HISAT2 (v2.0.5) and Bowtie2 (via ENCODE ATAC-seq Pipeline v1.9.3), respectively. Gene expression was quantified by featureCounts (v1.5.0-p3), while chromatin accessible peaks were called using MACS2. Differential analyses were performed using DESeq2 (v1.20.0) for RNA-seq (adjusted P < 0.05, Log_2_FC > 1) and DiffBind for ATAC-seq (FDR < 0.05, Log_2_FC > 1). Functional enrichment (GO, KEGG, GSEA) was conducted using clusterProfiler (v3.8.1), and motif enrichment was analyzed with HOMER. To identify key drivers, we intersected upregulated DEGs, accessible chromatin regions, and potential targets from the ChIP-Atlas database (AhR/RelA binding). Clinical relevance was validated using TCGA and CGGA datasets via GEPIA2^77^ and GlioVis^78^.

### Quantitative real-time PCR (RT-qPCR)

Total RNA was extracted using an RNA extraction kit (TIANGEN) and reverse-transcribed into cDNA using a reverse transcription kit (Takara) according to the manufacturers’ instructions. Quantitative PCR was performed using SYBR Green Master Mix on a LightCycler 96 System (Roche). Relative mRNA expression was calculated using the 2^−ΔΔCt^ method and normalized to GAPDH. Three independent biological replicates were analyzed for each experiment. The primer sequences are provided in the Supplementary Table 5.

### Plasmids, lentiviral transduction and transfection

The full-length human cDNAs encoding *EGFR*vIII, *CPT1A*, and *IDO1* were PCR-amplified and subcloned into the pCDH-CMV-MCS-EF1-Puro lentiviral vector or the pcDNA3.1 mammalian expression vector. Site-directed mutagenesis was performed using a mutagenesis kit (Vazyme) to generate CPT1A mutants (G710E and H473A) and IDO1 mutants (K366R, K377R, and K389R). All constructs and point mutations were verified by Sanger sequencing. For the generation of stable cell lines, lentiviral particles were produced in HEK293T cells by co-transfecting the indicated expression vectors with the packaging plasmids psPAX2 and pMD2.G. Viral supernatants were collected at 48 and 72 h after transfection, filtered through a 0.45-μm filter, and used to infect GBM cells in the presence of 8 μg/mL polybrene. Infected cells were selected with puromycin for at least 7 days to establish stable cell pools. The puromycin concentration was determined separately for each cell line using prior antibiotic-selection assays. Small interfering RNAs targeting *IDO1*, *TRIM21*, *CPT1A*, *DCLK1*, and *ARHGEF2*, together with the corresponding non-targeting control siRNA, were synthesized and supplied by RiboBio. Cells were transfected using Lipofectamine 3000 (Invitrogen) at the siRNA concentrations recommended by the manufacturer. The siRNA sequences are provided in the Supplementary Table 4. Newly generated plasmids and stable cell lines are available from the corresponding author at dr.ligang@sdu.edu.cn, subject to applicable institutional requirements and a material transfer agreement.

### Chromatin Immunoprecipitation (ChIP)

ChIP assays were conducted using the EZ-Magna ChIP™ A/G Kit (Millipore) according to the manufacturer’s instructions. Briefly, cells were cross-linked with 1% formaldehyde, quenched with glycine, lysed, and sonicated to generate DNA fragments of 200-1000 bp. Chromatin was immunoprecipitated overnight at 4°C with antibodies against RelA, AhR, or PPARα. The immune complexes were sequentially washed with Low Salt, High Salt, LiCl, and TE buffers, followed by elution and reverse cross-linking with Proteinase K at 62 °C. Purified DNA was analyzed by qPCR using primers specific for the promoter regions of *IDO1*, *CPT1A*, *DCLK1*, and *ARHGEF2*. Enrichment was calculated relative to input DNA compared with IgG. The ChIP-qPCR primer sequences are provided in Supplementary Table 6.

### Co-Immunoprecipitation (Co-IP) and Western blotting

Interactions were analyzed using the Pierce™ Direct Magnetic IP/Co-IP Kit (Thermo Scientific). To eliminate antibody heavy/light chain interference, specific antibodies (anti-IDO1 and anti-PPARα) were covalently coupled to NHS-activated magnetic beads. Cell lysates were incubated with the antibody-coupled beads overnight at 4 °C. Immune complexes were eluted, neutralized, and prepared for immunoblotting. For western blotting, proteins were separated by SDS-PAGE and transferred to PVDF membranes. After blocking, membranes were incubated overnight at 4 °C with primary antibodies. Blots were probed with HRP-conjugated secondary antibodies and visualized using an ECL detection system. Band intensities were quantified using ImageJ. For mitochondrial-fraction Co-IP, IDO1 was immunoprecipitated from purified mitochondrial fractions, and the precipitates were immunoblotted for CPT1A.

### Liquid chromatography-tandem mass spectrometry (LC-MS/MS)

Three qualitative LC-MS/MS experiments comprising six samples were performed: an IDO1 immunoprecipitation (IP) experiment without TEFT (IDO1 IP and IgG control), an IP experiment after TEFT treatment (IDO1 IP, α-tubulin IP and IgG control), and an IDO1 gel-band analysis for post-translational modification mapping. Each sample represented one independent biological preparation analyzed by one LC-MS/MS injection (biological n = 1; technical *n* = 1). The mass-spectrometry experiments were used for qualitative candidate discovery rather than quantitative statistical inference. No samples were pooled across experiments, and no quantitative comparisons were made between samples analyzed in different LC-MS/MS batches.

For both IP-MS experiments, immunoprecipitated proteins were reduced with DTT, alkylated with iodoacetamide and digested with trypsin overnight at 37 °C. The resulting peptides were acidified, desalted using C18 material and separated using a 60-min acetonitrile gradient. LC-MS/MS was performed in data-dependent acquisition mode using either a Vanquish Neo UHPLC system coupled to a Q Exactive HF-X Orbitrap mass spectrometer (Thermo Fisher Scientific) for the two-sample experiment or an L-3000 LC system (RIGOL) coupled to a Q Exactive HF-X mass spectrometer for the three-sample experiment. Full MS scans were acquired over m/z 360–1350 at a resolution of 60,000 or over m/z 350–1500 at a resolution of 120,000, respectively, followed by HCD MS/MS at a resolution of 15,000. For IDO1 modification mapping, the excised gel band was reduced, alkylated and subjected to in-gel tryptic digestion. Peptides were analyzed on the same L-3000/Q Exactive HF-X platform using a 30-min gradient and the acquisition settings described above.

Raw data were searched using Proteome Discoverer v3.1 or v2.4 against the UniProt human proteome database or the IDO1 target sequence. Searches allowed two missed tryptic cleavages (three for modification mapping), with carbamidomethylation of cysteine as a fixed modification and methionine oxidation and protein N-terminal acetylation as variable modifications. Lysine succinylation and GlyGly modification of lysine were additionally included for IDO1 modification mapping. Peptide- and protein-level false discovery rates were controlled at <1%. The experiments were used for qualitative candidate identification. Candidate interactions and modifications were subsequently validated by immunoprecipitation, immunoblotting and functional assays.

### Mitochondria isolation, purification, and quality control

Mitochondria were isolated using the Mitochondria Isolation Kit for Cultured Cells (Thermo Scientific) according to the manufacturer’s instructions. Briefly, approximately 2 ×  10⁷ cells were lysed using Reagents A and B, followed by differential centrifugation at 700 × g to remove nuclei and cellular debris and at 12,000 × g to pellet the mitochondrial fraction. For Western blotting, mitochondrial pellets were lysed in RIPA buffer supplemented with protease and phosphatase inhibitors. The purity of the mitochondrial fraction was assessed by immunoblotting for ATP5A as a mitochondrial marker and for Calnexin and Na⁺/K⁺-ATPase as endoplasmic reticulum and plasma membrane contamination markers, respectively. Equal amounts of protein from the mitochondrial fractions were used for subsequent immunoprecipitation.

For flow cytometric analysis, intact mitochondrial pellets were resuspended in PBS containing 2% BSA, fixed and permeabilized, and subsequently stained with antibodies against ATP5A, Calnexin, IDO1, and CPT1A. The mitochondrial population was identified as ATP5A-positive and Calnexin-negative, with unstained and single-stained controls used to define gating and fluorescence compensation. IDO1 and CPT1A signals were quantified within the gated mitochondrial population.

### Nuclear and cytoplasmic protein fractionation

Nuclear and cytoplasmic proteins were separated using a Nuclear and Cytoplasmic Protein Extraction Kit (Beyotime) according to the manufacturer’s instructions. Briefly, treated cells were washed with ice-cold PBS, collected, and resuspended in cytoplasmic protein extraction reagent A supplemented with 1 mM PMSF. After incubation on ice for 10–15 min, cytoplasmic protein extraction reagent B was added, and the samples were vortexed and centrifuged at 12,000–16,000 × g for 5 min at 4 °C. The resulting supernatants were collected as the cytoplasmic fractions. The remaining nuclear pellets were washed to remove residual cytoplasmic extract and resuspended in nuclear protein extraction reagent supplemented with 1 mM PMSF. Samples were incubated on ice for 30 min with intermittent vortexing and then centrifuged at 12,000–16,000 × g for 10 min at 4 °C. The supernatants were collected as the nuclear fractions. Protein concentrations were determined using a BCA assay, and equal amounts of protein were subjected to Western blotting to assess AhR distribution in the cytoplasmic and nuclear fractions. Fraction quality was evaluated using the corresponding cytoplasmic and nuclear protein markers.

### Mitochondrial function analysis

Mitochondrial membrane potential was assessed using TMRM (100 nM; Invitrogen) or a JC-1 Assay Kit (Beyotime) according to the manufacturers’ instructions, followed by flow cytometric analysis. For TMRM staining, the mean fluorescence intensity was quantified, with decreased fluorescence indicating mitochondrial depolarization. For JC-1 staining, mitochondrial membrane potential was evaluated by calculating the ratio of red fluorescence from JC-1 aggregates to green fluorescence from JC-1 monomers.

Mitochondrial calcium accumulation was assessed using Rhod-2 AM (Beyotime). After staining, cells were washed and incubated to allow complete intracellular de-esterification of the probe. Rhod-2 fluorescence was measured by flow cytometry, and the mean fluorescence intensity was used to quantify changes in mitochondrial calcium levels.

### Lipid metabolism and peroxidation analysis

Neutral lipid accumulation was assessed by staining cells with Nile Red (2 μg/mL; MedChemExpress) for 15 min, followed by flow cytometric analysis in the FL2 channel. Lipid peroxidation was evaluated using Liperfluo (Dojindo), a fluorescent probe specific for lipid peroxides. A 1 mM Liperfluo stock solution was prepared in DMSO and protected from light. Cells were stained with Liperfluo according to the manufacturer’s instructions, and fluorescence was measured by flow cytometry. The mean fluorescence intensity was used to quantify cellular lipid peroxide accumulation.

### Surface plasmon resonance (SPR) and structural modeling

The binding affinity between recombinant CPT1A and IDO1 proteins was measured using a Biacore T200 system (GE Healthcare). CPT1A was immobilized on a CM5 sensor chip, and varying concentrations of IDO1 were flowed over the chip surface. The dissociation constant was calculated using the BIAevaluation software. Protein-protein interaction interfaces, specifically the docking of CPT1A and IDO1, were predicted using AlphaFold3. The model was analyzed to identify key contact residues involved in the occlusion of the IDO1 ubiquitination site (K389).

### In vitro tubulin polymerization assay

Tubulin polymerization kinetics were monitored using the Tubulin Polymerization Assay Kit (BK006P, Cytoskeleton, Inc.) according to the manufacturer’s protocol. Briefly, porcine brain tubulin was resuspended in G-PEM buffer containing GTP and glycerol. Cell lysates (10 μg) from TEFT or drug-treated groups were added to the tubulin reaction mixture (final concentration 3 mg/mL) in a pre-warmed 96-well plate. The polymerization kinetics were monitored immediately by measuring the absorbance at 340 nm every 30 s for 60 min at 37 °C using a kinetic microplate reader. The maximum polymerization rate (*V*_max_) was calculated to quantify microtubule stability.

### Kynurenine and succinyl-CoA quantification

Kynurenine concentrations in culture supernatants were quantified using a competitive Kynurenine ELISA Kit (EU0188; FineTest) according to the manufacturer’s instructions. Culture supernatants were collected after the indicated treatments and clarified by centrifugation to remove cells and debris. The clarified supernatants and standards were assayed in duplicate. Absorbance was measured at 450 nm using a TECAN microplate reader. Kynurenine concentrations were calculated from a four-parameter logistic standard curve.

Total cellular succinyl-CoA was quantified using an SCoA ELISA Kit (Fusheng, Shanghai) according to the manufacturer’s instructions. Cells were lysed using a non-denaturing lysis buffer to minimize interference with the assay, and the lysates were clarified by centrifugation. Samples and standards were analyzed in duplicate. Absorbance was measured at 450 nm with 630 nm used as the reference wavelength on a TECAN microplate reader. The mean optical density of duplicate wells was corrected by subtracting the blank value, and succinyl-CoA concentrations were calculated using a four-parameter logistic standard curve.

### Cell-free IDO1 succinylation assay

To assess CPT1A-mediated IDO1 succinylation in a cell-free system, purified recombinant IDO1 was incubated with purified recombinant CPT1A-WT or the catalytically inactive CPT1A-H473A mutant. Bovine serum albumin (BSA) was included as a negative protein control. Reactions were performed in succinylation buffer containing 50 mM Tris-HCl (pH 8.0), 50 mM KCl, 5% glycerol, 0.1 mM EDTA, 1 mM dithiothreitol, 1 mM PMSF, and 10 mM sodium butyrate. Succinyl-CoA was added as the acyl donor, and the reaction mixtures were incubated at 37 °C for 10 min.

To examine the dependence of IDO1 succinylation on succinyl-CoA availability, purified CPT1A-WT and IDO1 were incubated with increasing concentrations of succinyl-CoA (0, 20, 100, 200, 500, and 1000 μM) under the same reaction conditions. Reactions were terminated by adding SDS sample buffer, followed by SDS-PAGE. Coomassie Brilliant Blue staining was performed to verify the presence and comparable loading of the recombinant CPT1A and IDO1 proteins. IDO1 succinylation was assessed by immunoblotting using an anti-succinyl-lysine antibody. Immunoblotting for CPT1A and IDO1 was performed in parallel to confirm the protein input in each reaction.

### Orthotopic xenograft mouse model

Six-week-old male BALB/c nude mice were stereotactically implanted with 5 × 10⁵ luciferase-labeled TBD0220 or LN229-EGFRvIII cells into the right striatum. Only male mice were included. Sex was therefore not included as a factor in the statistical analyses, and the study was not designed to assess sex-specific effects. Accordingly, no conclusions regarding sex differences are drawn. One week after implantation, tumor establishment was confirmed by bioluminescence imaging using an IVIS Spectrum system. For each orthotopic model, mice were randomly assigned to four treatment groups (*n* = 5 per group): vehicle control, TEFT alone, osimertinib alone, and TEFT plus osimertinib.  This corresponded  to 40 mice in total. Exact numbers analyzed for individual endpoints are provided in the corresponding figure legends and Source Data. TEFT was delivered through two pairs of insulated electrode arrays affixed to the shaved scalp using conductive gel sheets. The electric field was applied continuously throughout the treatment period at 2 V/cm and 200 kHz. Electrode attachment and the general condition and mobility of the mice were routinely monitored, and the electrode arrays were inspected, maintained, and replaced or repositioned every 2–3 days as necessary. Osimertinib was administered by oral gavage at 5 mg/kg once daily, whereas mice in the corresponding control groups received vehicle. Tumor burden was monitored weekly by bioluminescence imaging, and survival was recorded until the predefined humane endpoint. The maximal tumor burden permitted under the approved animal protocol was defined by the predetermined humane endpoints, including a loss of 20% or more of the initial body weight, severe or progressive neurological impairment, inability to access food or water, or a moribund condition. Animals were euthanized immediately upon reaching any of these criteria. The maximal permitted tumor burden was not exceeded in any experiment.

### Statistics and reproducibility

Statistical analyses were performed using GraphPad Prism 9.0. Quantitative data are presented as mean ± SD or mean ± SEM, as specified in the corresponding figure legends. Unless otherwise stated, n denotes the number of independent biological replicates. Comparisons between two independent groups were performed using two-sided unpaired Student’s t-tests. When equal variances were not assumed, two-sided unpaired Welch’s t-tests were used. Comparisons among multiple groups were performed using one-way ANOVA. Experiments involving two experimental factors were analyzed using two-way ANOVA. The statistical test and multiple-comparison procedure used for each experiment are specified in the corresponding figure legend. Survival curves were estimated using the Kaplan-Meier method and compared using the two-sided log-rank (Mantel-Cox) test. Univariate and multivariate Cox proportional-hazards regression models were used for clinical survival analyses, with hazard ratios and 95% confidence intervals reported where applicable. All statistical tests were two-sided, and P < 0.05 was considered statistically significant. Exact P values, including multiplicity-adjusted P values where applicable, are provided whenever available. When GraphPad Prism displayed very small P values only using threshold notation, these values are reported as P < 0.001. Source data are provided as a Source Data file.

Details of the antibodies, reagents and kits used in this study are provided in Supplementary Tables 1-3, respectively. Unless otherwise stated, experiments supporting the reported findings were independently performed three times with comparable results using independently prepared biological samples. Pilot experiments conducted during assay optimization and exploratory evaluation were not included in the count of independent confirmatory repetitions. Representative images shown in the main and Supplementary Figs. were selected from these confirmatory experiments. Uncropped and unprocessed blot and gel images are provided in the Source Data file.

### Reporting summary

Further information on research design is available in the Nature Portfolio Reporting Summary linked to this article.

## Supplementary information

Supplementary Information

Reporting Summary

Transparent Peer Review file

## Source data

Source Data

## Data Availability

The RNA-seq and ATAC-seq data generated in this study have been deposited in the Genome Sequence Archive^79^ under accession code PRJCA057933. The mass-spectrometry proteomics data generated in this study have been deposited in the iProX/ProteomeXchange^80^ repository under accession code IPX0015664000. The publicly available datasets analyzed in this study include the TCGA-GBM and TCGA-LGG datasets^24^, the GTEx Analysis Release V8 dataset (dbGaP accession phs000424.v8.p2)^25^, and the CGGA dataset^26^. These data were analyzed through GEPIA2^77^ and GlioVis^78^. Public AHR target-gene data were obtained from ChIP-Atlas 3.0^40^ and GTRD^41^. Source data are provided with this paper. The de-identified patient information provided in the main manuscript and Supplementary Information is limited to sex, a brief clinical history, routine histopathological findings, the categorical molecular status of *IDH*, *EGFR*, *MGMT* promoter and 1p/19q co-deletion, and selected magnetic resonance imaging findings necessary to support the reported conclusions. No direct identifiers are included. Detailed patient-level molecular pathology sequencing results, including comprehensive variant-level reports beyond the reported categorical molecular markers, are available only under restricted access because they contain sensitive individual genomic information and unrestricted public disclosure is not permitted by the applicable participant consent, institutional ethical approval and patient-privacy requirements. These detailed data are therefore not included in the Source Data file or deposited in a public repository. Access may be granted to qualified researchers affiliated with recognized research or healthcare institutions for research purposes compatible with the original consent and ethical approval. Requests should include a research proposal and, where applicable, evidence of ethics approval, and should be directed to the corresponding author at dr.ligang@sdu.edu.cn. Approved requesters must sign a data-use agreement restricting use to the approved purpose and prohibiting participant re-identification and onward distribution. Requests will be acknowledged within 10 working days, and a decision will normally be provided within 30 calendar days. Approved access will be provided through a secure data-transfer mechanism for 12 months, with extensions subject to renewed institutional approval. No directly identifying patient information will be shared. Source data are provided with this paper.
