# Supplementary material for: CPT1A-mediated IDO1 succinylation shapes EGFRvIII-driven resistance to tumor electric field therapy in glioblastoma
Source: Nat Commun. 2026 Sep 23;17:10277. doi: 10.1038/s41467-026-77927-w (PMC13620138; doi:10.1038/s41467-026-77927-w)
Supplement: Supplementary file 1 [file 41467_2026_77927_MOESM1_ESM.pdf]

CPT1A-mediated IDO1 Succinylation Shapes EGFRvIII-driven Resistance to Tumor Electric Field Therapy in Glioblastoma

Author: Bowen Feng[1,2,3]#, Boyan Li[1,2,3]#, Qinran Zhang[1,2,3]#, Daiyan Wang[1,2,3], Weiyang Ma[1,2,3], Qingtong Wang[1,2,3], Huize Xia[1,2,3], Rongrong Zhao[1,2,3], Hongyu Zhao[1,2,3], Kailiang Zhang[1,2,3], Shaobo Wang[1,2,3], Xinglong Zhang[1,2,3], Chuanzheng Wang[1,2,3], Leyang Hao[1,2,3], Yanhua Qi[1,2,3], Zijie Gao[1,2,3], Ziwen Pan[1,2,3], Jiawei Qiu[1,2,3], Zhe Han[1,2,3], Junzhi Liu[1,2,3], Fangyong Wang[4,5,6,7]*, Gang Li[1,2,3]*, Hao Xue[1,2,3]*

# These authors contributed equally.

*Corresponding authors

Correspondence to Fangyong Wang (email: wfybeijing@163.com), Gang Li (email: dr.ligang@sdu.edu.cn), or Hao Xue (email: xuehao@sdu.edu.cn).

List of Supplementary Information

Fig S1 to S12 for multiple supplementary figures.

Supplementary Note for detailed clinical histories of patients in the exploratory case series.

Tables S1 to S6 for multiple supplementary tables.

Supplementary Information

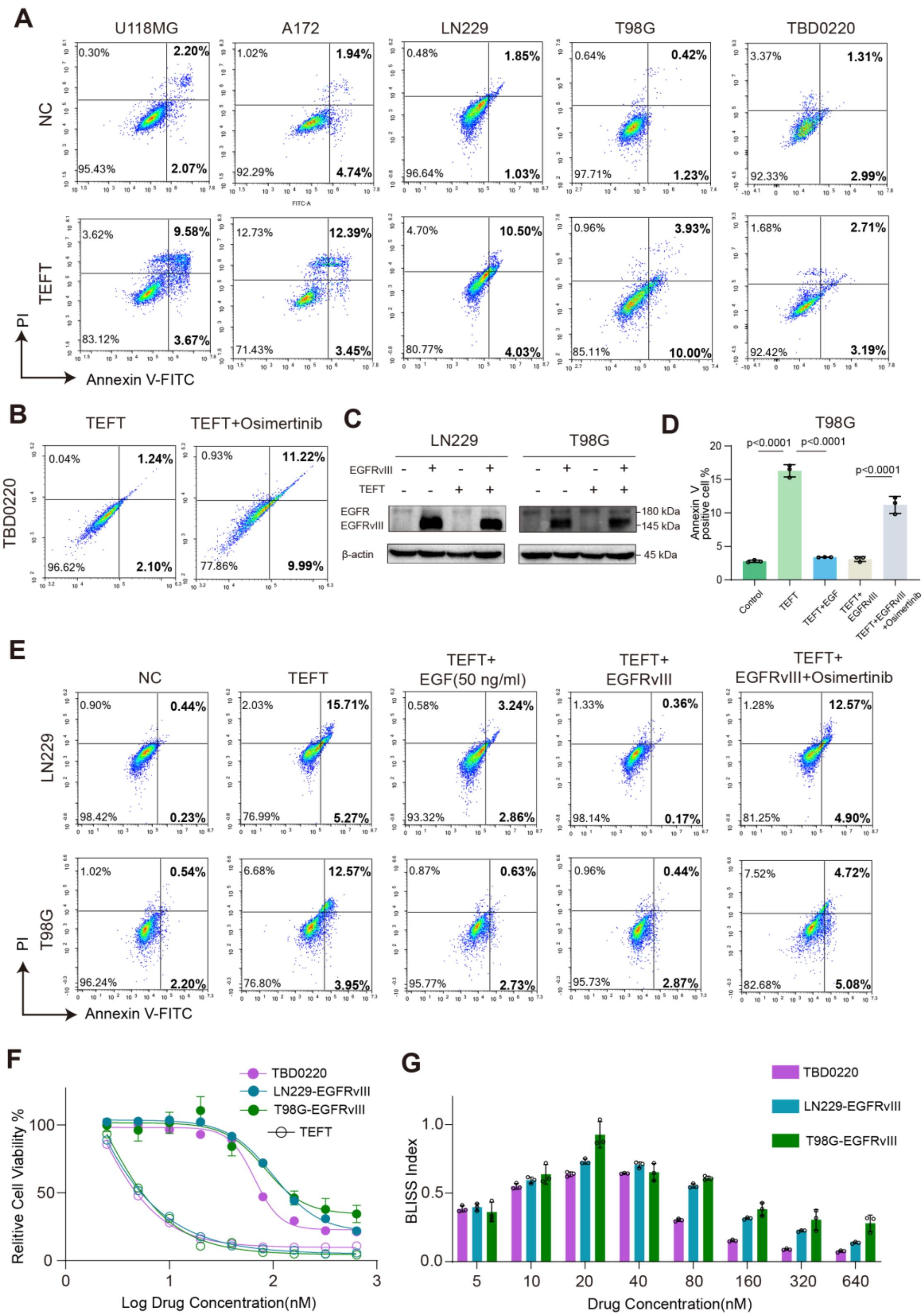

fig. S1. EGFR activation reduces TEFT sensitivity and increases the response to osimertinib. (A) Representative flow cytometry plots of Annexin V/PI staining in

U118MG, A172, LN229, T98G, and TBD0220 cells treated with or without TEFT. The percentage of apoptotic cells (Annexin V+) corresponds to the quantification shown in Fig. 1D, **(B)** Representative flow cytometry plots of Annexin V/PI staining in TBD0220 cells treated with TEFT alone or TEFT plus osimertinib. These plots correspond to the quantification shown in Fig. 1E, **(C)** Western blot analysis validating the expression of EGFR and EGFRvIII in LN229 and T98G cells transduced with EGFRvIII or empty vector, with or without TEFT treatment, **(D)** Flow cytometric quantification of apoptosis in T98G cells under the indicated conditions: control, TEFT alone, TEFT plus EGF stimulation, TEFT plus EGFRvIII transduction, and TEFT plus EGFRvIII with osimertinib (n = 3 biological replicates per group), **(E)** Representative flow cytometry plots of Annexin V/PI staining in LN229 and T98G cells under different treatment conditions: NC, TEFT, TEFT + EGF (50 ng/ml), TEFT + EGFRvIII, and TEFT + EGFRvIII + Osimertinib. These plots correspond to the quantification shown in Fig. 1F and fig. S1D, **(F)** Dose-response viability curves of TBD0220, LN229-EGFRvIII, and T98G-EGFRvIII cells treated with increasing concentrations of osimertinib in the presence or absence of TEFT. TEFT shifted the osimertinib dose-response curves toward lower concentrations, indicating enhanced drug sensitivity under TEFT exposure (n = 3 biological replicates per group), **(G)** Bliss synergy analysis of osimertinib and TEFT in TBD0220, LN229-EGFRvIII, and T98G-EGFRvIII cells across the indicated osimertinib concentrations. Positive Bliss index values support a synergistic interaction between osimertinib and TEFT (n = 3 biological replicates per group). Data are presented as mean ± SD. The exact n values and definitions of biological replicates are specified for the relevant panels. Ordinary one-way ANOVA followed by two-sided Šídák-adjusted multiple-comparisons tests for panel **D**. Exact P values, adjusted for multiple comparisons where applicable, are shown in the corresponding panels. Source data are provided as a Source Data file.

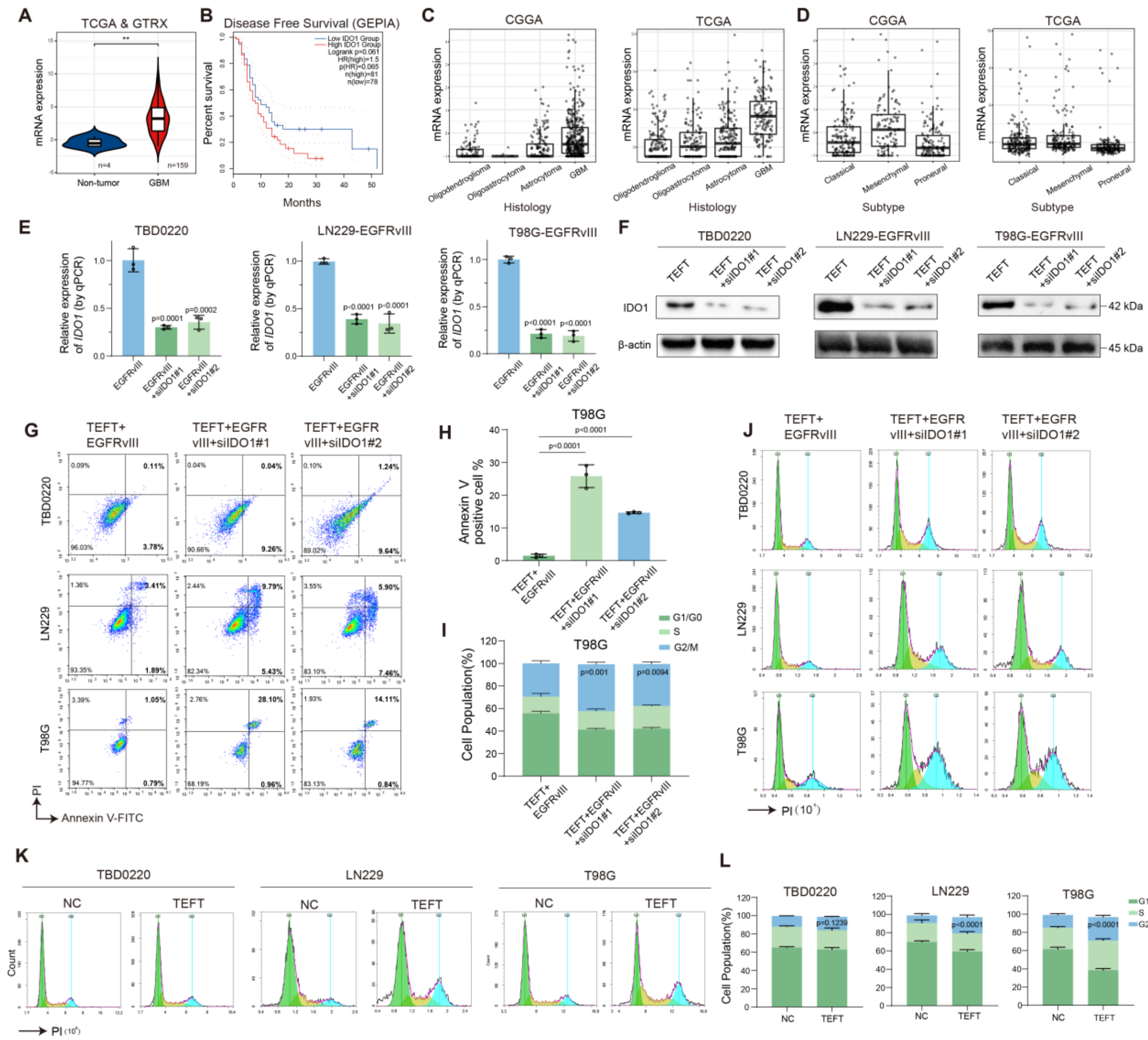

fig. S2. IDO1 expression in glioblastoma models. **(A)** *IDO1* mRNA expression in GBM tissues (n=159) compared to non-tumor tissues (n=4) from the TCGA and GTEx databases, **(B)** Kaplan-Meier analysis of disease-free survival (DFS) based on *IDO1* expression levels in GBM patients (GEPIA database), **(C)** *IDO1* mRNA expression levels across different glioma histological subtypes (Oligodendroglioma, Oligoastrocytoma, Astrocytoma, GBM) in the CGGA (left) and TCGA (right) datasets. IDO1 is significantly upregulated in GBM, **(D)** *IDO1* mRNA expression levels across different molecular subtypes (Classical, Mesenchymal, Proneural) in the CGGA (left) and TCGA (right) datasets, **(E)** RT-qPCR validation of *IDO1* knockdown efficiency in TBD0220, and EGFRvIII-overexpressing LN229 and T98G cells transfected with two independent siRNAs (si*IDO1*#1, si*IDO1*#2) (n = 3 biological replicates per group), **(F)** Western blot validation of IDO1 knockdown efficiency at the protein level in TBD0220, LN229 and T98G cells, **(G)** Representative flow cytometry plots of Annexin V/PI staining showing that *IDO1* knockdown sensitizes EGFRvIII-driven cells to TEFT-induced apoptosis, **(H)** Quantification of Annexin V-positive T98G-EGFRvIII cells after TEFT treatment with or without *IDO1* knockdown (n = 3 biological replicates per group), **(I)** Quantification of cell cycle distribution in T98G-EGFRvIII cells after TEFT treatment with or without

IDO1 knockdown (n = 3 biological replicates per group), **(J)** Representative flow cytometry histograms of cell cycle analysis showing that *IDO1* knockdown restores TEFT-induced G2/M accumulation in EGFRvIII-driven cells, **(K and L)** Representative flow cytometry histograms **(K)** and quantification **(L)** of cell cycle distribution in TBD0220, LN229 and T98G cells treated with or without TEFT. TEFT induced G2/M accumulation in TEFT-sensitive LN229 and T98G cells, whereas this effect was less pronounced in TBD0220 cells (n = 3 biological replicates per group). Data are presented as mean ± SD. The exact n values and definitions of biological replicates are specified for the relevant panels. Statistical tests were as follows: two-tailed unpaired Student's t-test for panel **L**; ordinary one-way ANOVA followed by two-sided Šídák-adjusted multiple-comparisons tests for panels **E**, **H** and **I**. Exact P values, adjusted for multiple comparisons where applicable, are shown in the corresponding panels. Source data are provided as a Source Data file.

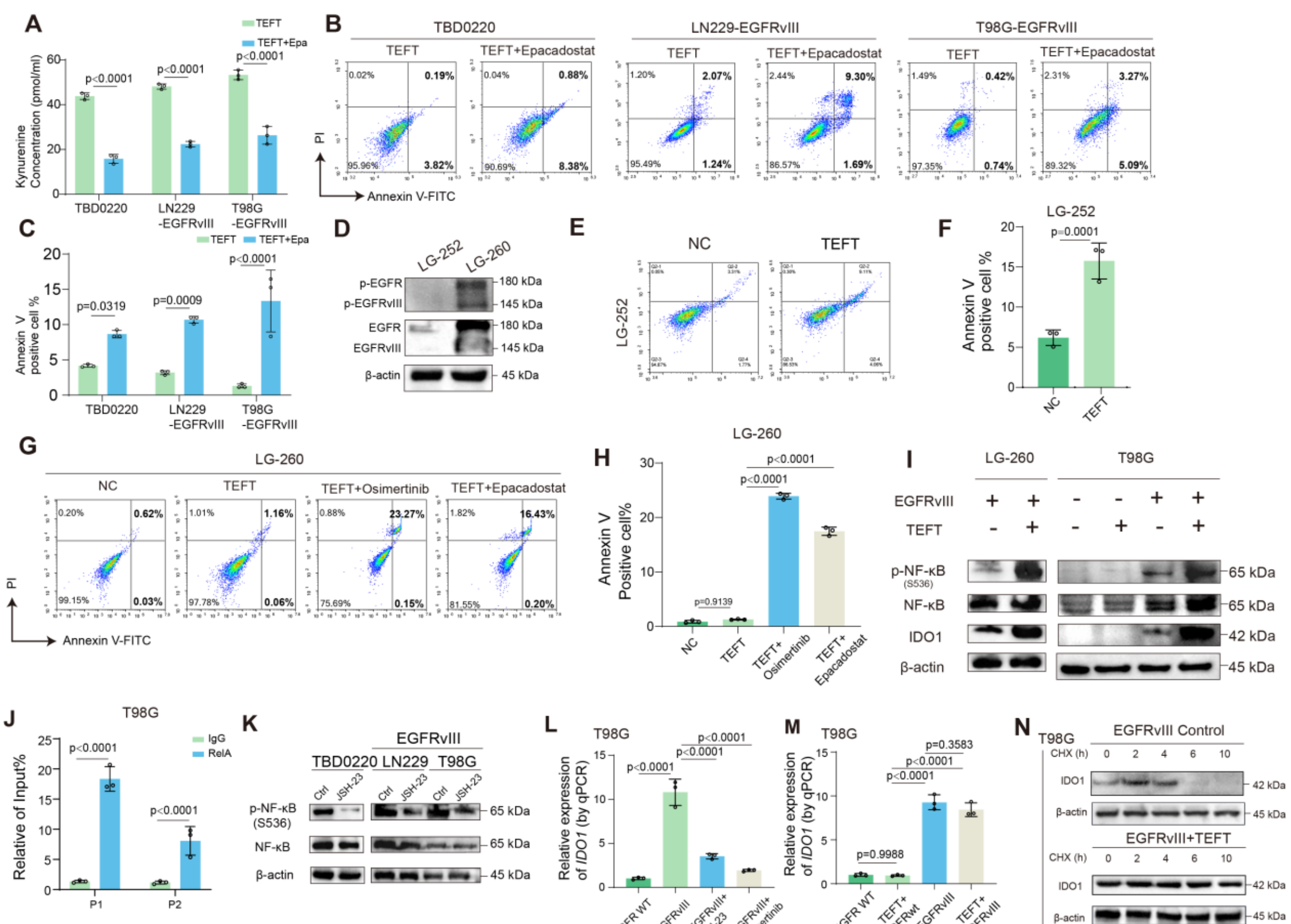

fig. S3. Pharmacological IDO1 inhibition and NF-κB-dependent regulation of IDO1 under TEFT. (A) Kynurenine concentrations in culture supernatants from TBD0220, LN229-EGFRvIII and T98G-EGFRvIII cells treated with TEFT alone or TEFT plus epacadostat (Epa), measured using a competitive ELISA (n = 3 biological replicates per group), **(B)** Representative Annexin V/PI flow cytometry plots showing that epacadostat increases TEFT-induced apoptosis in TBD0220, LN229-EGFRvIII, and T98G-EGFRvIII cells, **(C)** Quantification of Annexin V-positive cells from panel B (n = 3 biological replicates per group), **(D)** Western blot analysis of EGFR/EGFRvIII expression in patient-derived LG-252 and LG-260 cells, **(E and F)** Representative Annexin V/PI flow cytometry plots and

quantification showing that patient-derived LG-252 cells are sensitive to TEFT-induced apoptosis (n = 3 biological replicates per group), **(G and H)** Representative Annexin V/PI flow cytometry plots and quantification in patient-derived LG-260 cells under the indicated conditions. (n = 3 biological replicates per group), **(I)** Western blot analysis of p-NF-κB, total NF-κB, and IDO1 in the indicated EGFRvIII-driven cells under TEFT treatment, **(J)** ChIP-qPCR analysis of RelA occupancy at the predicted IDO1 promoter regions P1 and P2 in T98G-EGFRvIII cells (n = 3 biological replicates per group), **(K)** Western blot analysis confirming inhibition of NF-κB phosphorylation by JSH-23 in different cells, **(L)** RT-qPCR analysis of IDO1 mRNA expression in T98G cells under EGFRvIII activation and treatment with JSH-23 or osimertinib (n = 3 biological replicates per group), **(M)** RT-qPCR analysis of IDO1 mRNA expression in T98G cells treated with or without TEFT (n = 3 biological replicates per group), **(N)** Cycloheximide (CHX) chase assay showing IDO1 protein degradation kinetics in T98G-EGFRvIII cells treated with or without TEFT. Data are presented as mean ± SD. The exact n values and definitions of biological replicates are specified for the relevant panels. Statistical tests were as follows: two-tailed unpaired Student's t-test for panels **F** and **J**; ordinary one-way ANOVA followed by two-sided Šídák-adjusted multiple-comparisons tests for panels **H**, **L** and **M**, and two-way repeated-measures ANOVA followed by two-sided Šídák-adjusted multiple-comparisons tests for panel **A** and **C**. Exact P values, adjusted for multiple comparisons where applicable, are shown in the corresponding panels. Source data are provided as a Source Data file. Abbreviations: Epa, epacadostat.

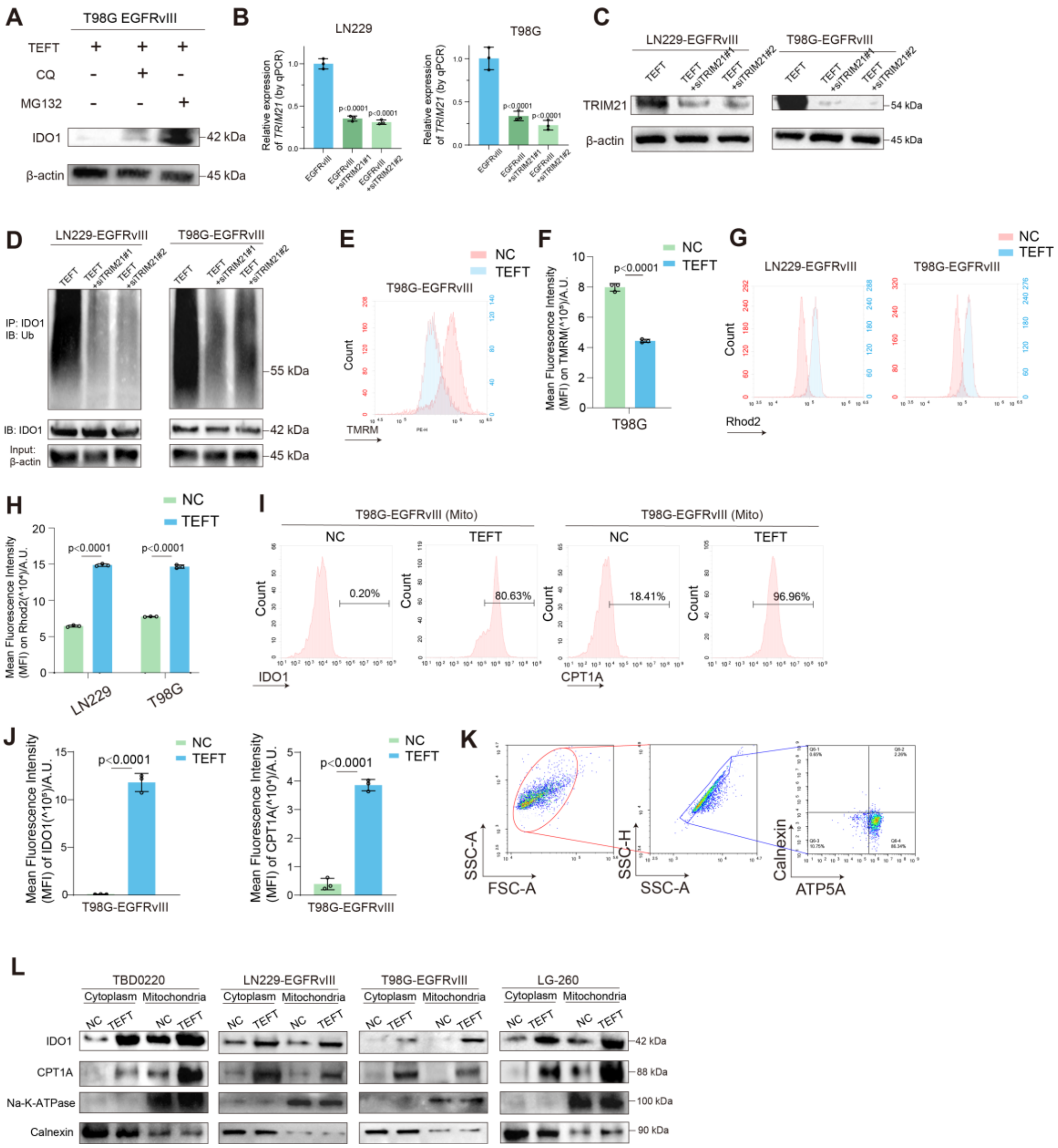

fig. S4. TRIM21-dependent IDO1 turnover and mitochondrial redistribution under TEFT. **(A)** Western blot analysis of IDO1 protein levels in TEFT-treated T98G-EGFRvIII cells following treatment with CQ or MG132. **(B)** RT-qPCR validation of TRIM21 knockdown efficiency in LN229-EGFRvIII and T98G-EGFRvIII cells transfected with two independent siRNAs targeting *TRIM21* (n = 3 biological replicates per group), **(C)** Western blot validation of TRIM21 knockdown efficiency at the protein level in LN229-EGFRvIII and T98G-EGFRvIII cells, **(D)** Immunoprecipitation analysis of IDO1 ubiquitination in LN229-EGFRvIII and T98G-EGFRvIII cells, **(E)** Representative flow cytometry histogram of mitochondrial membrane potential, assessed by TMRM staining, in T98G-EGFRvIII cells treated with or without TEFT, **(F)** Quantification of TMRM mean fluorescence intensity in T98G-EGFRvIII cells treated with or without TEFT (n = 3 biological replicates per group), **(G)** Representative flow cytometry histograms of Rhod-2 staining in LN229-EGFRvIII and T98G-EGFRvIII cells treated with or without TEFT,

indicating mitochondrial calcium accumulation, **(H)** Quantification of Rhod-2 mean fluorescence intensity in LN229-EGFRvIII and T98G-EGFRvIII cells (n = 3 biological replicates per group), **(I)** Flow cytometric analysis of isolated mitochondrial fractions stained for IDO1 and CPT1A in T98G-EGFRvIII cells treated with or without TEFT, **(J)** Quantification of IDO1 and CPT1A mean fluorescence intensity in isolated mitochondrial fractions from T98G-EGFRvIII cells (n = 3 biological replicates per group), **(K)** Representative gating strategy for flow cytometric analysis of isolated mitochondrial particles. ATP5A was used as a mitochondrial marker and Calnexin as an endoplasmic reticulum contamination control, **(L)** Subcellular fractionation followed by immunoblotting showing TEFT-induced enrichment of IDO1 and CPT1A in mitochondria-enriched fractions of TBD0220, LN229-EGFRvIII, T98G-EGFRvIII, and patient-derived LG-260 cells. Calnexin were used as non-mitochondrial fraction controls. Data are presented as mean ± SD. The exact n values and definitions of biological replicates are specified for the relevant panels. Statistical tests were as follows: two-tailed unpaired Student's t-test for panel **F** and **J**; ordinary one-way ANOVA followed by two-sided Šídák-adjusted multiple-comparisons tests for panels **B** and **H**. Exact P values, adjusted for multiple comparisons where applicable, are shown in the corresponding panels. Source data are provided as a Source Data file. Abbreviations: A.U., arbitrary units.

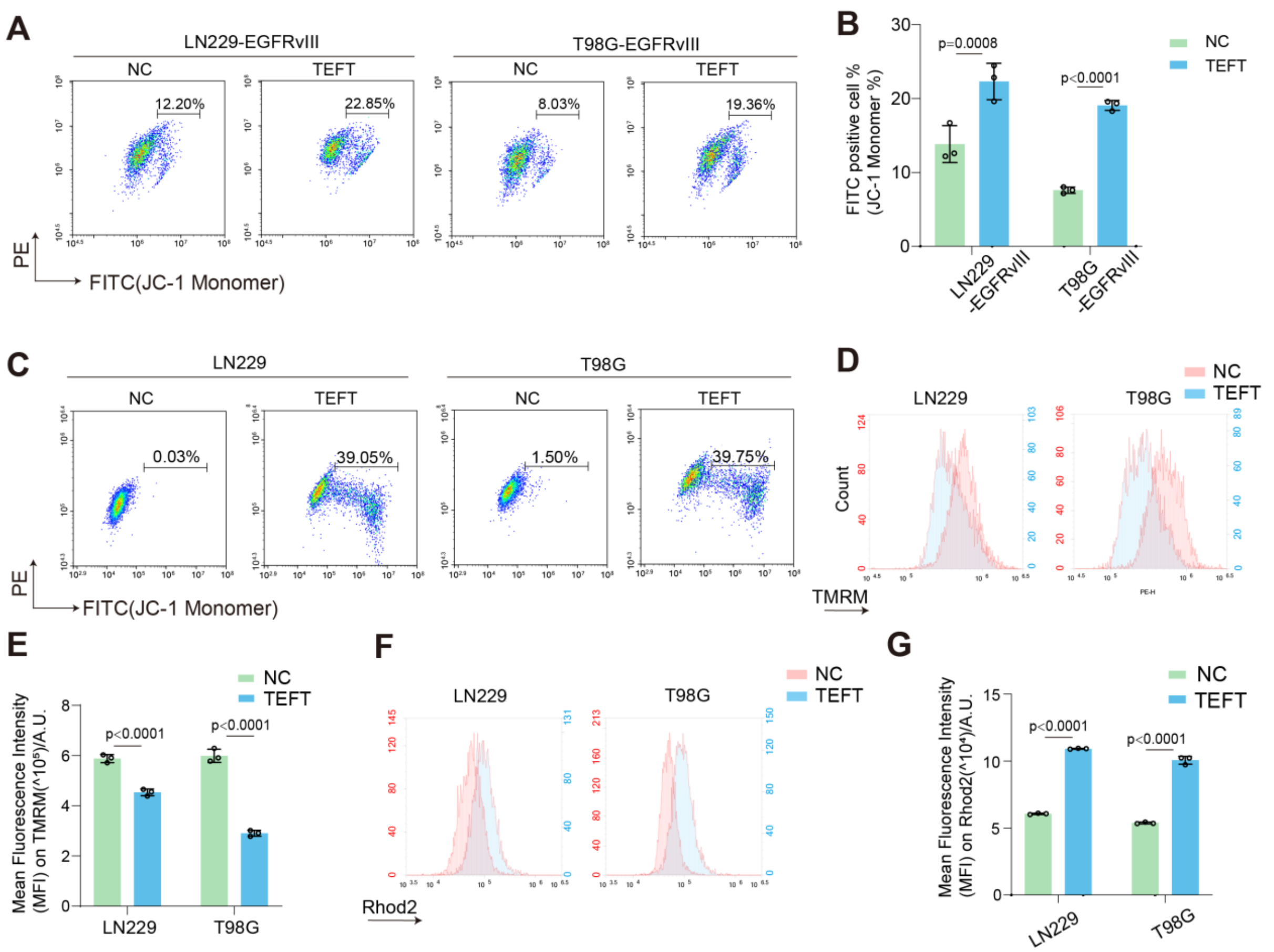

fig. S5. TEFT induces mitochondrial depolarization and calcium accumulation in GBM cells.

(A) Representative flow cytometry dot plots of JC-1 staining in LN229-EGFRvIII and

T98G-EGFRvIII cells treated with or without TEFT. Increased FITC-positive JC-1 monomer signal indicates mitochondrial depolarization, **(B)** Quantification of JC-1 monomer-positive cells in LN229-EGFRvIII and T98G-EGFRvIII cells (n = 3 biological replicates per group), **(C)** Representative flow cytometry dot plots of JC-1 staining in parental LN229 and T98G cells treated with or without TEFT, **(D)** Representative flow cytometry histograms of mitochondrial membrane potential, assessed by TMRM staining, in parental LN229 and T98G cells treated with or without TEFT, **(E)** Quantification of TMRM mean fluorescence intensity in parental LN229 and T98G cells (n = 3 biological replicates per group), **(F)** Representative flow cytometry histograms of Rhod-2 staining in parental LN229 and T98G cells treated with or without TEFT, **(G)** Quantification of Rhod-2 mean fluorescence intensity in parental LN229 and T98G cells (n = 3 biological replicates per group). Data are presented as mean ± SD. The exact n values and definitions of biological replicates are specified for the relevant panels. Statistical tests were as follows: Ordinary one-way ANOVA followed by two-sided Šídák-adjusted multiple-comparisons tests for panels **B**, **E** and **G**. Exact P values, adjusted for multiple comparisons where applicable, are shown in the corresponding panels. Source data are provided as a Source Data file. Abbreviations: A.U., arbitrary units.

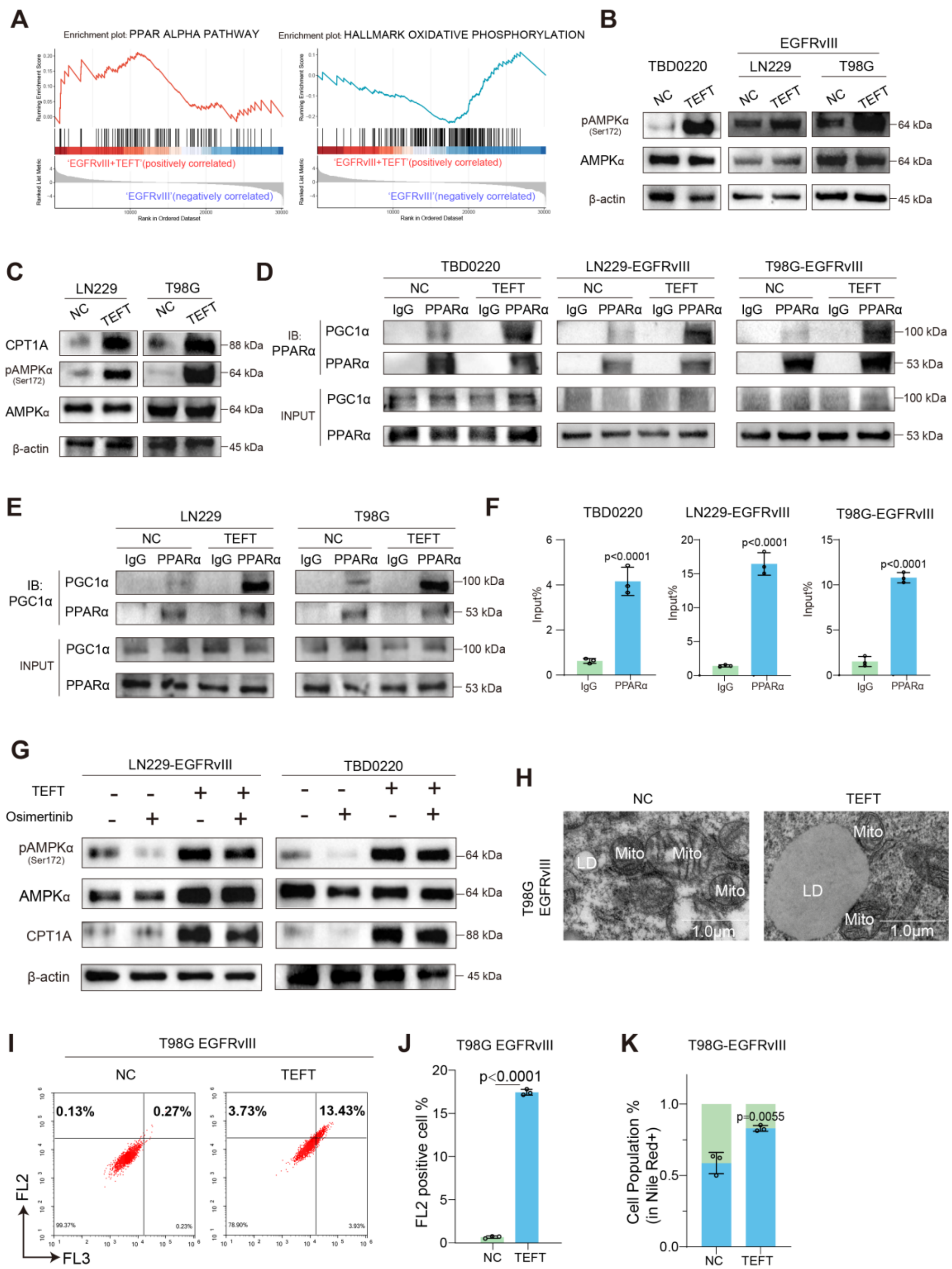

fig. S6. TEFT activates the AMPK–PPARα–CPT1A metabolic-stress response. (A) GSEA enrichment plots showing positive enrichment of the PPARα pathway and negative enrichment of oxidative phosphorylation signatures in EGFRvIII cells treated with TEFT, **(B)** Western blot analysis of phosphorylated AMPKα (p-AMPKα, Ser172) and total AMPKα in TBD0220, LN229-EGFRvIII, and T98G-EGFRvIII cells treated with or without TEFT, **(C)** Western blot analysis of CPT1A, phosphorylated AMPKα, and total

AMPKα in parental LN229 and T98G cells treated with or without TEFT, **(D)** Co-immunoprecipitation analysis of the interaction between PPARα and its transcriptional co-activator PGC1α in TBD0220, LN229-EGFRvIII, and T98G-EGFRvIII cells treated with or without TEFT, **(E)** Co-immunoprecipitation analysis of PPARα–PGC1α interaction in parental LN229 and T98G cells treated with or without TEFT, **(F)** ChIP-qPCR analysis of PPARα occupancy at predicted PPRE motifs within the CPT1A promoter region in TBD0220, LN229-EGFRvIII, and T98G-EGFRvIII cells. Data are presented as percentage of input (n = 3 biological replicates per group), **(G)** Western blot analysis of the AMPK-CPT1A axis in LN229-EGFRvIII and TBD0220 cells treated with osimertinib, TEFT, or the combination. Osimertinib did not abolish TEFT-induced AMPKα phosphorylation or CPT1A upregulation, **(H)** Representative transmission electron microscopy images showing TEFT-induced structural remodeling and increased physical proximity between mitochondria (Mito) and lipid droplets (LD) in T98G-EGFRvIII cells. Scale bars, 1.0 μm, **(I)** Representative flow cytometry dot plots of Nile Red staining showing neutral lipid accumulation in T98G-EGFRvIII cells treated with or without TEFT, **(J)** Quantification of the percentage of FL2-positive cells after Nile Red staining in T98G-EGFRvIII cells (n = 3 biological replicates per group), **(K)** Analysis of Nile Red-defined lipid droplet subpopulations in T98G-EGFRvIII cells treated with or without TEFT (n = 3 biological replicates per group). Data are presented as mean ± SD. The exact n values and definitions of biological replicates are specified for the relevant panels. Statistical tests were as follows: two-tailed unpaired Student's t-test for panel **F**, **J** and **K**. Exact P values, adjusted for multiple comparisons where applicable, are shown in the corresponding panels. Source data are provided as a Source Data file. Abbreviations: Mito, mitochondria; LD, lipid droplet.

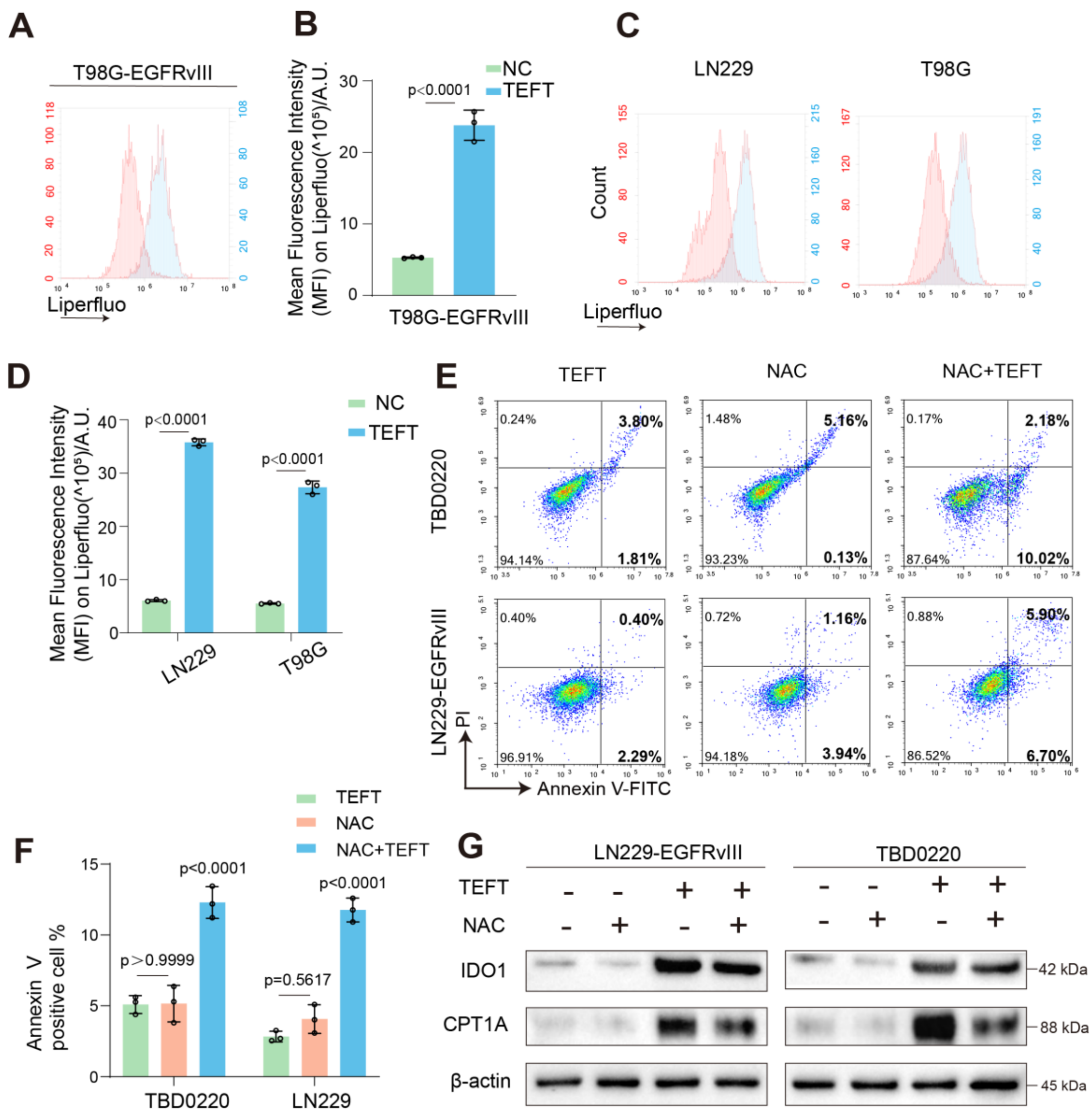

fig. S7. TEFT-induced lipid oxidative stress and the effects of NAC on TEFT sensitivity. (A) Representative flow cytometry histogram of lipid peroxidation assessed by Liperfluo staining in T98G-EGFRvIII cells treated with or without TEFT, **(B)** Quantification of Liperfluo mean fluorescence intensity in T98G-EGFRvIII cells (n = 3 biological replicates per group), **(C)** Representative flow cytometry histograms of lipid oxidative stress assessed by Liperfluo staining in parental LN229 and T98G cells treated with or without TEFT, **(D)** Quantification of Liperfluo mean fluorescence intensity in parental LN229 and T98G cells (n = 3 biological replicates per group), **(E)** Representative Annexin V/PI flow cytometry plots of TBD0220 and LN229-EGFRvIII cells treated with TEFT, NAC, or TEFT plus NAC, **(F)** Quantification of Annexin V-positive cells from panel E. NAC co-treatment increased apoptosis under TEFT exposure, indicating partial restoration of TEFT sensitivity (n = 3 biological replicates per group), **(G)** Western blot analysis of IDO1 and CPT1A protein levels in LN229-EGFRvIII and TBD0220 cells treated with TEFT, NAC, or TEFT plus NAC. Data are presented as mean ± SD. The exact n values and definitions of biological replicates are specified for the relevant panels. Statistical tests were as follows:

two-tailed unpaired Student's t-test for panel **B**; ordinary one-way ANOVA followed by two-sided Šídák-adjusted multiple-comparisons tests for panels **D** and **F**. Exact P values, adjusted for multiple comparisons where applicable, are shown in the corresponding panels. Source data are provided as a Source Data file. Abbreviations: A.U., arbitrary units.

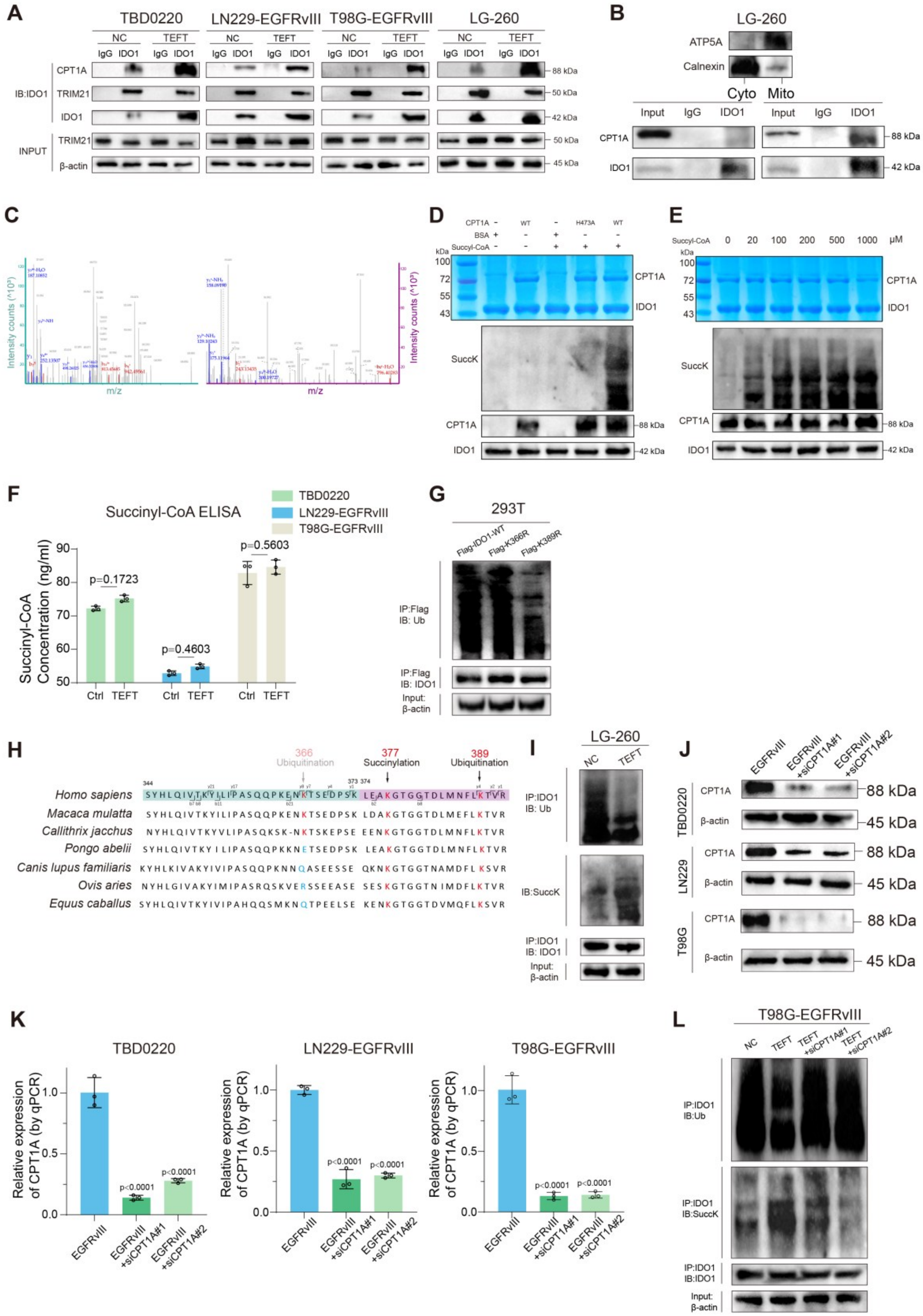

fig. S8. CPT1A regulates the succinylation-ubiquitination balance of IDO1. (A) IDO1 co-immunoprecipitation analysis in TBD0220, LN229-EGFRvIII, T98G-EGFRvIII, and

patient-derived wLG-260 cells treated with or without TEFT, **(B)** Subcellular fractionation followed by IDO1 co-immunoprecipitation in patient-derived LG-260 cells. The CPT1A-IDO1 complex was enriched in the mitochondrial fraction. ATP5A and Calnexin were used to assess mitochondrial and non-mitochondrial fraction quality, respectively, **(C)** Representative LC-MS/MS spectra identifying post-translational modification sites on IDO1, **(D)** In vitro recombinant protein succinylation assay using purified IDO1 and CPT1A proteins. Wild-type CPT1A promoted IDO1 lysine succinylation in the presence of succinyl-CoA, whereas the catalytically defective H473A mutant showed reduced activity, **(E)** Dose-dependent in vitro succinylation assay showing increased IDO1 lysine succinylation with increasing succinyl-CoA concentrations in the presence of recombinant CPT1A, **(F)** Quantification of total cellular succinyl-CoA levels by ELISA in TBD0220, LN229-EGFRvIII, and T98G-EGFRvIII cells treated with or without TEFT ($n = 3$ biological replicates per group), **(G)** Immunoprecipitation analysis of IDO1 ubiquitination in 293T cells expressing Flag-tagged IDO1-WT, IDO1-K366R, or IDO1-K389R. Mutation of K389 reduced IDO1 ubiquitination compared with wild-type IDO1, **(H)** Multiple sequence alignment of IDO1 across species showing conservation of the identified ubiquitination sites K366 and K389 and the succinylation site K377, **(I)** Immunoprecipitation analysis of endogenous IDO1 ubiquitination and succinylation in patient-derived EGFRvIII cells treated with or without TEFT, **(J)** Western blot validation of CPT1A knockdown efficiency in TBD0220, LN229-EGFRvIII, and T98G-EGFRvIII cells transfected with two independent siRNAs targeting CPT1A. β-actin served as a loading control, **(K)** RT-qPCR validation of CPT1A knockdown efficiency in cells transfected with two independent siRNAs targeting *CPT1A* ($n = 3$ biological replicates per group), **(L)** Immunoprecipitation analysis of endogenous IDO1 ubiquitination and succinylation in T98G-EGFRvIII cells. Data are presented as mean ± SD. The exact n values and definitions of biological replicates are specified for the relevant panels. Statistical tests were as follows: two-tailed unpaired Student's t-test for panel **F**; ordinary one-way ANOVA followed by two-sided Šídák-adjusted multiple-comparisons tests for panel **K**. Exact P values, adjusted for multiple comparisons where applicable, are shown in the corresponding panels. Source data are provided as a Source Data file.

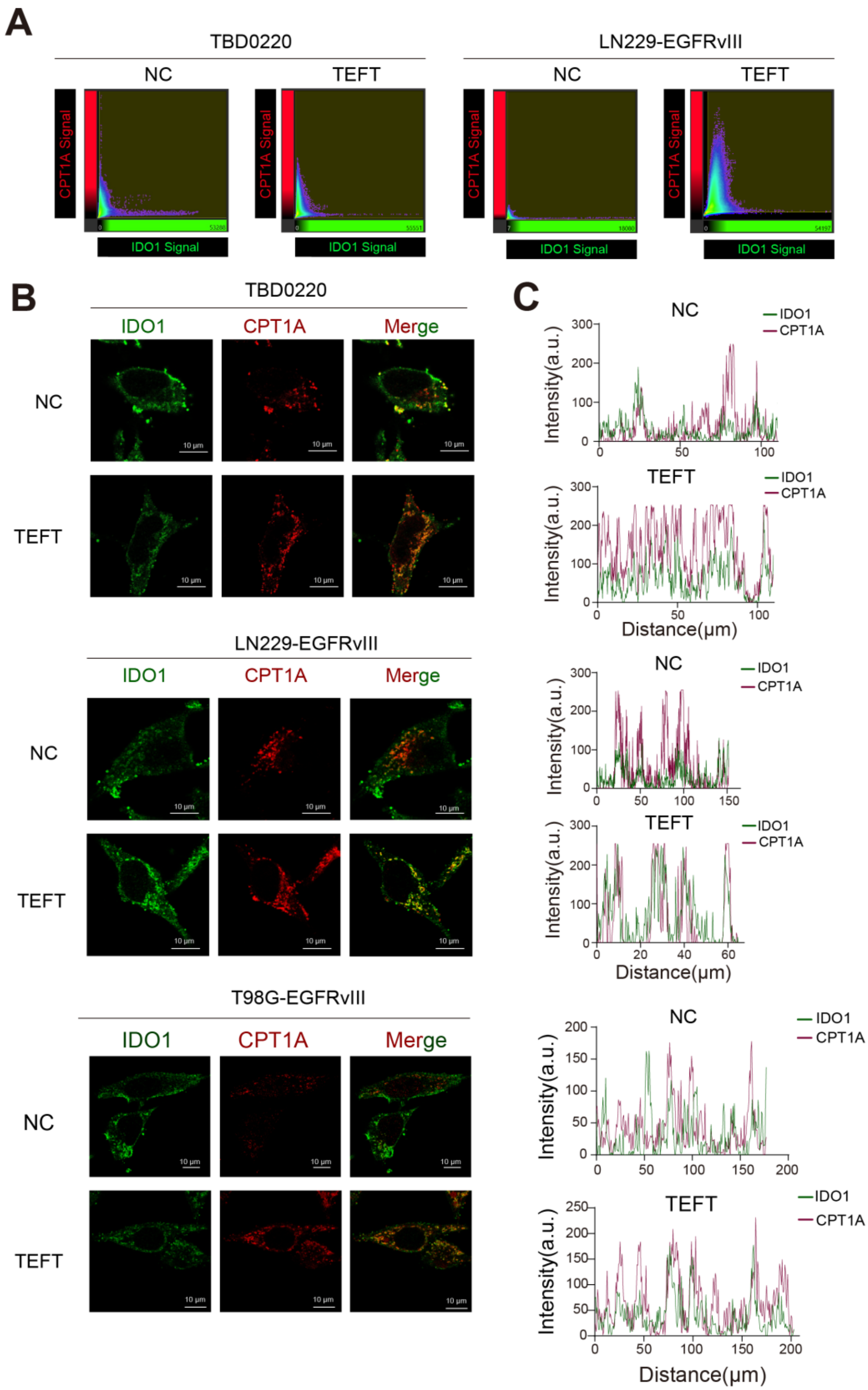

A
TBD0220
LN229-EGFRvIII
NC
TEFT
CPT1A Signal
IDO1 Signal
B
TBD0220
IDO1
CPT1A
Merge
NC
TEFT
LN229-EGFRvIII
T98G-EGFRvIII
C
IDO1
CPT1A
Intensity(a.u.)
Distance(μm)

fig. S9. TEFT increases the spatial association of IDO1 with CPT1A-positive mitochondrial structures. **(A)** Representative high-resolution Z-stack confocal colocalization analysis corresponding to Fig. 4I. The two-dimensional fluorescence intensity distribution of IDO1 and CPT1A signals in TBD0220 and LN229-EGFRvIII cells shows increased signal overlap after TEFT treatment, supporting TEFT-induced recruitment of IDO1 to CPT1A-positive mitochondrial structures, **(B)** Representative confocal immunofluorescence images showing IDO1 and CPT1A staining in TBD0220, LN229-EGFRvIII, and T98G-EGFRvIII cells treated with or without TEFT. TEFT increased the spatial overlap between IDO1 and CPT1A-positive mitochondrial structures. IDO1, green; CPT1A, red. Scale bars, 10 µm, **(C)** Line-scan fluorescence intensity profiles of IDO1 and CPT1A signals along the indicated regions in panel B. TEFT increased the synchronization of IDO1 and CPT1A fluorescence peaks, supporting enhanced spatial proximity between the two proteins. Source data are provided as a Source Data file. Abbreviations: a.u., arbitrary units.

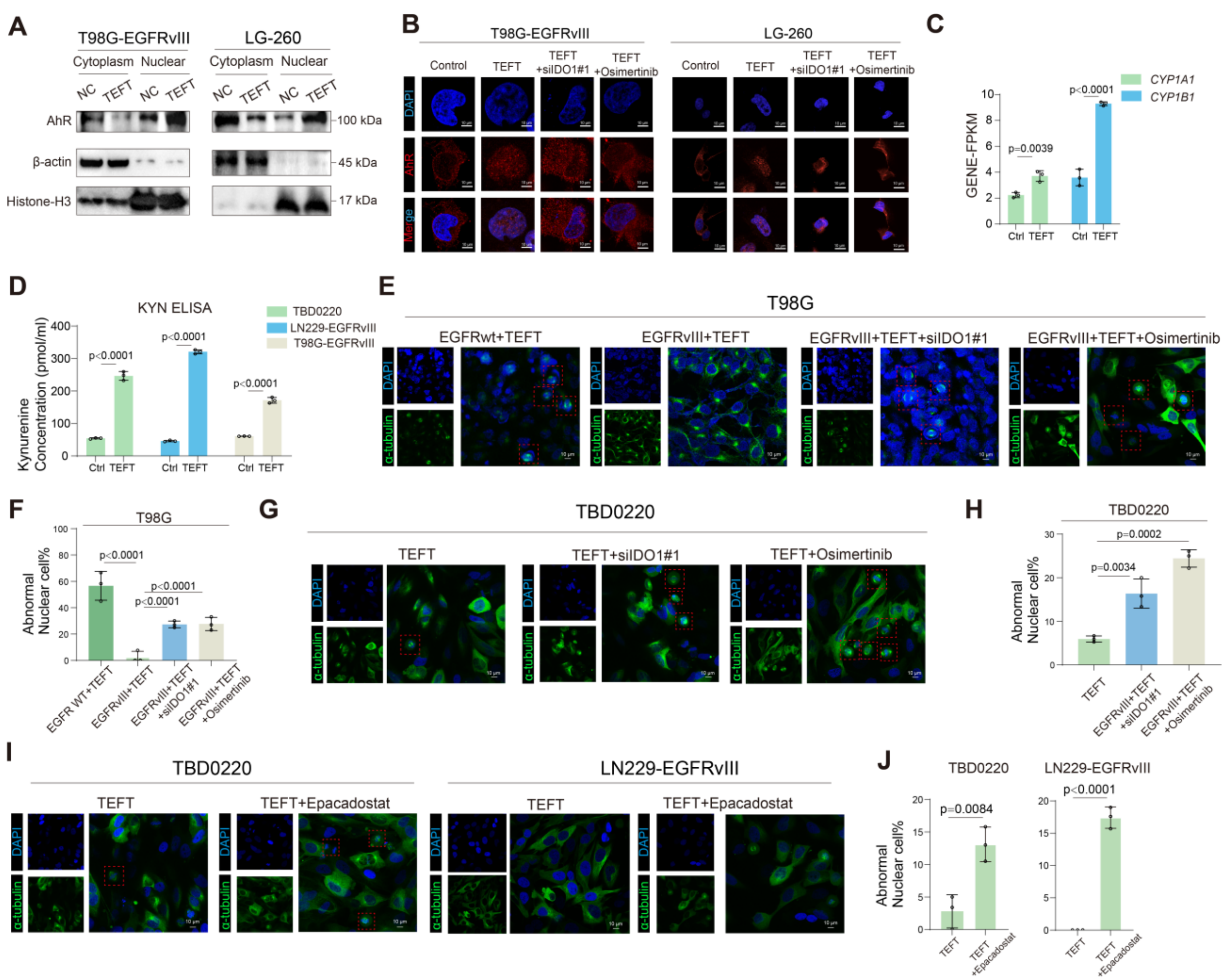

fig. S10. IDO1-dependent AhR activation supports microtubule organization under TEFT. **(A)** Western blot analysis of AhR in cytoplasmic and nuclear fractions of T98G-EGFRvIII and patient-derived LG-260 cells treated with or without TEFT, (**B**) Representative immunofluorescence images of AhR in T98G-EGFRvIII and LG-260 cells under the indicated conditions. DAPI, nuclei; AhR, red. Scale bars, 10 µm, (**C**)

Gene expression levels of canonical AhR target genes *CYP1A1* and *CYP1B1* in control and TEFT-treated EGFRvIII-LN229 cells, shown as FPKM values (n = 3 biological replicates per group), (**D**) Kynurenine ELISA showing increased extracellular kynurenine levels in the culture supernatants of TBD0220, LN229-EGFRvIII, and T98G-EGFRvIII cells after TEFT treatment (n = 3 biological replicates per group), (**E**) Representative immunofluorescence images of α-tubulin staining in T98G cells under the indicated conditions. TEFT induced spindle and nuclear abnormalities in EGFR wild-type cells, whereas EGFRvIII cells maintained a more preserved microtubule architecture under TEFT. This protective phenotype was disrupted by IDO1 knockdown or osimertinib treatment. DAPI, nuclei; α-tubulin, green. Scale bars, 10 μm, (**F**) Quantification of cells with abnormal nuclear morphology in T98G cells under the indicated conditions (n = 3 biological replicates per group), (**G and H**) Representative immunofluorescence images (G) and quantification (H) of α-tubulin staining in TBD0220 cells treated with TEFT, TEFT plus IDO1 knockdown, or TEFT plus osimertinib. DAPI, nuclei; α-tubulin, green. Scale bars, 10 μm (n = 3 biological replicates per group), (**I and J**) Representative immunofluorescence images (I) and quantification (J) of α-tubulin staining in TBD0220 and LN229-EGFRvIII cells treated with TEFT alone or TEFT plus epacadostat. DAPI, nuclei; α-tubulin, green. Scale bars, 10 μm (n = 3 biological replicates per group). Data are presented as mean ± SD. The exact n values and definitions of biological replicates are specified for the relevant panels. Statistical tests were as follows: two-tailed unpaired Student's t-test for panels **C**, **D** and **J**; ordinary one-way ANOVA followed by two-sided Šídák-adjusted multiple-comparisons tests for panels **F** and **H**. Exact P values, adjusted for multiple comparisons where applicable, are shown in the corresponding panels. Source data are provided as a Source Data file.

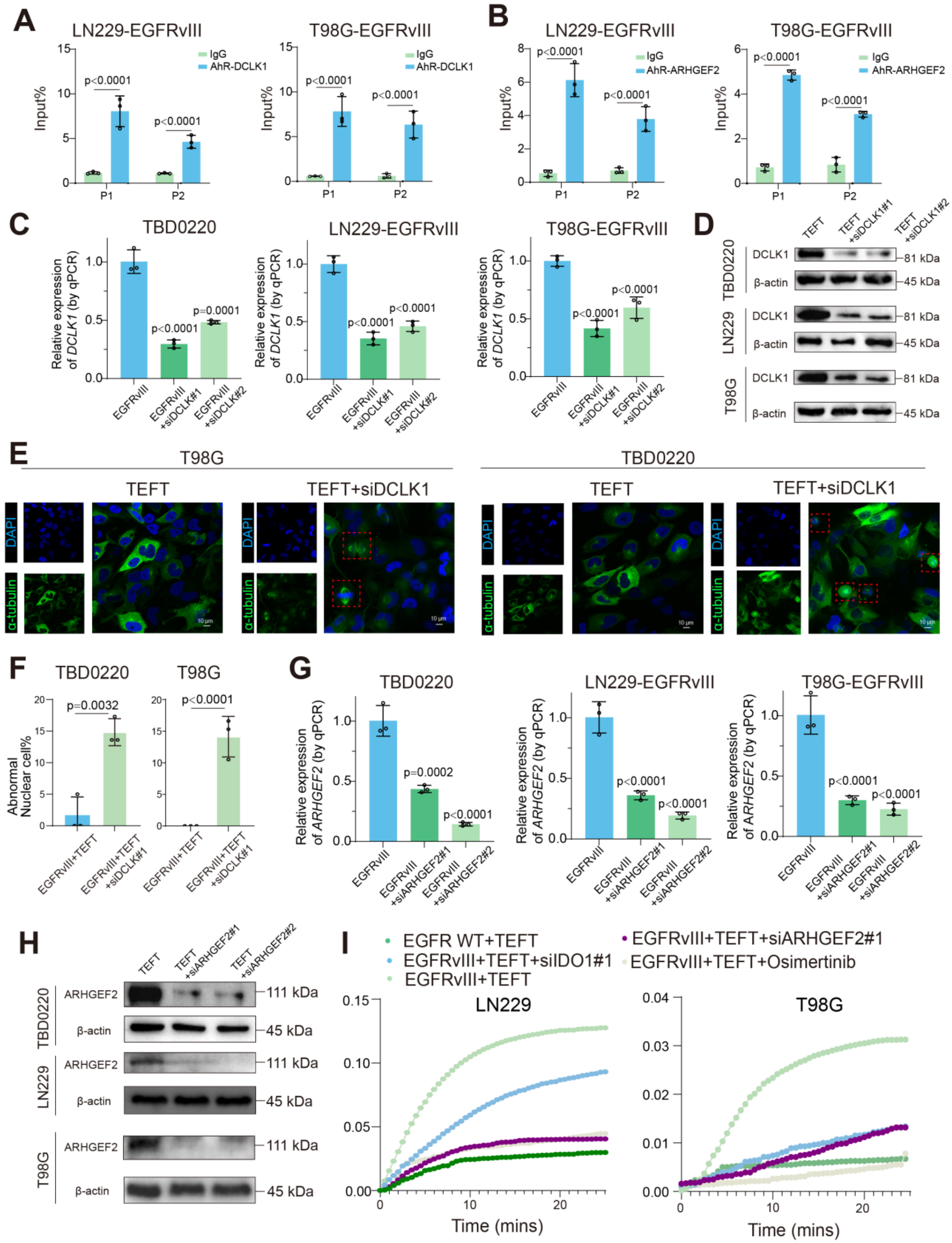

fig. S11. AhR-regulated DCLK1 and ARHGEF2 support microtubule organization under TEFT. (A) ChIP-qPCR analysis of AhR occupancy at the predicted *DCLK1* promoter

regions (P1 and P2) in LN229 and T98G EGFRvIII cells (n = 3 biological replicates per group), **(B)** ChIP-qPCR analysis of AhR occupancy at the *ARHGEF2* promoter regions (P1 and P2) (n = 3 biological replicates per group), **(C)** RT-qPCR validation of *DCLK1* knockdown efficiency in TBD0220, LN229-EGFRvIII, and T98G-EGFRvIII cells transfected with two independent siRNAs targeting DCLK1 (n = 3 biological replicates per group), **(D)** Western blot validation of *DCLK1* knockdown efficiency in TBD0220, LN229-EGFRvIII, and T98G-EGFRvIII cells, **(E)** Representative immunofluorescence images of α-tubulin staining in T98G-EGFRvIII and TBD0220 cells treated with TEFT with or without DCLK1 knockdown. DCLK1 depletion increased TEFT-induced spindle and nuclear abnormalities. DAPI, nuclei; α-tubulin, green. Scale bars, 10 µm, **(F)** Quantification of cells with abnormal nuclear morphology in TBD0220 and T98G-EGFRvIII cells treated with TEFT with or without DCLK1 knockdown (n = 3 biological replicates per group), **(G)** RT-qPCR validation of *ARHGEF2* knockdown efficiency in TBD0220, LN229-EGFRvIII, and T98G-EGFRvIII cells transfected with two independent siRNAs targeting ARHGEF2 (n = 3 biological replicates per group), **(H)** Western blot validation of ARHGEF2 knockdown efficiency in TBD0220, LN229-EGFRvIII, and T98G-EGFRvIII cell, **(I)** In vitro tubulin polymerization assay in LN229 and T98G cells under the indicated conditions. IDO1 knockdown, ARHGEF2 knockdown, or osimertinib reduced the microtubule-polymerizing activity associated with EGFRvIII-mediated TEFT resistance. Data are presented as mean ± SD. The exact n values and definitions of biological replicates are specified for the relevant panels. Statistical tests were as follows: two-tailed unpaired Student's t-test for panels **A**, **B** and **F**; ordinary one-way ANOVA followed by two-sided Šídák-adjusted multiple-comparisons tests for panels **C** and **G**. Exact P values, adjusted for multiple comparisons where applicable, are shown in the corresponding panels. Source data are provided as a Source Data file.

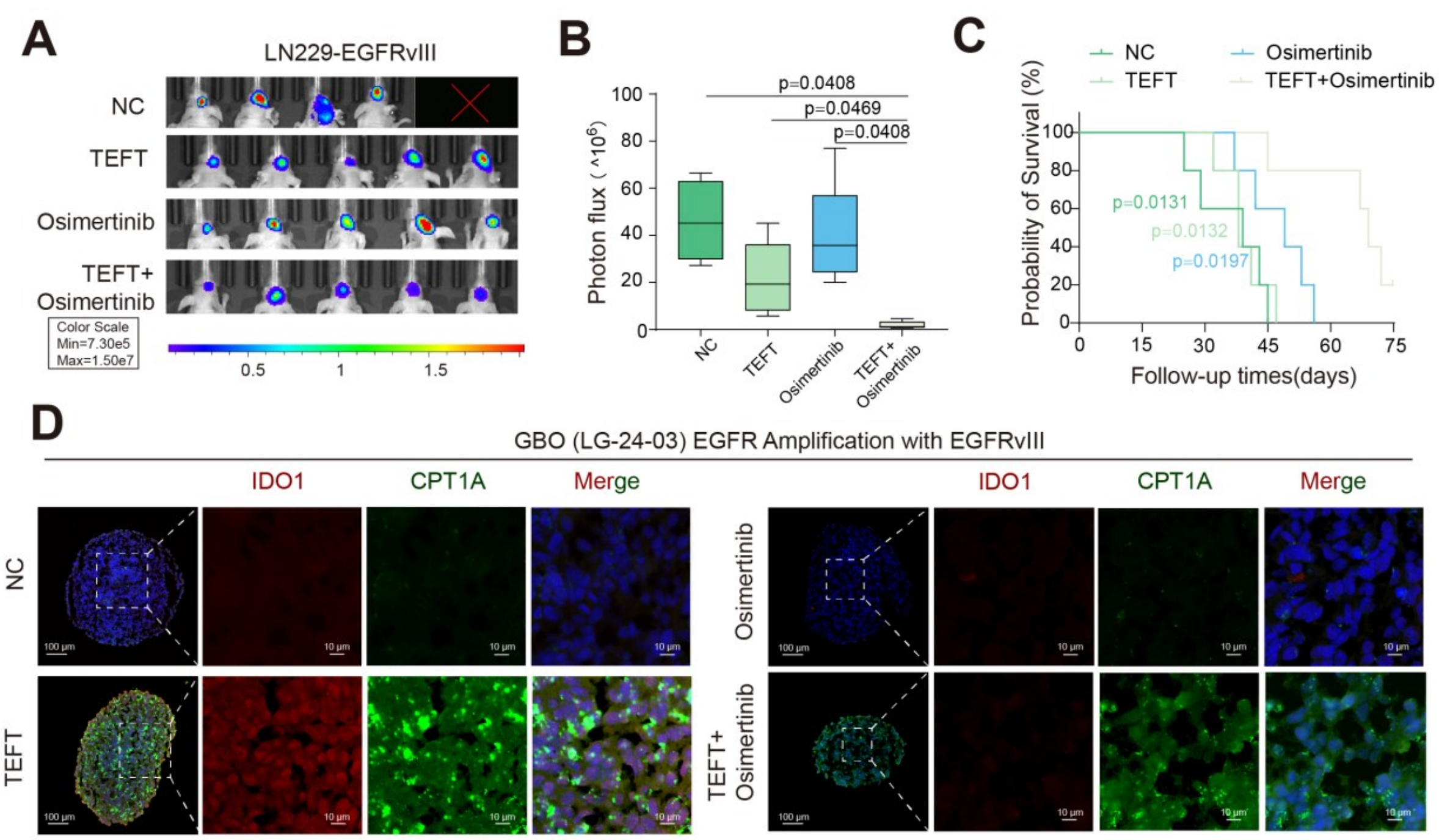

fig. S12. Osimertinib enhances the response of EGFRvIII-driven GBM models to TEFT in vivo and ex vivo. (A) Representative bioluminescence images of mice bearing orthotopic LN229-EGFRvIII xenografts from different treatment groups (NC, TEFT, Osimertinib, Combination), **(B)** Quantification of tumor photon flux for the LN229-EGFRvIII model, The NC group included n = 4 mice, whereas the TEFT, osimertinib, and TEFT plus osimertinib groups each included n = 5 mice. One in the NC group reached the predefined humane endpoint before the final imaging assessment. Data are presented as box-and-whisker plots. The centre line indicates the median, the lower and upper bounds of the box indicate the 25th and 75th percentiles, respectively, and the whiskers extend from the minimum to the maximum value. **(C)** Kaplan-Meier survival curves for mice in the NC, TEFT, osimertinib, and TEFT plus osimertinib groups. Three prespecified comparisons of the TEFT plus osimertinib group with the NC, TEFT, and osimertinib groups were performed using two-sided log-rank (Mantel-Cox) tests, with Holm-Šídák adjustment for multiple comparisons. The multiplicity-adjusted P values are shown, **(D)** Representative immunofluorescence images of IDO1 (red) and CPT1A (green) in patient-derived glioblastoma organoids (GBO, LG-24-03) treated *ex vivo*. Data are presented as mean ± SD. The exact n values and definitions of biological replicates are specified for the relevant panels. Statistical tests of panel **B** were as follows: overall group differences were assessed using Brown-Forsythe and Welch one-way ANOVA. Three prespecified comparisons of the TEFT plus osimertinib group with the NC, TEFT, and osimertinib groups were subsequently performed using two-sided unpaired Welch's t-tests, with Holm adjustment for the three comparisons. Exact P values, adjusted for multiple comparisons where applicable, are shown in the corresponding panels. Source data are provided as a Source Data file.

Supplementary Note: Detailed Clinical Case Histories

Below, we provide detailed clinical vignettes for the four glioblastoma patients included in the exploratory compassionate-use case series. Given the refractory nature of the disease and lack of alternative effective options, Case#3 and Case#4 received combination therapy on a compassionate use basis. Written informed consent was obtained from all four patients for publication of the potentially identifiable information presented here, including clinical histories, imaging findings and indirect identifiers such as sex and molecular diagnosis (ethics approval no. KYLL-2025B-107). These summaries document the disease course, molecular pathology, and radiological responses to the combination regimen of Tumor Electric Field Therapy (TEFT) and osimertinib, extending the data presented in Fig. 6.

Case #1. Female. The patient was admitted with a 4-day history of dizziness and nausea and a 2-day history of bilateral temporal pain. After admission, relevant laboratory and imaging examinations were completed. The pre-operative diagnosis was **temporal-lobe glioblastoma.** On the third hospital day she underwent temporal-lobe tumor resection. Post-operative histopathology and molecular studies established the integrated diagnosis of **glioblastoma, *IDH*-wildtype, WHO grade IV, Ki-67 index 30 %**. Postoperative MRI indicates a total excision. Molecular findings: ***EGFR* negative, *MGMT* promoter methylation positive, 1p/19q co-deletion negative**. Post-operatively the patient received the standard STUPP protocol (radiotherapy plus concomitant and adjuvant temozolomide) together with TEFT. PFS lasted **16 months**. Surveillance imaging then revealed a local recurrence; the patient underwent a second resection at

another hospital. **21 months** have now elapsed since the initial diagnosis. The patient remains under active follow-up.

Case #2. Male. The patient was admitted with a 4-day history of paroxysmal numbness of the left limbs. After admission, relevant laboratory and imaging examinations were completed. The pre-operative diagnosis was **temporal-lobe glioblastoma**. On the third hospital day he underwent temporal-lobe tumor resection. Post-operative histopathology and molecular studies established the integrated diagnosis of **glioblastoma, *IDH*-wildtype, WHO grade IV, Ki-67 index 20 %**. Postoperative MRI indicates a total excision. Molecular findings: ***EGFR* amplification positive, *EGFR*vIII positive, *MGMT* promoter methylation negative, 1p/19q co-deletion negative**. Post-operatively the patient received the standard STUPP protocol (radiotherapy plus concomitant and adjuvant temozolomide) together with TEFT. PFS lasted only **5 months**. Surveillance imaging then revealed a local recurrence that progressed rapidly. No second resection was performed. OS was **12 months**.

Case #3. Male. The patient was admitted with a 5-day history of headache and dizziness. After admission, relevant laboratory and imaging examinations were completed. The pre-operative diagnosis was **occipital-lobe glioblastoma**. On the third hospital day he underwent occipital-lobe tumor resection. Post-operative histopathology and molecular studies established the integrated diagnosis of **glioblastoma, *IDH*-wildtype, WHO grade IV, Ki-67 index 15 %**. Postoperative MRI indicates a total excision. Molecular findings: ***EGFR* amplification positive, *EGFR* p.E709K mutation, *EGFR*vIII negative, *MGMT* promoter methylation negative, 1p/19q co-deletion negative**. Post-operatively the patient received the standard STUPP protocol (radiotherapy plus concomitant and adjuvant temozolomide) together with TEFT. PFS lasted **11 months**. Surveillance imaging then revealed multiple recurrent lesions, midline shift, and subfalcine herniation; ECOG performance status was **4**. Independently assessed by two clinicians, the patient was not eligible for second surgery. At the family's fully informed request, the patient was enrolled in an experimental regimen of **TEFT plus osimertinib 80 mg po qd**; no additional interventions were administered. As of the current data-cutoff, no further progression has been observed during the **3-month follow-up period**, midline structures have re-aligned, and ECOG has improved to **3**. The patient remains under active follow-up.

Case #4. Male. The patient was admitted with a 3-day history of headache and dizziness. After admission, relevant laboratory and imaging examinations were completed. The pre-operative diagnosis was **occipital-lobe glioblastoma**. On the third hospital day he underwent occipital-lobe tumor resection. Post-operative histopathology and molecular studies established the integrated diagnosis of **glioblastoma, IDH-wildtype, WHO grade IV, Ki-67 index 20 %.** Postoperative MRI indicates a total excision. Molecular findings: ***EGFR* amplification positive, *EGFR*vIII positive, *MGMT* promoter methylation negative, 1p/19q co-deletion negative.** Post-operatively the patient received the standard STUPP protocol (radiotherapy plus concomitant and adjuvant temozolomide) together with TEFT. First progression-free survival (PFS-1) lasted **7 months**; surveillance imaging revealed a local recurrence, and a second resection was performed. Unfortunately, the second PFS (PFS-2) lasted only **3 months**; imaging again showed in-situ recurrence; ECOG performance status was **4**. Independently

assessed by the treating clinician, the patient was not eligible for further surgery. At the family's fully informed request, the patient was enrolled in an experimental regimen of **TEFT plus osimertinib 80 mg po qd**; no additional interventions were administered. As of the current data-cutoff, no further progression has been observed during the 3-month follow-up period, and ECOG has improved to 3. The patient remains under active follow-up.

Supplementary Table 1. Antibodies used for experiments

Antibody	Source	Identifier
EGF Receptor (D38B1) Rabbit Monoclonal Antibody	Cell Signaling Technology	Cat#4267
Phospho-EGF Receptor (Tyr1068) (D7A5) Rabbit Monoclonal Antibody	Cell Signaling Technology	Cat#3777
Ki-67 (8D5) Mouse Monoclonal Antibody	Cell Signaling Technology	Cat#9449
NF-κB p65 Recombinant monoclonal antibody	Proteintech	Cat#80979-1-RR
Phospho-NF-κB p65 (Ser536) Recombinant monoclonal antibody	Proteintech	Cat#80379-2-RR
Anti-Indoleamine 2, 3-dioxygenase antibody	Abcam	Cat#EPR20374
Beta Actin Monoclonal antibody	Proteintech	Cat#66009-1-Ig
ubiquitin Polyclonal Antibody	Proteintech	Cat#10201-2-AP
Anti-CPT1A antibody [8F6AE9]	Abcam	Cat#ab128568
TRIM21 Recombinant monoclonal antibody	Proteintech	Cat#83530-3-RR
Anti-Succinyllysine Mouse mAb	PTMBio	Cat#PTM-419

AhR (D5S6H) Rabbit Monoclonal Antibody	Cell Signaling Technology	Cat#83200
Histone H3 Polyclonal antibody	Proteintech	Cat#17168-1-AP
Alpha Tubulin Recombinant monoclonal antibody	Proteintech	Cat#80762-1-RR
PPARA Monoclonal antibody	Proteintech	Cat#66826-1-Ig
PGC-1 alpha (3G6) Rabbit Monoclonal Antibody	Cell Signaling Technology	Cat#2178
AMPK Alpha Polyclonal antibody	Proteintech	Cat#10929-2-AP
Phospho-AMPK Alpha (Thr172) Recombinant monoclonal antibody	Proteintech	Cat#80209-6-RR
Na,K-ATPase alpha1 (D4Y7E) Rabbit Monoclonal Antibody	Cell Signaling Technology	Cat#23565
Calnexin Antibody	Cell Signaling Technology	Cat#2433
DCLK1 Polyclonal antibody	Proteintech	Cat#21699-1-AP
GEF-H1 Polyclonal antibody	Proteintech	Cat#24472-1-AP
Rabbit IgG control Polyclonal antibody	Proteintech	Cat#30000-0-AP
ATP5A1 Polyclonal antibody	Proteintech	Cat#14676-1-AP

416 **Supplementary Table 2. Reagents used for experiments**

Reagent	Source	Identifier

BD Pharmingen™ PI/RNase Staining Buffer	BD Biosciences	Cat#550825
JSH-23	MCE	Cat#HY-13982
Osimertinib	MCE	Cat#HY-15772
Cycloheximide	MCE	Cat#HY-12320
Chloroquine	MCE	Cat#HY-17589A
MG-132	MCE	Cat#HY-13259
Tetramethylrhodamine, Methyl Ester, Perchlorate	Thermo Fisher Scientific	Cat#T668
Nile Red	MCE	Cat# HY-D0718
Recombinant human CPT1A protein	Proteintech	Cat#Ag7202
Recombinant Human IDO-1 Protein (RP00023)	abclonal	Cat#RP00023
Coomassie Blue Fast Staining Solution	Beyotime	Cat#P0017
D-Luciferin potassium	MCE	Cat# HY-12591B
Epacadostat	MCE	Cat# HY-15689

417 **Supplementary Table 3. Kits used for experiments**

Kit	Source	Identifier
Annexin V-PE Apoptosis Detection Kit	Beyotime	Cat#C1065S
One Step TUNEL Apoptosis Assay Kit	Beyotime	Cat#C1086
One-Day Chromatin Immunoprecipitation Kits	Merck	Cat#17-10086
Fast Silver Stain Kit	Beyotime	Cat#P0017

BODIPY™ 581/591 C11	Thermo Fisher Scientific	Cat#D3861
Mitochondria Isolation Kit for Cultured Cells	Thermo Fisher Scientific	Cat#89874
Nuclear and Cytoplasmic Protein Extraction Kit	Beyotime	Cat#P0027
Tubulin Polymerization Assay Kit	Cytoskeleton, Inc.	Cat#BK006P
ATP Assay Kit	Beyotime	Cat#S0026
Enhanced mitochondrial membrane potential assay kit with JC-1	Beyotime	Cat#C2003
Lipid Peroxidation MDA Assay Kit	Beyotime	Cat#S0131
Mitochondrial Calcium Influx Assay Kit with Rhod-2 AM	Beyotime	Cat#S1062

Supplementary Table 4. Sequences of siRNAs used for gene knockdown

**Strand**		**Sequence (5'-3')**
si-IDO1#1	Passenger	GCUGUUGGAAAUAGCUUCUTT
	Guide	AGAAGCUAUUUCCAACAGCTT
si-IDO1#2	Passenger	GUCUCUCUAUUGGUGGAAATT
	Guide	UUUCCACCAAUAGAGAGACTT
si-CPT1A#1	Passenger	GACGUUAGAUGAAACUGAATT
	Guide	UUCAGUUUCAUCUAACGUCTT
si-CPT1A#2	Passenger	GCGUGAUGACAACGAUGUATT
	Guide	UACAUCGUUGUCAUCACGCTT
si-TRIM21#1	Passenger	GCAGCACGCUUGACAAUGATT
	Guide	UCAUUGUCAAGCGUGCUGCTG
	Passenger	GCAGGAGUUGGCUGAGAAGTT

si-TRIM21#2	Guide	CUUCUCAGCCAACUCCUGCTT
	Passenger	GUAGAAAGAUCGACUGCUATT
si-DCLK1#1	Guide	UAGCAGUCGAUCUUUCUACTT
	Passenger	GGUCGAUGUAGAUCAGCGATT
si-DCLK1#2	Guide	UCGCUGAUCUACAUCGACCTT
	Passenger	CCAAGUACCCGUUACUCAUTT
si-ARHGEF2#1	Guide	AUGAGUAACGGGUACUUGGTG
	Passenger	GCUUACCUGCGGCGAAUUATT
si-ARHGEF2#2	Guide	UAAUUCGCCGCAGGUAAGCCT

Supplementary Table 5. Sequences of primers used for qRT-PCR

Gene		Sequence (5’-3’)
Gapdh	Forward	TGTCTCCTGCGACTTCAACA
	Reverse	GGTGGTCCAGGGTTTCTTACT
IDO1	Forward	CAACCCCCAGCTATCAGACG
	Reverse	AAAGCACTGAAAGACGCTGC
CPT1A	Forward	GTCTCTTGATGCTGTGGAGAAAG
	Reverse	GTAACATCGGCCGTGTAGTAGAG
TRIM21	Forward	TCCTTCTACAACATCACTGACC
	Reverse	CAATATTCAGTGGACAGAGGGT
DCLK1	Forward	TATATCCTGCTGTGTGGTTTCC
	Reverse	GTATGGAGAAGGAAAGTCCACCT
ARHGEF2	Forward	TCGCCAACTGTACCAAGGTC
	Reverse	AAGCTGTCGGAGGGGTAGAT

Supplementary Table 6. Sequences of primers used for ChIP-qPCR

Gene	Sequence (5’-3’)

RelA*IDO1*P1	Forward	GGAAGTGGAGAGTTACTATGTTTCT
	Reverse	GGCTTCCACAGTTCTTTGAAAAT
RelA*IDO1*P2	Forward	GTAAAATGTTCTTCTCCGGCCA
	Reverse	CTGCCATATAGGAAGCCAGAAAG
PPARα*CPT1A*	Forward	GCATGTGAAGGCTGTAAGGG
	Reverse	TTGTGTGACATCCCCGACAGA
AhR*DCLK1*P1	Forward	GAGGGTTGGTCATCCGAAGAG
	Reverse	TTGATGTGACCTCACGGTCTG
AhR*DCLK1*P2	Forward	GGAGGGAGGAGGACCTGG
	Reverse	CCCTTTTCTTGTCTCACTCGC
AhR*ARHGEF2*P1	Forward	CTACACCCTCCCCGTAGGTAT
	Reverse	AGAGGAAATAAACCGTGCCCA
AhR*ARHGEF2*P	Forward	TGGAGAATCCCAACCTAGTCCC
	Reverse	TCACTCCCAGAGGTCGAAGA
