# Supplementary material for: CPT1A-mediated IDO1 succinylation shapes EGFRvIII-driven resistance to tumor electric field therapy in glioblastoma
Source: Nat Commun. 2026 Sep 23;17:10277. doi: 10.1038/s41467-026-77927-w (PMC13620138; doi:10.1038/s41467-026-77927-w)
Supplement: Supplementary file 2 [file 41467_2026_77927_MOESM2_ESM.pdf]

nature portfolio

Corresponding author(s): FangyongWang, Gang Li, Hao Xue

Last updated by author(s): Aug 5, 2026

Reporting Summary

Nature Portfolio wishes to improve the reproducibility of the work that we publish. This form provides structure for consistency and transparency in reporting. For further information on Nature Portfolio policies, see our Editorial Policies and the Editorial Policy Checklist.

Statistics

For all statistical analyses, confirm that the following items are present in the figure legend, table legend, main text, or Methods section.

n/a	Confirmed	
☐	☒	The exact sample size (*n*) for each experimental group/condition, given as a discrete number and unit of measurement
☐	☒	A statement on whether measurements were taken from distinct samples or whether the same sample was measured repeatedly
☐	☒	The statistical test(s) used AND whether they are one- or two-sided<br>*Only common tests should be described solely by name; describe more complex techniques in the Methods section.*
☒	☐	A description of all covariates tested
☐	☒	A description of any assumptions or corrections, such as tests of normality and adjustment for multiple comparisons
☐	☒	A full description of the statistical parameters including central tendency (e.g. means) or other basic estimates (e.g. regression coefficient) AND variation (e.g. standard deviation) or associated estimates of uncertainty (e.g. confidence intervals)
☐	☒	For null hypothesis testing, the test statistic (e.g. *F*, *t*, *r*) with confidence intervals, effect sizes, degrees of freedom and *P* value noted<br>*Give P values as exact values whenever suitable.*
☒	☐	For Bayesian analysis, information on the choice of priors and Markov chain Monte Carlo settings
☒	☐	For hierarchical and complex designs, identification of the appropriate level for tests and full reporting of outcomes
☐	☒	Estimates of effect sizes (e.g. Cohen's *d*, Pearson's *r*), indicating how they were calculated

Our web collection on statistics for biologists contains articles on many of the points above.

Software and code

Policy information about availability of computer code

Data collection	Microplate reader PerkinElmer EnSpire, Confocal laser scanning microscope Leica-SP8 and Olympus SpinSR10, Applied Biosystems7900 Real-Time PCR system, NovoCyte Quanteon 4020, PerkinElmer IVIS Spectrum, TECAN microplate reader.
Data analysis	Statistical analyses were performed using GraphPad Prism 9.0 and R version 4.3.1. Kaplan-Meier survival curves were compared using twosided<br>log-rank tests. Dose-response and Bliss independence analyses were performed using Microsoft Excel. Flow-cytometry data were analyzed using NovoExpress 1.6.2 and FlowJo v10. Microscopy images were quantified using Imaris 9.9.0 and Fiji v1.54p. RNA-seq, ATAC-seq, enrichment, and motif analyses were performed using the software and packages specified in the Methods.

For manuscripts utilizing custom algorithms or software that are central to the research but not yet described in published literature, software must be made available to editors and reviewers. We strongly encourage code deposition in a community repository (e.g. GitHub). See the Nature Portfolio guidelines for submitting code & software for further information.

Data

Policy information about availability of data

All manuscripts must include a data availability statement. This statement should provide the following information, where applicable:

- Accession codes, unique identifiers, or web links for publicly available datasets
- A description of any restrictions on data availability
- For clinical datasets or third party data, please ensure that the statement adheres to our policy

The RNA-seq and ATAC-seq data generated in this study have been deposited in the Genome Sequence Archive under accession code PRJCA057933 Browse - BioProject - CNCB-NGDC. The mass-spectrometry proteomics data generated in this study have been deposited in the iProX/ProteomeXchange repository under accession code IPX0015664000 iProX - integrated Proteome resources. The publicly available datasets analyzed in this study include the TCGA-GBM and TCGA-LGG datasets, the GTEx Analysis Release V8 dataset (dbGaP accession phs000424.v8.p2), and the CGGA dataset. These data were analyzed through GEPIA2 and GlioVis. Public AHR target-gene data were obtained from ChIP-Atlas 3.0 and GTRD. Source data are provided with this paper.
The de-identified patient information provided in the main manuscript and Supplementary Information is limited to sex, a brief clinical history, routine histopathological findings, the categorical molecular status of IDH, EGFR, MGMT promoter and 1p/19q co-deletion, and selected magnetic resonance imaging findings necessary to support the reported conclusions. No direct identifiers are included. Detailed patient-level molecular pathology sequencing results, including comprehensive variant-level reports beyond the reported categorical molecular markers, are available only under restricted access because they contain sensitive individual genomic information and unrestricted public disclosure is not permitted by the applicable participant consent, institutional ethical approval and patient-privacy requirements. These detailed data are therefore not included in the Source Data file or deposited in a public repository.
Access may be granted to qualified researchers affiliated with recognized research or healthcare institutions for research purposes compatible with the original consent and ethical approval. Requests should include a research proposal and, where applicable, evidence of ethics approval, and should be directed to the corresponding author at dr.ligang@sdu.edu.cn. Approved requesters must sign a data-use agreement restricting use to the approved purpose and prohibiting participant re-identification and onward distribution. Requests will be acknowledged within 10 working days, and a decision will normally be provided within 30 calendar days. Approved access will be provided through a secure data-transfer mechanism for 12 months, with extensions subject to renewed institutional approval.
NO directly identifying patient information will be shared.

Research involving human participants, their data, or biological material

Policy information about studies with human participants or human data. See also policy information about sex, gender (identity/presentation), and sexual orientation and race, ethnicity and racism.

Reporting on sex and gender	Sex was determined based on clinical medical records. The retrospective cohort (n=31) included 16 male and 15 female participants. The prospective compassionate use case series included 2 male participants. Sex-based analyses were not performed due to the limited sample size.
Reporting on race, ethnicity, or other socially relevant groupings	Race and ethnicity data were not collected for this study. All participants were recruited from a single center, Qilu Hospital of Shandong University (Shandong, China).
Population characteristics	The retrospective cohort (n=31) comprised patients with histologically confirmed glioblastoma (median age 59 years, range 28-75) who received Tumor Electric Field Therapy (TEFT) after standard chemoradiotherapy. Molecular profiling identified EGFRvIII mutations in 9 (29%) patients. The prospective compassionate use case series included 2 patients (ages 42 and 67) with recurrent, EGFR-driven glioblastoma who had exhausted standard therapeutic options.
Recruitment	For the retrospective analysis, patients treated at the Department of Neurosurgery, Qilu Hospital of Shandong University between 2021 and the data-cutoff date were consecutively enrolled if they met the inclusion criteria (histologically confirmed GBM, completed Stupp protocol, and TEFT use for at least 18 hours per day). The two compassionate-use cases involved patients with recurrent disease who had exhausted standard therapeutic options and received the combination treatment following multidisciplinary clinical assessment, institutional approval, and written informed consent.
Ethics oversight	The study was approved by the Medical Ethics Committee of Qilu Hospital of Shandong University (approval no. KYLL-2025B-107). Written informed consent was obtained from all participants or their legal guardians, as applicable.

Note that full information on the approval of the study protocol must also be provided in the manuscript.

Field-specific reporting

Please select the one below that is the best fit for your research. If you are not sure, read the appropriate sections before making your selection.

[x] Life sciences [ ] Behavioural & social sciences [ ] Ecological, evolutionary & environmental sciences

For a reference copy of the document with all sections, see nature.com/documents/nr-reporting-summary-flat.pdf

Life sciences study design

All studies must disclose on these points even when the disclosure is negative.

Sample size	No formal a priori statistical power calculation was performed for the mechanistic cell-based experiments. Sample sizes were based on independent biological replication commonly used for comparable assays and on preliminary estimates of experimental variability. Most cellbased experiments included at least three independent biological replicates, with exact sample sizes reported in the figure legends. Animal group sizes were selected based on prior experience with the orthotopic glioblastoma model and experimental feasibility. The retrospective clinical cohort included all consecutively eligible patients treated during the predefined study period, and the compassionate-use series comprised the two eligible cases available during the reporting period.
Data exclusions	One animal in the control group died at the experimental endpoint before the final bioluminescence measurement could be obtained and was therefore not included in the endpoint analysis. No other animals or data were excluded, and no exclusions were made on the basis of the observed outcome values.
Replication	Cell-based validation experiments were generally performed in at least three independent biological replicates, unless otherwise indicated. Exact biological and technical replicate numbers are reported in the corresponding figure legends and Source Data. High-throughput assays were conducted with the biological replication described in the Methods and were independently validated using orthogonal experiments.
Randomization	For animal experiments, mice were randomly assigned to treatment groups after confirmation of successful tumor establishment and, where applicable, after baseline bioluminescence imaging. Group allocation was performed using simple randomization. In vitro treatment conditions were assigned according to predefined experimental designs and were not subject to formal randomization.
Blinding	Investigators were not blinded during administration of the experimental treatments because the treatment conditions were operationally distinguishable. Where feasible, quantitative assessment of animal imaging, histological sections, and microscopy images was performed by investigators blinded to group allocation.

Reporting for specific materials, systems and methods

We require information from authors about some types of materials, experimental systems and methods used in many studies. Here, indicate whether each material, system or method listed is relevant to your study. If you are not sure if a list item applies to your research, read the appropriate section before selecting a response.

Materials & experimental systems

n/a	Involved in the study	
☐	☒	Antibodies
☐	☒	Eukaryotic cell lines
☒	☐	Palaeontology and archaeology
☐	☒	Animals and other organisms
☐	☒	Clinical data
☒	☐	Dual use research of concern
☒	☐	Plants

Methods

n/a	Involved in the study	
☒	☐	ChIP-seq
☐	☒	Flow cytometry
☐	☒	MRI-based neuroimaging

Antibodies

Antibodies used

EGF Receptor (D38B1) Rabbit Monoclonal Antibody Cell Signaling Technology Cat#4267
Phospho-EGF Receptor (Tyr1068) (D7A5) Rabbit Monoclonal Antibody Cell Signaling Technology Cat#3777
Ki-67 (8D5) Mouse Monoclonal Antibody Cell Signaling Technology Cat#9449
NF-"B p65 Recombinant monoclonal antibody Proteintech Cat#80979-1-RR
Phospho-NF-"B p65 (Ser536) Recombinant monoclonal antibody Proteintech Cat#80379-2-RR
Anti-Indoleamine 2, 3-dioxygenase antibody Abcam Cat#EPR20374
Beta Actin Monoclonal antibody Proteintech Cat#66009-1-Ig
ubiquitin Polyclonal Antibody Proteintech Cat#10201-2-AP
Anti-CPT1A antibody [8F6AE9] Abcam Cat#ab128568
TRIM21 Recombinant monoclonal antibody Proteintech Cat#83530-3-RR
Anti-Succinyllysine Mouse mAb PTMBio Cat#PTM-419
AhR (D5S6H) Rabbit Monoclonal Antibody Cell Signaling Technology Cat#83200
Histone H3 Polyclonal antibody Proteintech Cat#17168-1-AP
Alpha Tubulin Recombinant monoclonal antibody Proteintech Cat#80762-1-RR
PPARA Monoclonal antibody Proteintech Cat#66826-1-Ig
PGC-1 alpha (3G6) Rabbit Monoclonal Antibody Cell Signaling Technology Cat#2178
AMPK Alpha Polyclonal antibody Proteintech Cat#10929-2-AP
Phospho-AMPK Alpha (Thr172) Recombinant monoclonal antibody Proteintech Cat#80209-6-RR

	Na,K-ATPase alpha1 (D4Y7E) Rabbit Monoclonal Antibody Cell Signaling Technology Cat#23565<br>Calnexin Antibody Cell Signaling Technology Cat#2433<br>DCLK1 Polyclonal antibody Proteintech Cat#21699-1-AP<br>GEF-H1 Polyclonal antibody Proteintech Cat#24472-1-AP<br>Rabbit IgG control Polyclonal antibody Proteintech Cat#30000-0-AP<br>ATP5A1 Polyclonal antibody Proteintech Cat#14676-1-AP
Validation	Antibodies were selected based on manufacturer validation for the relevant species and applications. All primary antibodies were commercially sourced and selected on the basis of manufacturer validation for the relevant species and experimental applications. Antibodies were used at the working concentrations or dilutions recommended on the corresponding manufacturer product pages. Specificity of key antibodies was additionally supported by detection at the expected molecular weight. All the antibodies used are from commercial sources and have been validated by the vendors. Validation data are available on the manufacturer's website.

Eukaryotic cell lines

Policy information about cell lines and Sex and Gender in Research

Cell line source(s)	Human glioblastoma cell lines U118MG, A172, LN229 and T98G, together with HEK293T cells, were obtained from the American Type Culture Collection (ATCC). LN229-EGFRvIII and T98G-EGFRvIII cells were generated by stable transduction and validated by immunoblotting. The patient-derived EGFRvIII-positive GBM culture TBD0220, established from a male patient, was kindly provided by Professor Chunsheng Kang of Tianjin Medical University. The low-passage patient-derived GBM cultures LG-260 and LG-252 were established from freshly resected tumour specimens obtained from a male and a female patient, respectively, with written informed consent. The patient-derived glioblastoma organoids LG-25-05 and LG-24-03 were both established from male patients.
Authentication	Established cell lines were authenticated by short-tandem-repeat profiling and matched to reference profiles. The identity of the engineered EGFRvIII-expressing derivatives was confirmed by immunoblotting. Patient-derived cultures were verified using the available molecular pathological characteristics of the corresponding tumors.
Mycoplasma contamination	All cell cultures were tested for mycoplasma contamination using a PCR-based assay before use and at regular intervals during the study; all results were negative.
Commonly misidentified lines<br>(See ICLAC register)	No commonly misidentified cell lines were used.

Animals and other research organisms

Policy information about studies involving animals; ARRIVE guidelines recommended for reporting animal research, and Sex and Gender in Research

Laboratory animals	Six-week-old male BALB/c nude mice were obtained from Beijing Vital River Laboratory Animal Technology Co., Ltd. (Beijing, China), and housed under specific pathogen-free conditions at 22 ± 2 °C, 40-70% relative humidity, and a 12-h light/12-h dark cycle.
Wild animals	No wild animal was used in this study.
Reporting on sex	Only male mice were used in the present efficacy experiments. The study was not designed or powered to assess sex-dependent treatment effects.
Field-collected samples	The study did not involve samples collected from field.
Ethics oversight	The human primary GBM samples used in this study were obtained from Qilu Hospital of Shandong University according to a protocol approved by the Medical Ethics Committee of Qilu Hospital of Shandong University. All participants provided written informed consent. All animal experiments were approved by the Ethics Committee On Animal Experiment of Shandong University Qilu Hospital (protocol no. DWLL-2024-020) and were performed in accordance with the approved institutional guidelines.

Note that full information on the approval of the study protocol must also be provided in the manuscript.

Clinical data

Policy information about clinical studies

All manuscripts should comply with the ICMJE guidelines for publication of clinical research and a completed CONSORT checklist must be included with all submissions.

Clinical trial registration	Not applicable. The clinical component comprised a retrospective observational cohort and two individually treated compassionate-use cases. The compassionate-use treatments were not conducted under a prospectively designed clinical trial protocol and are reported descriptively under institutional ethics approval.
Study protocol	This study comprised a retrospective observational analysis of the TEFT-GBM cohort and an exploratory compassionate-use component; it was not a prospective interventional clinical trial. Following multidisciplinary assessment and written informed consent, two patients with recurrent EGFR-driven glioblastoma who had exhausted standard treatment options received osimertinib at 80 mg orally once daily in combination with TEFT. Clinical and radiological responses were evaluated longitudinally using RANO criteria.

Data collection	Clinical data for the retrospective TEFT-GBM cohort were extracted from the electronic medical records of patients treated at the Department of Neurosurgery, Qilu Hospital of Shandong University, between 2021 and the prespecified data-cutoff date. Collected variables included demographic characteristics, treatment history, TEFT exposure and compliance, histopathological and molecular findings, longitudinal MRI findings, progression-free survival and follow-up status. Eligibility and clinical records were centrally reviewed according to predefined criteria. For the two patients receiving TEFT plus osimertinib on a compassionate-use basis, treatment, imaging, clinical response and follow-up data were prospectively documented during routine clinical care from July to December 2025. Sex was obtained from the medical records.
Outcomes	Clinical outcomes were assessed using the Response Assessment in Neuro-Oncology (RANO) criteria based on longitudinal MRI examinations. Outcomes included radiological response and progression-free survival (PFS).

Plants

Seed stocks	*Report on the source of all seed stocks or other plant material used. If applicable, state the seed stock centre and catalogue number. If plant specimens were collected from the field, describe the collection location, date and sampling procedures.*
Novel plant genotypes	*Describe the methods by which all novel plant genotypes were produced. This includes those generated by transgenic approaches, gene editing, chemical/radiation-based mutagenesis and hybridization. For transgenic lines, describe the transformation method, the number of independent lines analyzed and the generation upon which experiments were performed. For gene-edited lines, describe the editor used, the endogenous sequence targeted for editing, the targeting guide RNA sequence (if applicable) and how the editor was applied.*
Authentication	*Describe any authentication procedures for each seed stock used or novel genotype generated. Describe any experiments used to assess the effect of a mutation and, where applicable, how potential secondary effects (e.g. second site T-DNA insertions, mosiacism, off-target gene editing) were examined.*

Flow Cytometry

Plots

Confirm that:

- [x] The axis labels state the marker and fluorochrome used (e.g. CD4-FITC).
- [x] The axis scales are clearly visible. Include numbers along axes only for bottom left plot of group (a 'group' is an analysis of identical markers).
- [ ] All plots are contour plots with outliers or pseudocolor plots.
- [x] A numerical value for number of cells or percentage (with statistics) is provided.

Methodology

Sample preparation	Cells were harvested at approximately 80% confluence. For adherent cells (e.g., GBM cell lines), cells were detached using 0.25% Trypsin-EDTA , neutralized with complete medium, and centrifuged at 300g for 5 min. Pellets were washed twice with ice-cold PBS.
Instrument	NovoCyte Quanteon 4020
Software	NovoExpress 1.6.2<br>FlowJo v10
Cell population abundance	No cell sorting was performed. For analytical flow cytometry, at least 5000 events were acquired per sample after exclusion of debris and doublets.
Gating strategy	The gating strategy was applied consistently across experiments, with separate workflows used for intact-cell and isolated-mitochondrial analyses. For intact-cell flow cytometry, cells were first identified on forward-scatter area versus side-scatter area (FSC-A versus SSC-A) plots to exclude debris and small particles. Doublets and aggregates were then excluded using FSC-H versus FSC-W and SSC-H versus SSC-Wplots, and all subsequent analyses were performed on the resulting singlet population. For apoptosis assays, quadrant boundaries were established using unstained and single-stained controls to distinguish viable (Annexin V-/PI-), early apoptotic (Annexin V+/PI-), and late apoptotic or necrotic (Annexin V+/PI+) cells. For cell-cycle analysis, PI fluorescence intensity was evaluated within the singlet population, and the proportions of cells in the G0/G1, S, and G2/M phases were determined using the Watson (Pragmatic) model in FlowJo. For TMRM-based assessment of mitochondrial membrane potential, fluorescence distributions and median fluorescence intensity values were quantified relative to the corresponding untreated or biological negative controls.For mitochondrial flow cytometry, isolated mitochondrial preparations were first gated on the basis of their forward- and side-scatter characteristics to select the small particle population and exclude residual cellular debris. Mitochondrial identity and gating specificity were further confirmed by ATP5A staining. IDO1 and CPT1A fluorescence signals were subsequently quantified within the ATP5A-positive mitochondrial population. Where positive and negative populations were defined, gate boundaries were established using appropriate biological negative controls and were applied consistently to all samples within the same experiment.

- [x] Tick this box to confirm that a figure exemplifying the gating strategy is provided in the Supplementary Information.

Magnetic resonance imaging

Experimental design

Design type	Retrospective longitudinal assessment of clinically acquired structural brain MRI scans; no task-based or resting-state functional MRI design was used.
Design specifications	Serial MRI examinations obtained during routine clinical care were reviewed at baseline and during follow-up. Imaging time points were determined by the clinical treatment and follow-up schedule rather than by a research-specific block or event-related design.
Behavioral performance measures	Not applicable. No behavioral task or performance measure was acquired in association with MRI.

Acquisition

Imaging type(s)	Structural, contrast-enhanced clinical brain MRI.
Field strength	Clinical MRI examinations were performed on 1.5-T or 3.0-T scanners, depending on scanner availability and routine clinical practice.
Sequence & imaging parameters	Routine clinical brain MRI protocols included axial T2-weighted and T2-FLAIR sequences and pre- and post-gadolinium T1-weighted imaging. Acquisition parameters varied according to the clinical scanner and examination date. No research-specific standardized MRI acquisition protocol was applied.
Area of acquisition	Whole-brain imaging was acquired. Tumor regions were evaluated on contrast-enhanced T1-weighted and T2-FLAIR images according to the Response Assessment in Neuro-Oncology criteria.

Diffusion MRI ☐ Used ☒ Not used

Preprocessing

Preprocessing software	Images were reviewed using the institutional picture archiving and communication system. No research-specific preprocessing software was used.
Normalization	Not applicable. Images were not spatially normalized because no voxel-wise, group-level, or atlas-based MRI analysis was performed.
Normalization template	Not applicable.
Noise and artifact removal	No research-specific noise or artifact-removal procedure was applied. All MRI examinations included in the descriptive assessment were of sufficient diagnostic quality.
Volume censoring	Not applicable. No functional MRI time series or volume censoring was involved.

Statistical modeling & inference

Model type and settings	No voxel-wise, cluster-wise, or image-based statistical model was applied. Longitudinal radiological response was assessed descriptively by comparing follow-up MRI examinations with baseline imaging according to the Response Assessment in Neuro-Oncology (RANO) criteria.
Effect(s) tested	Changes in contrast-enhancing tumor burden and T2/FLAIR abnormalities were evaluated longitudinally relative to baseline imaging according to the RANO criteria. No MRI-specific hypothesis testing, voxel-wise inference, or multivariate imaging analysis was performed.

Specify type of analysis: ☐ Whole brain ☒ ROI-based ☐ Both

Anatomical location(s)	Tumor-associated regions were identified clinically on contrast-enhanced T1-weighted and T2/FLAIR images according to the RANO criteria. No automated segmentation, atlas-based labeling, or formal voxel-wise ROI analysis was performed.
Statistic type for inference (See Eklund et al. 2016)	Not applicable. No voxel-wise or cluster-wise statistical inference was performed.
Correction	Not applicable. No correction for multiple comparisons was required because no voxel-wise, cluster-wise, or image-based hypothesis testing was performed.

Models & analysis

n/a	Involved in the study
x	Functional and/or effective connectivity
x	Graph analysis
x	Multivariate modeling or predictive analysis
