# Supplementary material for: CPT1A-mediated IDO1 succinylation shapes EGFRvIII-driven resistance to tumor electric field therapy in glioblastoma
Source: Nat Commun. 2026 Sep 23;17:10277. doi: 10.1038/s41467-026-77927-w (PMC13620138; doi:10.1038/s41467-026-77927-w)
Supplement: Supplementary file 3 [file 41467_2026_77927_MOESM3_ESM.pdf]

nature portfolio

# Peer Review File

# CPT1A-mediated IDO1 Succinylation Shapes EGFRvIII-driven Resistance to Tumor Electric Field Therapy in Glioblastoma

Corresponding Author: Professor Gang Li

**This file contains all reviewer reports in order by version, followed by all author rebuttals in order by version.**

Version 0:

Reviewer comments:

Reviewer #1

(Remarks to the Author)
In this manuscript, the authors stacked up multiple signaling pathways, including NF-kappaB, TRIM21-mediated ubiquitination, AMPK, PPARa, CPT1a-induced protein succinylation, AhR, DCLK1, among others, in TEFT-induced IDO1 accumulation and therapeutic resistance of GBM harboring EGFR mutation. While integrating multiple signaling pathways is highly interesting and mechanistically ambitious, the analyses of the specific contribution of each pathway to IDO1-mediated TEFT resistance in EGFR-mutant GBM remain superficial and lack sufficient depth. Other weaknesses:
1. Uncertainty in selection bias of retrospective cohort. The clinical association between EGFRvIII and TEFT resistance is derived from a retrospective cohort with a relatively small sample size, which is vulnerable to selection bias due to treatment heterogeneity, and confounding variables.
2. EGFRvIII-GBM is a biologically/biochemically distinct subset. It is unclear whether the proposed NF-kappaB, AMPK-PPARa-CPT1a-IDO1-AhR axis represents a broadly relevant TEFT resistance or specific to EGFRvIII tumors.
3. Risks of over-interpretation. The authors proposed a mechanistic model with multi-signaling steps. It is very likely each step is only partially correlative rather than causally required.
4. Lack of stronger biochemical validation of the non-canonical CPT1a succinyltransferase activity. CPT1a is a classical rate-limiting enzyme in fatty acid oxidation. Additional evidence is needed to rule out indirect succinylation through altered succinyl-CoA pools, other known succinyltransferases, and non-enzymatic succinylation under metabolic stress.
5. The data on AhR regulation in the resistant progresses is not convincing.
6. The quality of Fig. 1c and Fig. 5A AhR panel is unacceptable for publication.

Reviewer #2

(Remarks to the Author)
Feng et al. investigate why a subset of GBM exhibits intrinsic resistance to tumor electric field therapy (TEFT). Using a retrospective clinical cohort together with cell and organoid models, they propose that EGFRvIII drives resistance by increasing IDO1 transcription through NF-κB. In parallel, TEFT-induced bioenergetic stress activates an AMPK-PPARα program that upregulates CPT1A, which the authors propose stabilizes IDO1 via succinylation and inhibition of TRIM21-mediated ubiquitination. Stabilized IDO1 is then suggested to activate AhR signaling to induce DCLK1/ARHGEF2 and preserve spindle/microtubule integrity during TEFT. Finally, the authors present preclinical evidence supporting osimertinib plus TEFT as a strategy to reverse resistance, along with a small proof-of-concept clinical experience.

Overall, the study is ambitious and conceptually interesting, with a plausible resistance axis linking metabolic adaptation to mitotic fidelity and an attractive translational hypothesis. The work is timely given the growing clinical use of TEFT across multiple cancer types and the continued uncertainty around its mechanism of action. However, major concerns regarding model choice, insufficient mechanistic localization/validation of the CPT1A-IDO1 interaction, and overinterpretation of a very limited clinical experience substantially reduce confidence in the generality and impact of the claims.

Major comments:

1. A major limitation is the heavy reliance on obsolete, high-passage GBM lines (e.g., LN229, T98G) for most mechanistic work. The only primary EGFRvIII line (TBD0220) appears early (Fig. 1C–D) but is not carried through the core mechanistic experiments (Figs. 2–5). Key conclusions (IDO1 dependence, CPT1A-driven IDO1 stabilization on the mitochondrial

membrane, and AhR–DCLK1/ARHGEF2 axis) should be reproduced in multiple low-passage, patient-derived GBM cultures with and without EGFRvIII (and ideally with defined EGFR alterations). At minimum, the central mechanistic nodes should be validated in TBD0220 plus additional independent patient-derived models.

2. CPT1A–IDO1 localization/interaction needs stronger support. The manuscript does not convincingly establish where CPT1A binds IDO1 under TEFT or whether this occurs at mitochondria versus other compartments. Colocalization is suggestive but not definitive (Fig. 4K, Fig. 6F). Strengthen this with higher-resolution, quantified localization (z-stacks with mitochondrial markers) and compartment-specific interaction assays (co-IP/PLA from mitochondrial vs cytosolic fractions with purity controls). Also map the binding interface and test binding-deficient mutants for loss of IDO1 stabilization under TEFT.

3. Clinical claims are overinterpreted. The case series is very small (n = 4), and biomarker-driven benefit is not supported. The manuscript frames resistance as EGFRvIII-specific, yet the vignettes include other EGFR alterations and at least one apparent responder without EGFRvIII (Fig. 6G–H), raising whether the phenotype reflects EGFRvIII or broader EGFR activation. Please clarify the biomarker hypothesis and align the framing (including the title); otherwise temper conclusions and present the clinical data as hypothesis-generating or expand the cohort.

4. If the translational goal is combination therapy, it would be valuable to define the minimum effective osimertinib dose required for resensitization under TEFT (in vitro and ideally in vivo), rather than relying on a single dose. This is especially relevant for tolerability in a TEFT-treated population.

5. Synergy should be quantified (e.g., Bliss/Loewe) rather than described qualitatively, particularly for the core claim that osimertinib “abrogates” resistance.

6. Given IDO1 is positioned as the key effector, the translational logic would be strengthened by showing that pharmacologic IDO1 inhibition (and genetic approaches in multiple patient-derived models) can phenocopy osimertinib-mediated resensitization under TEFT. This would also help distinguish whether osimertinib is acting strictly upstream via EGFR signaling versus through additional effects.

Minor comments:

1. The manuscript is dense and would benefit from tighter writing and clearer subheading of the main causal chain. A figure summarizing the model would be very helpful for the readers to follow.

2.Please ensure TEFT settings (frequency/intensity/exposure duration) are consistently reported across figures and methods, and that comparisons across experiments use matched parameters.

Reviewer #3

(Remarks to the Author)
This study presents a comprehensive and mechanically novel investigation into the resistance mechanisms of EGFRvIII-positive glioblastoma (GBM) against Tumor Electric Field Therapy (TEFT). The authors construct a compelling logical chain linking biophysical stress (TEFT) to metabolic reprogramming (AMPK-PPARa-CPT1A), post-translational modification (IDO1 succinylation), and finally to structural protection of the mitotic spindle (AhR-DCLK1/ARHGEF2). The integration of multi-omics data, biochemical validation, and a prospective clinical case series makes this a robust study. The findings regarding CPT1A’s moonlighting LSTase activity and the "metabolic-structural" coupling are particularly interesting. This work is very novel, high interests and well conducted. Written is clear. There are a few gaps regarding the generalization of the metabolic findings and the precise biochemical mechanism could be addressed to further improve the study.

1. Since TEFT relies on a physical mechanism (dipole disruption), the resulting bioenergetic stress should theoretically be universal. Do EGFRwt cells also exhibit the AMPK-PPARa-CPT1A metabolic shift upon TEFT treatment? The authors should discuss whether this is a pan-GBM response that is specifically exploited by EGVRvIII cells.
2. CPT1A is localized on the outer membrane of mitochondria, while Succinyl-CoA is generated in the matrix. Given the TEFT-induced mitochondrial dysfunction, the source of sufficient Succinyl-CoA is unclear. Furthermore, the topological mechanism is puzzling: does IDO1 translocate into the matrix, or is Succinyl-CoA transported out? Please clarify this spatial compartmentalization in the Discussion section and update the schematic model (Fig. 4M) accordingly.
3. CPT1A succinylates IDO1 at K377 under TEFT-induced metabolic stress. However, TEFT can also reduce mitochondrial membrane potential and ATP level. Did authors measure the succinyl CoA levels under TEFT? How succinyl CoA pools are maintained? Whether TEFT increases CPT1A LSTase activity specifically.
4. While Osimertinib reduces IDO1 mRNA via NF-kB (supply side), the paper focuses on protein stabilization (stability side). Does Osimertinib also prevent the TEFT-induced metabolic adaptation (i.e., blocking AMPK/CPT1A upregulation)? Clarifying whether the drug targets both transcription and the metabolic stress response would refine the therapeutic logic.
5. In vitro TEFT can generate heat. Did the authors monitor culture temperature to rule out Heat Shock Protein (HSP) activation as a confounder for protein stabilization? A brief confirmation of temperature control in the Materials and Methods is recommended.
6. To further corroborate the functional enzymatic activity of the stabilized IDO1, it would be strengthened to directly measure the concentration of Kynurenine in the culture supernatant (via ELISA or HPLC) in EGFRvIII cells treated with TEFT versus

controls
7. In Figure 3E authors analyze protein levels in isolated mitochondria using flow cytometry. Please provide a control histogram using a known mitochondrial marker (e.g., ATP5A / Complex V) to confirm the identity of the gated population.
8. Since TEFT induces mitochondrial stress and lipid peroxidation (Fig. 3L), treating cells with an ROS scavenger (like NAC) to see if it modulates IDO1 stabilization or cell survival would provide an insight into the redox-dependency of this mechanism.

Version 1:

Reviewer comments:

Reviewer #1

(Remarks to the Author)

Reviewer #2

(Remarks to the Author)
The authors have substantially revised the manuscript and addressed the majority of my prior comments. Key findings are now validated in TBD0220 and 2 additional low-passage patient-derived models, with added genetic and pharmacologic IDO1 studies, mitochondrial-fraction interaction assays, higher-resolution microscopy, recombinant succinylation experiments, kynurenine measurements, and Bliss synergy analysis. Together, these data provide consistent support for the proposed mechanism.

Some mechanistic details, including precise mapping of the CPT1A–IDO1 interaction interface and its submitochondrial topology, remain unresolved, but these are appropriate for future studies rather than required for publication. The clinical claims are now appropriately restrained, with the cohort and compassionate-use cases presented as exploratory. Larger prospective studies will be needed to confirm these findings but are beyond the scope of the current work. The in vitro osimertinib dose-response studies address my prior comment, while in vivo dose optimization can be pursued in future preclinical work before clinical testing.

In my view, the authors also responded appropriately to the other reviewers' concerns. The distinction between a broadly induced TEFT metabolic-stress response and EGFRvIII-dependent IDO1 priming is now clearer. The recombinant succinylation assays, kynurenine measurements, mitochondrial gating controls, thermal controls, and integrated mechanistic model all strengthen the work.
Overall, the revision is materially stronger and has passed the bar for the major concerns raised. I therefore support publication. Congratulations.

Reviewer #3

(Remarks to the Author)
The authors have provided a comprehensive revision that completely answers all the concerns. I do not have any questions and prefer acceptance for publishing.

**Open Access** This Peer Review File is licensed under a Creative Commons Attribution 4.0 International License, which permits use, sharing, adaptation, distribution and reproduction in any medium or format, as long as you give appropriate credit to the original author(s) and the source, provide a link to the Creative Commons license, and indicate if changes were made.
In cases where reviewers are anonymous, credit should be given to 'Anonymous Referee' and the source.
The images or other third party material in this Peer Review File are included in the article's Creative Commons license, unless indicated otherwise in a credit line to the material. If material is not included in the article's Creative Commons license and your intended use is not permitted by statutory regulation or exceeds the permitted use, you will need to obtain permission directly from the copyright holder.
To view a copy of this license, visit https://creativecommons.org/licenses/by/4.0/

# Response Letter to Reviewers

## Responses to the comments of Reviewer #1

Reviewer 1 Comments to the Author: In this manuscript, the authors stacked up multiple signaling pathways, including NF-kappaB, TRIM21-mediated ubiquitination, AMPK, PPARa, CPT1a-induced protein succinylation, AhR, DCLK1, among others, in TEFT-induced IDO1 accumulation and therapeutic resistance of GBM harboring EGFR mutation. While integrating multiple signaling pathways is highly interesting and mechanistically ambitious, the analyses of the specific contribution of each pathway to IDO1-mediated TEFT resistance in EGFR-mutant GBM remain superficial and lack sufficient depth.

**Response**: We thank the reviewer for this important and constructive criticism. We agree that, in the original manuscript, the presentation of multiple signaling pathways could give the impression of a broadly assembled and predominantly correlative cascade, without sufficiently distinguishing the specific contribution of each pathway to TEFT resistance.

In the revised manuscript, we have therefore reorganized the mechanistic framework around a genotype–field convergence model, in which the major pathways are assigned distinct and experimentally testable functions rather than being presented as parallel associations. Specifically, EGFRvIII provides a transcriptional input that increases the availability of IDO1, whereas TEFT induces a broadly shared metabolic-stress response that promotes CPT1A-dependent post-translational stabilization of IDO1. The stabilized IDO1 protein then supports a downstream cytoskeletal protection program. To strengthen this model, we added complementary genetic, pharmacological, biochemical, spatial, and functional perturbation experiments.

**1. EGFRvIII–NF-κB signaling provides the transcriptional input for IDO1.**

We first clarified the contribution of the EGFRvIII–NF-κB axis. EGFRvIII expression was associated with increased NF-κB/RelA activity and RelA occupancy at the predicted IDO1 promoter regions. Pharmacological inhibition of NF-κB with JSH-23, as well as upstream EGFR inhibition with osimertinib, reduced IDO1 mRNA expression. These findings define NF-κB as the transcriptional link through which EGFRvIII primes the IDO1 supply arm of the resistance program.

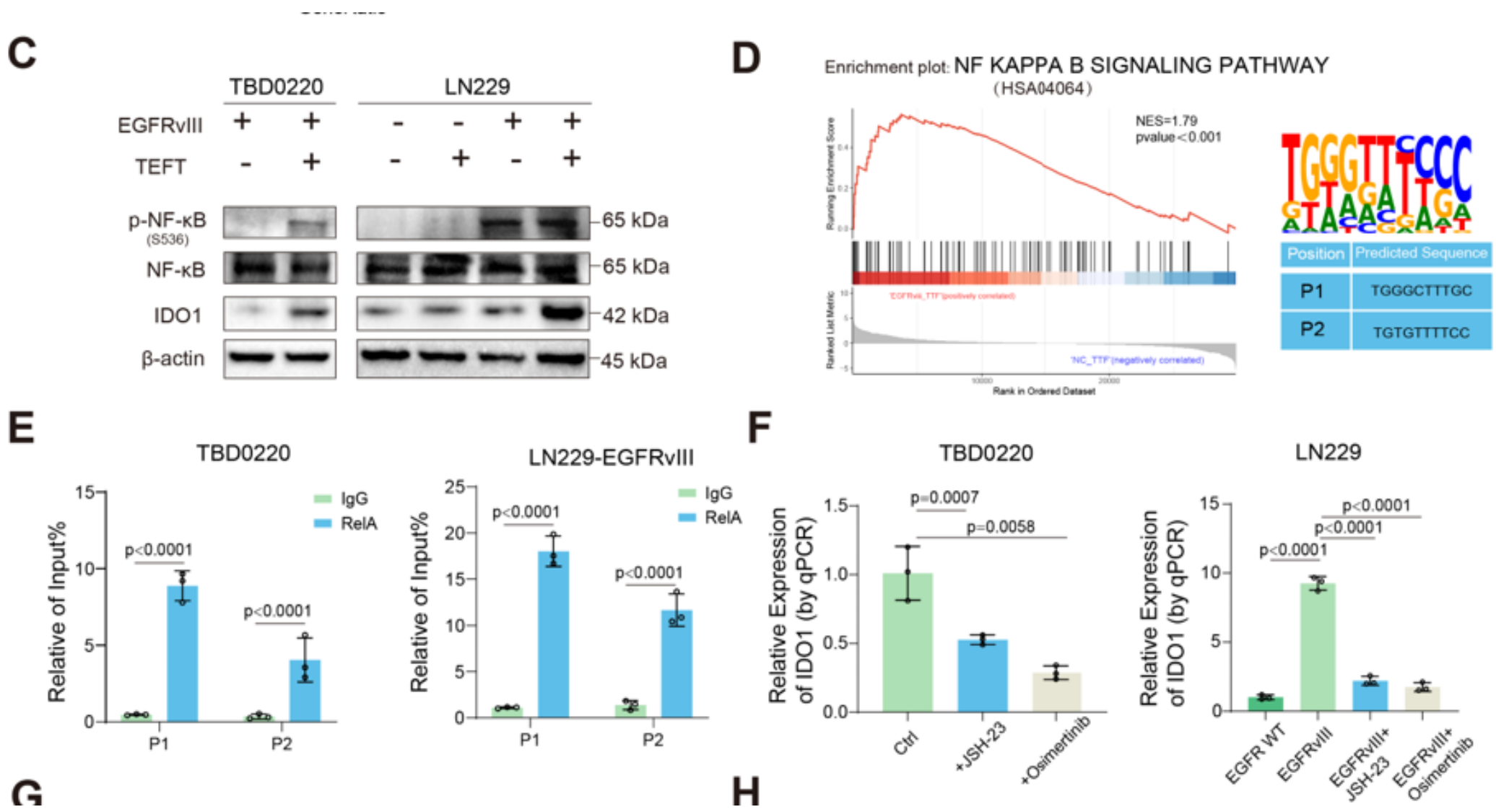


**Fig. 2. (C)** Western blot analysis of phosphorylated NF-κB (p-p65), total NF-κB (p65), showing IDO1 in TBD0220 cells with or without TEFT treatment,and in LN229 cells with or without EGFRvIII expression and TEFT treatment. **(D)** GSEA enrichment plot confirming the activation of the NF-κB signaling pathway in EGFRvIII cells. Right: Motif logo and predicted binding sequences (P1, P2) of NF-κB on the IDO1 promoter, **(E)** ChIP-qPCR analysis of RelA (p65) occupancy at the predicted IDO1 promoter regions (P1 and P2) in TBD0220 and LN229 cells. Data are presented as percent of input, **(F)** RT-qPCR analysis of IDO1 mRNA levels in EGFRvIII-expressing cells treated with the NF-κB inhibitor JSH-23 or osimertinib.

2. TEFT induces a broadly shared AMPK-PPARα-CPT1A metabolic-stress response.

We next distinguished the genotype-dependent transcriptional input from the metabolic response induced by TEFT. TEFT caused mitochondrial depolarization, mitochondrial calcium accumulation, lipid oxidative stress, AMPKα phosphorylation, PPARα–PGC1α engagement, and CPT1A upregulation not only in EGFRvIII-driven cells, but also in parental LN229 and T98G cells. Importantly, osimertinib did not abolish TEFT-induced AMPKα phosphorylation or CPT1A accumulation. These results indicate that EGFR inhibition does not suppress the primary metabolic-stress response to TEFT, but instead preferentially disrupts the EGFRvIII-dependent IDO1 transcriptional input.

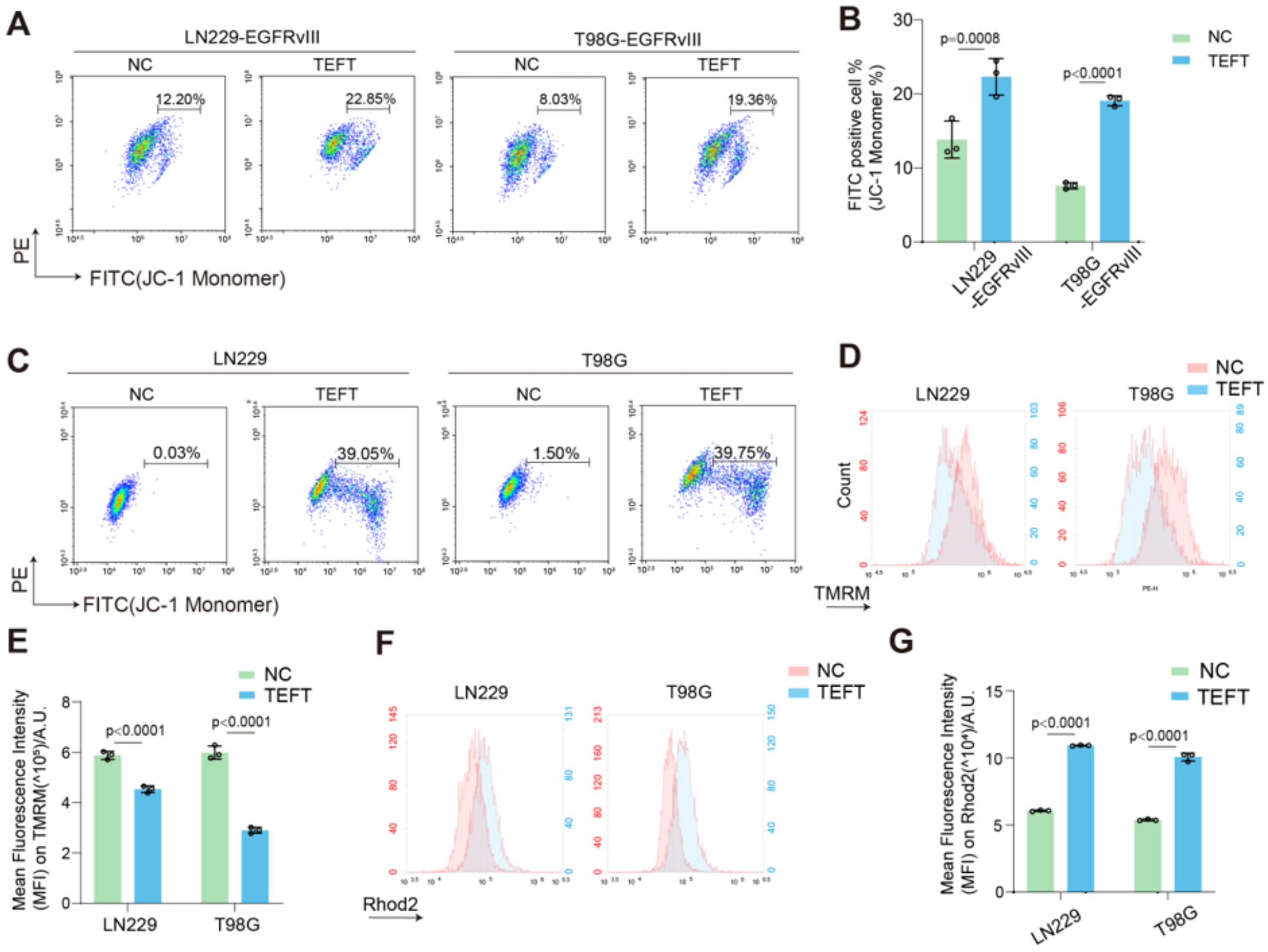


**fig. S5. (A)** Representative flow cytometry dot plots of JC-1 staining in LN229-EGFRvIII and T98G-EGFRvIII cells treated with or without TEFT. Increased FITC-positive JC-1 monomer signal indicates mitochondrial depolarization, **(B)** Quantification of JC-1 monomer-positive cells in LN229-EGFRvIII and T98G-EGFRvIII cells, **(C)** Representative flow cytometry dot plots of JC-1 staining in parental LN229 and T98G cells treated with or without TEFT, **(D)** Representative flow cytometry histograms of mitochondrial membrane potential, assessed by TMRM staining, in parental LN229 and T98G cells treated with or without TEFT, **(E)** Quantification of TMRM mean fluorescence intensity in parental LN229 and T98G cells, **(F)** Representative flow cytometry histograms of Rhod-2 staining in parental LN229 and T98G cells treated with or without TEFT, **(G)** Quantification of Rhod-2 mean fluorescence intensity in parental LN229 and T98G cells.

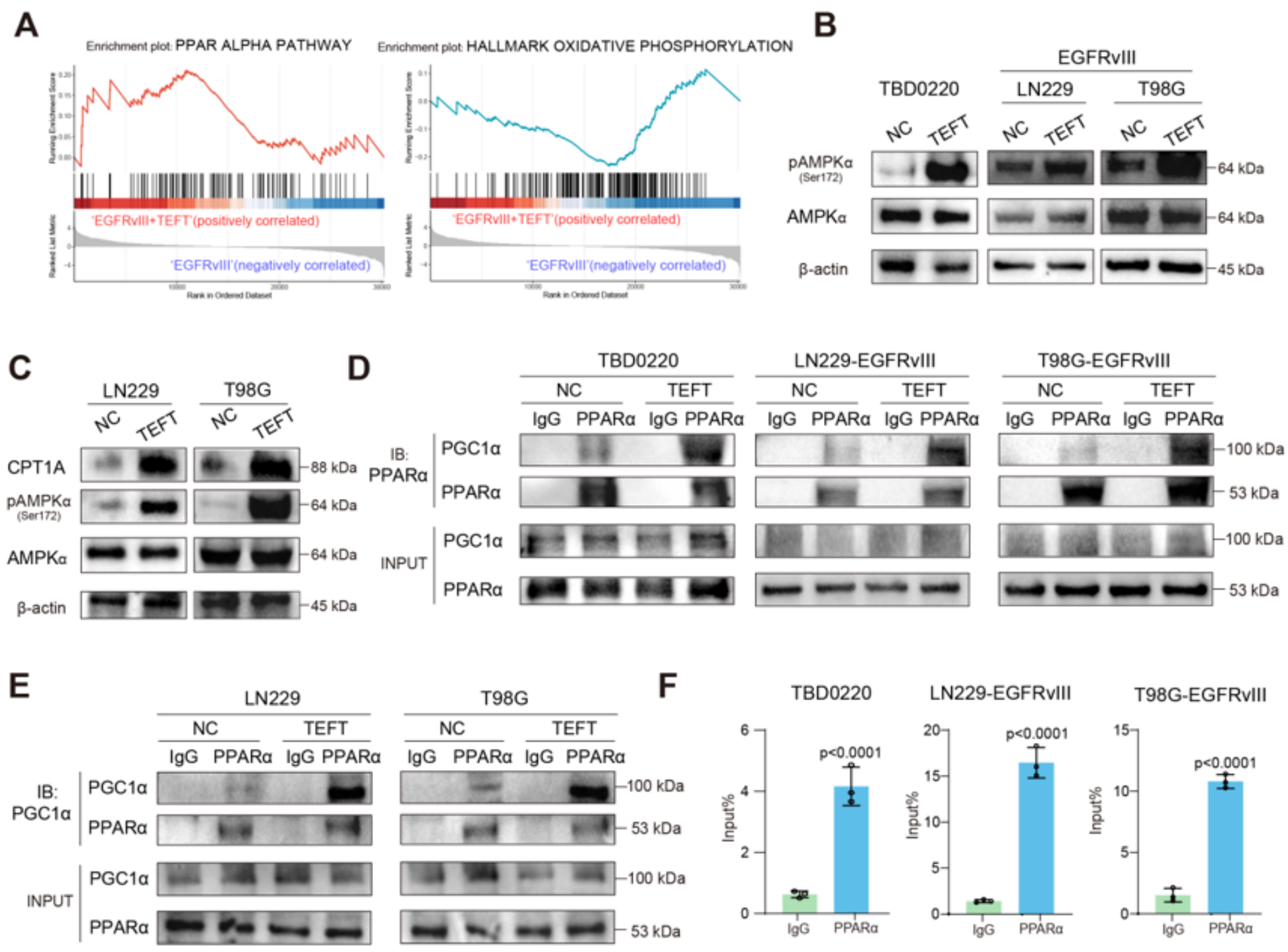


**fig. S6. (A)** GSEA enrichment plots showing positive enrichment of the PPARα pathway and negative enrichment of oxidative phosphorylation signatures in EGFRvIII cells treated with TEFT, **(B)** Western blot analysis of phosphorylated AMPKα (p-AMPKα, Ser172) and total AMPKα in TBD0220, LN229-EGFRvIII, and T98G-EGFRvIII cells treated with or without TEFT, **(C)** Western blot analysis of CPT1A, phosphorylated AMPKα, and total AMPKα in parental LN229 and T98G cells treated with or without TEFT. Data show that TEFT-induced AMPK-CPT1A activation is also observed in parental GBM cells, **(D)** Co-immunoprecipitation analysis of the interaction between PPARα and its transcriptional co-activator PGC1α in TBD0220, LN229-EGFRvIII, and T98G-EGFRvIII cells treated with or without TEFT, **(E)** Co-immunoprecipitation analysis of PPARα–PGC1α interaction in parental LN229 and T98G cells treated with or without TEFT, **(F)** ChIP-qPCR analysis of PPARα occupancy at predicted PPRE motifs within the CPT1A promoter region in TBD0220, LN229-EGFRvIII, and T98G-EGFRvIII cells. Data are presented as percentage of input.

3. CPT1A promotes post-translational stabilization of IDO1 through its LSTase-related activity.

We further strengthened the biochemical evidence defining the contribution of CPT1A. CPT1A depletion reduced IDO1 succinylation and increased IDO1 ubiquitination. Re-expression of wild-type CPT1A or the LSTase-retaining/CPTase-deficient G710E mutant restored IDO1 succinylation, whereas the H473A mutant with reduced LSTase-related activity did not. In addition, cell-free assays using purified recombinant CPT1A and IDO1 demonstrated CPT1A-dependent IDO1 succinylation in the presence of succinyl-CoA. Together, these experiments establish that CPT1A contributes to resistance not only through metabolic adaptation, but also through a non-canonical post-translational mechanism that promotes IDO1 stability.

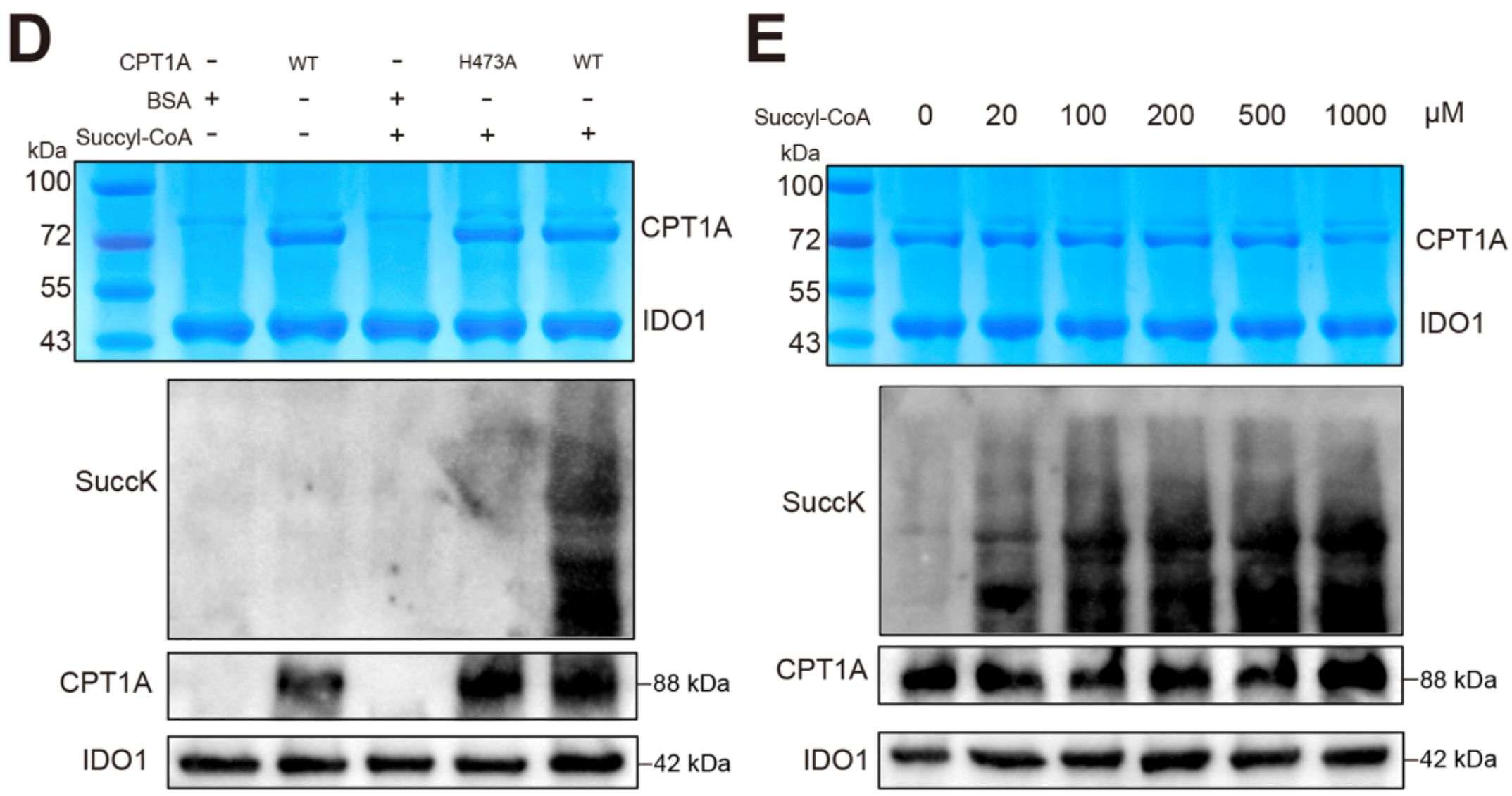


**fig. S8. (D)** In vitro recombinant protein succinylation assay using purified IDO1 and CPT1A proteins. Wild-type CPT1A promoted IDO1 lysine succinylation in the presence of succinyl-CoA, whereas the catalytically defective H473A mutant showed reduced activity, **(E)** Dose-dependent in vitro succinylation assay showing increased IDO1 lysine succinylation with increasing succinyl-CoA concentrations in the presence of recombinant CPT1A.

4. IDO1 and its downstream cytoskeletal effectors are functionally required for the resistant phenotype.

To determine whether IDO1 and the downstream pathway were functionally relevant rather than merely associated with resistance, we performed both genetic and pharmacological perturbations. Two independent IDO1-targeting siRNAs increased TEFT-induced apoptosis and restored TEFT-associated mitotic perturbation. Pharmacological inhibition of IDO1 enzymatic activity with epacadostat produced a similar resensitizing effect in multiple EGFRvIII-driven models, including patient-derived cells. Downstream, DCLK1 depletion disrupted spindle organization, whereas depletion of IDO1 or ARHGEF2, as well as treatment with epacadostat or osimertinib, reduced the enhanced tubulin-polymerizing activity associated with the resistant phenotype. Kynurenine production, AhR nuclear accumulation, AhR target-gene induction, and AhR occupancy at predicted regulatory regions of DCLK1 and ARHGEF2 further support the involvement of an IDO1–kynurenine–AhR-associated cytoskeletal protection program.

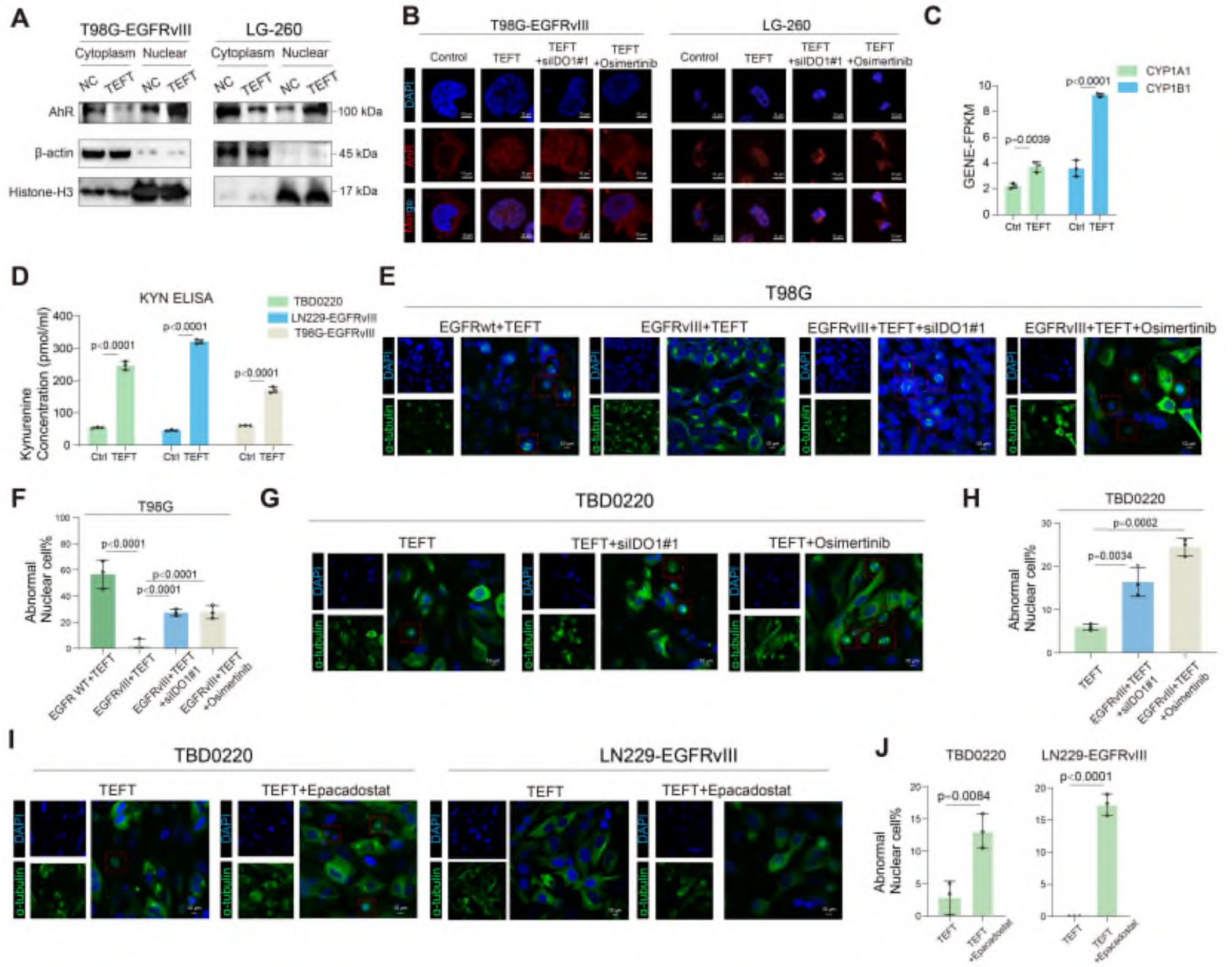

**fig. S10. (A)** Western blot analysis of AhR in cytoplasmic and nuclear fractions of T98G-EGFRvIII and patient-derived LG-260 cells treated with or without TEFT, (**B**) Representative immunofluorescence images of AhR in T98G-EGFRvIII and LG-260 cells under the indicated conditions. TEFT induced AhR nuclear accumulation, whereas IDO1 knockdown or osimertinib treatment reduced AhR nuclear localization. DAPI, nuclei; AhR, red. Scale bars, 10 µm, (**C**) Gene expression levels of canonical AhR target genes CYP1A1 and CYP1B1 in control and TEFT-treated EGFRvIII-LN229 cells, shown as FPKM values, (**D**) Kynurenine ELISA showing increased extracellular kynurenine levels in the culture supernatants of TBD0220, LN229-EGFRvIII, and T98G-EGFRvIII cells after TEFT treatment, (**E**) Representative immunofluorescence images of α-tubulin staining in T98G cells under the indicated conditions. TEFT induced spindle and nuclear abnormalities in EGFR wild-type cells, whereas EGFRvIII cells maintained a more preserved microtubule architecture under TEFT. This protective phenotype was disrupted by IDO1 knockdown or osimertinib treatment. DAPI, nuclei; α-tubulin, green. Scale bars, 10 µm, (**F**) Quantification of cells with abnormal nuclear morphology in T98G cells under the indicated conditions, (**G**) Representative immunofluorescence images of α-tubulin staining in TBD0220 cells treated with TEFT, TEFT plus IDO1 knockdown, or TEFT plus osimertinib. IDO1 knockdown or osimertinib disrupted the TEFT-resistant spindle-protective phenotype. DAPI, nuclei; α-tubulin, green. Scale bars, 10 µm, (**H**) Quantification of cells with abnormal nuclear morphology in TBD0220 cells under the indicated conditions, (**I**) Representative immunofluorescence images of α-tubulin staining in TBD0220 and LN229-EGFRvIII cells treated with TEFT alone or TEFT plus epacadostat. Pharmacological IDO1 inhibition disrupted the spindle-protective phenotype under TEFT exposure. DAPI, nuclei; α-tubulin, green. Scale bars, 10 µm, (**J**) Quantification of cells with abnormal nuclear morphology in TBD0220 and LN229-EGFRvIII cells after TEFT treatment with or without epacadostat.

Taken together, these data support a mechanistically connected model in which the individual pathways make distinct contributions to TEFT resistance:

We have revised the Results and Discussion accordingly to distinguish these functional modules, to specify the experimental evidence supporting each major mechanistic link. Representative results are shown above, the complete experimental data, statistical analyses, and corresponding interpretation are provided in the revised manuscript.

Other weaknesses: 1. Uncertainty in selection bias of retrospective cohort. The clinical association between EGFRvIII and TEFT resistance is derived from a retrospective cohort with a relatively small sample size, which is vulnerable to selection bias due to treatment heterogeneity, and confounding variables.

**Response:** We agree that the retrospective, single-center design and modest cohort size limit the strength of the clinical inference and may introduce selection bias, treatment heterogeneity, and residual confounding. To better contextualize our clinical observation, we expanded the Introduction by incorporating independent clinical studies that reported related associations between EGFR status and outcomes in patients receiving electric field therapy. For example, Pandey et al. reported that patients with EGFR wild-type tumors may derive greater progression-free survival benefit from TEFT **(Pandey et al., 2022, https://doi.org/10.1093/noajnl/vdac096)**. In addition, a clinical study from Shanghai, China, suggested that EGFR amplification may serve as a predictive factor for progression-free survival in patients treated with electric field therapy **(Hong et al., 2024, DOI: 10.3969/j.issn.1672-7770.2024.01.002, (https://www.scribd.com/document/998235473/))**. These independent observations do not eliminate the inherent limitations of our retrospective cohort, but they support the clinical plausibility of our findings and provide a rationale for our subsequent mechanistic investigation.

We have revised the manuscript in four ways. First, the cohort is now explicitly described as consecutively and retrospectively enrolled, with standardized TEFT exposure and centrally reviewed molecular profiles. Second, the association between EGFRvIII status and progression-free survival is presented as hypothesis-generating rather than as evidence that EGFRvIII independently predicts TEFT resistance. Third, the Introduction now places our observation in the context of independent reports suggesting that EGFR-related alterations may influence outcomes during electric-field therapy. Fourth, the Discussion contains an explicit limitation statement noting that neither the retrospective cohort nor the exploratory compassionate-use cases can establish a validated predictive biomarker or demonstrate clinical efficacy.

The clinical cohort is therefore used as the starting observation for the mechanistic study, whereas the functional contribution of EGFRvIII signaling is supported primarily by the subsequent cell-based, patient-derived, organoid, and orthotopic experiments. Accordingly, adequately powered prospective, multicenter, biomarker-stratified studies will be essential to establish whether EGFRvIII and broader EGFR pathway alterations

can serve as clinically actionable predictive biomarkers of TEFT response and guide the selection of patients most likely to benefit from TEFT-based combination therapy.

**Changes in the revised manuscript:**
**Abstract, lines 21-23; 32-34**

In a retrospective cohort of TEFT-treated GBM, EGFRvIII was associated with shorter progression-free survival, prompting us to investigate a genotype-linked resistance mechanism.

Osimertinib suppressed the EGFRvIII-NF-κB-IDO1 arm and enhanced TEFT efficacy, while exploratory clinical cases provided preliminary mechanism-informed support for the combination in recurrent EGFR-driven GBM.

**Introduction, lines 59-67;**

Through a retrospective analysis of a single-center clinical cohort, we observed that patients harboring EGFRvIII alterations had shorter progression-free survival compared with their EGFRvIII-wild-type counterparts when treated with electric field therapy. Although this observation is limited by the retrospective design and modest cohort size, it is consistent with emerging clinical evidence suggesting that EGFR-related alterations may influence the therapeutic benefit of electric field therapy, including reports that EGFR wild-type status or EGFR amplification may stratify progression-free survival in TEFT-treated GBM patients(*19*). These clinical observations motivated us to investigate whether and how EGFRvIII mechanistically contributes to TEFT resistance in GBM.

**Results, lines 92–104;**

Since 2021, the Department of Neurosurgery at Qilu Hospital of Shandong University has established a real-world "TEFT-GBM" cohort. We consecutively and retrospectively enrolled patients with histologically confirmed glioblastoma who received TEFT after standard Stupp protocol. By the data-cutoff date, 31 patients with complete clinical and molecular profiles were included (median age 59 years, range 28-75; 16 males and 15 females). All patients received TEFT at a field intensity of 2 V $cm^{-1}$ and a frequency of 200 kHz, with treatment compliance of ≥18 h $day^{-1}$. Central neuropathology review identified EGFRvIII alterations in 9 cases (29.0%). Survival analysis showed that the EGFRvIII-altered group had a shorter median progression-free survival than the EGFRvIII-wild-type group (12.97 versus 16.60 months; HR, 3.144; 95% CI, 1.136-8.700; P = 0.0482) (Fig. 1A, B). Given the retrospective nature and limited size of this cohort, this association should be interpreted as hypothesis-generating rather than definitive evidence that EGFRvIII independently predicts TEFT resistance. Nevertheless, these clinical findings warranted further mechanistic investigation.

**Discussion and Limitations, lines 541–567;**

This strategy exploits complementary mechanisms: TEFT exerts physical pressure on the mitotic spindle, whereas osimertinib prevents EGFR-driven tumor cells from efficiently converting metabolic adaptation into IDO1-AhR-dependent cytoskeletal protection. Based on this rationale, our preliminary clinical observations in Cases #3 and #4 provide mechanism-informed proof-of-concept support that osimertinib may enhance TEFT responsiveness in recurrent EGFR-driven GBM. However, these observations

remain exploratory because of the small number of patients and treatment heterogeneity and therefore require prospective biomarker-guided validation.

Our study has limitations that point toward critical avenues for future research. First, the association between EGFRvIII status and reduced TEFT benefit was derived from a retrospective single-center cohort, and the clinical observations involving osimertinib plus TEFT were based on a small exploratory case series. These findings are hypothesis-generating and mechanism-informed but cannot establish EGFR alterations as definitive predictive biomarkers or demonstrate clinical efficacy of the combination. Larger prospective, biomarker-guided studies are required. Second, although TEFT-induced mitochondrial stress and AMPK-PPARα-CPT1A activation were observed in both EGFRvIII-driven and parental GBM cells, it remains unknown whether this metabolic program becomes a general mechanism of acquired resistance across molecular subtypes. Longitudinal TEFT exposure models and pan-genotypic patient-derived systems will be needed to determine how broadly this adaptive response applies and whether CPT1A represents a shared therapeutic vulnerability. Third, although we mapped the CPT1A LSTase-related activity, the precise source and submitochondrial accessibility of the succinyl-CoA donor require further biochemical dissection. Although total cellular succinyl-CoA was not depleted under TEFT, direct submitochondrial metabolite tracing will be necessary to test the proposed CPT1A-associated outer mitochondrial membrane microdomain model. Finally, the safety and efficacy of TEFT combined with osimertinib require prospective clinical evaluation, including optimization of osimertinib dose, treatment timing, tolerability, and molecular patient selection.

### Methods, lines 569–583;

**Clinical Cohorts and Patient Samples**

Retrospective TEFT-GBM Cohort: Patients with histologically confirmed glioblastoma (GBM) treated at the Department of Neurosurgery, Qilu Hospital of Shandong University between 2021 and the data-cutoff date were consecutively and retrospectively enrolled. Inclusion criteria required patients to have completed the standard Stupp protocol followed by tumor electric field therapy with a compliance of ≥18 hours/day at a field intensity of 2 V/cm. Clinical data and molecular profiles, including EGFR amplification and EGFRvIII alteration status, were collected and centrally reviewed. Two patients with recurrent EGFR-driven GBM received compassionate-use salvage treatment with osimertinib (80 mg, orally once daily) and TEFT. Both patients provided written informed consent for treatment and publication of their clinical data and imaging findings. Treatment response was assessed according to RANO criteria based on longitudinal MRI scans. Human Tissue and Organoids: Primary GBM tissue samples were obtained with written informed consent. Patient-derived organoids (GBOs) were established from recurrent GBM patients. The study was approved by the Institutional Review Board (IRB) of Qilu Hospital of Shandong University.

2. EGFRvIII-GBM is a biologically/biochemically distinct subset. It is unclear whether the proposed NF-kappa B, AMPK-PPARa-CPT1a-IDO1-AhR axis represents a broadly relevant TEFT resistance or specific to EGFRvIII tumors.

**Response:** We agree that the original manuscript did not clearly distinguish the broadly induced cellular response to electric-field exposure from the genotype-dependent pathway that converts this response into resistance.

To address this issue, we performed additional experiments in parental EGFRvIII-negative LN229 and T98G cells. TEFT induced mitochondrial depolarization, mitochondrial calcium accumulation, and lipid oxidative stress in both parental cell lines. TEFT also increased AMPKα phosphorylation and CPT1A expression and enhanced the interaction between PPARα and its transcriptional co-activator PGC1α. These findings indicate that mitochondrial stress and activation of the AMPK–PPARα–CPT1A metabolic program are broadly inducible responses across the GBM models tested rather than EGFRvIII-specific events. The relevant changes made in the results have been added to figs. S5A-G and S6A-F.

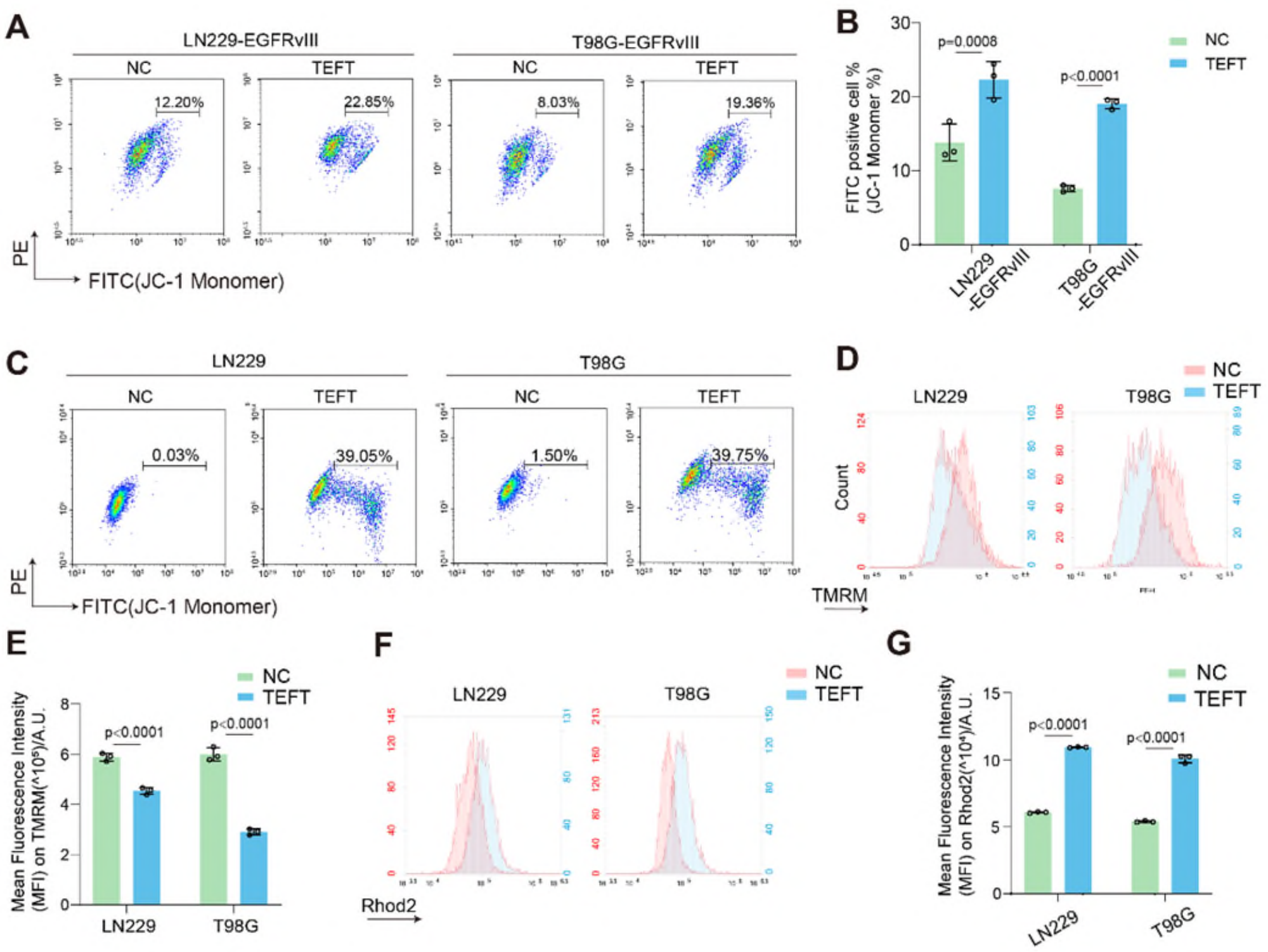


**fig. S5 (A)** Representative flow cytometry dot plots of JC-1 staining in LN229-EGFRvIII and T98G-EGFRvIII cells treated with or without TEFT. Increased FITC-positive JC-1 monomer signal indicates mitochondrial depolarization, **(B)** Quantification of JC-1 monomer-positive cells in LN229-EGFRvIII and T98G-EGFRvIII cells, **(C)** Representative flow cytometry dot plots of JC-1 staining in parental LN229 and T98G cells treated with or without TEFT, **(D)** Representative flow cytometry histograms of mitochondrial membrane potential, assessed by TMRM staining, in parental LN229 and T98G cells treated with or without TEFT, **(E)** Quantification of TMRM mean fluorescence intensity in parental LN229 and T98G cells, **(F)** Representative flow cytometry histograms of Rhod-2 staining in parental LN229 and T98G cells treated with or without TEFT, **(G)** Quantification of Rhod-2 mean fluorescence intensity in parental LN229 and T98G cells.

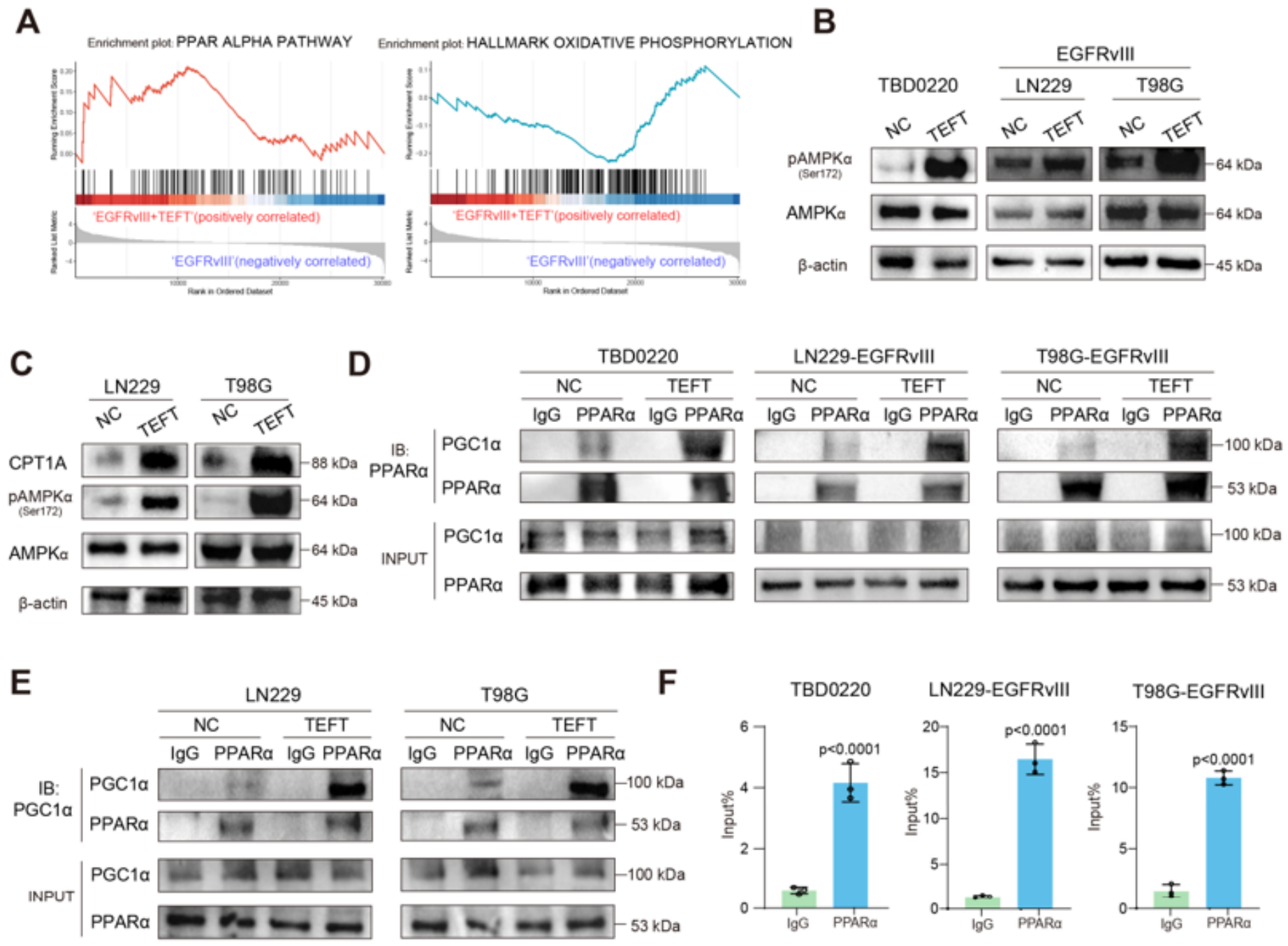

**fig. S6 (A)** GSEA enrichment plots showing positive enrichment of the PPARα pathway and negative enrichment of oxidative phosphorylation signatures in EGFRvIII cells treated with TEFT, **(B)** Western blot analysis of phosphorylated AMPKα (p-AMPKα, Ser172) and total AMPKα in TBD0220, LN229-EGFRvIII, and T98G-EGFRvIII cells treated with or without TEFT, **(C)** Western blot analysis of CPT1A, phosphorylated AMPKα, and total AMPKα in parental LN229 and T98G cells treated with or without TEFT. Data show that TEFT-induced AMPK-CPT1A activation is also observed in parental GBM cells, **(D)** Co-immunoprecipitation analysis of the interaction between PPARα and its transcriptional co-activator PGC1α in TBD0220, LN229-EGFRvIII, and T98G-EGFRvIII cells treated with or without TEFT, **(E)** Co-immunoprecipitation analysis of PPARα–PGC1α interaction in parental LN229 and T98G cells treated with or without TEFT, **(F)** ChIP-qPCR analysis of PPARα occupancy at predicted PPRE motifs within the CPT1A promoter region in TBD0220, LN229-EGFRvIII, and T98G-EGFRvIII cells. Data are presented as percentage of input.

In contrast, the complete IDO1-dependent resistance program required an additional genotype-linked input. In our models, EGFRvIII increased NF-κB/RelA activity and IDO1 transcription, thereby providing an IDO1 pool that could subsequently be stabilized by TEFT-induced CPT1A. Consistent with this distinction, osimertinib reduced the EGFRvIII–NF-κB–IDO1 transcriptional arm but did not abolish TEFT-induced AMPKα phosphorylation or CPT1A upregulation. The missing statistical annotations have been added to figs. S6G.

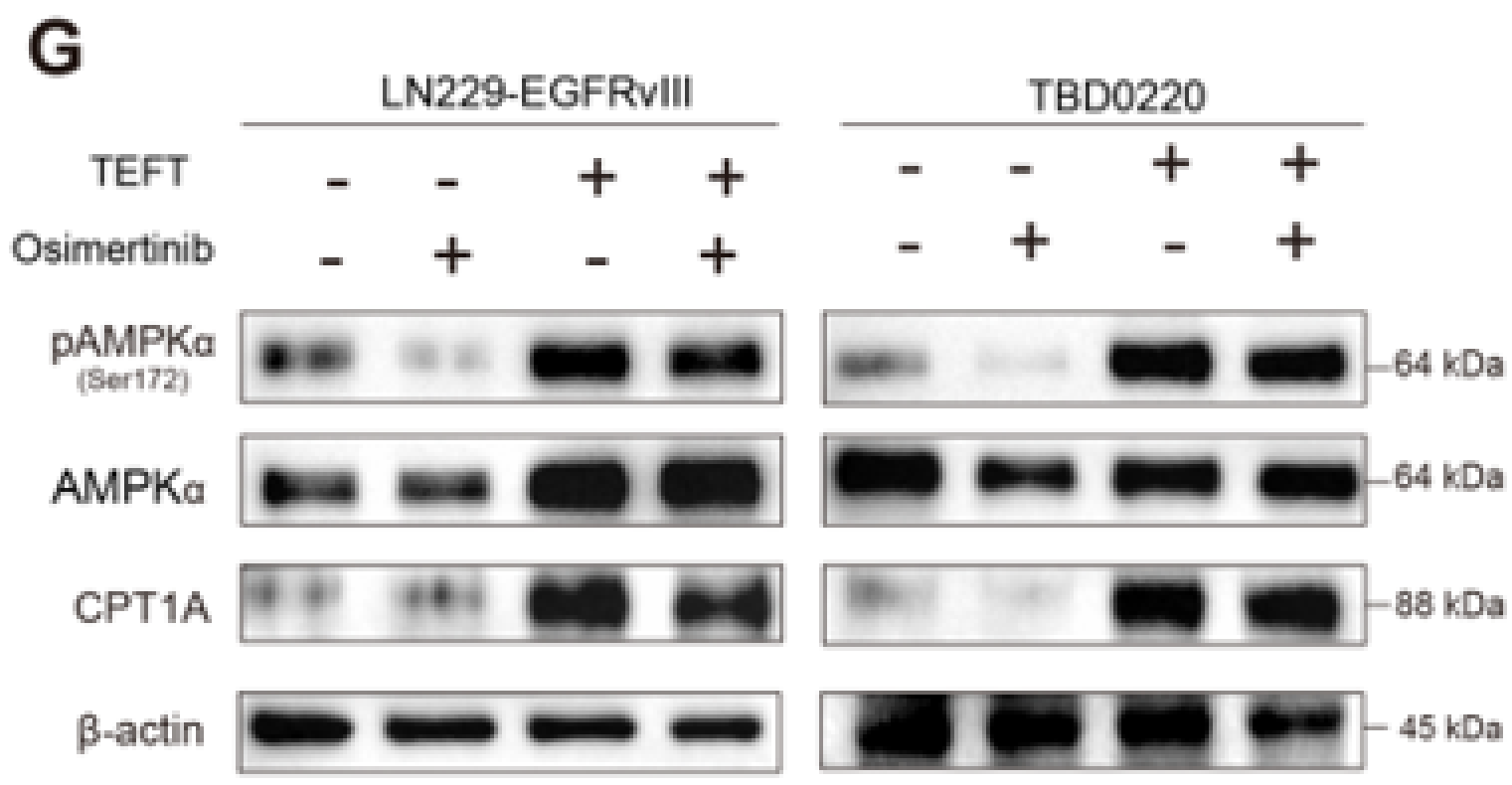


**fig. S8 (G)** Western blot analysis of the AMPK-CPT1A axis in LN229-EGFRvIII and TBD0220 cells treated with osimertinib, TEFT, or the combination. Osimertinib did not abolish TEFT-induced AMPKα phosphorylation or CPT1A upregulation.

We have therefore revised the manuscript to present a genotype–field convergence model. TEFT broadly induces the AMPK–PPARα–CPT1A metabolic-stress response, whereas EGFRvIII provides NF-κB-dependent IDO1 transcriptional priming. Their convergence enables CPT1A-dependent IDO1 stabilization and subsequent activation of the kynurenine–AhR cytoskeletal protection program. Thus, the upstream metabolic response may be broadly relevant, whereas the complete IDO1-dependent resistance program is preferentially engaged in EGFRvIII-driven or otherwise IDO1-primed GBM cells.

**Other Changes in the revised manuscript are provided below.**

**Results, lines 219-241;**

To determine whether this stress response was restricted to EGFRvIII-mutant cells, we examined parental LN229 and T98G cells. Additional JC-1 analyses confirmed mitochondrial depolarization in EGFRvIII-driven models (fig. S5A, B). In parental LN229 and T98G cells, TEFT induced mitochondrial depolarization and mitochondrial calcium accumulation, as shown by JC-1, TMRM, and Rhod-2 staining (fig. S5C-G). These results indicate that TEFT-induced mitochondrial and redox stress is broadly inducible across GBM cells rather than being restricted to EGFRvIII-driven models. GSEA analysis of the transcriptome provided mechanistic insight, showing negative enrichment of oxidative phosphorylation signatures but positive enrichment of the PPARα pathway (fig. S6A). Validating this cellular energy deficit, Western blotting showed activation of the energy sensor AMPK**,** evidenced by increased AMPKα phosphorylation in EGFRvIII-driven models (fig. S6B). TEFT also increased AMPKα phosphorylation and CPT1A expression in parental LN229 and T98G cells, further supporting a broadly induced metabolic-stress response (fig. S6C). Furthermore, Co-IP assays showed that TEFT enhanced the interaction between PPARα and its co-activator PGC1α in both EGFRvIII-driven and parental GBM cells (fig. S6D, E). ChIP-qPCR further showed increased PPARα occupancy at predicted PPRE motifs within the CPT1A promoter (fig. S6F). Together, these findings indicate that TEFT activates a broadly inducible AMPK-PPARα-CPT1A metabolic-stress program. However, this upstream response alone is insufficient to establish the complete IDO1-dependent resistance cascade, which also

requires EGFRvIII-mediated IDO1 transcriptional priming. Consistent with this distinction, osimertinib did not abolish TEFT-induced AMPKα phosphorylation or CPT1A upregulation (fig. S6G), suggesting that EGFR inhibition primarily disrupts the IDO1 supply arm rather than the upstream metabolic-stress response.

**Discussion, lines 442-475;**

While the updated Stupp regimen combined with TEFT has improved survival in GBM, the heterogeneity of clinical responses remains a critical challenge. Our study identifies EGFRvIII not merely as an oncogenic driver, but as an enabling component within a multi-layered resistance program that couples metabolic plasticity with mitotic fidelity. Rather than indicating that EGFRvIII alone generates all components of this resistance program, our data support a genotype field convergence model. TEFT induces a broadly inducible mitochondrial and bioenergetic stress response that activates the AMPK-PPARα-CPT1A axis, whereas EGFRvIII provides NF-κB-dependent transcriptional priming of IDO1. The convergence of these two inputs promotes CPT1A-dependent IDO1 stabilization and subsequent activation of an IDO1-kynurenine-AhR cytoskeletal protection program. This finding bridges the gap between metabolic reprogramming and the physical mechanism of action of electric fields.

The first layer of this resistance is transcriptional. While wild type EGFR activates NF-κB in a ligand-dependent manner(*9*, *47*), our data indicate that constitutively active EGFRvIII maintains persistent NF-κB/RelA signaling and increases RelA occupancy at the IDO1 promoter**.** Mechanistically, this results in increased basal IDO1 transcription in EGFRvIII-driven cells**.** Although IDO1 is classically recognized for its role in tryptophan catabolism: depleting essential amino acids to impair T-cell proliferation and generating kynurenine to support immunosuppressive T-cell states(*36*, *48*, *49*); our findings suggest that, in the context of TEFT, IDO1 also serves an intrinsic tumor-protective role beyond immune evasion. The NF-κB-driven increase in IDO1 mRNA provides the necessary substrate for subsequent CPT1A-dependent post-translational stabilization, creating a transcriptional “supply arm” that maintains an IDO1 pool capable of supporting downstream AhR-associated cytoskeletal protection.

A pivotal discovery of our work is the counter-intuitive metabolic response triggered by TEFT. We observed that TEFT induces mitochondrial and bioenergetic stress characterized by mitochondrial depolarization, mitochondrial calcium accumulation, and lipid oxidative stress**.** Instead of succumbing to this stress, tumor cells initiate an active adaptation via the AMPK–PPARα axis to upregulate CPT1A. Crucially, although we initially delineated this pathway in EGFRvIII-mutant cells, the triggering bioenergetic stress is a broadly inducible biophysical consequence of electric-field exposure rather than a unique feature of the mutation. Parental LN229 and T98G cells also exhibited mitochondrial stress, AMPKα phosphorylation, PPARα–PGC1α engagement, and CPT1A induction following TEFT exposure. This suggests that upregulation of CPT1A-mediated fatty-acid metabolism may represent a common metabolic adaptation employed by diverse GBM cells under TEFT stress, although this upstream response alone is insufficient to establish the complete EGFRvIII–IDO1 resistance cascade.

3. Risks of over-interpretation. The authors proposed a mechanistic model with multi-signaling steps. It is very likely each step is only partially correlative rather than causally required.

**Response:** We agree that coordinated changes across a multi-step pathway would

remain correlative unless the major nodes were tested using independent functional perturbations. We therefore strengthened the revised study using complementary genetic depletion, pharmacological inhibition, mutant-rescue experiments, biochemical reconstitution, and downstream functional assays.

First, two independent IDO1-targeting siRNAs increased TEFT-induced apoptosis and restored TEFT-induced G2/M accumulation in TBD0220, LN229-EGFRvIII, and T98G-EGFRvIII cells. Pharmacological inhibition of IDO1 catalytic activity with epacadostat similarly increased TEFT-induced apoptosis in established and low-passage patient-derived EGFR-driven GBM cells. These findings support a functional requirement for IDO1 rather than a bystander association. The relevant changes made in the results have been added to Figs. 1K, L; figs. S2E-J, S3A-H.

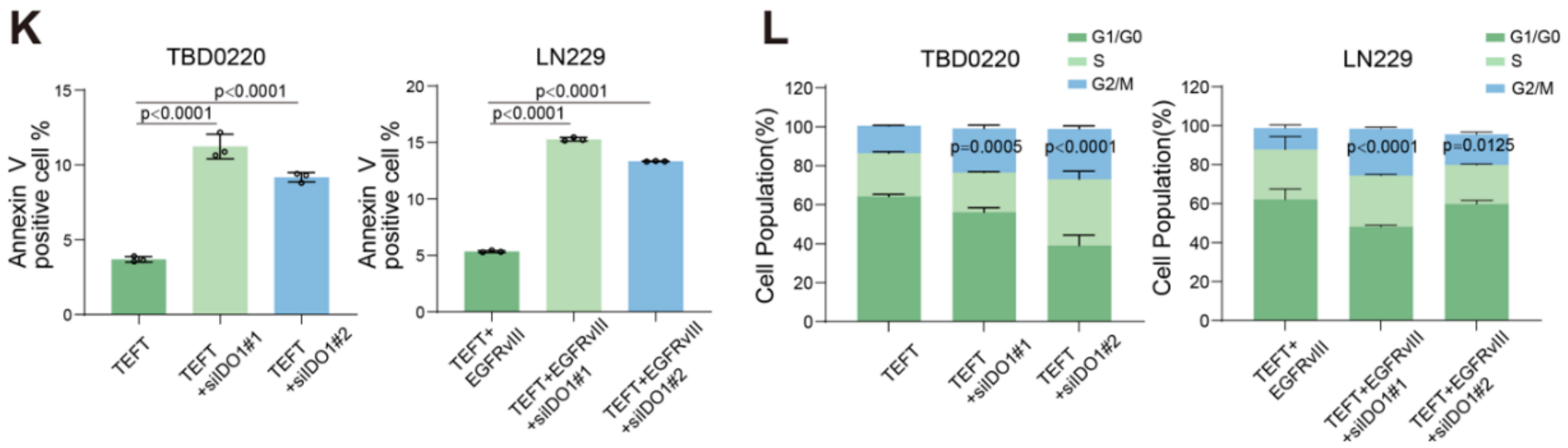


**Fig.1 (K)** Rescue effects of IDO1 knockdown (siRNA) on apoptosis in TEFT-treated EGFRvIII-driven TBD0220 and LN229 cells, **(L)** Cell cycle distribution analysis showing that IDO1 knockdown restores sensitivity to TEFT-induced mitotic perturbation (G2/M arrest) in EGFRvIII cells.

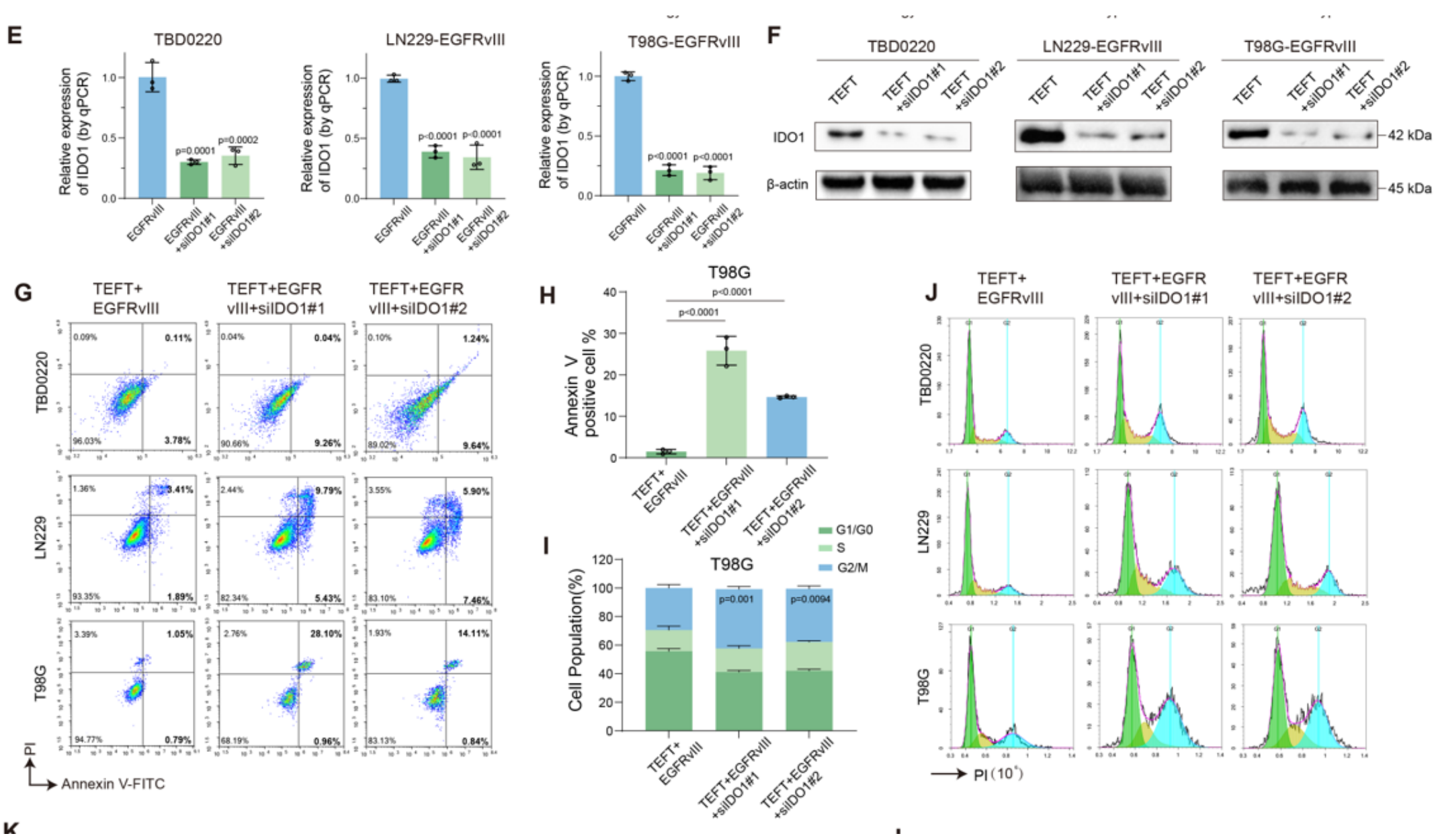


**fig. S2 (E)** RT-qPCR validation of IDO1 knockdown efficiency in TBD0220, and EGFRvIII-overexpressing LN229 and T98G cells transfected with two independent siRNAs (siIDO1#1, siIDO1#2), **(F)** Western blot validation of IDO1 knockdown efficiency at the protein level in TBD0220, LN229 and T98G cells, **(G)** Representative flow cytometry plots of Annexin V/PI staining showing that IDO1

knockdown sensitizes EGFRvIII-driven cells to TEFT-induced apoptosis, **(H)** Quantification of Annexin V-positive T98G-EGFRvIII cells after TEFT treatment with or without IDO1 knockdown, **(I)** Quantification of cell cycle distribution in T98G-EGFRvIII cells after TEFT treatment with or without IDO1 knockdown, **(J)** Representative flow cytometry histograms of cell cycle analysis showing that IDO1 knockdown restores TEFT-induced G2/M accumulation in EGFRvIII-driven cells.

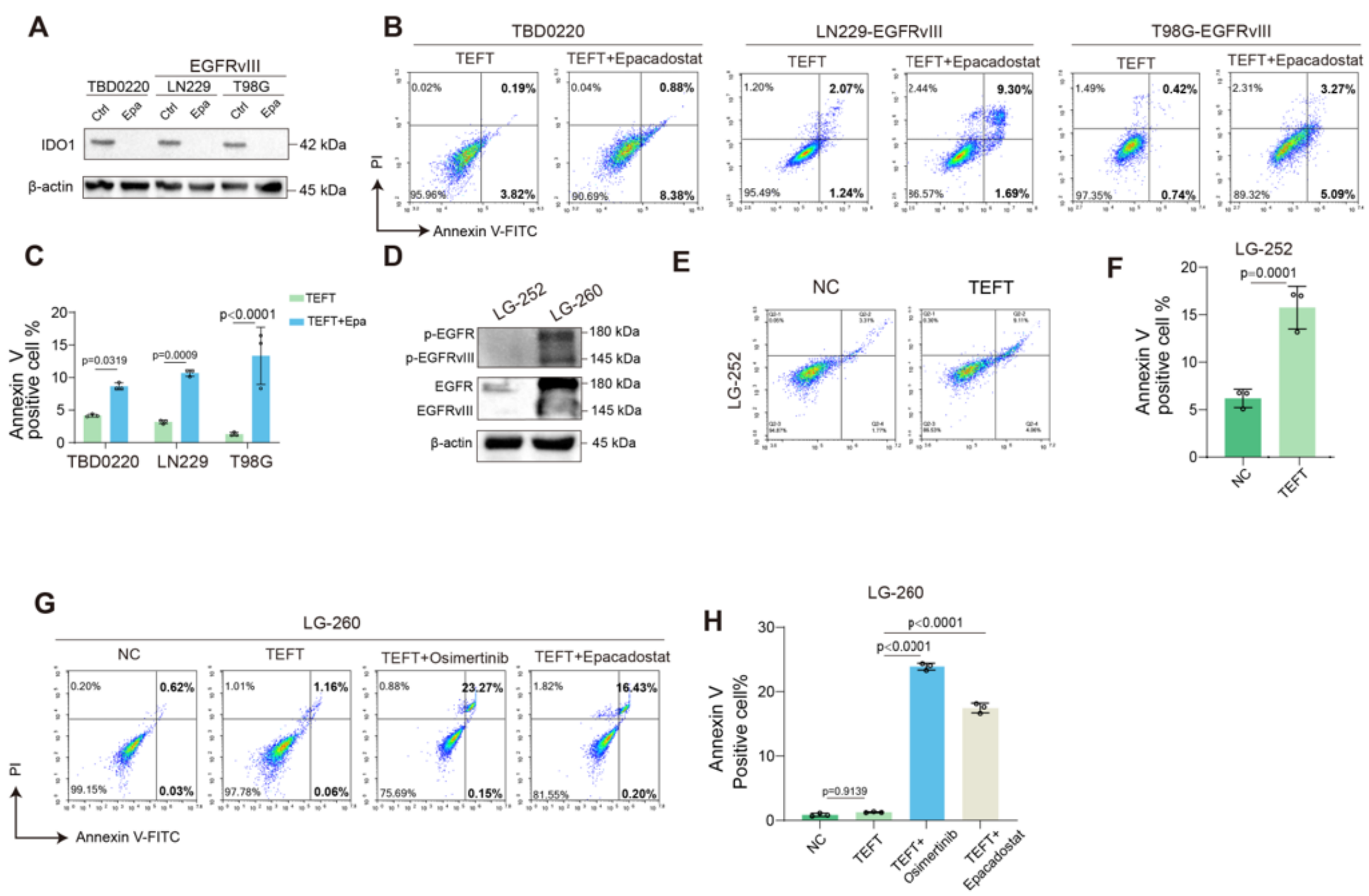

**fig. S3 (A)** Western blot analysis of IDO1 protein levels in TBD0220, LN229-EGFRvIII, and T98G-EGFRvIII cells treated with vehicle or epacadostat, **(B)** Representative Annexin V/PI flow cytometry plots showing that epacadostat increases TEFT-induced apoptosis in TBD0220, LN229-EGFRvIII, and T98G-EGFRvIII cells, **(C)** Quantification of Annexin V-positive cells from panel B, **(D)** Western blot analysis of EGFR/EGFRvIII phosphorylation and total EGFR/EGFRvIII expression in patient-derived LG-252 and LG-260 cells. β-actin served as a loading control, **(E and F)** Representative Annexin V/PI flow cytometry plots and quantification showing that patient-derived LG-252 cells are sensitive to TEFT-induced apoptosis, **(G and H)** Representative Annexin V/PI flow cytometry plots and quantification in patient-derived LG-260 cells under the indicated conditions. Osimertinib or epacadostat enhanced apoptosis under TEFT treatment.

Second, CPT1A depletion reduced IDO1 succinylation and increased IDO1 ubiquitination. Re-expression of wild-type CPT1A or the LSTase-retaining/CPTase-deficient G710E mutant restored IDO1 succinylation, whereas the catalytically defective H473A mutant did not. Cell-free assays using purified recombinant proteins further supported direct CPT1A-dependent IDO1 succinylation. The relevant changes made in the results have been added to fig. S8C-L.

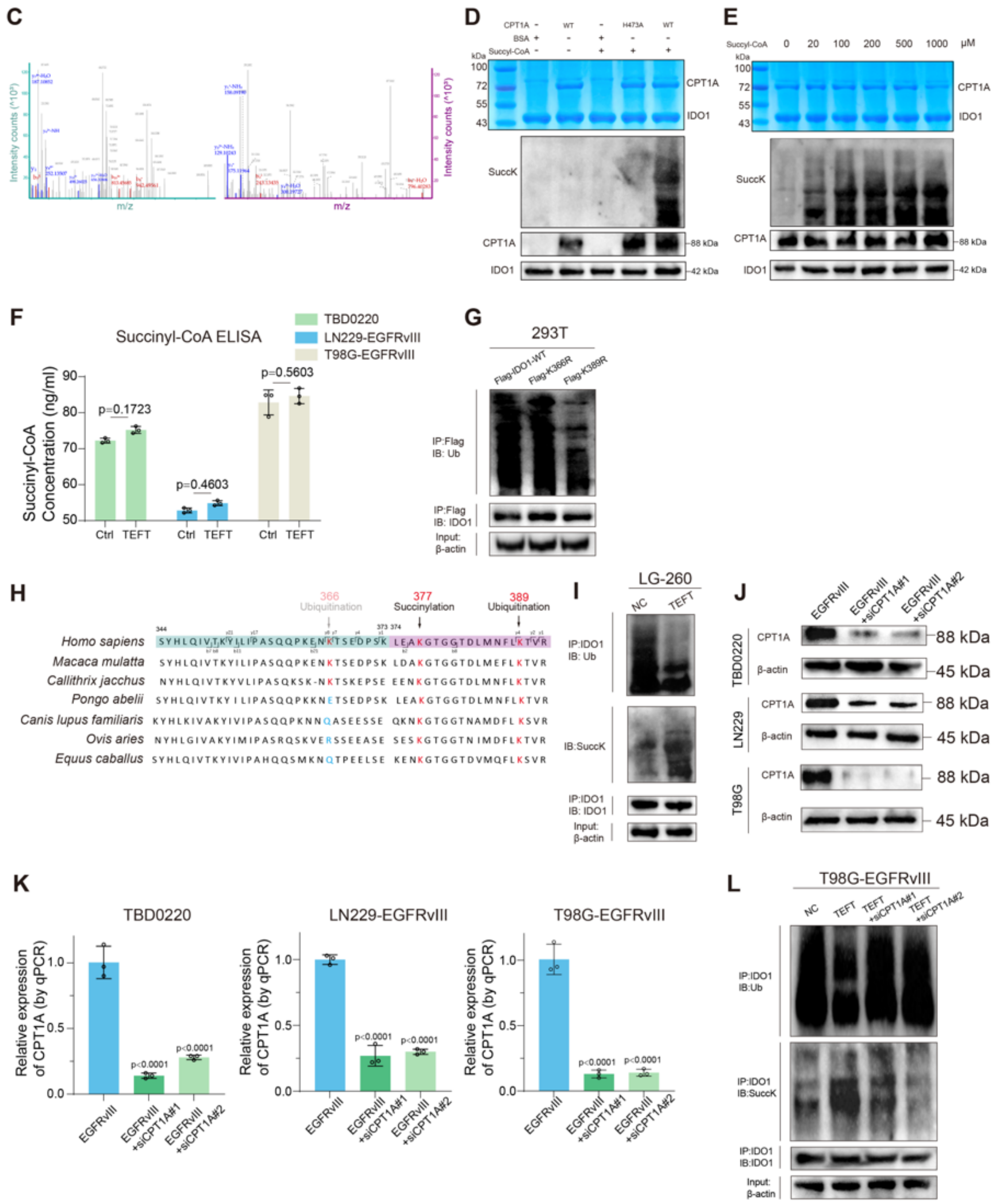


**fig. S8 (C)** Representative LC–MS/MS spectra identifying post-translational modification sites on IDO1, **(D)** In vitro recombinant protein succinylation assay using purified IDO1 and CPT1A proteins. Wild-type CPT1A directly induced IDO1 lysine succinylation in the presence of succinyl-CoA, whereas the catalytically defective H473A mutant showed reduced activity, **(E)** Dose-dependent in vitro succinylation assay showing increased IDO1 lysine succinylation with increasing succinyl-CoA concentrations in the presence of recombinant CPT1A, **(F)** Quantification of total cellular succinyl-CoA levels by ELISA in TBD0220, LN229-EGFRvIII, and T98G-EGFRvIII cells treated with or without TEFT. TEFT did not significantly deplete the total cellular succinyl-CoA pool, **(G)** Immunoprecipitation analysis of IDO1 ubiquitination in 293T cells expressing Flag-tagged IDO1-WT, IDO1-K366R, or IDO1-K389R. Mutation of K389 reduced IDO1 ubiquitination compared with wild-type IDO1, **(H)** Multiple sequence alignment of IDO1 across species showing conservation of the identified ubiquitination sites K366 and K389 and the succinylation site K377, **(I)** Immunoprecipitation analysis of endogenous IDO1 ubiquitination and succinylation in patient-derived EGFRvIII cells treated with or

without TEFT. TEFT reduced IDO1 ubiquitination and increased IDO1 succinylation, **(J and K)** Western blot (J) and RT-qPCR (K) validation of CPT1A knockdown efficiency in TBD0220, LN229-EGFRvIII, and T98G-EGFRvIII cells transfected with two independent siRNAs targeting CPT1A. β-actin served as a loading control, **(L)** Immunoprecipitation analysis of endogenous IDO1 ubiquitination and succinylation in T98G-EGFRvIII cells. TEFT promoted IDO1 succinylation and reduced IDO1 ubiquitination, whereas CPT1A knockdown attenuated this TEFT-induced post-translational switch.

Third, the downstream structural phenotype was tested directly. IDO1 depletion, epacadostat, or osimertinib disrupted the spindle-protective phenotype under TEFT. DCLK1 depletion increased spindle and nuclear abnormalities, while IDO1 depletion, ARHGEF2 depletion, epacadostat, or osimertinib reduced the enhanced tubulin-polymerizing activity associated with the resistant state. The relevant changes made in the results have been added to Fig. 5E, L, S10D-J, and S11A-I.

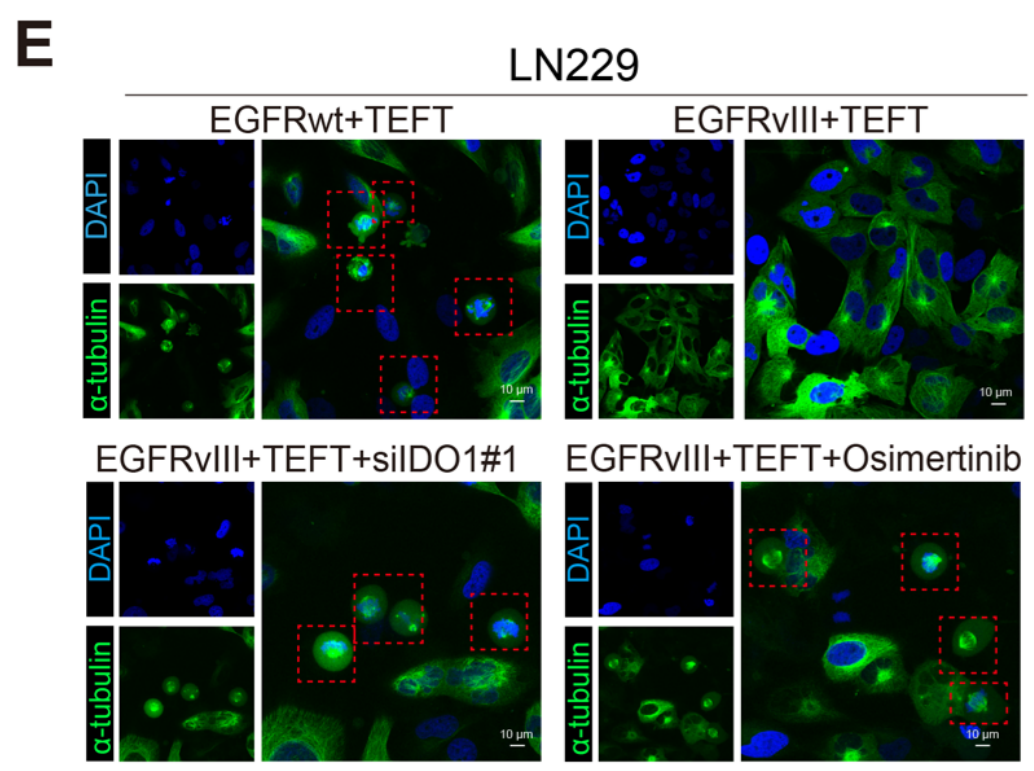


**Fig. 5 (E)** Representative immunofluorescence images of mitotic spindles (α-tubulin, green) and chromosomes (DAPI, blue) in LN229 cells. TEFT causes severe spindle disruption and multipolar mitosis in wild-type cells, whereas EGFRvIII cells maintain bipolar spindles. This protection is lost upon IDO1 knockdown or osimertinib treatment.

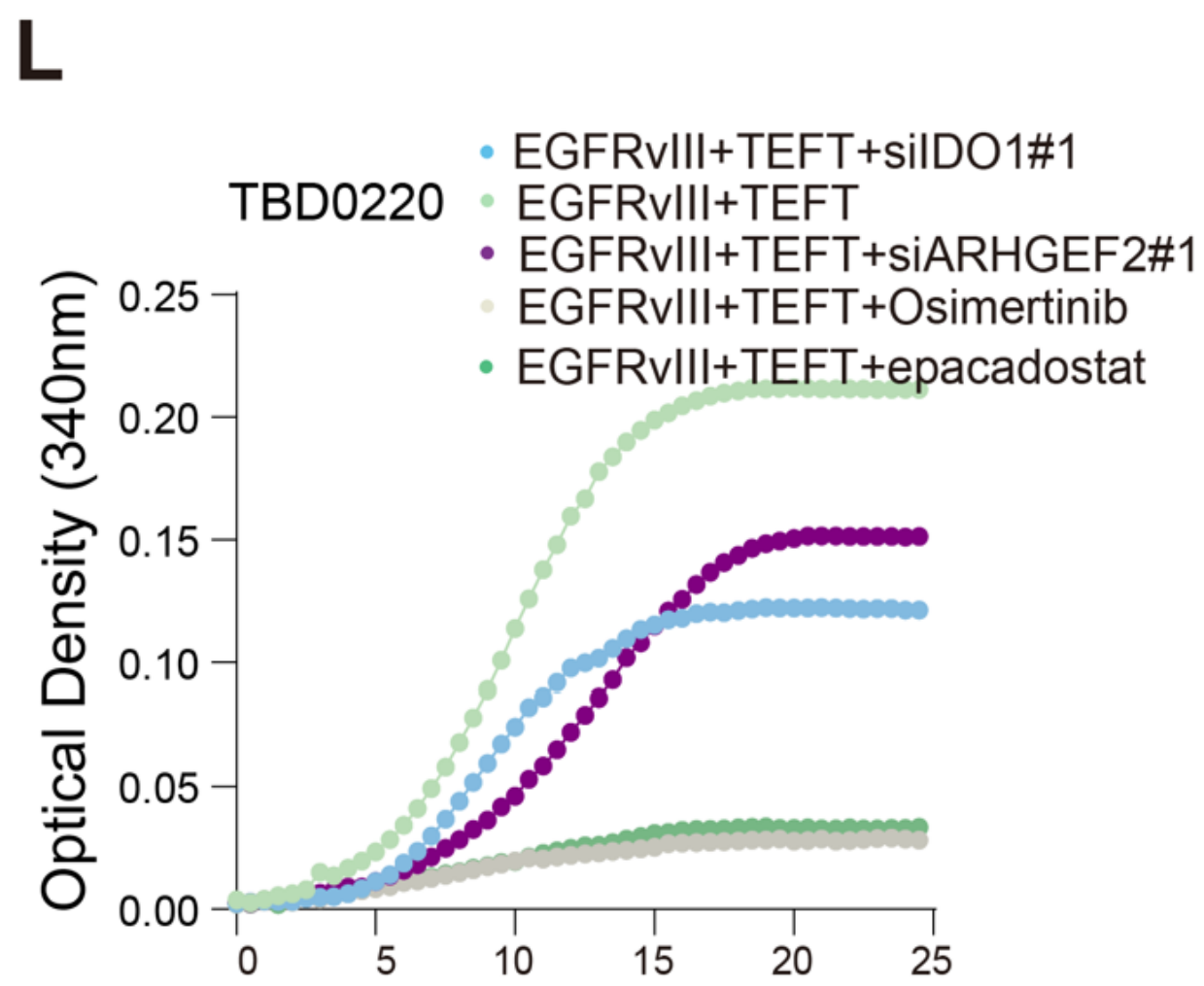


**Fig. 5 (L)** In vitro tubulin polymerization assay in TBD0220 cells under the indicated conditions. IDO1

knockdown, ARHGEF2 knockdown, osimertinib, or epacadostat reduced the microtubule-polymerizing activity associated with EGFRvIII-mediated TEFT resistance.

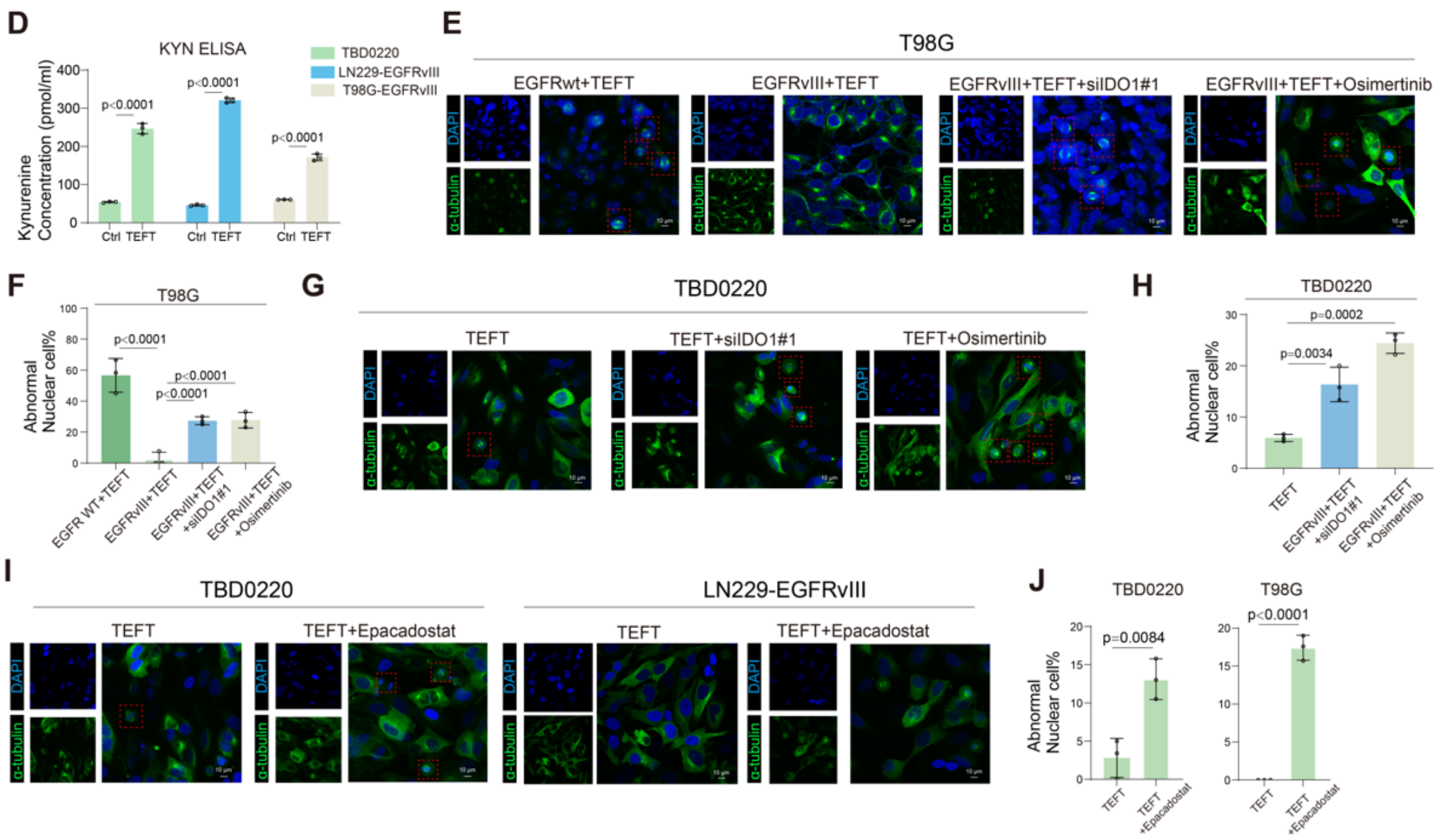


**fig. S10** (**D**) Kynurenine ELISA showing increased extracellular kynurenine levels in the culture supernatants of TBD0220, LN229-EGFRvIII, and T98G-EGFRvIII cells after TEFT treatment, (**E**) Representative immunofluorescence images of α-tubulin staining in T98G cells under the indicated conditions. TEFT induced spindle and nuclear abnormalities in EGFR wild-type cells, whereas EGFRvIII cells maintained a more preserved microtubule architecture under TEFT. This protective phenotype was disrupted by IDO1 knockdown or osimertinib treatment. DAPI, nuclei; α-tubulin, green. Scale bars, 10 μm, (**F**) Quantification of cells with abnormal nuclear morphology in T98G cells under the indicated conditions, (**G**) Representative immunofluorescence images of α-tubulin staining in TBD0220 cells treated with TEFT, TEFT plus IDO1 knockdown, or TEFT plus osimertinib. IDO1 knockdown or osimertinib disrupted the TEFT-resistant spindle-protective phenotype. DAPI, nuclei; α-tubulin, green. Scale bars, 10 μm, (**H**) Quantification of cells with abnormal nuclear morphology in TBD0220 cells under the indicated conditions, (**I**) Representative immunofluorescence images of α-tubulin staining in TBD0220 and LN229-EGFRvIII cells treated with TEFT alone or TEFT plus epacadostat. Pharmacological IDO1 inhibition disrupted the spindle-protective phenotype under TEFT exposure. DAPI, nuclei; α-tubulin, green. Scale bars, 10 μm, (**J**) Quantification of cells with abnormal nuclear morphology in TBD0220 and T98G cells after TEFT treatment with or without epacadostat.

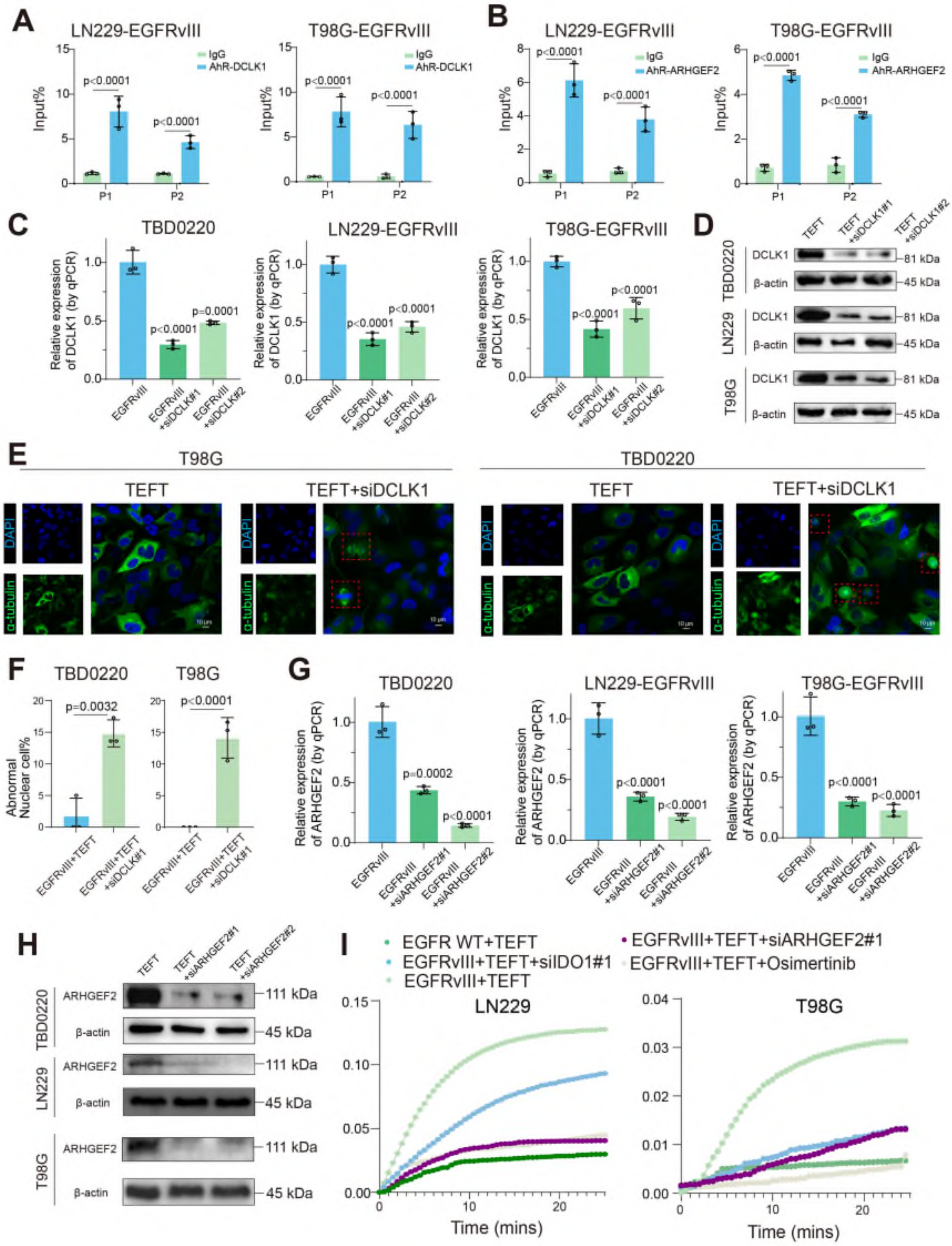

**fig. S11 (A)** ChIP-qPCR analysis of AhR occupancy at the predicted *DCLK1* promoter regions (P1 and P2) in LN229 and T98G EGFRvIII cells, **(B)** ChIP-qPCR analysis of AhR occupancy at the *ARHGEF2* promoter regions (P1 and P2), **(C)** RT-qPCR validation of DCLK1 knockdown efficiency in TBD0220, LN229-EGFRvIII, and T98G-EGFRvIII cells transfected with two independent siRNAs targeting DCLK1, **(D)** Western blot validation of DCLK1 knockdown efficiency in TBD0220, LN229-EGFRvIII, and T98G-EGFRvIII cells, **(E)** Representative immunofluorescence images of α-tubulin staining in T98G-EGFRvIII and TBD0220 cells treated with TEFT with or without DCLK1 knockdown. DCLK1 depletion increased TEFT-induced spindle and nuclear abnormalities. DAPI, nuclei; α-tubulin, green.

Scale bars, 10 µm, **(F)** Quantification of cells with abnormal nuclear morphology in TBD0220 and T98G-EGFRvIII cells treated with TEFT with or without DCLK1 knockdown, **(G)** RT-qPCR validation of ARHGEF2 knockdown efficiency in TBD0220, LN229-EGFRvIII, and T98G-EGFRvIII cells transfected with two independent siRNAs targeting ARHGEF2, **(H)** Western blot validation of ARHGEF2 knockdown efficiency in TBD0220, LN229-EGFRvIII, and T98G-EGFRvIII cell, **(I)** In vitro tubulin polymerization assay in LN229 and T98G cells under the indicated conditions. IDO1 knockdown, ARHGEF2 knockdown, or osimertinib reduced the microtubule-polymerizing activity associated with EGFRvIII-mediated TEFT resistance.

These complementary perturbations support the causal involvement of the major nodes in the proposed pathway rather than a model based only on coordinated expression changes. We have nevertheless revised the manuscript to avoid implying that every molecular event is absolutely or exclusively required in every GBM context. The revised conclusions refer to a functionally supported resistance program in EGFRvIII-driven and IDO1-primed models.

**Other Changes in the revised manuscript are provided below.**

**Results, lines 130-175, 295-315, and 349-388;**

To determine whether IDO1 is a functional mediator of EGFRvIII-driven resistance rather than a bystander, we performed loss-of-function assays. We first validated the knockdown efficiency of two independent small interfering RNAs in TBD0220, LN229-EGFRvIII, and T98G-EGFRvIII cells, confirming robust depletion at both the mRNA and protein levels (fig. S2E, F). We then assessed the impact of IDO1 silencing on cell survival under electric field exposure. Flow cytometric analysis of apoptosis showed that while EGFRvIII expression reduced TEFT-induced cell death, silencing IDO1 attenuated this protective phenotype, resulting in a marked increase in apoptosis following TEFT treatment (Fig. 1K and fig. S2G, H). Consistently, cell-cycle analysis revealed that IDO1 knockdown resensitized EGFRvIII-driven cells to TEFT-induced mitotic perturbation. Whereas EGFRvIII-driven cells displayed less pronounced TEFT-induced G2/M accumulation, IDO1-depleted cells exhibited restored G2/M accumulation and mitotic arrest (Fig. 1L and fig. S2I-L). These data support a functional requirement for IDO1 in sustaining the TEFT-resistant phenotype in EGFRvIII-driven GBM cells.

To complement these genetic loss-of-function data, we next tested whether pharmacological inhibition of IDO1 catalytic activity could similarly restore TEFT sensitivity. Epacadostat did not appreciably alter total IDO1 protein abundance (fig. S3A), but significantly increased TEFT-induced apoptosis in TBD0220, LN229-EGFRvIII, and T98G-EGFRvIII cells (fig. S3B, C). We then extended this analysis to independent low-passage patient-derived GBM cultures. Molecular characterization showed that LG-260 exhibited EGFR amplification and EGFRvIII expression, whereas LG-252 lacked the corresponding EGFRvIII-driven signaling profile (fig. S3D). LG-252 cells remained sensitive to TEFT-induced apoptosis (fig. S3E, F). In contrast, LG-260 cells showed a limited apoptotic response to TEFT alone, whereas either osimertinib or epacadostat markedly increased TEFT-induced apoptosis (fig. S3G, H). Thus, direct pharmacological inhibition of IDO1 enzymatic activity phenocopied the resensitizing effect of upstream EGFR blockade in an independent patient-derived EGFR-driven

model. Together with the genetic depletion experiments, these findings establish IDO1 as a functional downstream effector of EGFR-driven TEFT resistance.

**EGFRvIII Up-regulates IDO1 Transcription in an NF-κB-dependent Manner**

To precisely dissect the molecular basis of EGFRvIII-driven IDO1 induction, we first focused on the upstream transcriptional regulatory mechanisms. KEGG pathway analysis revealed enrichment of the NF-κB signaling pathway in EGFRvIII-mutant GBM cells compared with their wild-type counterparts (Fig. 2A). Consistent with this transcriptional signature, ATAC-seq footprinting analysis showed increased accessibility around NFKB1 and RELA binding motifs in EGFRvIII-expressing cells (Fig. 2B). Western blot analysis further supported activation of this pathway, showing elevated phosphorylation of NF-κB p65 together with IDO1 accumulation in TBD0220 and LN229-EGFRvIII cells (Fig. 2C). Additional immunoblotting in an independent EGFRvIII-positive primary model and T98G cells further showed that EGFRvIII expression was associated with enhanced NF-κB phosphorylation and IDO1 abundance, with further IDO1 accumulation following TEFT exposure (fig. S3I). GSEA analysis further revealed that the NF-κB pathway was enriched in EGFRvIII-mutant GBM cells under TEFT treatment (Fig. 2D). Focusing specifically on the IDO1 locus, we identified two predicted NF-κB-binding sequences, P1 and P2, within the IDO1 promoter region. ChIP-qPCR assays showed enhanced RelA occupancy at these predicted IDO1 promoter regions in TBD0220 and LN229 cells (Fig. 2E), with additional validation in T98G-EGFRvIII cells (fig. S3J). Functionally, pharmacological inhibition of NF-κB signaling with JSH-23, or upstream blockade of EGFR signaling with osimertinib, reduced IDO1 mRNA levels (Fig. 2F and fig. S3K, L). (lines 130-175)

To obtain direct biochemical evidence for CPT1A-mediated IDO1 succinylation, we performed cell-free assays using purified recombinant proteins. CPT1A-WT promoted IDO1 succinylation in the presence of succinyl-CoA, whereas the H473A mutant showed reduced activity toward IDO1 (fig. S8D). IDO1 succinylation also increased with increasing succinyl-CoA concentrations in the presence of CPT1A (fig. S8E). Moreover, TEFT did not significantly increase total cellular succinyl-CoA concentrations in TBD0220, LN229-EGFRvIII, or T98G-EGFRvIII cells, arguing against a mechanism driven solely by a global increase in succinyl-CoA availability or nonspecific non-enzymatic succinylation (fig. S8F).

To determine the precise mechanism of stabilization, we generated IDO1 lysine mutants. Cycloheximide (CHX) chase assays revealed that the IDO1-K389R mutant exhibited a significantly extended half-life compared with wild-type IDO1, implicating K389 as an important ubiquitination-associated degron that contributes to IDO1 turnover (Fig. 4G). Consistent with this, the K389R mutation reduced IDO1 ubiquitination (fig. S8G). Evolutionary analysis showed that the K366, K377, and K389 sites are highly conserved across species, supporting the potential functional importance of this regulatory region (fig. S8H). Finally, we verified this axis in the context of TEFT. In TBD0220 and LN229-EGFRvIII cells, TEFT treatment increased IDO1 succinylation and reduced its ubiquitination; importantly, CPT1A knockdown attenuated this TEFT-induced post-translational switch (Fig. 4H). Consistent results were obtained in T98G-EGFRvIII and patient-derived EGFR-driven cells (fig. S8I-L). Thus, TEFT promotes a CPT1A-dependent shift in IDO1 post-translational regulation from ubiquitination toward succinylation, thereby supporting IDO1 stabilization. (lines 295-315)

Phenotypic analysis demonstrated that while TEFT typically induces mitotic defects, characterized by spindle misalignment and abnormal nuclear morphology in control cells, EGFRvIII expression conferred a structural protective effect, maintaining bipolar spindle integrity and reducing the percentage of abnormal nuclei. This protection was impaired upon IDO1 silencing or inhibition with osimertinib**,** supporting a functional role for IDO1 signaling in TEFT resistance (Fig. 5E, F). Similar spindle-disruptive effects of IDO1 knockdown, osimertinib, or the IDO1 inhibitor epacadostat were observed in TBD0220 and LN229-EGFRvIII cells (fig. S10E-J). Together, these findings support an IDO1-kynurenine-AhR cascade that contributes to mitotic and microtubule protection during TEFT resistance.

To identify the downstream structural effectors, we employed a multi-omics intersection strategy. Specifically, we intersected public AhR cistrome data from ChIP-Atlas(*37*), ATAC-seq data, and our RNA-seq data to screen for genes regulating spindle assembly, which led to the identification of DCLK1 (doublecortin-like kinase 1) (Fig. 5G). To validate the direct regulatory link, we performed ChIP-qPCR assays, which detected AhR occupancy at the predicted DCLK1 and ARHGEF2 promoter regions, supporting direct AhR-dependent transcriptional regulation of DCLK1 **(**fig. S11A). We confirmed that TEFT treatment upregulated DCLK1 protein levels in EGFRvIII-expressing LN229 and T98G cells (Fig. 5H). Functionally, DCLK1 contributed to the observed structural protection(*40*, *41*); silencing DCLK1 in EGFRvIII cells disrupted spindle organization and increased abnormal nuclear morphology (Fig. 5I, J). These effects were further validated in TBD0220 and T98G-EGFRvIII cells (fig. S11C-F). Thus, DCLK1 functions as a direct AhR-regulated mitotic effector that contributes to cytoskeletal protection under TEFT stress.

To further dissect the mechanism underlying microtubule stability under physical stress, we sought to identify effectors that directly associate with the cytoskeleton. We performed immunoprecipitation coupled with mass spectrometry (IP-MS) using anti-tubulin antibodies in TEFT-treated EGFRvIII cells to isolate the tubulin interactome. By intersecting these proteomic data with our genomic ATAC-seq and public AhR target datasets, we identified ARHGEF2 (GEF-H1) as a microtubule-associated effector (Fig. 5K) (*17*, *42*-*46*). Similar to DCLK1, ChIP-qPCR analysis demonstrated increased AhR occupancy at predicted motifs within the ARHGEF2 promoter in LN229-EGFRvIII and T98G-EGFRvIII cells after TEFT treatment, supporting ARHGEF2 as a direct transcriptional target of AhR (fig. S11B). Efficient ARHGEF2 knockdown was confirmed at both the mRNA and protein levels (fig. S11G, H). To validate its functional role, we performed in vitro tubulin polymerization assays. Lysates from TEFT-treated EGFRvIII cells exhibited a capacity to promote microtubule polymerization. Importantly, this stabilizing effect was attenuated by IDO1 knockdown, ARHGEF2 knockdown, osimertinib, or epacadostat in TBD0220 cells (Fig. 5L), with consistent effects observed in LN229-EGFRvIII and T98G-EGFRvIII cells (fig. S11I). These complementary perturbations place IDO1 activity, EGFR signaling, and AhR-regulated ARHGEF2 within the same functional cytoskeletal protection program rather than representing independent correlative events. (lines 349-388)

**Discussion, lines 496-522;**

Our identification of the CPT1A-IDO1 stabilization axis provides an additional framework for considering IDO1 enzymatic inhibitors, including epacadostat(*56*-*58*). While previous strategies have focused on blocking the catalytic pocket(*59*, *60*) , our epacadostat experiments indicate that the enzymatic output of IDO1 remains functionally important: pharmacological inhibition increased TEFT-

induced apoptosis and impaired microtubule protection, phenocopying key aspects of genetic IDO1 depletion and osimertinib-mediated re-sensitization. At the same time, our findings show that TEFT resistance is also regulated at the level of IDO1 protein abundance through CPT1A-dependent succinylation and stabilization. Therefore, strategies that reduce IDO1 transcription or stability-such as EGFR/NF-κB inhibition or targeting the CPT1A LSTase-related stabilization mechanism, may complement catalytic IDO1 inhibition by limiting both kynurenine production and protein-abundance-dependent downstream signaling.

Downstream of IDO1, our study uncovers a novel function for the Aryl Hydrocarbon Receptor (AhR). Typically viewed as a sensor for environmental toxins(*61*), our data support a role for AhR in linking tryptophan metabolism to cytoskeletal adaptation in EGFRvIII-driven GBM under TEFT stress. Increased kynurenine production, AhR nuclear translocation, and induction of canonical AhR-responsive genes collectively support activation of this pathway. AhR activation is associated with increased transcription of DCLK1 and ARHGEF2, with ChIP-qPCR supporting AhR occupancy at their predicted promoter regions. DCLK1 is a microtubule-associated kinase involved in bipolar spindle formation(*40, 41*), while ARHGEF2/GEF-H1 regulates RhoA-dependent microtubule dynamics(*17, 46*). Promoter ChIP-qPCR and functional perturbation experiments support DCLK1 and ARHGEF2 as direct AhR-regulated cytoskeletal effectors in this setting. In the context of TEFT, which physically disrupts dipole alignment and spindle assembly, upregulation of these factors supports spindle organization and microtubule polymerization, thereby helping tumor cells tolerate electric-field-induced mitotic stress**.** This finding links metabolic inputs, particularly the tryptophan metabolite kynurenine, to physical structural adaptations of the cytoskeleton and provides a mechanistic explanation for how tumor cells can partially buffer the anti-mitotic effects of physical therapy.

4. Lack of stronger biochemical validation of the non-canonical CPT1a succinyltransferase activity. CPT1a is a classical rate-limiting enzyme in fatty acid oxidation. Additional evidence is needed to rule out indirect succinylation through altered succinyl-CoA pools, other known succinyltransferases, and non-enzymatic succinylation under metabolic stress.

**Response:** We agree that changes in cellular IDO1 succinylation alone would not be sufficient to attribute this modification directly to the non-canonical LSTase-related activity of CPT1A. We therefore added complementary genetic, biochemical, and metabolite analyses.

First, CPT1A mutant-rescue experiments were used to separate its LSTase-related activity from its canonical CPTase function. CPT1A depletion reduced IDO1 succinylation and increased IDO1 ubiquitination. Re-expression of wild-type CPT1A restored this phenotype. Importantly, the CPTase-deficient but LSTase-retaining G710E mutant also restored IDO1 succinylation, whereas the catalytically defective H473A mutant did not. These findings support dependence on CPT1A LSTase-related activity rather than an indirect consequence of its canonical fatty-acid transport function. The relevant changes made in the results have been added to Fig. 4F-H.

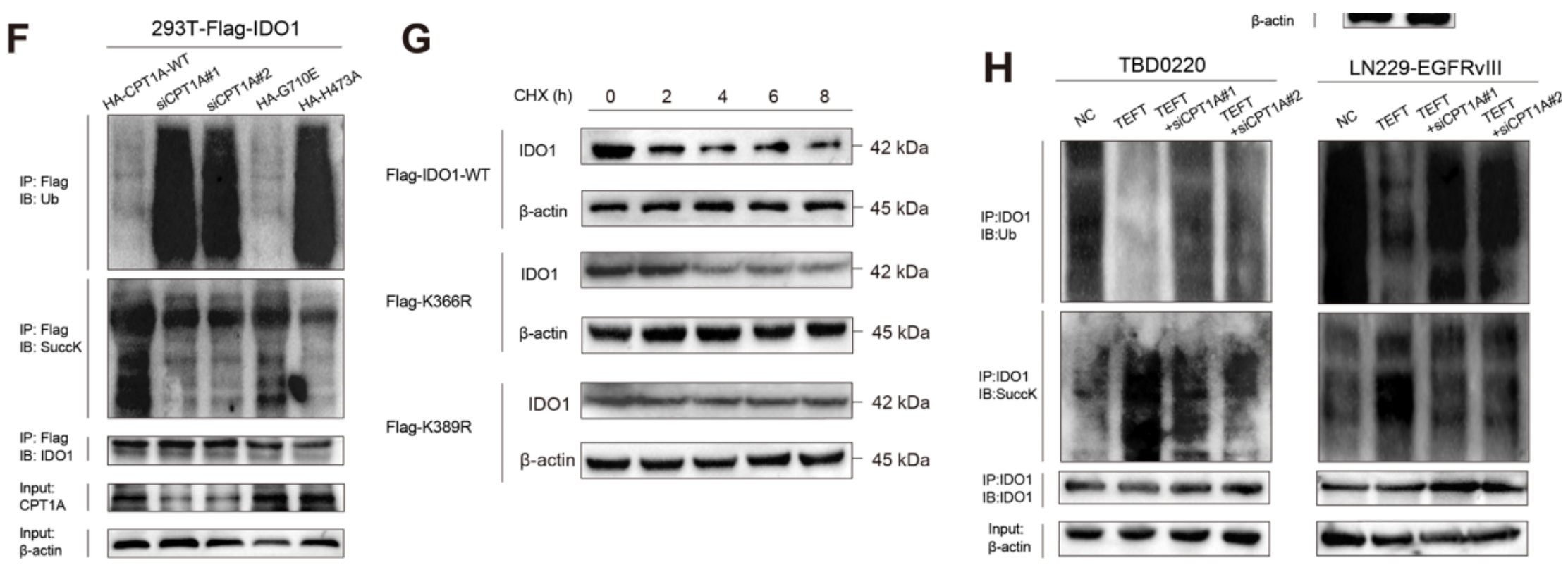


**Fig. 4 (F)** Rescue experiment in CPT1A-knockdown cells. Re-expression of wild-type (WT) CPT1A or the LSTase-active mutant (G710E) restores IDO1 succinylation and stability, whereas the LSTase-dead mutant (H473A) fails to do so, **(G)** Cycloheximide (CHX) chase assay in 293T cells transfected with IDO1-WT or mutants (K366R, K389R). The K389R mutant exhibits a significantly extended half-life, mimicking the stabilized state, **(H)** IP analysis of endogenous IDO1 ubiquitination and succinylation in EGFRvIII-GBM cells. TEFT promotes IDO1 succinylation and reduces ubiquitination; this effect is reversed by CPT1A knockdown.

Second, we performed cell-free assays using purified recombinant proteins. Recombinant CPT1A-WT promoted succinylation of purified IDO1 in the presence of succinyl-CoA, whereas the H473A mutant showed reduced activity. IDO1 succinylation also increased with increasing succinyl-CoA concentrations in the presence of CPT1A. Because this reconstituted system did not contain other cellular enzymes, it supports the biochemical capacity of CPT1A to directly promote IDO1 succinylation. The relevant changes made in the results have been added to fig. S8D, E.

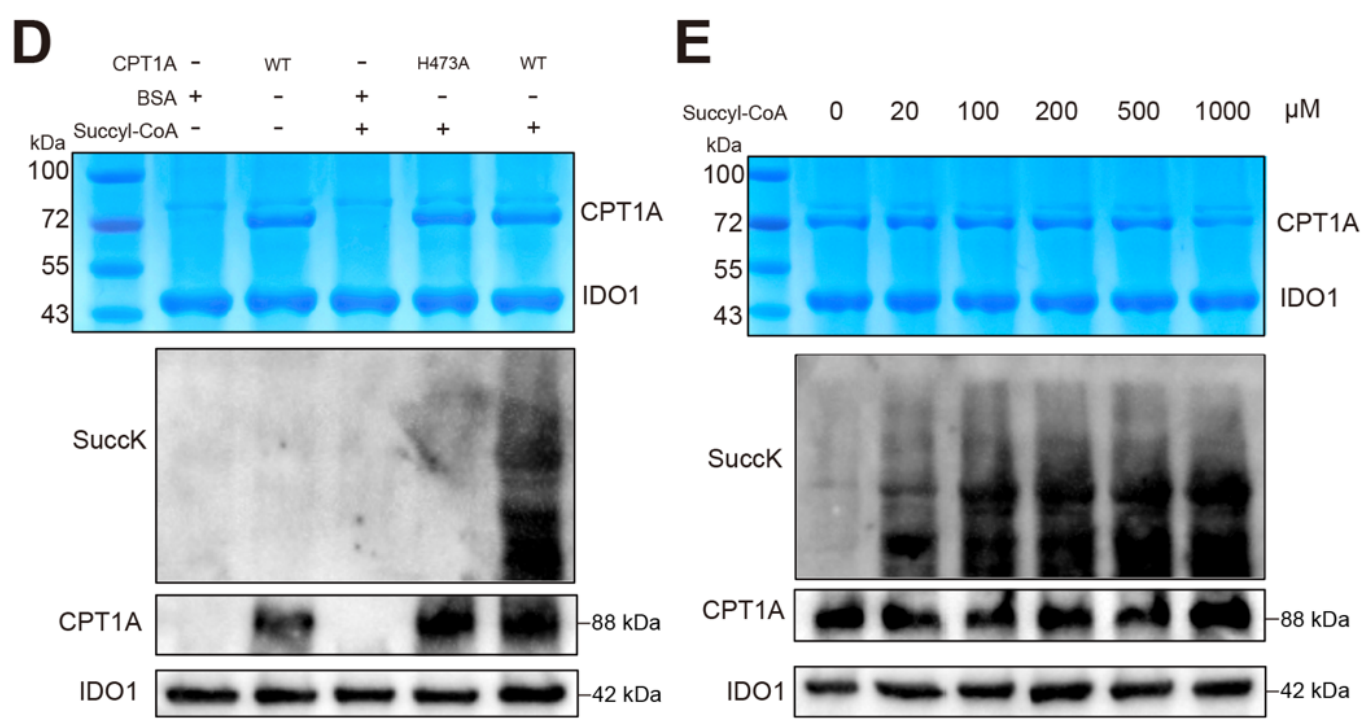


**fig. S8 (D)** In vitro recombinant protein succinylation assay using purified IDO1 and CPT1A proteins. Wild-type CPT1A directly induced IDO1 lysine succinylation in the presence of succinyl-CoA, whereas the catalytically defective H473A mutant showed reduced activity, **(E)** Dose-dependent in vitro succinylation assay showing increased IDO1 lysine succinylation with increasing succinyl-CoA concentrations in the presence of recombinant CPT1A, **(F)** Quantification of total cellular succinyl-CoA levels by ELISA in TBD0220, LN229-EGFRvIII, and T98G-EGFRvIII cells treated with or without TEFT. TEFT did not significantly deplete the total cellular succinyl-CoA pool.

Third, TEFT did not significantly increase total cellular succinyl-CoA concentrations in TBD0220, LN229-EGFRvIII, or T98G-EGFRvIII cells. This finding argues against a mechanism driven solely by a global increase in substrate availability or nonspecific non-enzymatic succinylation. The relevant changes made in the results have been added to fig. S8F.

F

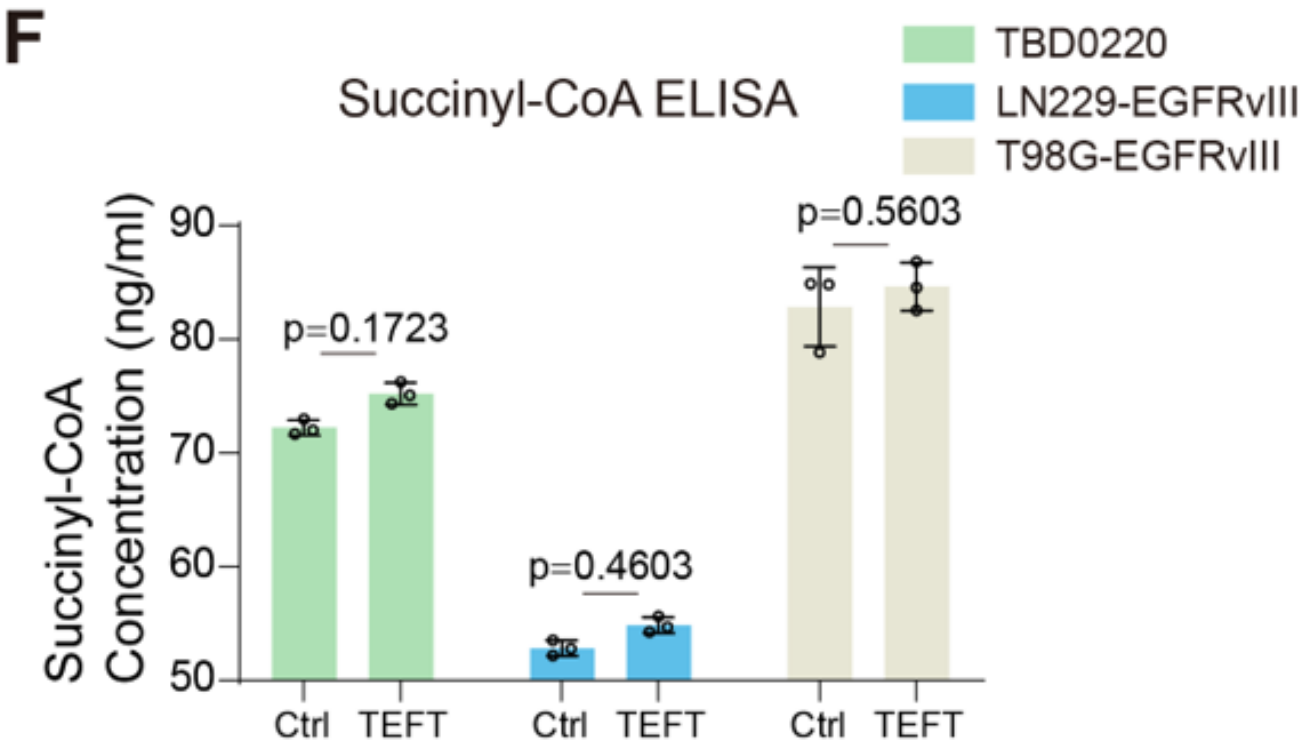


**fig. S8 (F)** Quantification of total cellular succinyl-CoA levels by ELISA in TBD0220, LN229-EGFRvIII, and T98G-EGFRvIII cells treated with or without TEFT. TEFT did not significantly deplete the total cellular succinyl-CoA pool.

Together, the mutant dependency, purified-protein reconstitution, and metabolite measurements strongly support CPT1A-dependent IDO1 succinylation through non-canonical LSTase-related activity. We do not claim that every context-dependent contribution from other succinylation-related enzymes or local metabolite microdomains has been excluded. The revised Discussion explicitly notes that the precise submitochondrial accessibility of the succinyl donor remains unresolved and will require direct submitochondrial metabolite tracing.

**Other Changes in the revised manuscript are provided below.**

**Results, lines 278–315;**

CPT1A possesses carnitine palmitoyl transferase (CPTase) activity and has also been reported to exhibit non-canonical lysine succinyltransferase-related (LSTase) activity(*31, 32*). We next investigated whether CPT1A exerts a post-translational modification on IDO1. Given CPT1A's reported moonlighting function as a lysine succinyltransferase, we performed LC-MS/MS analysis on IDO1. This identified lysine 377 (K377) as a candidate succinylation site and lysine 366/389 (K366/K389) as ubiquitination-associated sites (Fig. 4D and fig. S8C). Functional validation demonstrated that CPT1A overexpression increased IDO1 succinylation (Fig. 4E). Conversely, CPT1A depletion reduced IDO1 succinylation and increased IDO1 ubiquitination, whereas re-expression of wild-type CPT1A restored succinylation and reduced ubiquitination (Fig. 4F). To distinguish this effect of the LSTase-related activity of CPT1A from its canonical carnitine palmitoyl transferase (CPTase) function(*33, 34*), we utilized specific point mutants: CPT1A-G710E, which retains LSTase activity but lacks CPTase activity, and CPT1A-H473A, a catalytically inactive mutant. In CPT1A-depleted cells,

re-expression of wild-type CPT1A or the G710E mutant restored IDO1 succinylation and reduced IDO1 ubiquitination, whereas the H473A mutant failed to do so (Fig. 4F). These mutant-rescue data support the requirement of CPT1A LSTase-related activity, rather than classical CPTase activity alone, for the post-translational regulation of IDO1.

To obtain direct biochemical evidence for CPT1A-mediated IDO1 succinylation, we performed cell-free assays using purified recombinant proteins. CPT1A-WT promoted IDO1 succinylation in the presence of succinyl-CoA, whereas the H473A mutant showed reduced activity toward IDO1 (fig. S8D). IDO1 succinylation also increased with increasing succinyl-CoA concentrations in the presence of CPT1A (fig. S8E). Moreover, TEFT did not significantly increase total cellular succinyl-CoA concentrations in TBD0220, LN229-EGFRvIII, or T98G-EGFRvIII cells, arguing against a mechanism driven solely by a global increase in succinyl-CoA availability or nonspecific non-enzymatic succinylation (fig. S8F).

To determine the precise mechanism of stabilization, we generated IDO1 lysine mutants. Cycloheximide (CHX) chase assays revealed that the IDO1-K389R mutant exhibited a significantly extended half-life compared with wild-type IDO1, implicating K389 as an important ubiquitination-associated degron that contributes to IDO1 turnover (Fig. 4G). Consistent with this, the K389R mutation reduced IDO1 ubiquitination (fig. S8G). Evolutionary analysis showed that the K366, K377, and K389 sites are highly conserved across species, supporting the potential functional importance of this regulatory region (fig. S8H). Finally, we verified this axis in the context of TEFT. In TBD0220 and LN229-EGFRvIII cells, TEFT treatment increased IDO1 succinylation and reduced its ubiquitination; importantly, CPT1A knockdown attenuated this TEFT-induced post-translational switch (Fig. 4H). Consistent results were obtained in T98G-EGFRvIII and patient-derived EGFR-driven cells (fig. S8I-L). Thus, TEFT promotes a CPT1A-dependent shift in IDO1 post-translational regulation from ubiquitination toward succinylation, thereby supporting IDO1 stabilization.

### Discussion, lines 480–495;

CPT1A is an established outer mitochondrial membrane-resident enzyme, whereas succinyl-CoA is primarily generated within the mitochondrial matrix. Although TEFT did not globally increase total cellular succinyl-CoA, the metabolite pool was not depleted, indicating that donor availability was maintained. We therefore propose a CPT1A-associated mitochondrial microdomain model in which local metabolic organization may permit access to succinyl-CoA or related acyl-donor availability at the outer mitochondrial membrane interface(*52, 53*). This topological model will require direct submitochondrial metabolite tracing. NAC increased TEFT-induced apoptosis and attenuated CPT1A accumulation, suggesting that redox stress contributes to the upstream metabolic adaptation(*54*, *55*). Crucially, upregulated CPT1A also transcends its canonical metabolic role. Through its LSTase-related activity, CPT1A promotes IDO1 succinylation at K377, which functionally antagonizes ubiquitination at the adjacent K389 degron and limits TRIM21-mediated proteasomal degradation. This reveals a dual-input mechanism: TEFT-induced metabolic stress upregulates CPT1A, whereas EGFRvIII supplies the transcriptionally primed IDO1 substrate. When these inputs converge, the metabolic adaptation induced by TEFT stabilizes the protein that supports downstream resistance, thereby creating a compensatory mechanism that limits therapeutic efficacy.

**Limitations, lines 559–563;**

Third, although we mapped the CPT1A LSTase-related activity, the precise source and submitochondrial accessibility of the succinyl-CoA donor require further biochemical dissection. Although total cellular succinyl-CoA was not depleted under TEFT, direct submitochondrial metabolite tracing will be necessary to test the proposed CPT1A-associated outer mitochondrial membrane microdomain model.

5. The data on AhR regulation in the resistant progresses is not convincing.

**Response:** We agree that the original evidence for AhR regulation relied too heavily on pathway analysis and required stronger biochemical, spatial, and transcriptional validation. We therefore expanded the analysis at four levels.

First, we directly measured kynurenine, the IDO1-derived endogenous ligand for AhR. TEFT increased extracellular kynurenine concentrations in TBD0220, LN229-EGFRvIII, and T98G-EGFRvIII cells, demonstrating that TEFT-stabilized IDO1 retained functional enzymatic output. The relevant changes made in the results have been added to fig. S10D.

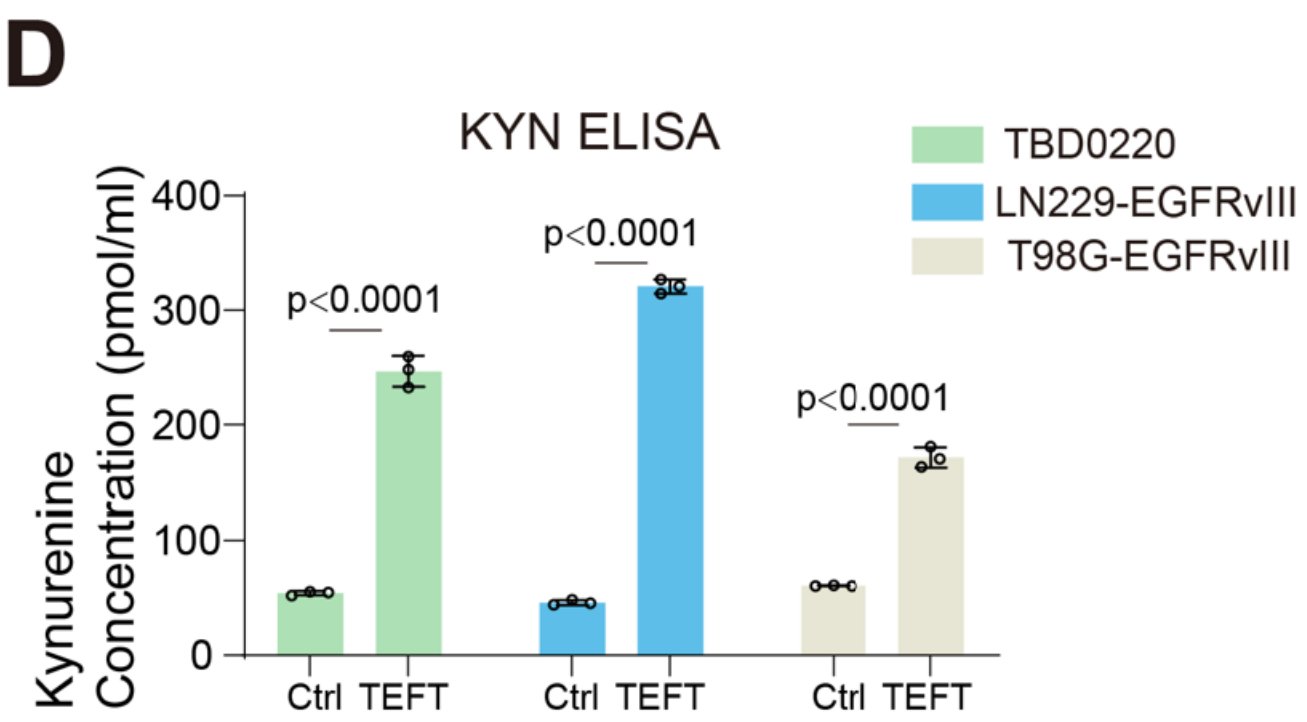


**fig. S10** (**D**) Kynurenine ELISA showing increased extracellular kynurenine levels in the culture supernatants of TBD0220, LN229-EGFRvIII, and T98G-EGFRvIII cells after TEFT treatment.

Second, we examined AhR subcellular localization in multiple EGFRvIII-driven models. TEFT promoted AhR nuclear accumulation in LN229-EGFRvIII, T98G-EGFRvIII, TBD0220, and patient-derived LG-260 cells. IDO1 depletion or EGFR inhibition with osimertinib reduced this nuclear accumulation, placing AhR activation downstream of the EGFRvIII–IDO1 arm. The relevant changes made in the results have been added to Fig 5A, B and fig. S10A, B.

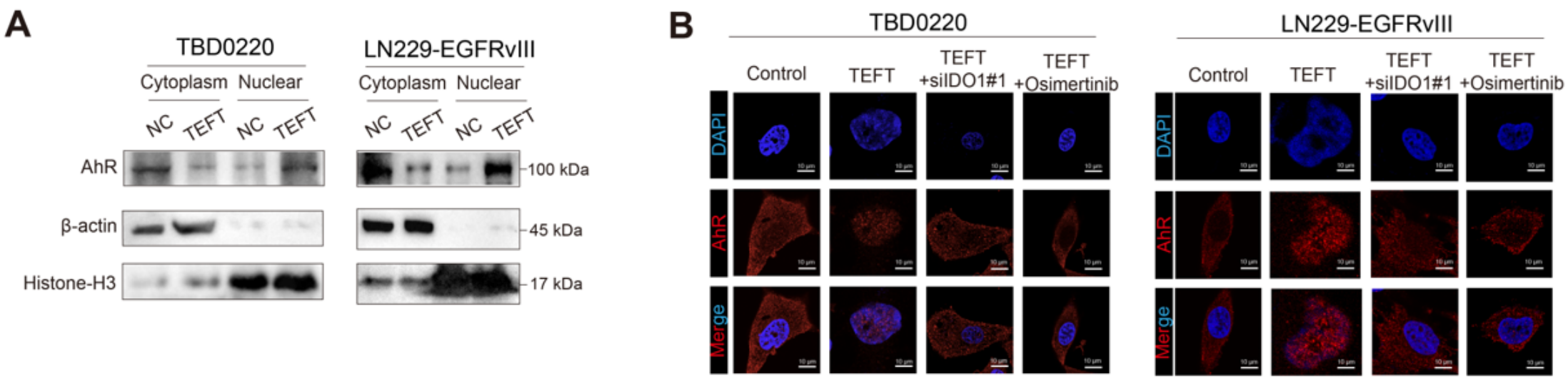


**Fig. 5 (A)** Western blot analysis of AhR in cytoplasmic and nuclear fractions of TBD0220 and LN229 EGFRvIII cells. TEFT treatment promotes AhR nuclear translocation, **(B)** Immunofluorescence staining of AhR (red) and DAPI (blue). TEFT induces AhR nuclear accumulation, which is reversed by IDO1 knockdown or osimertinib. Scale bars, 10 μm.

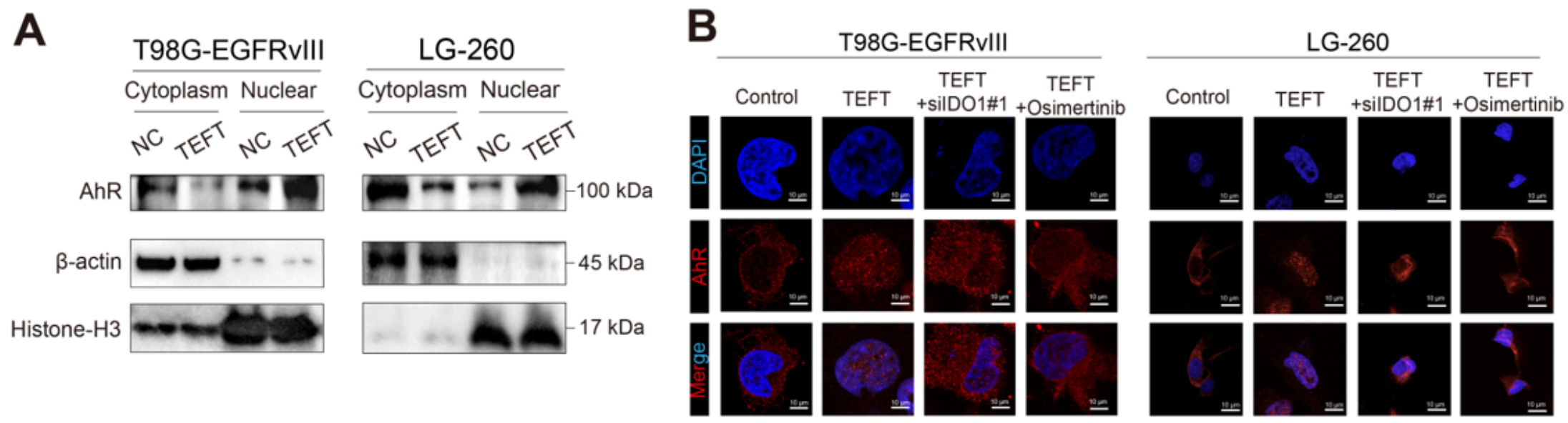


**fig. S10 (A)** Western blot analysis of AhR in cytoplasmic and nuclear fractions of T98G-EGFRvIII and patient-derived LG-260 cells treated with or without TEFT, (**B**) Representative immunofluorescence images of AhR in T98G-EGFRvIII and LG-260 cells under the indicated conditions. TEFT induced AhR nuclear accumulation, whereas IDO1 knockdown or osimertinib treatment reduced AhR nuclear localization. DAPI, nuclei; AhR, red. Scale bars, 10 μm.

Third, we evaluated transcriptional activation. TEFT increased accessibility of AhR/ARNT motifs and induced the canonical AhR-responsive genes CYP1A1 and CYP1B1. These results indicate that AhR nuclear accumulation is accompanied by execution of an AhR-associated transcriptional program. The relevant changes made in the results have been added to fig. S10C.

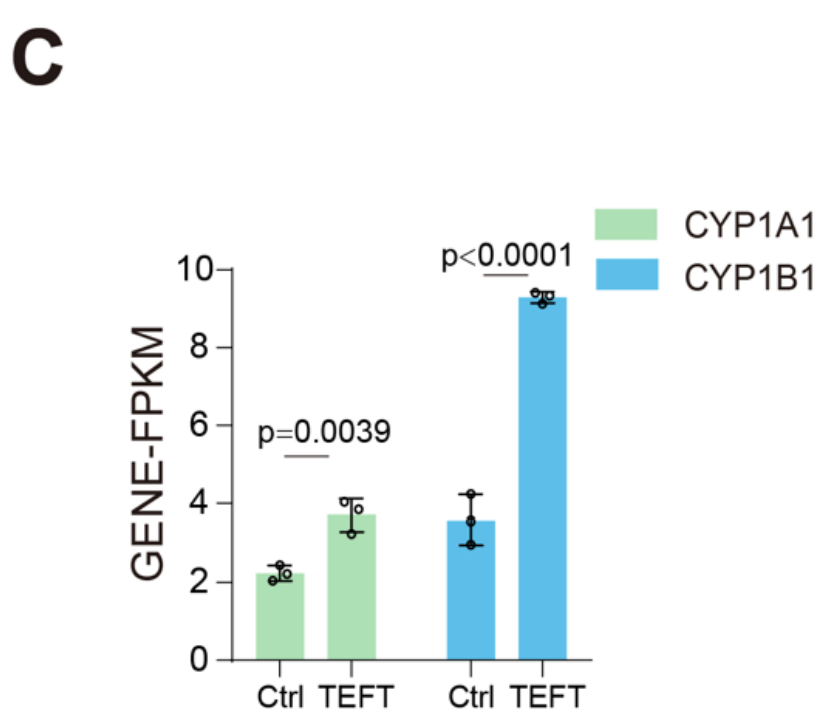


**fig. S10** (**C**) Gene expression levels of canonical AhR target genes CYP1A1 and CYP1B1 in control and

TEFT-treated EGFRvIII-LN229 cells, shown as FPKM values.

Fourth, we connected this transcriptional program to the resistant phenotype. AhR ChIP-qPCR showed increased occupancy at predicted regulatory regions within the DCLK1 and ARHGEF2 promoters. DCLK1 depletion disrupted spindle organization and increased abnormal nuclear morphology, whereas ARHGEF2 depletion reduced the microtubule-polymerizing activity associated with the resistant state. Similar structural effects were observed after IDO1 depletion, epacadostat, or osimertinib treatment. The relevant changes made in the results have been added to fig. S11A, B and Fig. 5E, L.

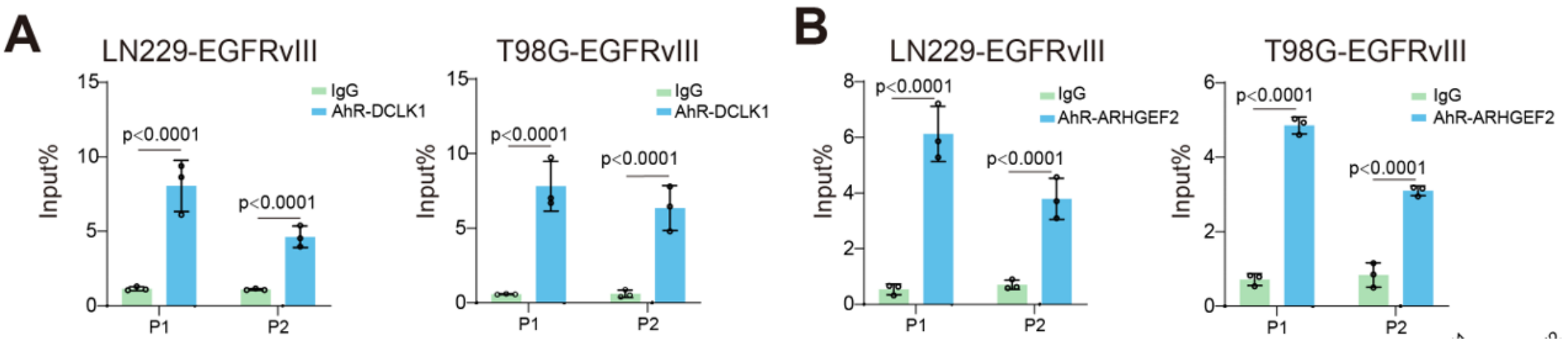


**fig. S11 (A**) ChIP-qPCR analysis of AhR occupancy at the predicted *DCLK1* promoter regions (P1 and P2) in LN229 and T98G EGFRvIII cells, **(B)** ChIP-qPCR analysis of AhR occupancy at the *ARHGEF2* promoter regions (P1 and P2).

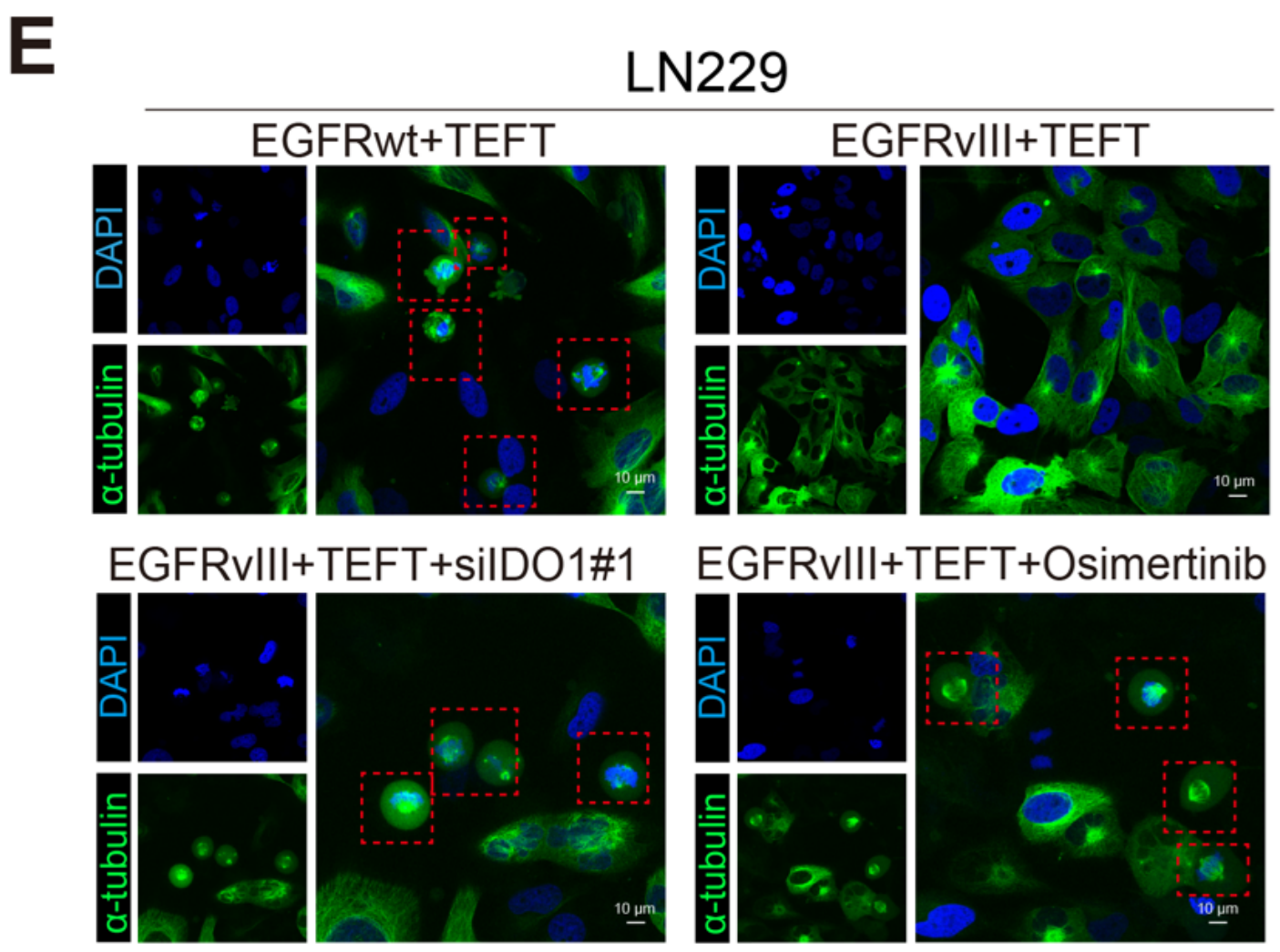


**Fig. 5 (E)** Representative immunofluorescence images of mitotic spindles (α-tubulin, green) and chromosomes (DAPI, blue) in LN229 cells. TEFT causes severe spindle disruption and multipolar mitosis in wild-type cells, whereas EGFRvIII cells maintain bipolar spindles. This protection is lost upon IDO1 knockdown or osimertinib treatment.

**L**

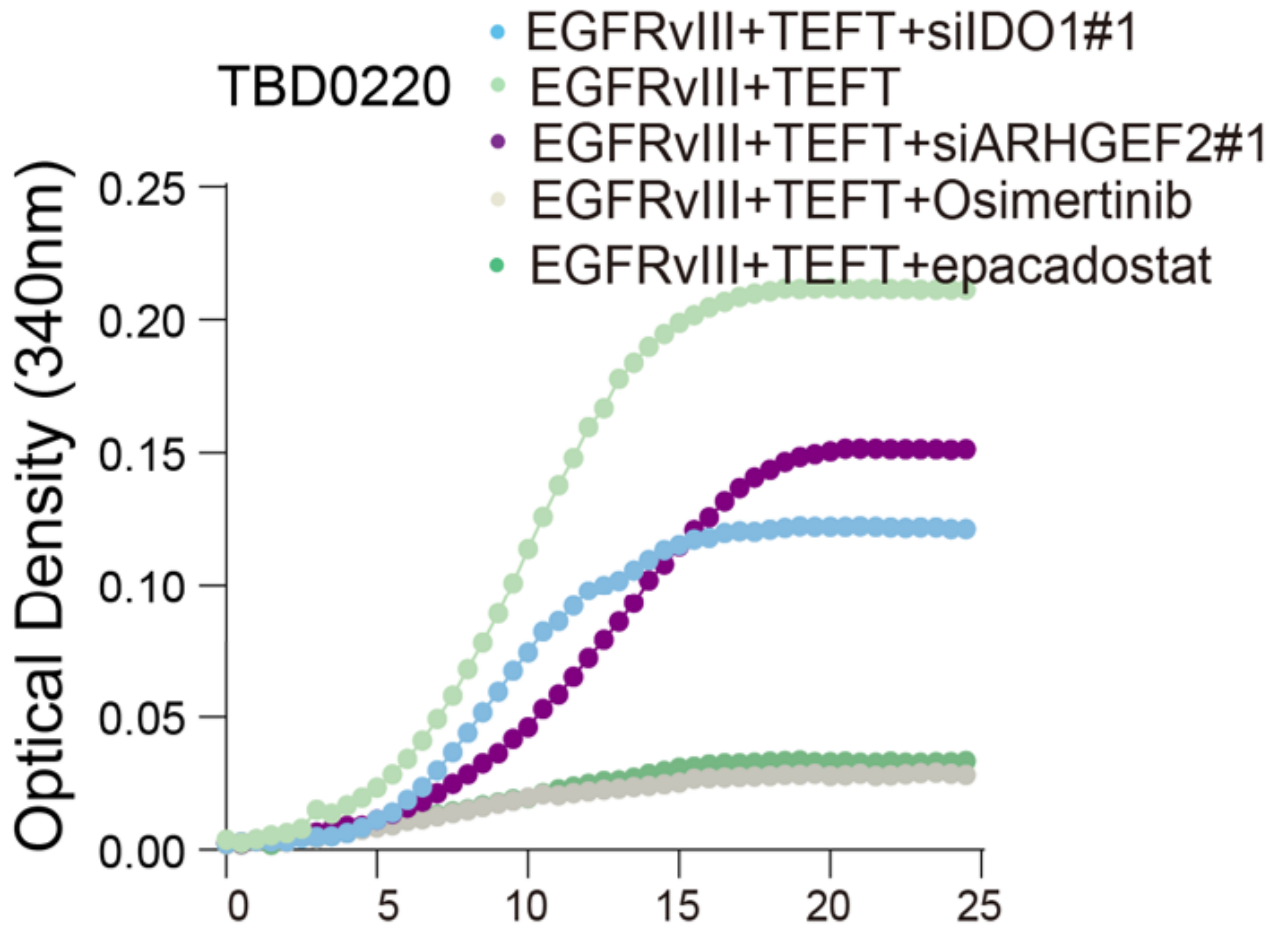


**Fig. 5 (L)** In vitro tubulin polymerization assay in TBD0220 cells under the indicated conditions. IDO1 knockdown, ARHGEF2 knockdown, osimertinib, or epacadostat reduced the microtubule-polymerizing activity associated with EGFRvIII-mediated TEFT resistance.

Together, these biochemical, spatial, transcriptional, and functional data support activation of an IDO1–kynurenine–AhR-associated cytoskeletal protection program under TEFT. Because the present revision does not include direct AhR genetic ablation, we have avoided claiming that AhR alone is an exclusive determinant of resistance. Instead, the revised manuscript presents AhR as a transcriptionally active downstream mediator linking IDO1 metabolism to DCLK1/ARHGEF2-dependent spindle and microtubule protection.

**Other Changes in the revised manuscript are provided below.**
**Results, lines 329–388;**

The stabilization of IDO1 initiates a crucial downstream signaling cascade by converting tryptophan into kynurenine, a high-affinity endogenous ligand for the aryl hydrocarbon receptor (AhR) (*35, 36*). To determine whether TEFT-stabilized IDO1 remained enzymatically active, we measured kynurenine levels in culture supernatants. Kynurenine ELISA showed that TEFT increased extracellular kynurenine concentrations in EGFRvIII-driven GBM models, including TBD0220, LN229-EGFRvIII, and T98G-EGFRvIII cells (fig. S10D). We found that TEFT treatment in EGFRvIII cells significantly promoted AhR nuclear translocation (Fig. 5A, B), supporting its activation. Similar AhR nuclear accumulation was observed in T98G-EGFRvIII and patient-derived LG-260 cells (fig. S10A, B). Importantly, IDO1 knockdown or EGFR inhibition with osimertinib reduced TEFT-induced AhR nuclear accumulation, supporting placement of AhR activation downstream of EGFRvIII-dependent IDO1 signaling (Fig. 5B and fig. S10B).

Further genomic analysis using our ATAC-seq data revealed enrichment of AhR/ARNT motifs in EGFRvIII cells under TEFT exposure (Fig. 5C). Consistent with transcriptionally active AhR signaling, RNA-seq analysis showed induction of the canonical AhR-responsive genes CYP1A1 and CYP1B1 after TEFT treatment (fig. S10C). To identify the physiological targets of this axis, we evaluated AhR target

genes in the public ChIP-Atlas and GTRD databases(*37*) and performed GO enrichment analysis. The results showed that "Mitotic Cell Cycle" and "Microtubule Cytoskeleton" were among the most highly enriched pathways (Fig. 5D), consistent with the core biophysical mechanism of TEFT(*11, 38, 39*). This molecular observation directed us to investigate the phenotypic consequences on mitosis. Phenotypic analysis demonstrated that while TEFT typically induces mitotic defects, characterized by spindle misalignment and abnormal nuclear morphology in control cells, EGFRvIII expression conferred a structural protective effect, maintaining bipolar spindle integrity and reducing the percentage of abnormal nuclei. This protection was impaired upon IDO1 silencing or inhibition with osimertinib**,** supporting a functional role for IDO1 signaling in TEFT resistance (Fig. 5E, F). Similar spindle-disruptive effects of IDO1 knockdown, osimertinib, or the IDO1 inhibitor epacadostat were observed in TBD0220 and LN229-EGFRvIII cells (fig. S10E-J). Together, these findings support an IDO1-kynurenine-AhR cascade that contributes to mitotic and microtubule protection during TEFT resistance.

To identify the downstream structural effectors, we employed a multi-omics intersection strategy. Specifically, we intersected public AhR cistrome data from ChIP-Atlas(*37*), ATAC-seq data, and our RNA-seq data to screen for genes regulating spindle assembly, which led to the identification of DCLK1 (doublecortin-like kinase 1) (Fig. 5G). To validate the direct regulatory link, we performed ChIP-qPCR assays, which detected AhR occupancy at the predicted DCLK1 and ARHGEF2 promoter regions, supporting direct AhR-dependent transcriptional regulation of DCLK1 **(**fig. S11A). We confirmed that TEFT treatment upregulated DCLK1 protein levels in EGFRvIII-expressing LN229 and T98G cells (Fig. 5H). Functionally, DCLK1 contributed to the observed structural protection(*40*, *41*); silencing DCLK1 in EGFRvIII cells disrupted spindle organization and increased abnormal nuclear morphology (Fig. 5I, J). These effects were further validated in TBD0220 and T98G-EGFRvIII cells (fig. S11C-F). Thus, DCLK1 functions as a direct AhR-regulated mitotic effector that contributes to cytoskeletal protection under TEFT stress.

To further dissect the mechanism underlying microtubule stability under physical stress, we sought to identify effectors that directly associate with the cytoskeleton. We performed immunoprecipitation coupled with mass spectrometry (IP-MS) using anti-tubulin antibodies in TEFT-treated EGFRvIII cells to isolate the tubulin interactome. By intersecting these proteomic data with our genomic ATAC-seq and public AhR target datasets, we identified ARHGEF2 (GEF-H1) as a microtubule-associated effector (Fig. 5K) (*17*, *42-46*). Similar to DCLK1, ChIP-qPCR analysis demonstrated increased AhR occupancy at predicted motifs within the ARHGEF2 promoter in LN229-EGFRvIII and T98G-EGFRvIII cells after TEFT treatment, supporting ARHGEF2 as a direct transcriptional target of AhR (fig. S11B). Efficient ARHGEF2 knockdown was confirmed at both the mRNA and protein levels (fig. S11G, H). To validate its functional role, we performed in vitro tubulin polymerization assays. Lysates from TEFT-treated EGFRvIII cells exhibited a capacity to promote microtubule polymerization. Importantly, this stabilizing effect was attenuated by IDO1 knockdown, ARHGEF2 knockdown, osimertinib, or epacadostat in TBD0220 cells (Fig. 5L), with consistent effects observed in LN229-EGFRvIII and T98G-EGFRvIII cells (fig. S11I). These complementary perturbations place IDO1 activity, EGFR signaling, and AhR-regulated ARHGEF2 within the same functional cytoskeletal protection program rather than representing independent correlative events.

**Discussion, lines 507–522;**

Downstream of IDO1, our study uncovers a novel function for the Aryl Hydrocarbon Receptor (AhR). Typically viewed as a sensor for environmental toxins(*61*), our data support a role for AhR in linking tryptophan metabolism to cytoskeletal adaptation in EGFRvIII-driven GBM under TEFT stress. Increased kynurenine production, AhR nuclear translocation, and induction of canonical AhR-responsive genes collectively support activation of this pathway. AhR activation is associated with increased transcription of DCLK1 and ARHGEF2, with ChIP-qPCR supporting AhR occupancy at their predicted promoter regions. DCLK1 is a microtubule-associated kinase involved in bipolar spindle formation(*40*, *41*), while ARHGEF2/GEF-H1 regulates RhoA-dependent microtubule dynamics(*17*, *46*). Promoter ChIP-qPCR and functional perturbation experiments support DCLK1 and ARHGEF2 as direct AhR-regulated cytoskeletal effectors in this setting. In the context of TEFT, which physically disrupts dipole alignment and spindle assembly, upregulation of these factors supports spindle organization and microtubule polymerization, thereby helping tumor cells tolerate electric-field-induced mitotic stress**.** This finding links metabolic inputs, particularly the tryptophan metabolite kynurenine, to physical structural adaptations of the cytoskeleton and provides a mechanistic explanation for how tumor cells can partially buffer the anti-mitotic effects of physical therapy.

6. The quality of Fig. 1c and Fig. 5A AhR panel is unacceptable for publication.

**Response:** We sincerely apologize for the suboptimal quality of the original Western blot images and fully agree that these panels did not meet the required publication standard. To address this concern, we repeated the Western blot experiments shown in Fig. 1C and Fig. 5A under the same experimental conditions and replaced the original panels with new representative images.

For Fig. 1C, we repeated the analysis of basal EGFR and EGFRvIII expression across U118MG, A172, LN229, T98G, and the patient-derived EGFRvIII-positive TBD0220 cells. The revised panel displays clearer and more readily interpretable EGFR and EGFRvIII bands, with the corresponding loading control included.

For Fig. 5A, we repeated the nuclear and cytoplasmic fractionation experiments in TBD0220 and LN229-EGFRvIII cells. The revised Western blot panel clearly shows increased AhR abundance in the nuclear fraction following TEFT treatment, with appropriate cytoplasmic and nuclear fraction controls. This result is independently supported by the imaging results obtained in T98G-EGFRvIII and patient-derived LG-260 cells in fig. S10A.

Both original panels have now been replaced in the revised manuscript.

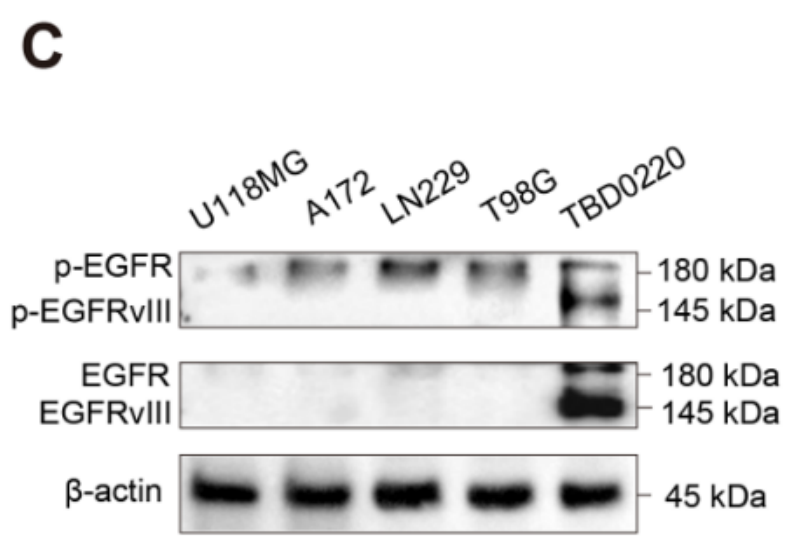

**Fig. 1 (C)** Western blot analysis of basal EGFR and EGFRvIII phosphorylation and expression levels in a panel of GBM cell lines (U118MG, A172, LN229, T98G) and a primary EGFRvIII-mutant line (TBD0220). β-actin served as a loading control.

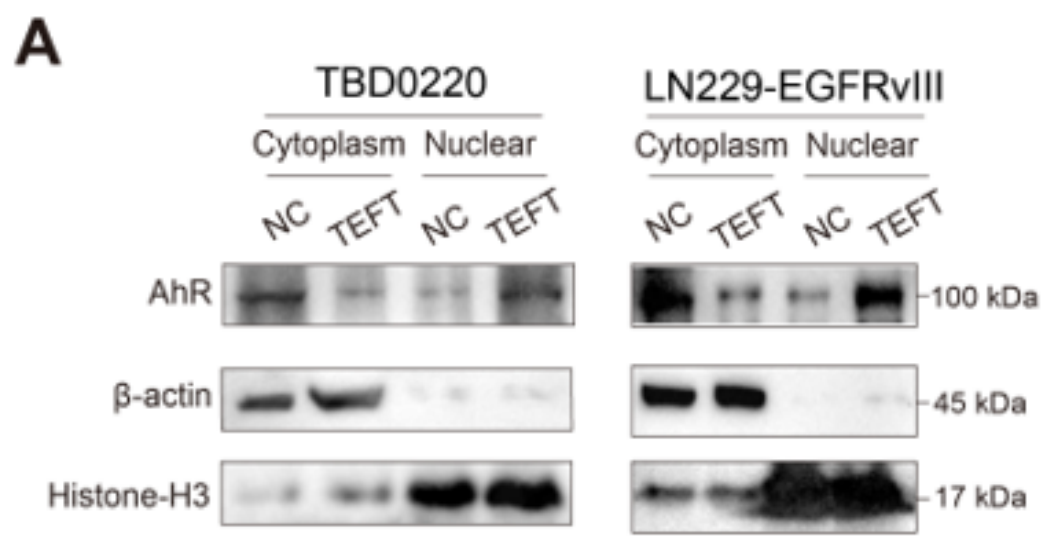


**Fig. 5 (A**) Western blot analysis of AhR in cytoplasmic and nuclear fractions of TBD0220 and LN229 EGFRvIII cells. TEFT treatment promotes AhR nuclear translocation.

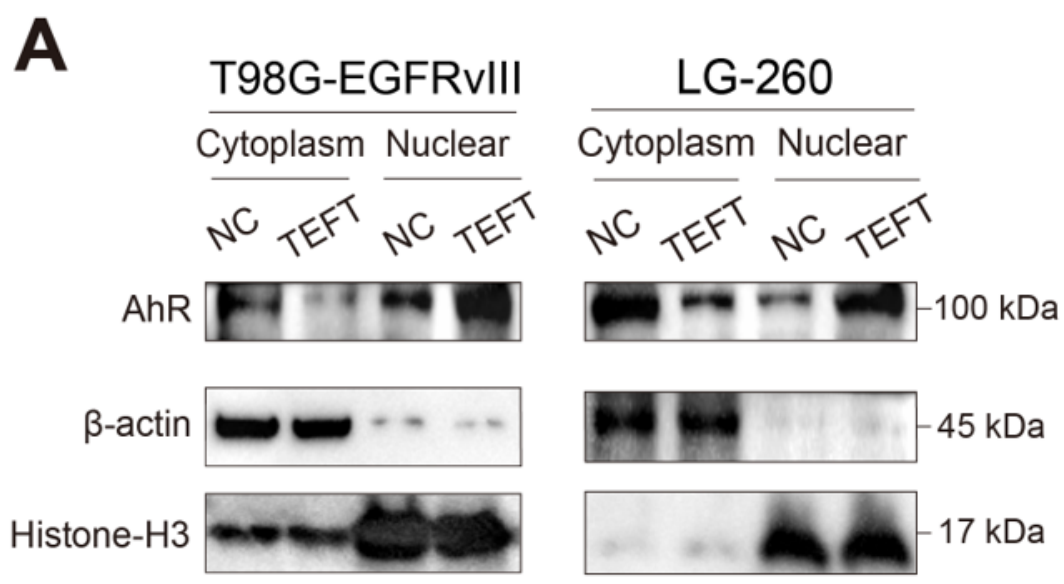


**fig. S10 (A**) Western blot analysis of AhR in cytoplasmic and nuclear fractions of T98G-EGFRvIII and patient-derived LG-260 cells treated with or without TEFT.

### Responses to the comments of Reviewer #2

Reviewer 2 Comments to the Author: Feng et al. investigate why a subset of GBM exhibits intrinsic resistance to tumor electric field therapy (TEFT). Using a retrospective clinical cohort together with cell and organoid models, they propose that EGFRvIII drives resistance by increasing IDO1 transcription through NF-κB. In parallel, TEFT-induced bioenergetic stress activates an AMPK-PPARα program that upregulates CPT1A, which the authors propose stabilizes IDO1 via succinylation and inhibition of TRIM21-mediated ubiquitination. Stabilized IDO1 is then suggested to activate AhR signaling to induce DCLK1/ARHGEF2 and preserve spindle/microtubule integrity during TEFT. Finally, the authors present preclinical evidence supporting osimertinib plus TEFT as a strategy to reverse resistance, along with a small proof-of-concept clinical experience.

Overall, the study is ambitious and conceptually interesting, with a plausible resistance axis linking metabolic adaptation to mitotic fidelity and an attractive translational hypothesis. The work is timely given the growing clinical use of TEFT across multiple cancer types and the continued uncertainty around its mechanism of action. However, major concerns regarding model choice, insufficient mechanistic localization/validation

of the CPT1A-IDO1 interaction, and overinterpretation of a very limited clinical experience substantially reduce confidence in the generality and impact of the claims.

**Response**: We sincerely thank the reviewer for the rigorous and constructive assessment of our study. We agree that the original manuscript required stronger validation in clinically relevant GBM models, more direct mechanistic evidence for the CPT1A–IDO1 interaction, clearer functional interrogation of the proposed pathway, more quantitative evaluation of the therapeutic combination, and greater restraint in interpreting the preliminary clinical observations. We have substantially revised the manuscript and performed extensive additional experiments to address these concerns.

First, we expanded the study beyond long-established GBM cell lines. The patient-derived EGFRvIII-positive TBD0220 culture was carried through the central mechanistic workflow, including IDO1 loss-of-function, CPT1A–IDO1 mitochondrial-fraction interaction, protein colocalization, kynurenine production, AhR activation, and spindle and microtubule analyses. We also introduced two independently established low-passage patient-derived cultures: LG-260, which harbors EGFR amplification and EGFRvIII, and LG-252, which lacks the corresponding EGFRvIII-driven signaling profile. These models were complemented by patient-derived glioblastoma organoids and orthotopic xenograft models.

Second, we strengthened the spatial and biochemical evidence for the CPT1A–IDO1 mechanism. Surface plasmon resonance using purified recombinant proteins supported direct binding, whereas mitochondrial fractionation followed by co-immunoprecipitation demonstrated preferential enrichment of the TEFT-enhanced CPT1A–IDO1 complex in the mitochondrial fraction. Fraction quality was assessed using mitochondrial and non-mitochondrial markers. High-resolution three-dimensional z-stack imaging and line-scan analyses independently visualized increased spatial overlap between IDO1 and CPT1A-positive mitochondrial structures. We further added CPT1A depletion and mutant-rescue experiments, cell-free succinylation assays, succinyl-CoA measurements, and IDO1 lysine-mutant analyses to establish the functional contribution of CPT1A LSTase-related activity and the K377/K389 regulatory region.

Third, we expanded the functional perturbation of the proposed pathway. Two independent IDO1-targeting siRNAs and the catalytic IDO1 inhibitor epacadostat restored TEFT sensitivity in established and patient-derived EGFR-driven models. Kynurenine measurements confirmed that TEFT-stabilized IDO1 remained enzymatically active. IDO1 depletion or osimertinib reduced AhR nuclear accumulation, placing AhR activation downstream of EGFRvIII-dependent IDO1 signaling. Promoter ChIP-qPCR, DCLK1 and ARHGEF2 depletion, spindle imaging, and tubulin-polymerization assays further established the functional hierarchy linking the IDO1–kynurenine–AhR pathway to cytoskeletal protection.

Fourth, we quantified the pharmacological interaction between TEFT and osimertinib. Dose–response experiments demonstrated that TEFT shifted the osimertinib response curves toward lower concentrations, and Bliss analysis supported a synergistic interaction in multiple EGFRvIII-driven models. A subtherapeutic osimertinib dose was subsequently used in the orthotopic experiments, in which the combination produced greater tumor suppression and survival prolongation than either monotherapy.

Fifth, we substantially moderated the clinical interpretation. The retrospective cohort association between EGFRvIII status and shorter progression-free survival is now explicitly described as hypothesis-generating. The osimertinib-plus-TEFT cases are presented as exploratory, mechanism-informed observations rather than evidence of clinical efficacy or validation of a predictive biomarker. The revised manuscript clearly states that prospective, molecularly stratified clinical studies will be required to determine whether EGFRvIII, EGFR amplification, or another measure of EGFR-pathway activation predicts TEFT response or benefit from the combination.

Finally, we reorganized and streamlined the manuscript around a genotype–field convergence model. In this framework, TEFT induces a broadly inducible mitochondrial and metabolic-stress response through the AMPK–PPARα–CPT1A axis, whereas EGFRvIII independently provides NF-κB-dependent transcriptional priming of IDO1. The convergence of these two inputs enables CPT1A-dependent IDO1 stabilization and activation of the kynurenine–AhR–DCLK1/ARHGEF2 cytoskeletal protection program. An integrated schematic has been added, and TEFT frequency, intensity, exposure duration, and temperature-control conditions have been standardized throughout the Methods and figure legends.

We believe that these additions substantially improve the biological relevance, mechanistic depth, causal support, and translational framing of the study while more clearly defining the limitations and remaining questions. The complete experimental data, statistical analyses, and corresponding interpretation are provided in the revised manuscript.

1. A major limitation is the heavy reliance on obsolete, high-passage GBM lines (e.g., LN229, T98G) for most mechanistic work. The only primary EGFRvIII line (TBD0220) appears early (Fig. 1C–D) but is not carried through the core mechanistic experiments (Figs. 2–5). Key conclusions (IDO1 dependence, CPT1A-driven IDO1 stabilization on the mitochondrial membrane, and AhR–DCLK1/ARHGEF2 axis) should be reproduced in multiple low-passage, patient-derived GBM cultures with and without EGFRvIII (and ideally with defined EGFR alterations). At minimum, the central mechanistic nodes should be validated in TBD0220 plus additional independent patient-derived models.

**Response:** We agree that mechanistic conclusions based predominantly on long-established GBM cell lines may not fully preserve the molecular dependencies of patient tumors. We therefore expanded the revised study using patient-derived GBM models with defined EGFR alterations.

The patient-derived EGFRvIII-positive TBD0220 model was carried throughout the central mechanistic workflow, including IDO1 depletion and pharmacological inhibition, mitochondrial-fraction CPT1A–IDO1 interaction assays, IDO1 succinylation and ubiquitination analyses, high-resolution spatial imaging, kynurenine measurement, AhR activation, and spindle and microtubule assays.

We also introduced two independent low-passage patient-derived cultures established from freshly resected tumors. LG-260 harbors EGFR amplification and EGFRvIII and displayed limited apoptosis after TEFT alone, whereas either osimertinib or the IDO1 inhibitor epacadostat enhanced TEFT-induced apoptosis. LG-252, which lacks the corresponding EGFRvIII-driven signaling profile, remained comparatively sensitive to TEFT. Both cultures were maintained under serum-free neural stem-cell conditions and used between passages 3 and 7.

In addition, TEFT-associated mitochondrial enrichment of IDO1 and CPT1A, CPT1A–IDO1 association, AhR nuclear accumulation, and pharmacological resensitization were independently evaluated in patient-derived systems. Patient-derived glioblastoma organoids with defined EGFR amplification and EGFRvIII alterations were also used to validate the resistance-associated CPT1A–IDO1 phenotype and its modulation by osimertinib.

The complete mechanistic workflow was carried through in TBD0220, whereas LG-260 and LG-252 provided independent patient-derived validation of the genotype-associated phenotype, selected pathway nodes, and pharmacological resensitization. The roles and validations performed in the three patient-derived cultures are summarized below:

| Model | Type | EGFR status | TEFT phenotype | Key validations added |
|---|---|---|---|---|
| TBD0220 | Patient-derived GBM culture | EGFRvIII-positive | TEFT-resistant | IDO1 dependence, CPT1A-IDO1 mitochondrial fraction Co-IP, colocalization, kynurenine ELISA, epacadostat/siIDO1 rescue, spindle/microtubule assays |
| LG-260 | Patient-derived GBM culture | EGFR amplification / EGFRvIII-positive | TEFT-resistant | EGFR activation, TEFT resistance, CPT1A-IDO1 mitochondrial fraction Co-IP, osimertinib/epacadostat sensitization |
| LG-252 | Patient-derived GBM culture | EGFRvIII-negative / non-EGFR-driven | TEFT-sensitive | Comparator model; TEFT-induced apoptosis; |

**Changes in the revised manuscript:**

Results, lines 130–156, 198–215, 262–327, and 328–388; Methods, lines 568–605; Figs. 1K, L, 3E–G, 4B, H, I, and 5A–L; figs. S3A–H, S4I–L, S8I–L, S9A–C, S10A–D, and S11C–I.

2. CPT1A-IDO1 localization/interaction needs stronger support. The manuscript does not convincingly establish where CPT1A binds IDO1 under TEFT or whether this occurs at mitochondria versus other compartments. Colocalization is suggestive but not definitive (Fig. 4K, Fig. 6F). Strengthen this with higher-resolution, quantified localization (z-stacks with mitochondrial markers) and compartment-specific interaction assays (co-IP/PLA from mitochondrial vs cytosolic fractions with purity controls). Also map the binding interface and test binding-deficient mutants for loss of IDO1 stabilization under TEFT.

**Response:** We agree that conventional colocalization alone cannot establish either direct protein binding or the subcellular compartment in which the interaction occurs. We therefore added complementary biophysical, compartment-specific biochemical, high-resolution imaging, structural-modeling, and functional-mutagenesis analyses.

First, surface plasmon resonance using purified recombinant proteins demonstrated direct binding between CPT1A and IDO1, establishing that their association does not require an additional cellular adaptor. The results are shown in Fig. 4A.

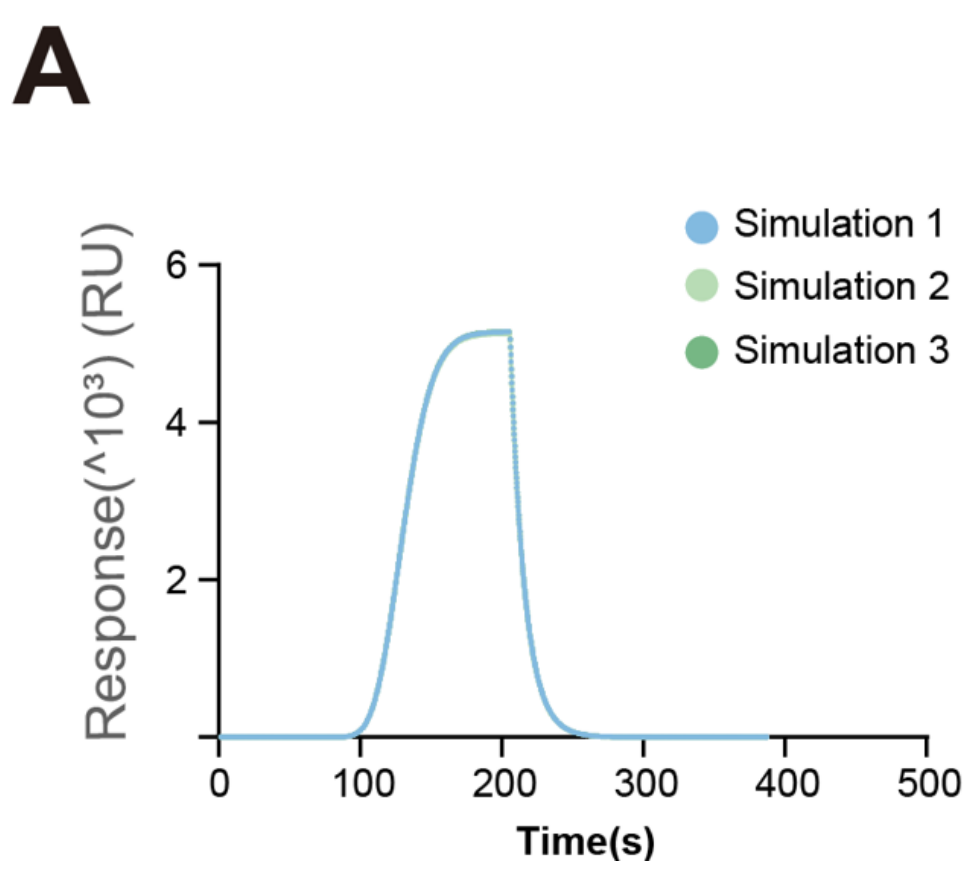


**Fig. 4 (A)** Surface plasmon resonance (SPR) analysis showing the direct physical interaction between purified CPT1A and IDO1 proteins.

Second, we performed subcellular fractionation followed by IDO1 co-immunoprecipitation in TBD0220 and LN229-EGFRvIII cells. TEFT enhanced CPT1A–IDO1 complex formation, and the complex was enriched in the mitochondrial fraction. ATP5A was used as a mitochondrial marker, whereas Calnexin was used as an endoplasmic-reticulum contamination control. TEFT-enhanced CPT1A–IDO1 association was independently reproduced in the patient-derived LG-260 model. Because CPT1A is an outer mitochondrial membrane-resident protein, these findings support recruitment of IDO1 to a CPT1A-associated mitochondrial interface rather than import of IDO1 into the mitochondrial matrix. The relevant changes made in the results have been added to Fig. 4B (TBD0220 and LN229-EGFRvIII) and fig. S8B(LG-260).

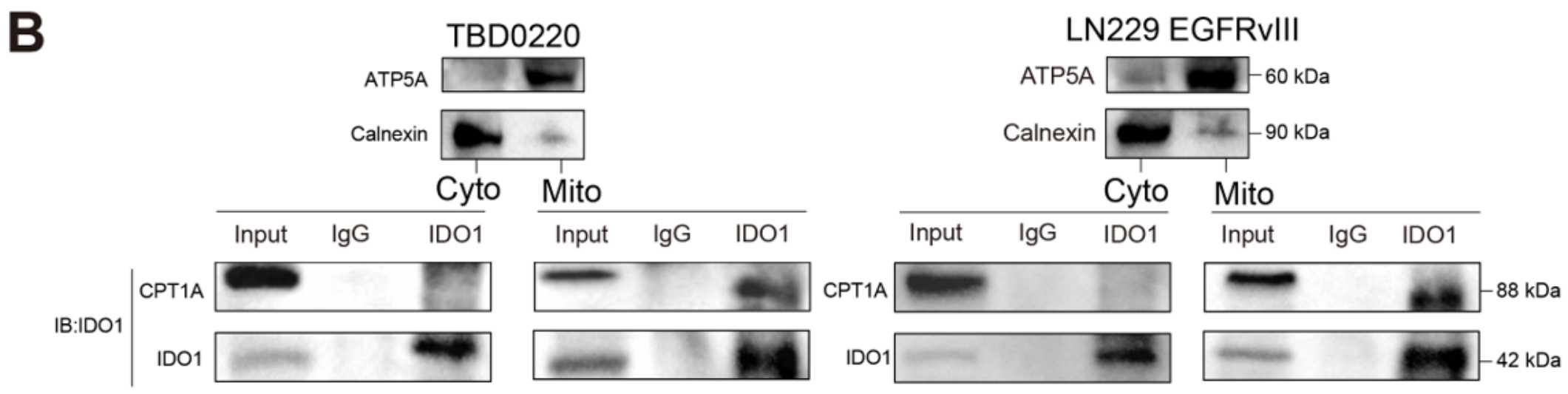


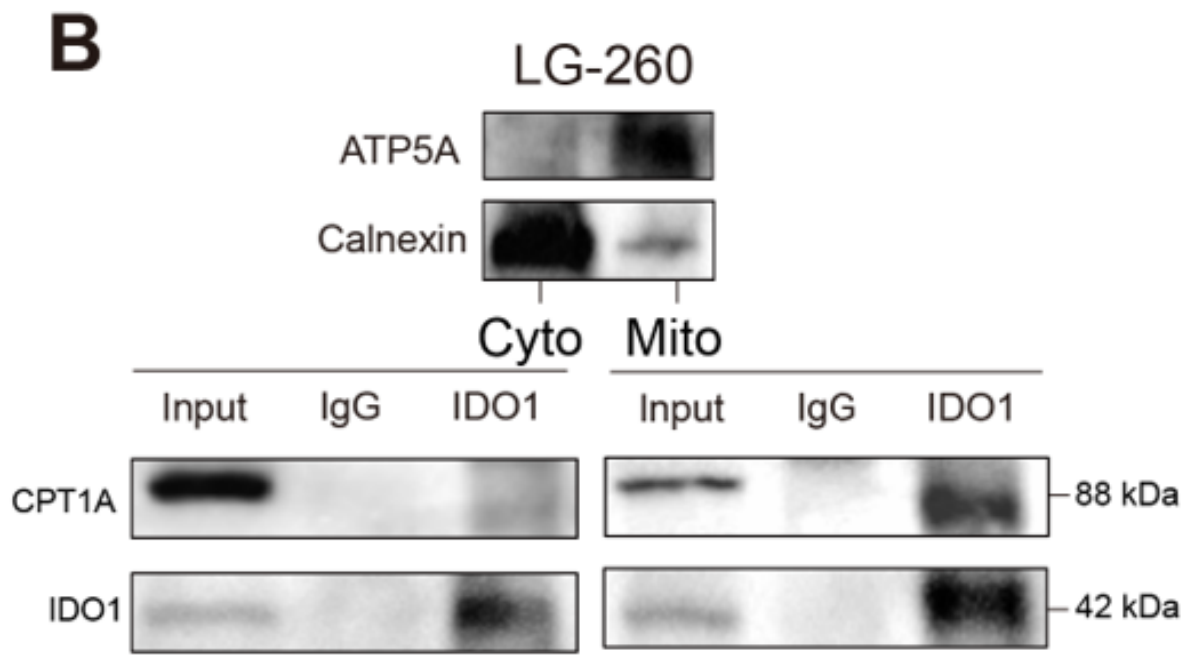


**Fig. 4 and fig. S8 (B)** Subcellular fractionation followed by IDO1 co-immunoprecipitation in TBD0220, LN229-EGFRvIII and patient-derived LG-260 cells. The CPT1A–IDO1 complex was enriched in the mitochondrial fraction. ATP5A and Calnexin were used to assess mitochondrial and non-mitochondrial fraction quality, respectively.

Third, we performed high-resolution three-dimensional z-stack imaging followed by reconstruction in Imaris. TEFT increased the spatial overlap between IDO1 and CPT1A-positive structures in TBD0220 and LN229-EGFRvIII cells. Conventional confocal imaging and line-scan profiles provided additional spatial support in TBD0220, LN229-EGFRvIII, and T98G-EGFRvIII cells. Because these imaging analyses were qualitative rather than coefficient-based quantitative colocalization measurements, we have revised the manuscript to avoid describing them as formal quantitative colocalization. The relevant changes made in the results have been added to Fig. 4I and fig. S9A-C.

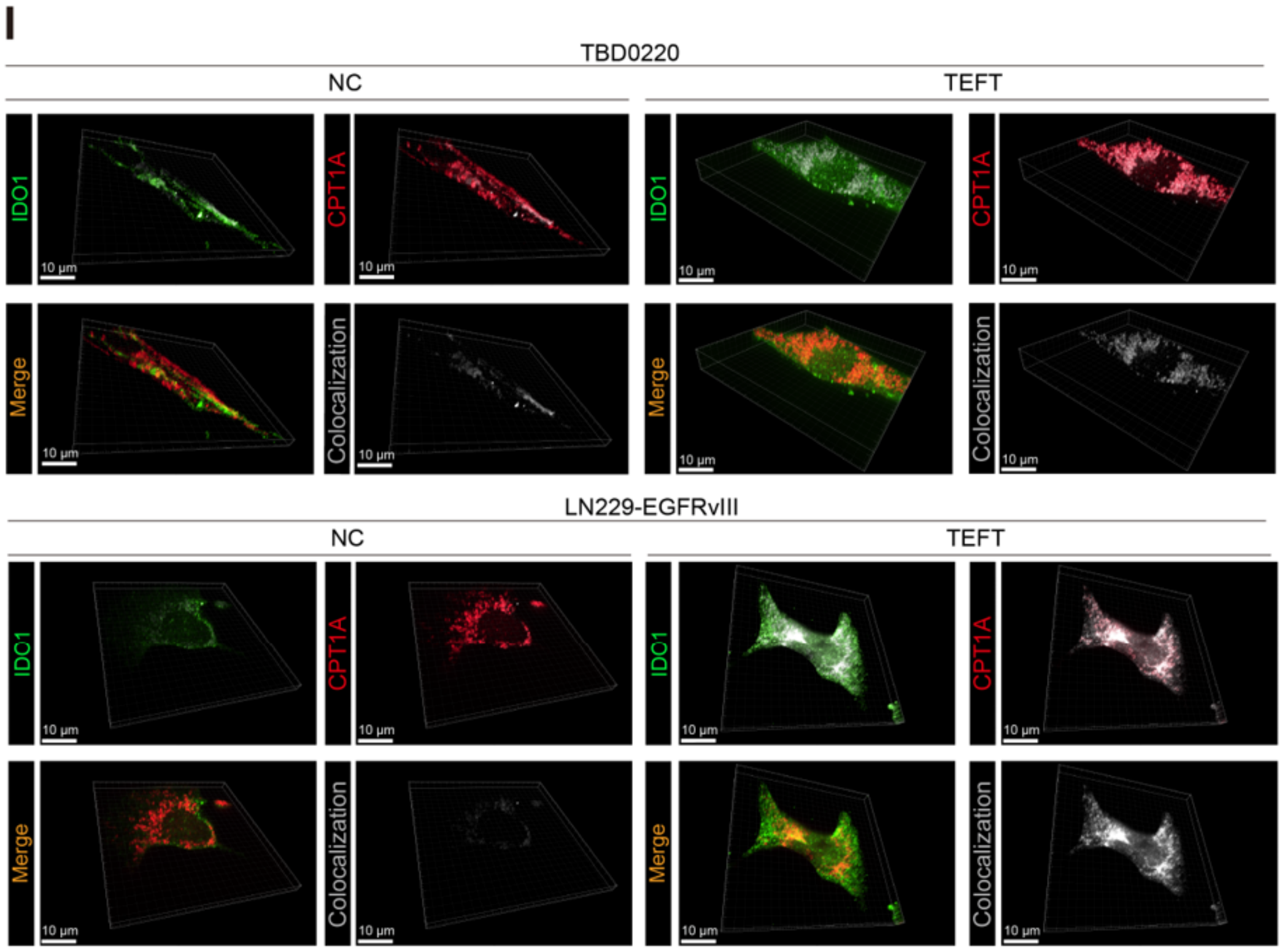


**Fig. 4 (I)** High-resolution 3D confocal imaging showing TEFT-enhanced colocalization between IDO1 and CPT1A-positive mitochondrial structures in TBD0220 and LN229-EGFRvIII cells. CPT1A was used as the OMM-resident mechanistic interactor defining the CPT1A-associated mitochondrial interface. Scale bars, 10 μm.

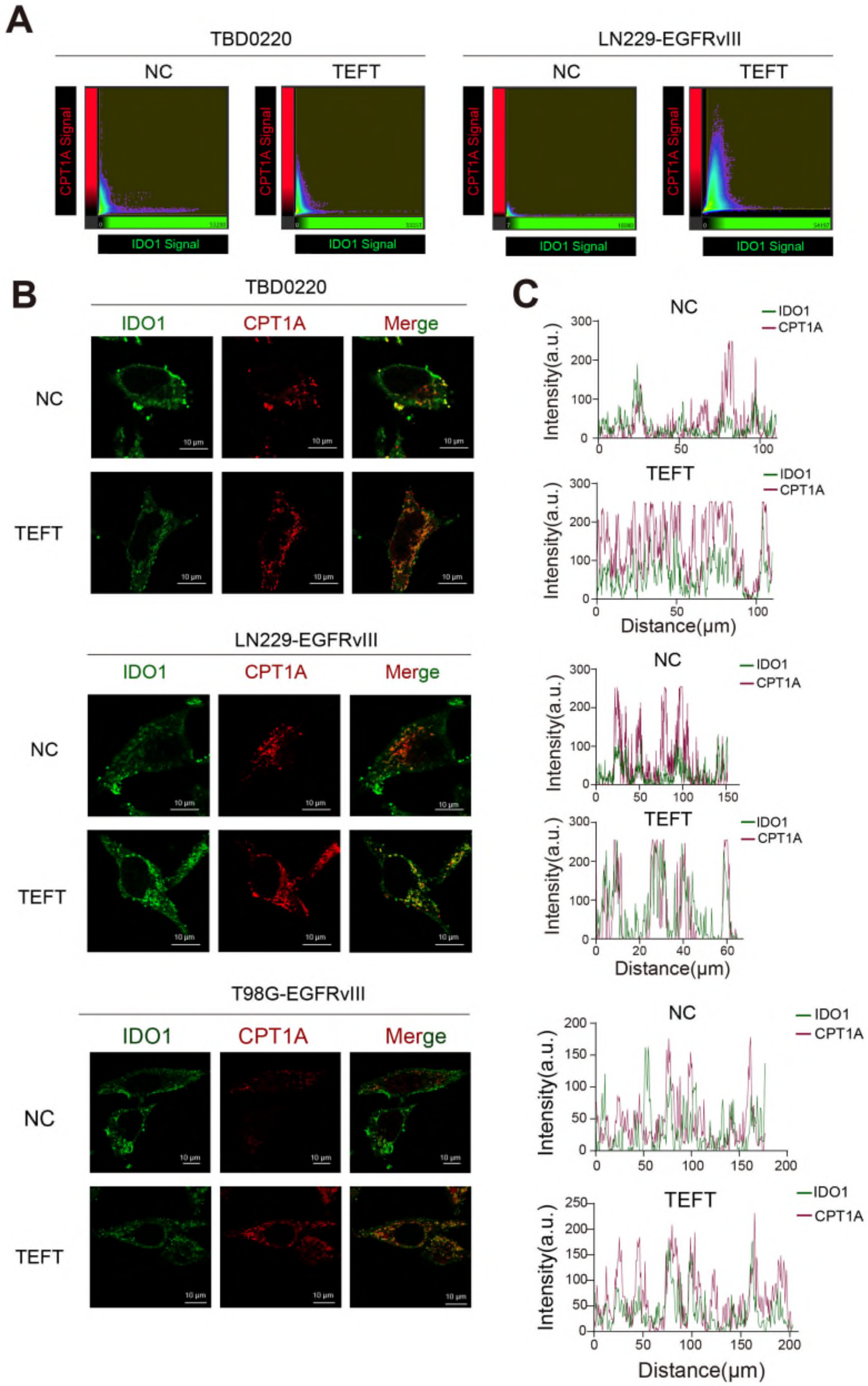


**fig. S9 (A)** Representative high-resolution Z-stack confocal colocalization analysis corresponding to Fig. 4I. The two-dimensional fluorescence intensity distribution of IDO1 and CPT1A signals in TBD0220 and LN229-EGFRvIII cells shows increased signal overlap after TEFT treatment, supporting TEFT-induced recruitment of IDO1 to CPT1A-positive mitochondrial structures, **(B)** Representative confocal

immunofluorescence images showing IDO1 and CPT1A staining in TBD0220, LN229-EGFRvIII, and T98G-EGFRvIII cells treated with or without TEFT. TEFT increased the spatial overlap between IDO1 and CPT1A-positive mitochondrial structures. IDO1, green; CPT1A, red. Scale bars, 10 μm, **(C)** Line-scan fluorescence intensity profiles of IDO1 and CPT1A signals along the indicated regions in panel B. TEFT increased the synchronization of IDO1 and CPT1A fluorescence peaks, supporting enhanced spatial proximity between the two proteins.

Fourth, AlphaFold3 modeling predicted a CPT1A–IDO1 contact surface near the IDO1 regulatory region containing K377 and K389. Functional mutagenesis further linked this region to IDO1 post-translational regulation: CPT1A-WT and the LSTase-retaining/CPTase-deficient G710E mutant restored IDO1 succinylation after CPT1A depletion, whereas the catalytically defective H473A mutant did not; independently, the IDO1-K389R mutant displayed reduced ubiquitination and increased protein stability. The results are shown in Fig. 4C-H.

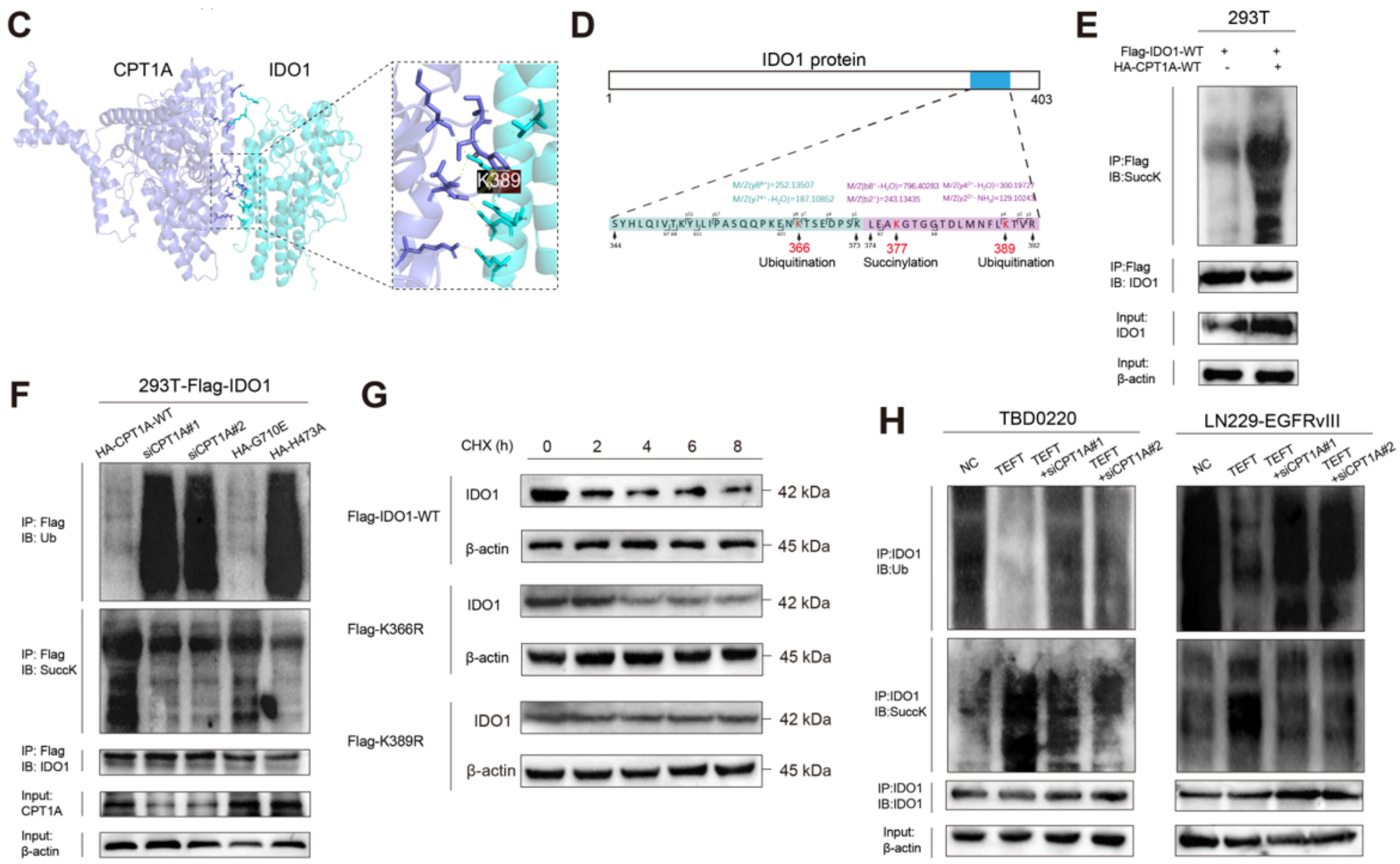


**Fig. 4 (C)** AlphaFold3-predicted protein-protein docking model of the CPT1A-IDO1 complex. The inset highlights the interaction interface covering the key lysine residues (e.g., K389), **(D)** Schematic map of IDO1 protein showing the identified post-translational modification sites: ubiquitination at K366/K389 and succinylation at K377, **(E)** Immunoprecipitation (IP) assay in 293T cells showing that CPT1A overexpression increases IDO1 succinylation (IB: SuccK) while decreasing IDO1 ubiquitination (IB: Ub), **(F)** Rescue experiment in CPT1A-knockdown cells. Re-expression of wild-type (WT) CPT1A or the LSTase-active mutant (G710E) restores IDO1 succinylation and stability, whereas the LSTase-dead mutant (H473A) fails to do so, **(G)** Cycloheximide (CHX) chase assay in 293T cells transfected with IDO1-WT or mutants (K366R, K389R). The K389R mutant exhibits a significantly extended half-life, mimicking the stabilized state, **(H)** IP analysis of endogenous IDO1 ubiquitination and succinylation in EGFRvIII-GBM cells. TEFT promotes IDO1 succinylation and reduces ubiquitination; this effect is reversed by CPT1A knockdown.

We acknowledge that these mutants interrogate catalytic activity and IDO1 post-translational regulation rather than constituting formally validated binding-deficient

mutants. Because the predicted interface spans a broader contact surface, we have not represented any single mutant as selectively abolishing binding. The revised manuscript therefore describes the interface as structurally predicted rather than experimentally resolved at residue-level resolution.

Together, the SPR, fraction-specific co-immunoprecipitation, high-resolution imaging, structural modeling, and functional mutagenesis substantially strengthen the spatial and mechanistic definition of the CPT1A–IDO1 interaction while clearly distinguishing established findings from the unresolved residue-level interface.

**Other Changes in the revised manuscript are provided below.**

**Results, lines 262–327;**

Motivated by the finding that TEFT drives mitochondrial-fraction enrichment of CPT1A and IDO1, we next investigated whether this spatial proximity facilitates a direct regulatory interaction. We hypothesized that CPT1A stabilizes IDO1 through physical binding and enzymatic modification. Surface plasmon resonance (SPR) assays supported a direct interaction between purified recombinant CPT1A and IDO1 proteins (Fig. 4A). To define the subcellular context of this interaction, we performed subcellular fractionation followed by IDO1 Co-IP in TBD0220 and LN229-EGFRvIII cells. TEFT enhanced the CPT1A-IDO1 interaction, and the complex was enriched in the mitochondrial fraction, with ATP5A and Calnexin used to assess fraction quality (Fig. 4B)**.** Given that CPT1A is an established outer mitochondrial membrane-resident enzyme, these findings support recruitment of IDO1 to a CPT1A-associated OMM interface rather than import of IDO1 into the mitochondrial matrix. Additional Co-IP assays in EGFRvIII-driven models, including patient-derived LG-260 cells, showed that TEFT increased IDO1-CPT1A association while reducing IDO1-TRIM21 association (fig. S8A, B). Structural modeling using AlphaFold3 predicted a CPT1A-IDO1 docking interface near the IDO1 regulatory region containing the K377 succinylation site and the adjacent K389 ubiquitination-associated degron (Fig. 4C).

CPT1A possesses carnitine palmitoyl transferase (CPTase) activity and has also been reported to exhibit non-canonical lysine succinyltransferase-related (LSTase) activity(*31*, *32*). We next investigated whether CPT1A exerts a post-translational modification on IDO1. Given CPT1A's reported moonlighting function as a lysine succinyltransferase, we performed LC-MS/MS analysis on IDO1. This identified lysine 377 (K377) as a candidate succinylation site and lysine 366/389 (K366/K389) as ubiquitination-associated sites (Fig. 4D and fig. S8C). Functional validation demonstrated that CPT1A overexpression increased IDO1 succinylation (Fig. 4E). Conversely, CPT1A depletion reduced IDO1 succinylation and increased IDO1 ubiquitination, whereas re-expression of wild-type CPT1A restored succinylation and reduced ubiquitination (Fig. 4F). To distinguish this effect of the LSTase-related activity of CPT1A from its canonical carnitine palmitoyl transferase (CPTase) function(*33*, *34*), we utilized specific point mutants: CPT1A-G710E, which retains LSTase activity but lacks CPTase activity, and CPT1A-H473A, a catalytically inactive mutant. In CPT1A-depleted cells, re-expression of wild-type CPT1A or the G710E mutant restored IDO1 succinylation and reduced IDO1 ubiquitination, whereas the H473A mutant failed to do so (Fig. 4F). These mutant-rescue data support the requirement of CPT1A LSTase-related activity, rather than classical CPTase activity alone, for the post-translational regulation of IDO1.

To obtain direct biochemical evidence for CPT1A-mediated IDO1 succinylation, we performed cell-free assays using purified recombinant proteins. CPT1A-WT promoted IDO1 succinylation in the presence of succinyl-CoA, whereas the H473A mutant showed reduced activity toward IDO1 (fig. S8D). IDO1 succinylation also increased with increasing succinyl-CoA concentrations in the presence of CPT1A (fig. S8E). Moreover, TEFT did not significantly increase total cellular succinyl-CoA concentrations in TBD0220, LN229-EGFRvIII, or T98G-EGFRvIII cells, arguing against a mechanism driven solely by a global increase in succinyl-CoA availability or nonspecific non-enzymatic succinylation (fig. S8F).

To determine the precise mechanism of stabilization, we generated IDO1 lysine mutants. Cycloheximide (CHX) chase assays revealed that the IDO1-K389R mutant exhibited a significantly extended half-life compared with wild-type IDO1, implicating K389 as an important ubiquitination-associated degron that contributes to IDO1 turnover (Fig. 4G). Consistent with this, the K389R mutation reduced IDO1 ubiquitination (fig. S8G). Evolutionary analysis showed that the K366, K377, and K389 sites are highly conserved across species, supporting the potential functional importance of this regulatory region (fig. S8H). Finally, we verified this axis in the context of TEFT. In TBD0220 and LN229-EGFRvIII cells, TEFT treatment increased IDO1 succinylation and reduced its ubiquitination; importantly, CPT1A knockdown attenuated this TEFT-induced post-translational switch (Fig. 4H). Consistent results were obtained in T98G-EGFRvIII and patient-derived EGFR-driven cells (fig. S8I-L). Thus, TEFT promotes a CPT1A-dependent shift in IDO1 post-translational regulation from ubiquitination toward succinylation, thereby supporting IDO1 stabilization.

To visually corroborate this interaction in situ, we performed immunofluorescence colocalization assays. High-resolution three-dimensional z-stack imaging revealed that TEFT increased the overlap between IDO1 and CPT1A-positive structures in TBD0220 and LN229-EGFRvIII cells (Fig. 4I and fig. S9A). Conventional confocal imaging and line-scan analysis in TBD0220, LN229-EGFRvIII, and T98G-EGFRvIII cells provided additional evidence of TEFT-enhanced spatial proximity between IDO1 and CPT1A-positive mitochondrial structures (fig. S9B-C). Together with the mitochondrial fractionation Co-IP data, these imaging results support recruitment of IDO1 to a CPT1A-associated OMM interface. Collectively, these data support a model in which TEFT-induced bioenergetic stress upregulates CPT1A and promotes its interaction with IDO1 at CPT1A-positive mitochondrial structures. CPT1A-dependent succinylation of IDO1 at K377 functionally antagonizes ubiquitination at the adjacent K389 degron, thereby preserving the IDO1 pool for downstream signaling (Fig. 4J).

### Methods, lines 786-800;

Mitochondria were isolated using the Mitochondria Isolation Kit for Cultured Cells (Thermo Scientific) according to the manufacturer's instructions. Briefly, approximately $2 \times 10^7$ cells were lysed using Reagents A and B, followed by differential centrifugation at $700 \times g$ to remove nuclei and cellular debris and at $12{,}000 \times g$ to pellet the mitochondrial fraction. For Western blotting, mitochondrial pellets were lysed in RIPA buffer supplemented with protease and phosphatase inhibitors. The purity of the mitochondrial fraction was assessed by immunoblotting for ATP5A as a mitochondrial marker and for Calnexin and $Na^+/K^+$-ATPase as endoplasmic reticulum and plasma membrane contamination markers,

respectively. Equal amounts of protein from the mitochondrial fractions were used for subsequent immunoprecipitation.

For flow cytometric analysis, intact mitochondrial pellets were resuspended in PBS containing 2% BSA, fixed and permeabilized, and subsequently stained with antibodies against ATP5A, Calnexin, IDO1, and CPT1A. The mitochondrial population was identified as ATP5A-positive and Calnexin-negative, with unstained and single-stained controls used to define gating and fluorescence compensation. IDO1 and CPT1A signals were quantified within the gated mitochondrial population.

3. Clinical claims are overinterpreted. The case series is very small (n = 4), and biomarker-driven benefit is not supported. The manuscript frames resistance as EGFRvIII-specific, yet the vignettes include other EGFR alterations and at least one apparent responder without EGFRvIII (Fig. 6G–H), raising whether the phenotype reflects EGFRvIII or broader EGFR activation. Please clarify the biomarker hypothesis and align the framing (including the title); otherwise temper conclusions and present the clinical data as hypothesis-generating or expand the cohort.

**Response:** We agree that the original clinical interpretation exceeded the evidentiary strength of the available data. The four clinical vignettes—and particularly the two patients who received compassionate-use osimertinib plus TEFT—cannot establish clinical efficacy or validate a predictive biomarker.

We have therefore separated the mechanistic and clinical hypotheses more clearly. At the mechanistic level, the experimental evidence is centered on EGFRvIII. EGFRvIII provides constitutive NF-κB-dependent transcriptional priming of IDO1, which converges with the broadly inducible TEFT-AMPK-PPARα-CPT1A response. The title accordingly limits the principal mechanistic claim to **EGFRvIII-driven resistance** rather than implying that all EGFR alterations engage an identical mechanism.

At the clinical level, the observations support only a broader and exploratory EGFR-pathway hypothesis. Case #3 harbored EGFR amplification and an EGFR p.E709K alteration but was EGFRvIII-negative, whereas Case #4 harbored both EGFR amplification and EGFRvIII. The observations in these cases therefore cannot be used to claim an EGFRvIII-specific clinical benefit. We now describe them as preliminary, mechanism-informed observations in recurrent **EGFR-driven or EGFR-altered GBM**. The retrospective association between EGFRvIII and shorter progression-free survival in the 31-patient cohort is also explicitly described as hypothesis-generating rather than evidence that EGFRvIII independently predicts TEFT resistance. The revised manuscript states that the available data cannot determine whether EGFRvIII, EGFR amplification, another activating EGFR alteration, or a broader measure of EGFR-pathway activity will ultimately prove predictive.

We have accordingly removed language implying biomarker validation or demonstrated clinical efficacy. The Abstract, Results, and Discussion now consistently describe the clinical observations as exploratory and requiring prospective biomarker-guided validation.

**Changes in the revised manuscript:**
**Introduction, lines 59–67;**

Through a retrospective analysis of a single-center clinical cohort, we observed that patients harboring EGFRvIII alterations had shorter progression-free survival compared with their EGFRvIII-wild-type counterparts when treated with electric field therapy. Although this observation is limited by the retrospective design and modest cohort size, it is consistent with emerging clinical evidence suggesting that EGFR-related alterations may influence the therapeutic benefit of electric field therapy, including reports that EGFR wild-type status or EGFR amplification may stratify progression-free survival in TEFT-treated GBM patients(*19*). These clinical observations motivated us to investigate whether and how EGFRvIII mechanistically contributes to TEFT resistance in GBM.

**Results, lines 92–104, Subtitle and lines 415–427;**

Since 2021, the Department of Neurosurgery at Qilu Hospital of Shandong University has established a real-world "TEFT-GBM" cohort. We consecutively and retrospectively enrolled patients with histologically confirmed glioblastoma who received TEFT after standard Stupp protocol. By the data-cutoff date, 31 patients with complete clinical and molecular profiles were included (median age 59 years, range 28-75; 16 males and 15 females). All patients received TEFT at a field intensity of 2 V $cm^{-1}$ and a frequency of 200 kHz, with treatment compliance of ≥18 h $day^{-1}$. Central neuropathology review identified EGFRvIII alterations in 9 cases (29.0%). Survival analysis showed that the EGFRvIII-altered group had a shorter median progression-free survival than the EGFRvIII-wild-type group (12.97 versus 16.60 months; HR, 3.144; 95% CI, 1.136-8.700; P = 0.0482) (Fig. 1A, B). Given the retrospective nature and limited size of this cohort, this association should be interpreted as hypothesis-generating rather than definitive evidence that EGFRvIII independently predicts TEFT resistance. Nevertheless, these clinical findings warranted further mechanistic investigation. (Results, lines 92–104)

**EGFR-TKI enhances TEFT efficacy in EGFR-driven GBM models and exploratory clinical cases** (Subtitle)

Motivated by these preclinical and ex vivo data, we evaluated exploratory clinical cases involving GBM patients receiving standard Stupp protocol chemoradiotherapy combined with TEFT. The patient with EGFR-wild-type disease maintained durable disease stability, whereas patients harboring EGFR amplification with or without EGFRvIII experienced earlier progression (Fig. 6G). Although these observations were based on a small number of clinically heterogeneous cases, they were consistent with the hypothesis that aberrant EGFR signaling may reduce TEFT responsiveness. Based on this mechanistic rationale, two patients with recurrent EGFR-driven GBM subsequently received osimertinib combined with TEFT as salvage treatment. Case #3, harboring EGFR amplification, presented with recurrence and severe mass effect and showed stable disease with radiological improvement and reduced mass effect after initiation of the combination regimen. Similarly, Case #4, harboring EGFR amplification with EGFRvIII, experienced rapid recurrence following repeat resection but achieved radiological disease stabilization after receiving the salvage combination therapy (Fig. 6G, H). (Results, lines 415–427)

**Discussion, lines 541–567;**

This strategy exploits complementary mechanisms: TEFT exerts physical pressure on the mitotic spindle, whereas osimertinib prevents EGFR-driven tumor cells from efficiently converting metabolic adaptation into IDO1-AhR-dependent cytoskeletal protection. Based on this rationale, our preliminary clinical observations in Cases #3 and #4 provide mechanism-informed proof-of-concept support that osimertinib may enhance TEFT responsiveness in recurrent EGFR-driven GBM. However, these observations remain exploratory because of the small number of patients and treatment heterogeneity and therefore require prospective biomarker-guided validation.

Our study has limitations that point toward critical avenues for future research. First, the association between EGFRvIII status and reduced TEFT benefit was derived from a retrospective single-center cohort, and the clinical observations involving osimertinib plus TEFT were based on a small exploratory case series. These findings are hypothesis-generating and mechanism-informed but cannot establish EGFR alterations as definitive predictive biomarkers or demonstrate clinical efficacy of the combination. Larger prospective, biomarker-guided studies are required. Second, although TEFT-induced mitochondrial stress and AMPK-PPARα-CPT1A activation were observed in both EGFRvIII-driven and parental GBM cells, it remains unknown whether this metabolic program becomes a general mechanism of acquired resistance across molecular subtypes. Longitudinal TEFT exposure models and pan-genotypic patient-derived systems will be needed to determine how broadly this adaptive response applies and whether CPT1A represents a shared therapeutic vulnerability. Third, although we mapped the CPT1A LSTase-related activity, the precise source and submitochondrial accessibility of the succinyl-CoA donor require further biochemical dissection. Although total cellular succinyl-CoA was not depleted under TEFT, direct submitochondrial metabolite tracing will be necessary to test the proposed CPT1A-associated outer mitochondrial membrane microdomain model. Finally, the safety and efficacy of TEFT combined with osimertinib require prospective clinical evaluation, including optimization of osimertinib dose, treatment timing, tolerability, and molecular patient selection.

4. If the translational goal is combination therapy, it would be valuable to define the minimum effective osimertinib dose required for resensitization under TEFT (in vitro and ideally in vivo), rather than relying on a single dose. This is especially relevant for tolerability in a TEFT-treated population.

**Response:** We agree that the translational value of osimertinib in this setting depends on whether TEFT sensitization can be achieved at an exposure with limited single-agent activity.

We therefore performed serial osimertinib dose-response experiments in TBD0220, LN229-EGFRvIII, and T98G-EGFRvIII cells in the presence or absence of TEFT. Across all three models, TEFT shifted the osimertinib dose-response curves toward lower concentrations, showing that less osimertinib was required to achieve a comparable inhibitory effect during electric-field exposure. IC50 values were calculated separately under control and TEFT conditions. The relevant changes made in the results have been added to fig. S1F, G.

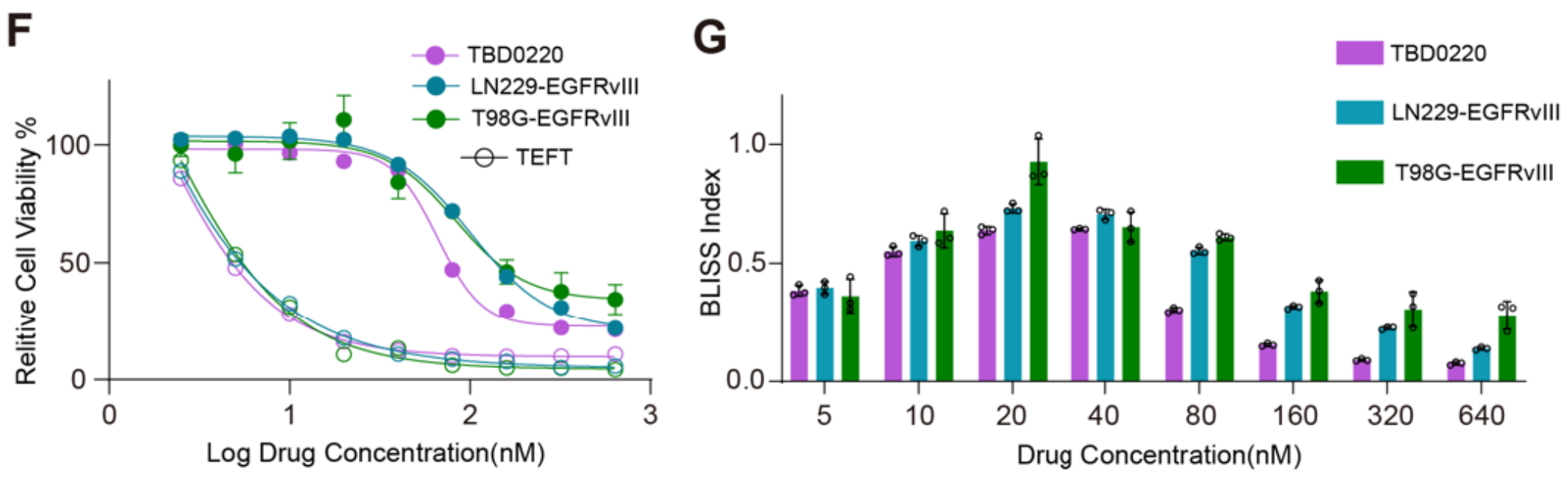


**fig. S1 (F)** Dose-response viability curves of TBD0220, LN229-EGFRvIII, and T98G-EGFRvIII cells treated with increasing concentrations of osimertinib in the presence or absence of TEFT. TEFT shifted the osimertinib dose-response curves toward lower concentrations, indicating enhanced drug sensitivity under TEFT exposure, **(G)** Bliss synergy analysis of osimertinib and TEFT in TBD0220, LN229-EGFRvIII, and T98G-EGFRvIII cells across the indicated osimertinib concentrations. Positive Bliss index values support a synergistic interaction between osimertinib and TEFT.

For the orthotopic experiments, osimertinib was administered at 5 mg/kg once daily. At this dose, osimertinib monotherapy produced only modest tumor control, whereas the same dose combined with TEFT more effectively reduced tumor burden and prolonged survival in both the TBD0220 and LN229-EGFRvIII models. These findings support a sensitizing effect at a dose with limited single-agent efficacy.

We have nevertheless avoided describing 5 mg/kg as a formally established minimum effective in vivo dose, because a complete in vivo dose-de-escalation series was not performed. The revised manuscript instead describes it as a low-dose, mechanism-based sensitizing regimen and separates this preclinical observation from clinical dose optimization.

**Other Changes in the revised manuscript are provided below.**

**Results, lines 111–114 and 391–398;**

Dose-response analysis further showed that TEFT shifted the osimertinib response curve toward lower concentrations, and Bliss analysis supported a synergistic interaction between osimertinib and TEFT in EGFRvIII-driven models (fig. S1F, G). (Results, lines 111–114)

To translate our mechanistic findings into a therapeutic strategy, we first evaluated the efficacy of combining TEFT with the third-generation EGFR-TKI osimertinib in orthotopic EGFRvIII-driven GBM models. In the TBD0220 orthotopic model, bioluminescence imaging revealed that while TEFT or osimertinib monotherapy only modestly inhibited tumor growth, the combination reduced tumor burden more effectively than either treatment alone (Fig. 6A, B). This enhanced tumor control translated into a significant survival benefit, with the combination group exhibiting extended overall survival compared with either monotherapy (Fig. 6C). A consistent therapeutic pattern was observed in an additional LN229-EGFRvIII orthotopic model (fig. S12A-C). (Results, lines 391-398)

**Methods, lines 648–657;**

Dose–response curves were generated using nonlinear regression, and the half-maximal inhibitory concentration (IC50) of osimertinib was calculated separately under control and TEFT conditions using GraphPad Prism software.

Drug-TEFT interactions were further evaluated using the Bliss independence model. For each combination condition, fractional inhibition was calculated from normalized cell viability. The expected Bliss inhibition was calculated as *E(Bliss) = EA+EB-EAEB*, where *EA* and *EB* represent the fractional inhibition produced by osimertinib and TEFT alone, respectively. The Bliss synergy score was calculated as the observed combination inhibition minus the expected Bliss inhibition. Positive scores indicated synergy, scores close to zero indicated an additive interaction, and negative scores indicated antagonism.

5. Synergy should be quantified (e.g., Bliss/Loewe) rather than described qualitatively, particularly for the core claim that osimertinib "abrogates" resistance.

**Response:** We agree that the interaction between osimertinib and TEFT should be formally quantified rather than inferred from qualitative comparisons.

We therefore performed Bliss independence analysis using the serial osimertinib dose-response data generated in TBD0220, LN229-EGFRvIII, and T98G-EGFRvIII cells. For each combination condition, the expected Bliss inhibition was calculated as:

*EBliss = EA + EB − EAEB*

where EA and EB represent the fractional inhibition produced by osimertinib and TEFT alone, respectively. The Bliss synergy score was defined as the observed combination inhibition minus the expected Bliss inhibition.

Positive Bliss scores were observed across the active osimertinib concentration range in all three EGFRvIII-driven models, supporting a synergistic interaction between osimertinib and TEFT. This quantitative analysis complements the leftward shift in the osimertinib dose-response curves and the enhanced activity of the combination in the orthotopic models.

We also replaced the term "abrogates resistance" with more precise language, including "enhances TEFT efficacy," "restores TEFT sensitivity," or "disrupts the EGFRvIII-dependent resistance program."

**Changes in the revised manuscript:**
Results, lines 111–114; Methods, lines 651-657; figs. S1F, G.

6. Given IDO1 is positioned as the key effector, the translational logic would be strengthened by showing that pharmacologic IDO1 inhibition (and genetic approaches in multiple patient-derived models) can phenocopy osimertinib-mediated resensitization under TEFT. This would also help distinguish whether osimertinib is acting strictly upstream via EGFR signaling versus through additional effects induced in vivo.

**Response:** We agree that direct perturbation of IDO1 is necessary to determine whether it is a functional downstream effector of EGFRvIII-mediated TEFT resistance rather than a correlative biomarker.

We first used two independent IDO1-targeting siRNAs in the patient-derived EGFRvIII-positive TBD0220 model and in LN229-EGFRvIII and T98G-EGFRvIII cells. IDO1 depletion increased TEFT-induced apoptosis and restored TEFT-induced G2/M accumulation and mitotic perturbation. The results are shown in Figs. 1K, L and figs. S2E-J.

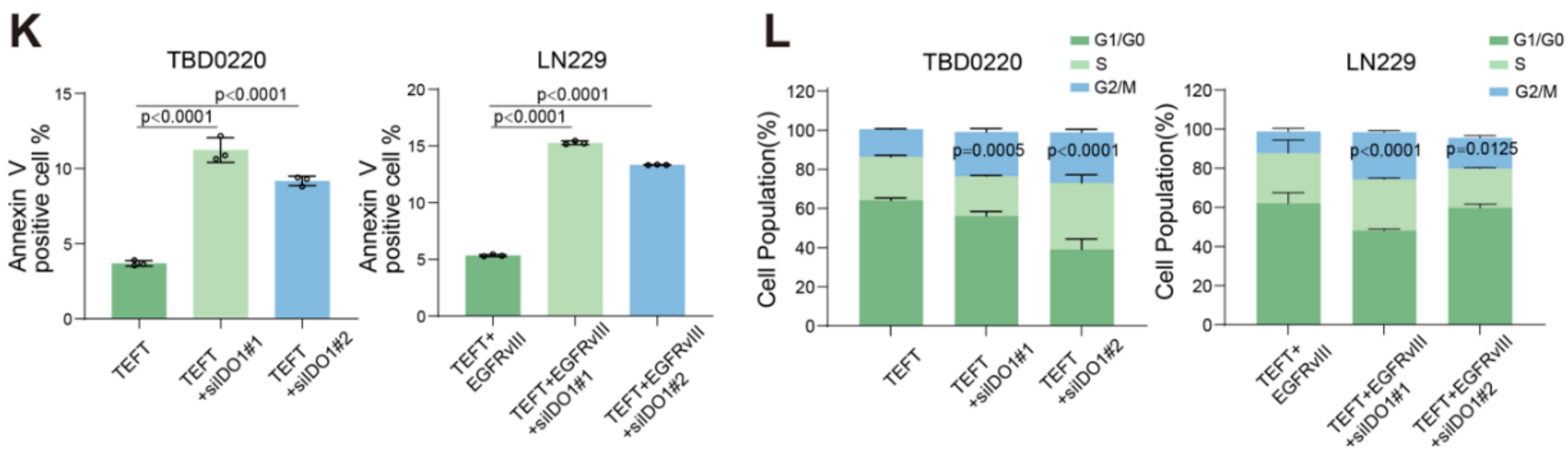


**Fig. 1 (K)** Rescue effects of IDO1 knockdown (siRNA) on apoptosis in TEFT-treated EGFRvIII-driven TBD0220 and LN229 cells, **(L)** Cell cycle distribution analysis showing that IDO1 knockdown restores sensitivity to TEFT-induced mitotic perturbation (G2/M arrest) in EGFRvIII cells.

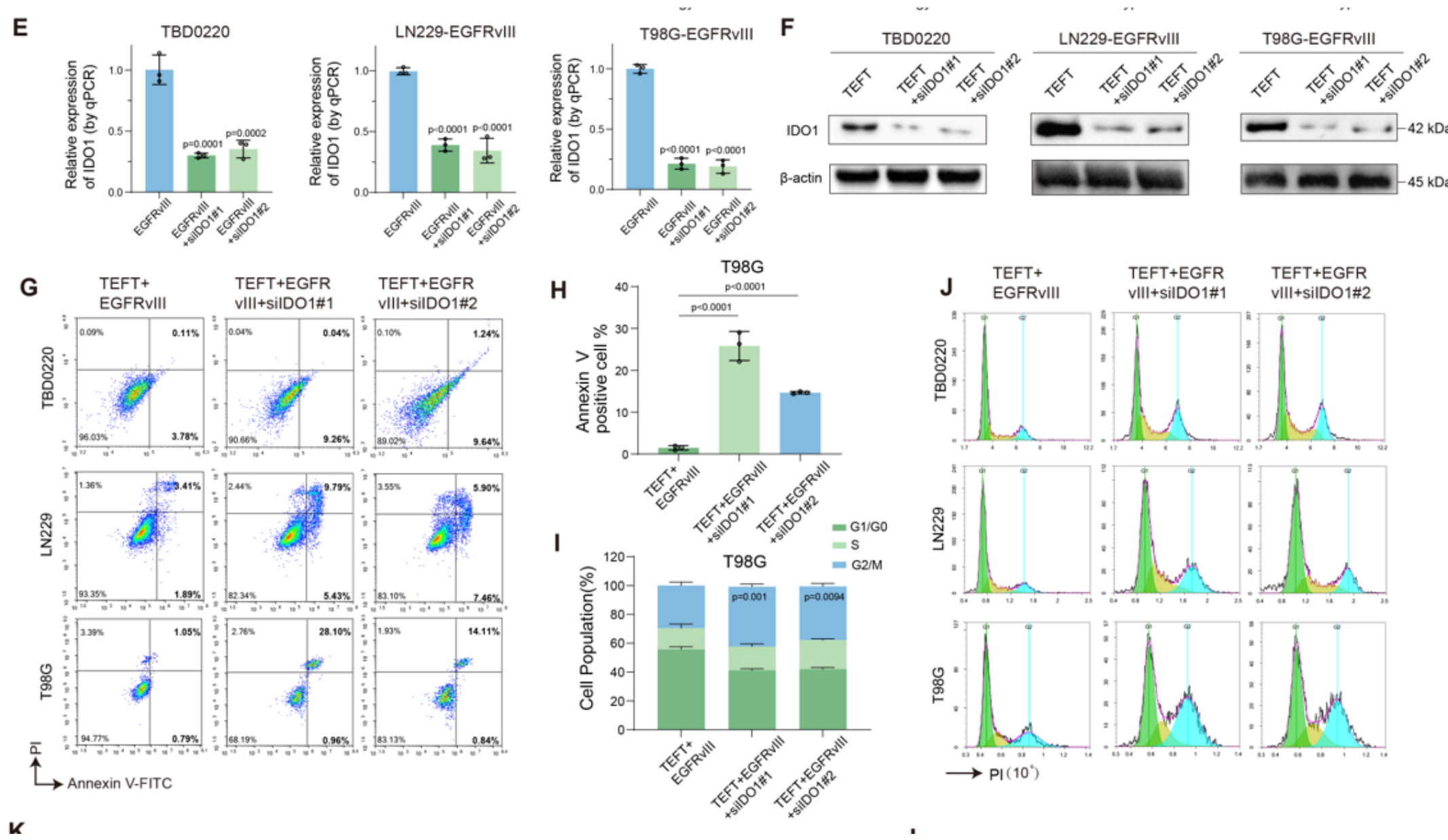


**fig. S2 (E)** RT-qPCR validation of IDO1 knockdown efficiency in TBD0220, and EGFRvIII-overexpressing LN229 and T98G cells transfected with two independent siRNAs (siIDO1#1, siIDO1#2), **(F)** Western blot validation of IDO1 knockdown efficiency at the protein level in TBD0220, LN229 and T98G cells, **(G)** Representative flow cytometry plots of Annexin V/PI staining showing that IDO1 knockdown sensitizes EGFRvIII-driven cells to TEFT-induced apoptosis, **(H)** Quantification of Annexin V-positive T98G-EGFRvIII cells after TEFT treatment with or without IDO1 knockdown, **(I)** Quantification of cell cycle distribution in T98G-EGFRvIII cells after TEFT treatment with or without

IDO1 knockdown, **(J)** Representative flow cytometry histograms of cell cycle analysis showing that IDO1 knockdown restores TEFT-induced G2/M accumulation in EGFRvIII-driven cells.

We next inhibited IDO1 catalytic activity using epacadostat. Epacadostat enhanced TEFT-induced apoptosis in TBD0220, LN229-EGFRvIII, and T98G-EGFRvIII cells. Importantly, this effect was reproduced in the independent low-passage EGFR-amplified/EGFRvIII-positive LG-260 model, in which epacadostat phenocopied the apoptosis-enhancing effect of osimertinib during TEFT exposure. The relevant changes made in the results have been added to S3A-H.

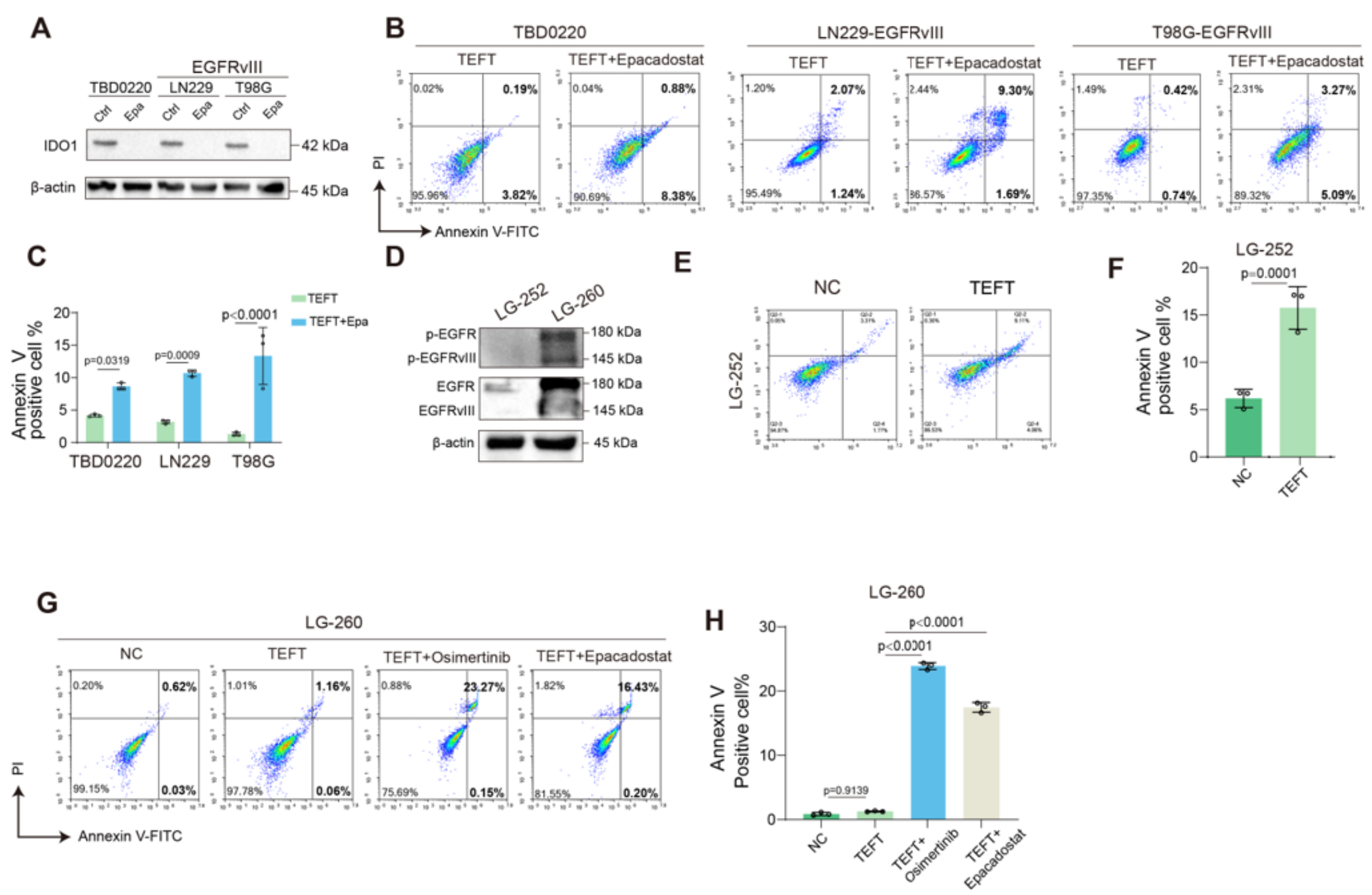


**fig. S3** **(A)** Western blot analysis of IDO1 protein levels in TBD0220, LN229-EGFRvIII, and T98G-EGFRvIII cells treated with vehicle or epacadostat, **(B)** Representative Annexin V/PI flow cytometry plots showing that epacadostat increases TEFT-induced apoptosis in TBD0220, LN229-EGFRvIII, and T98G-EGFRvIII cells, **(C)** Quantification of Annexin V-positive cells from panel B, **(D)** Western blot analysis of EGFR/EGFRvIII phosphorylation and total EGFR/EGFRvIII expression in patient-derived LG-252 and LG-260 cells. β-actin served as a loading control, **(E and F)** Representative Annexin V/PI flow cytometry plots and quantification showing that patient-derived LG-252 cells are sensitive to TEFT-induced apoptosis, **(G and H)** Representative Annexin V/PI flow cytometry plots and quantification in patient-derived LG-260 cells under the indicated conditions. Osimertinib or epacadostat enhanced apoptosis under TEFT treatment.

Direct IDO1 inhibition also disrupted the spindle-protective phenotype and reduced the enhanced microtubule-polymerizing activity associated with the resistant state. These findings connect IDO1 enzymatic activity to both cell survival and downstream cytoskeletal protection. The relevant changes made in the results have been added to Fig. 5E, L and S10D-J.

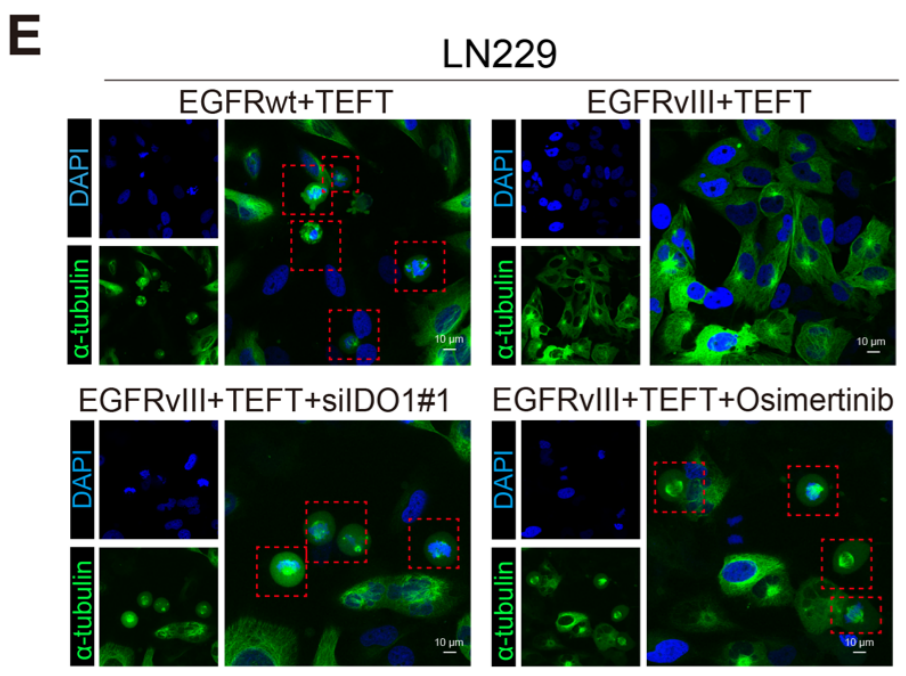


Fig. 5 (E) Representative immunofluorescence images of mitotic spindles (α-tubulin, green) and chromosomes (DAPI, blue) in LN229 cells. TEFT causes severe spindle disruption and multipolar mitosis in wild-type cells, whereas EGFRvIII cells maintain bipolar spindles. This protection is lost upon IDO1 knockdown or osimertinib treatment.

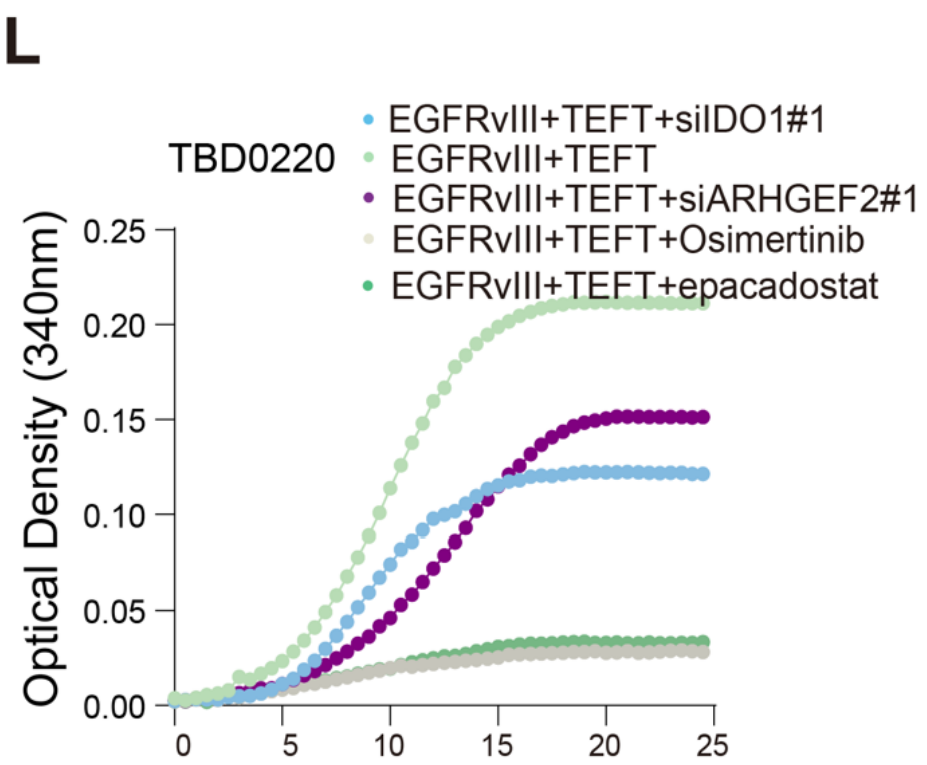


Fig. 5 (L) In vitro tubulin polymerization assay in TBD0220 cells under the indicated conditions. IDO1 knockdown, ARHGEF2 knockdown, osimertinib, or epacadostat reduced the microtubule-polymerizing activity associated with EGFRvIII-mediated TEFT resistance.

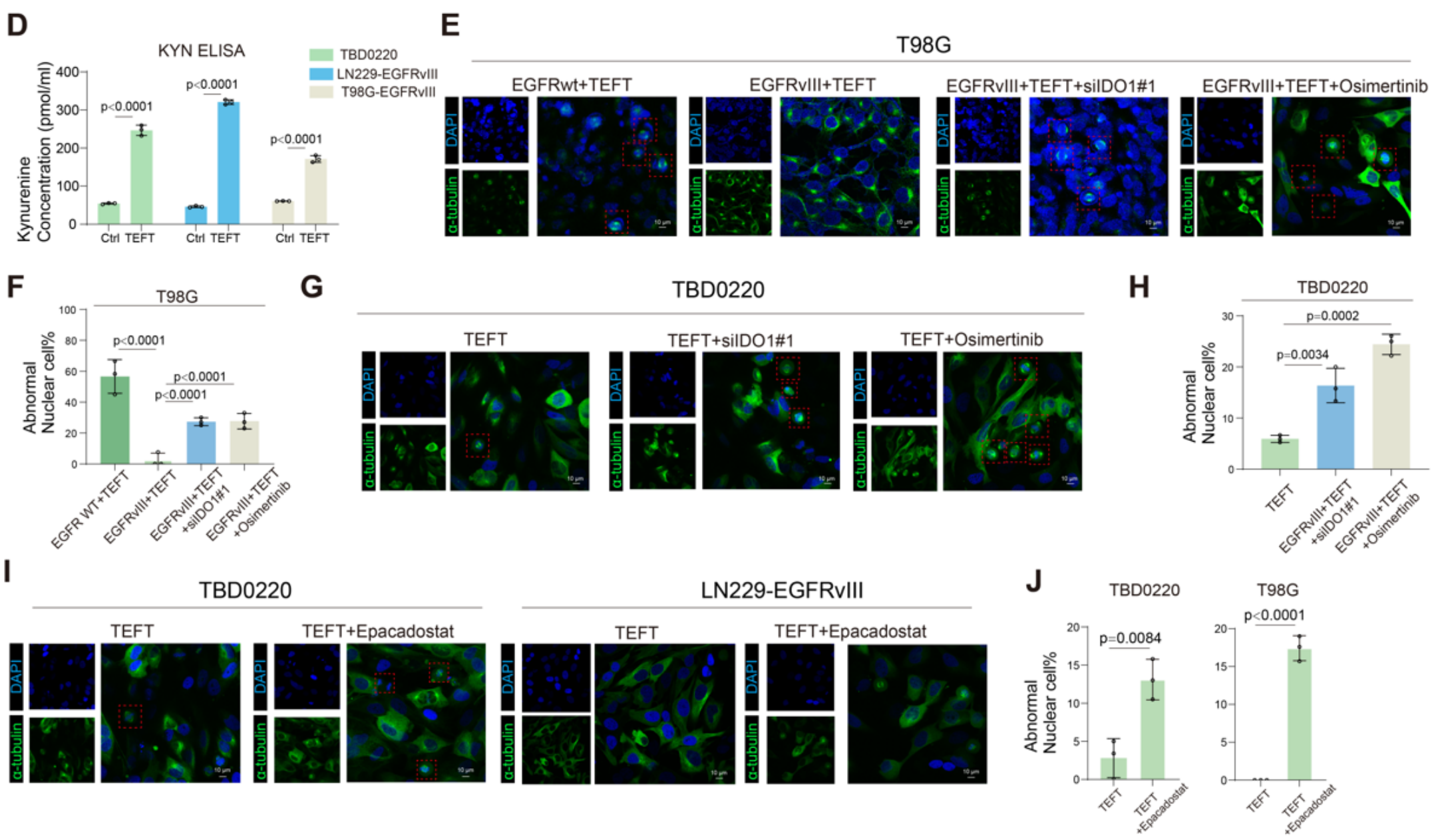

**fig. S10(D)** Kynurenine ELISA showing increased extracellular kynurenine levels in the culture supernatants of TBD0220, LN229-EGFRvIII, and T98G-EGFRvIII cells after TEFT treatment, (**E**) Representative immunofluorescence images of α-tubulin staining in T98G cells under the indicated conditions. TEFT induced spindle and nuclear abnormalities in EGFR wild-type cells, whereas EGFRvIII cells maintained a more preserved microtubule architecture under TEFT. This protective phenotype was disrupted by IDO1 knockdown or osimertinib treatment. DAPI, nuclei; α-tubulin, green. Scale bars, 10 μm, (**F**) Quantification of cells with abnormal nuclear morphology in T98G cells under the indicated conditions, (**G**) Representative immunofluorescence images of α-tubulin staining in TBD0220 cells treated with TEFT, TEFT plus IDO1 knockdown, or TEFT plus osimertinib. IDO1 knockdown or osimertinib disrupted the TEFT-resistant spindle-protective phenotype. DAPI, nuclei; α-tubulin, green. Scale bars, 10 μm, (**H**) Quantification of cells with abnormal nuclear morphology in TBD0220 cells under the indicated conditions, (**I**) Representative immunofluorescence images of α-tubulin staining in TBD0220 and LN229-EGFRvIII cells treated with TEFT alone or TEFT plus epacadostat. Pharmacological IDO1 inhibition disrupted the spindle-protective phenotype under TEFT exposure. DAPI, nuclei; α-tubulin, green. Scale bars, 10 μm, (**J**) Quantification of cells with abnormal nuclear morphology in TBD0220 and T98G cells after TEFT treatment with or without epacadostat.

Together, the genetic and pharmacological data show that IDO1 mediates a substantial component of osimertinib-induced TEFT resensitization. They support the proposed placement of osimertinib upstream of the NF-κB–IDO1 transcriptional supply arm, although they do not exclude additional IDO1-independent effects of EGFR inhibition.

**Other Changes in the revised manuscript are provided below.**

**Results, lines 130–156 and 354–358;**

To determine whether IDO1 is a functional mediator of EGFRvIII-driven resistance rather than a bystander, we performed loss-of-function assays. We first validated the knockdown efficiency of two independent small interfering RNAs in TBD0220, LN229-EGFRvIII, and T98G-EGFRvIII cells, confirming robust depletion at both the mRNA and protein levels (fig. S2E, F). We then assessed the impact of IDO1 silencing on cell survival under electric field exposure. Flow cytometric analysis of apoptosis showed that while EGFRvIII expression reduced TEFT-induced cell death, silencing IDO1 attenuated this protective phenotype, resulting in a marked increase in apoptosis following TEFT treatment (Fig. 1K and fig. S2G, H). Consistently, cell-cycle analysis revealed that IDO1 knockdown resensitized EGFRvIII-driven cells to TEFT-induced mitotic perturbation. Whereas EGFRvIII-driven cells displayed less pronounced TEFT-induced G2/M accumulation, IDO1-depleted cells exhibited restored G2/M accumulation and mitotic arrest (Fig. 1L and fig. S2I-L). These data support a functional requirement for IDO1 in sustaining the TEFT-resistant phenotype in EGFRvIII-driven GBM cells.

To complement these genetic loss-of-function data, we next tested whether pharmacological inhibition of IDO1 catalytic activity could similarly restore TEFT sensitivity. Epacadostat did not appreciably alter total IDO1 protein abundance (fig. S3A), but significantly increased TEFT-induced apoptosis in TBD0220, LN229-EGFRvIII, and T98G-EGFRvIII cells (fig. S3B, C). We then extended this analysis to independent low-passage patient-derived GBM cultures. Molecular characterization showed that LG-260 exhibited EGFR amplification and EGFRvIII expression, whereas LG-252 lacked the corresponding EGFRvIII-driven signaling profile (fig. S3D). LG-252 cells remained sensitive to TEFT-induced

apoptosis (fig. S3E, F). In contrast, LG-260 cells showed a limited apoptotic response to TEFT alone, whereas either osimertinib or epacadostat markedly increased TEFT-induced apoptosis (fig. S3G, H). Thus, direct pharmacological inhibition of IDO1 enzymatic activity phenocopied the resensitizing effect of upstream EGFR blockade in an independent patient-derived EGFR-driven model. Together with the genetic depletion experiments, these findings establish IDO1 as a functional downstream effector of EGFR-driven TEFT resistance. (Results, lines 130–156)

Similar spindle-disruptive effects of IDO1 knockdown, osimertinib, or the IDO1 inhibitor epacadostat were observed in T98G-EGFRvIII, TBD0220, and LN229-EGFRvIII cells (fig. S10E-J). Together, these findings support an IDO1-kynurenine-AhR cascade that contributes to mitotic and microtubule protection during TEFT resistance. (Results, lines 354–358)

7. Minor comments:

1. The manuscript is dense and would benefit from tighter writing and clearer subheading of the main causal chain. A figure summarizing the model would be very helpful for the readers to follow.

**Response:** We agree and have substantially streamlined the manuscript by removing repetitive explanations, shortening transitional passages, and reducing overlap between the Results and Discussion.The Results have been reorganized using six sequential, mechanism-oriented subheadings:

1. EGFRvIII confers resistance to tumor electric fields through IDO1 upregulation
2. EGFRvIII up-regulates IDO1 transcription in an NF-κB-dependent manner
3. TEFT-induced bioenergetic stress activates a broad AMPK–PPARα–CPT1A response and promotes mitochondrial enrichment of IDO1
4. CPT1A stabilizes IDO1 via LSTase activity to antagonize ubiquitination
5. IDO1–AhR axis drives TEFT resistance by rewiring mitotic progression
6. EGFR-TKI enhances TEFT efficacy in EGFR-driven GBM models and exploratory clinical cases

We also added an integrated schematic in Fig. 7A. The model distinguishes the broadly inducible TEFT–AMPK–PPARα–CPT1A response from EGFRvIII-dependent NF-κB–IDO1 transcriptional priming and shows how their convergence supports IDO1–kynurenine–AhR–DCLK1/ARHGEF2-mediated cytoskeletal protection. It also indicates where osimertinib interrupts the EGFR-dependent IDO1 supply arm.

**Changes in the revised manuscript:**
Results, lines 91–440; Discussion, lines 442–566; Fig. 7A.

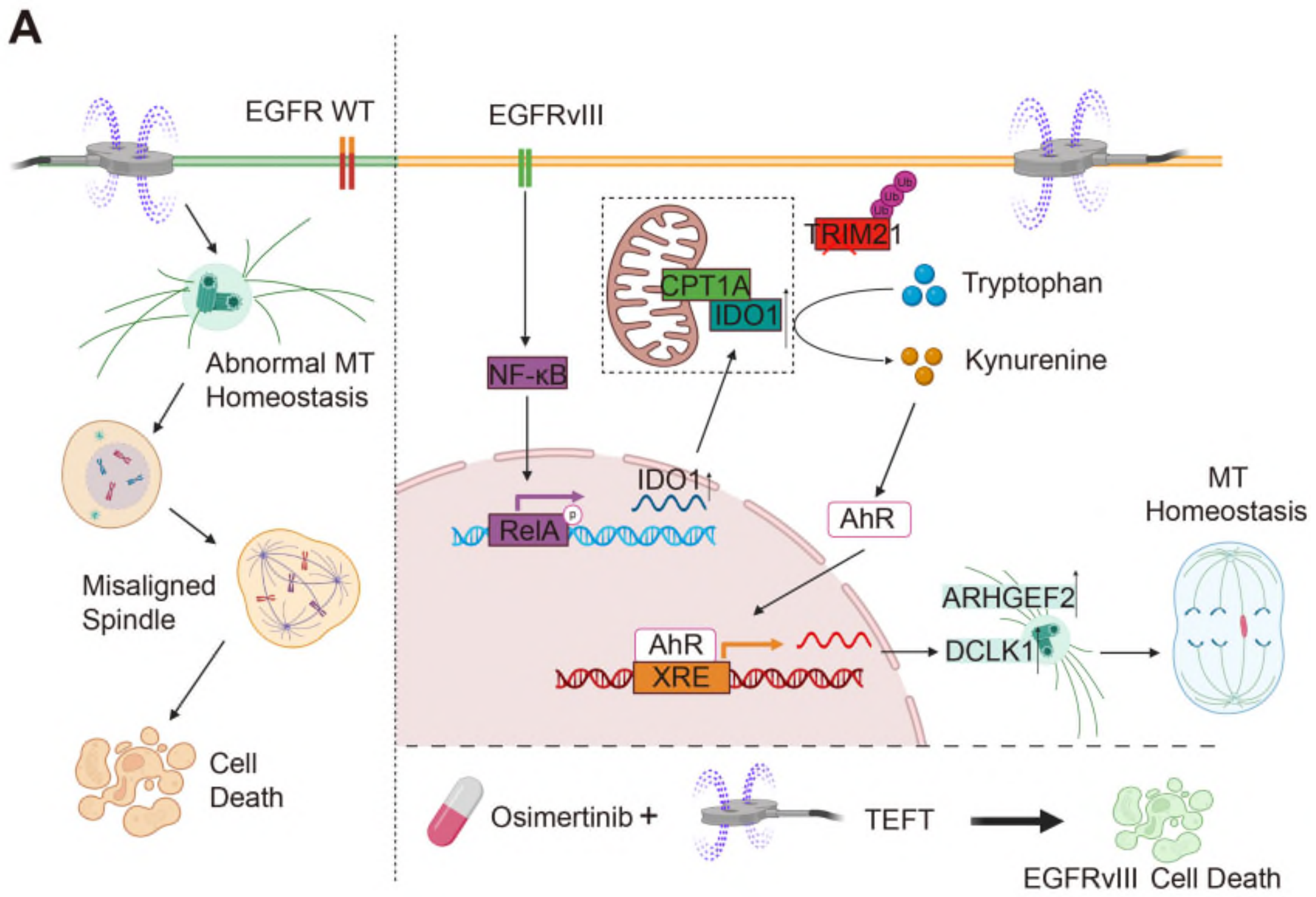


**Schematic model of the IDO1-AhR mediated cytoskeletal resistance program.**

**Fig. 7 (A)** In EGFR wild-type cells, TEFT disrupts microtubule homeostasis, leading tospindle misalignment and cell death. In EGFRvIII cells, NF-κB-dependent IDO1 transcription and TEFT-induced CPT1A-mediated IDO1 stabilization increase kynurenine production, activate AhR-dependent transcription, and upregulate cytoskeletal regulators such as DCLK1 and ARHGEF2, thereby preserving microtubule homeostasis and promoting TEFT resistance. Osimertinib blocks the upstream EGFRvIII-NFκB-IDO1 supply axis and resensitizes EGFRvIII driven cells to TEFT.

[Editorial note: Created in BioRender. Feng, B. (2026) https://BioRender.com/fvoy6ve]

2.Please ensure TEFT settings (frequency/intensity/exposure duration) are consistently reported across figures and methods, and that comparisons across experiments use matched parameters.

**Response:** We systematically reviewed the Results, Methods, and all main and supplementary figure legends to standardize the reporting of TEFT parameters.

For in vitro cell and glioblastoma organoid experiments, TEFT was applied at **1.5 V/cm and 200 kHz for 72 h**, unless otherwise indicated. The culture temperature was maintained at **37°C** using an integrated active temperature-control module.

For orthotopic mouse experiments, TEFT was applied continuously at **2 V/cm and 200 kHz** throughout the treatment period. Electrode arrays were monitored and replaced or repositioned every 2–3 days as necessary.

For the clinical cohort, TEFT was administered at **2 V/cm and 200 kHz**, with treatment

compliance of at least **18 h/day**.

All experimental comparisons within the same platform used matched field intensity, frequency, and exposure duration. The difference between the in vitro and in vivo or clinical field intensities reflects differences in device design and experimental geometry rather than unmatched treatment conditions.

**Changes in the revised manuscript:**
Results, lines 92–98; Methods, lines 570-583, 628–640, and 884–898; all corresponding main and supplementary figure legends.

### Responses to the comments of Reviewer #3

Reviewer 3 Comments to the Author: This study presents a comprehensive and mechanically novel investigation into the resistance mechanisms of EGFRvIII-positive glioblastoma (GBM) against Tumor Electric Field Therapy (TEFT). The authors construct a compelling logical chain linking biophysical stress (TEFT) to metabolic reprogramming (AMPK-PPARa-CPT1A), post-translational modification (IDO1 succinylation), and finally to structural protection of the mitotic spindle (AhR-DCLK1/ARHGEF2). The integration of multi-omics data, biochemical validation, and a prospective clinical case series makes this a robust study. The findings regarding CPT1A's moonlighting LSTase activity and the "metabolic-structural" coupling are particularly interesting. This work is very novel, high interests and well conducted. Written is clear. There are a few gaps regarding the generalization of the metabolic findings and the precise biochemical mechanism could be addressed to further improve the study.

**Response**: We sincerely thank the reviewer for this highly positive, encouraging, and insightful assessment. We are particularly grateful that the reviewer recognized the conceptual importance of linking TEFT-induced metabolic adaptation to post-translational regulation of IDO1 and downstream cytoskeletal protection.

We also agree that the original manuscript required clearer definition of the generality of the metabolic response and stronger spatial and biochemical resolution of the CPT1A–IDO1 mechanism. In response, we substantially expanded the study in four principal areas.

First, experiments in parental EGFRvIII-negative LN229 and T98G cells demonstrated that TEFT-induced mitochondrial depolarization, mitochondrial calcium accumulation, lipid oxidative stress, AMPKα phosphorylation, PPARα–PGC1α engagement, and CPT1A induction are not restricted to EGFRvIII-expressing cells. The revised manuscript therefore describes the AMPK–PPARα–CPT1A program as a broadly inducible metabolic-stress response across the GBM models tested.

Second, we defined the genotype-dependent boundary of this response. EGFRvIII provides NF-κB-dependent transcriptional priming of IDO1, whereas TEFT induces the

metabolic signal that increases CPT1A. The complete IDO1–kynurenine–AhR resistance program emerges when the broadly inducible CPT1A response converges with an EGFRvIII-primed IDO1 pool.

Third, we strengthened the spatial and biochemical definition of the CPT1A–IDO1 mechanism using purified-protein surface plasmon resonance, mitochondrial-fraction co-immunoprecipitation, high-resolution three-dimensional imaging, mutant-rescue experiments, cell-free succinylation assays, and succinyl-CoA measurements.

Fourth, we added functional measurements of IDO1 enzymatic output, mitochondrial-fraction quality controls, thermal control information, and NAC-based redox perturbation experiments.

These revisions support a more clearly bounded genotype–field convergence model while distinguishing experimentally demonstrated findings from remaining topological and biochemical questions. The complete experimental data, statistical analyses, and corresponding interpretation are provided in the revised manuscript.

1. Since TEFT relies on a physical mechanism (dipole disruption), the resulting bioenergetic stress should theoretically be universal. Do EGFRwt cells also exhibit the AMPK-PPARa-CPT1A metabolic shift upon TEFT treatment? The authors should discuss whether this is a pan-GBM response that is specifically exploited by EGVRvIII cells.

**Response:** We agree that the initial mitochondrial and bioenergetic consequences of electric-field exposure should not be intrinsically restricted to EGFRvIII-expressing cells. We therefore performed additional experiments in parental EGFRvIII-negative LN229 and T98G cells.

TEFT induced mitochondrial depolarization in both parental cell lines, as assessed by JC-1 and TMRM staining. Rhod-2 analysis showed mitochondrial calcium accumulation, whereas Liperfluo staining demonstrated increased lipid oxidative stress. These findings show that the initial mitochondrial and redox consequences of TEFT occur independently of EGFRvIII status. The relevant changes made in the results have been added to fig. S5C-G and fig. S7C.

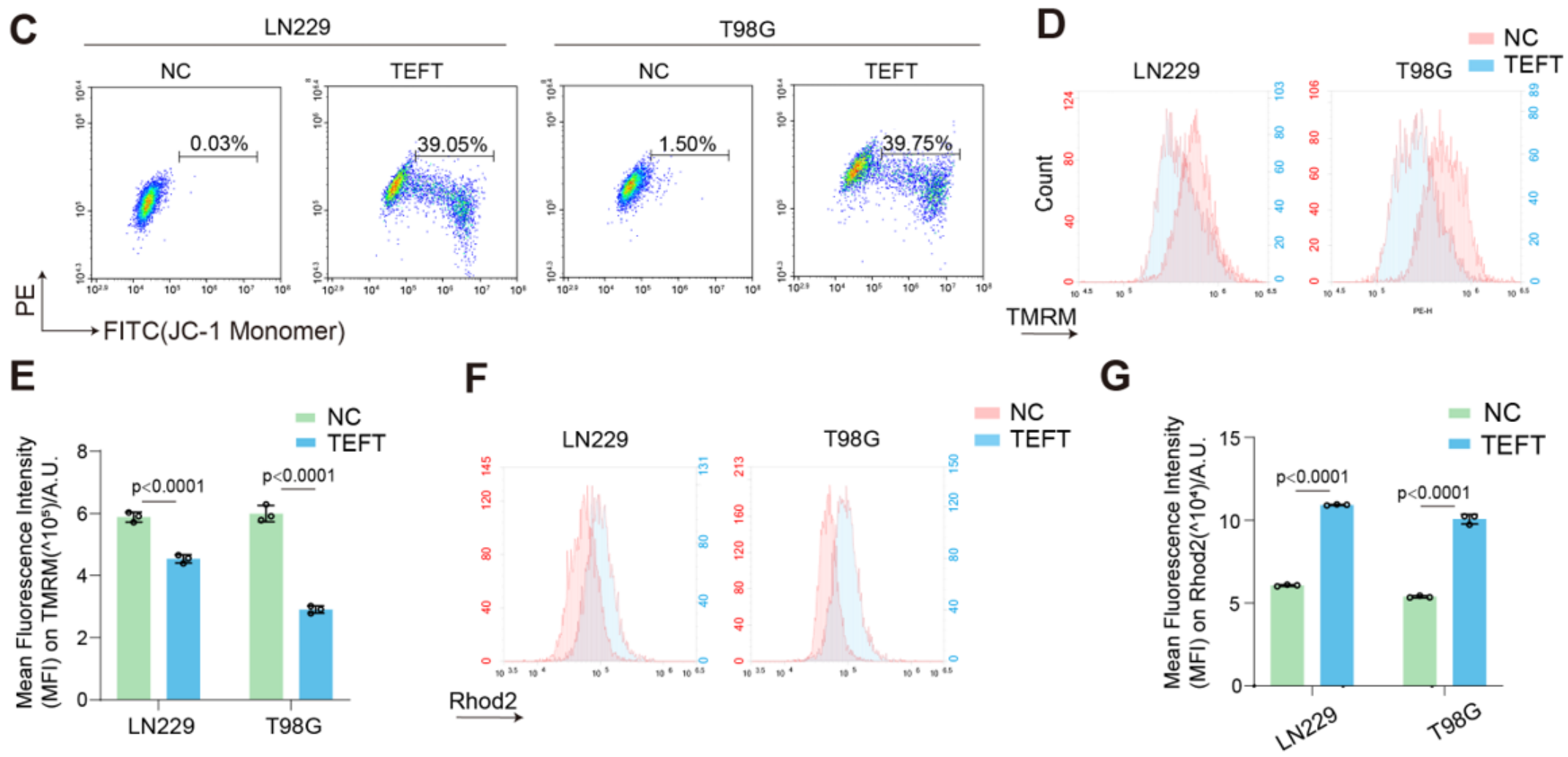


**fig. S5 (C)** Representative flow cytometry dot plots of JC-1 staining in parental LN229 and T98G cells treated with or without TEFT, **(D)** Representative flow cytometry histograms of mitochondrial membrane potential, assessed by TMRM staining, in parental LN229 and T98G cells treated with or without TEFT, **(E)** Quantification of TMRM mean fluorescence intensity in parental LN229 and T98G cells, **(F)** Representative flow cytometry histograms of Rhod-2 staining in parental LN229 and T98G cells treated with or without TEFT, **(G)** Quantification of Rhod-2 mean fluorescence intensity in parental LN229 and T98G cells. Data are presented as mean ± SD. Exact P values are shown in the corresponding panels.

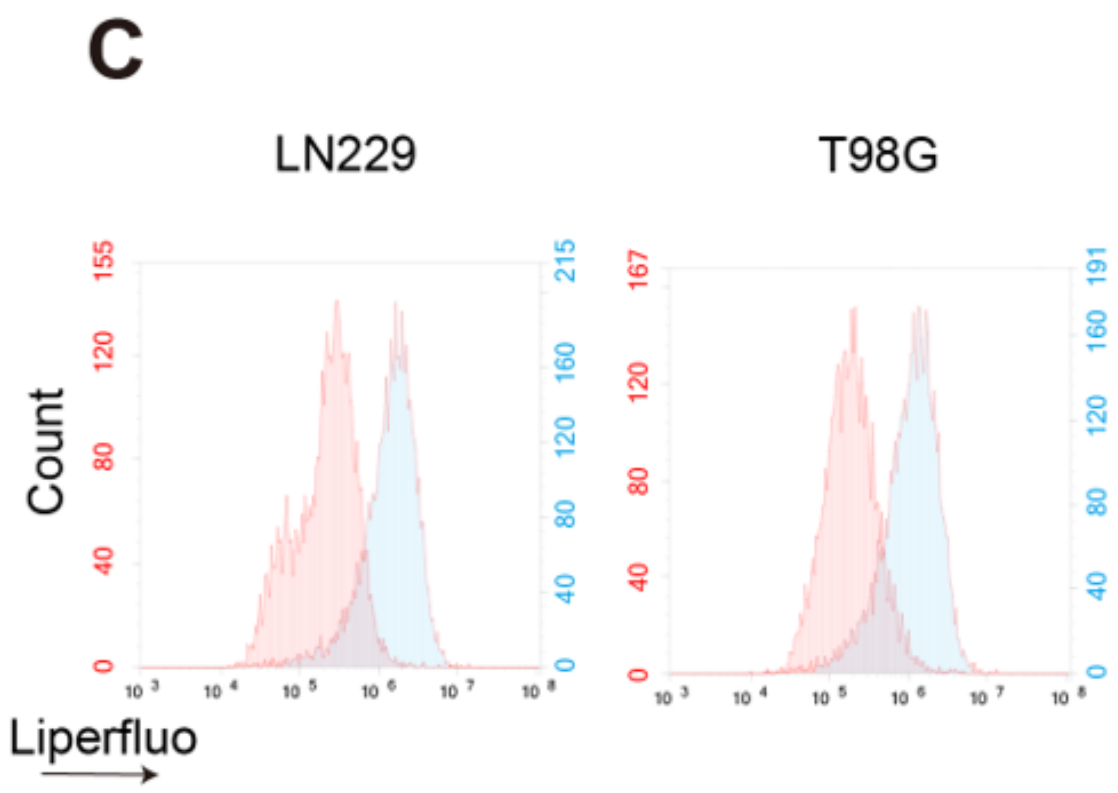


**fig. S7 (C)** Representative flow cytometry histograms of lipid oxidative stress assessed by Liperfluo staining in parental LN229 and T98G cells treated with or without TEFT.

We next examined the downstream metabolic-adaptation pathway. TEFT increased AMPKα phosphorylation and CPT1A protein expression in parental LN229 and T98G cells. Co-immunoprecipitation further demonstrated enhanced interaction between PPARα and its transcriptional co-activator PGC1α in both parental and EGFRvIII-driven models. ChIP-qPCR showed increased PPARα occupancy at predicted PPRE motifs within the CPT1A promoter. The relevant changes made in the results have been added to fig. S6C-F.

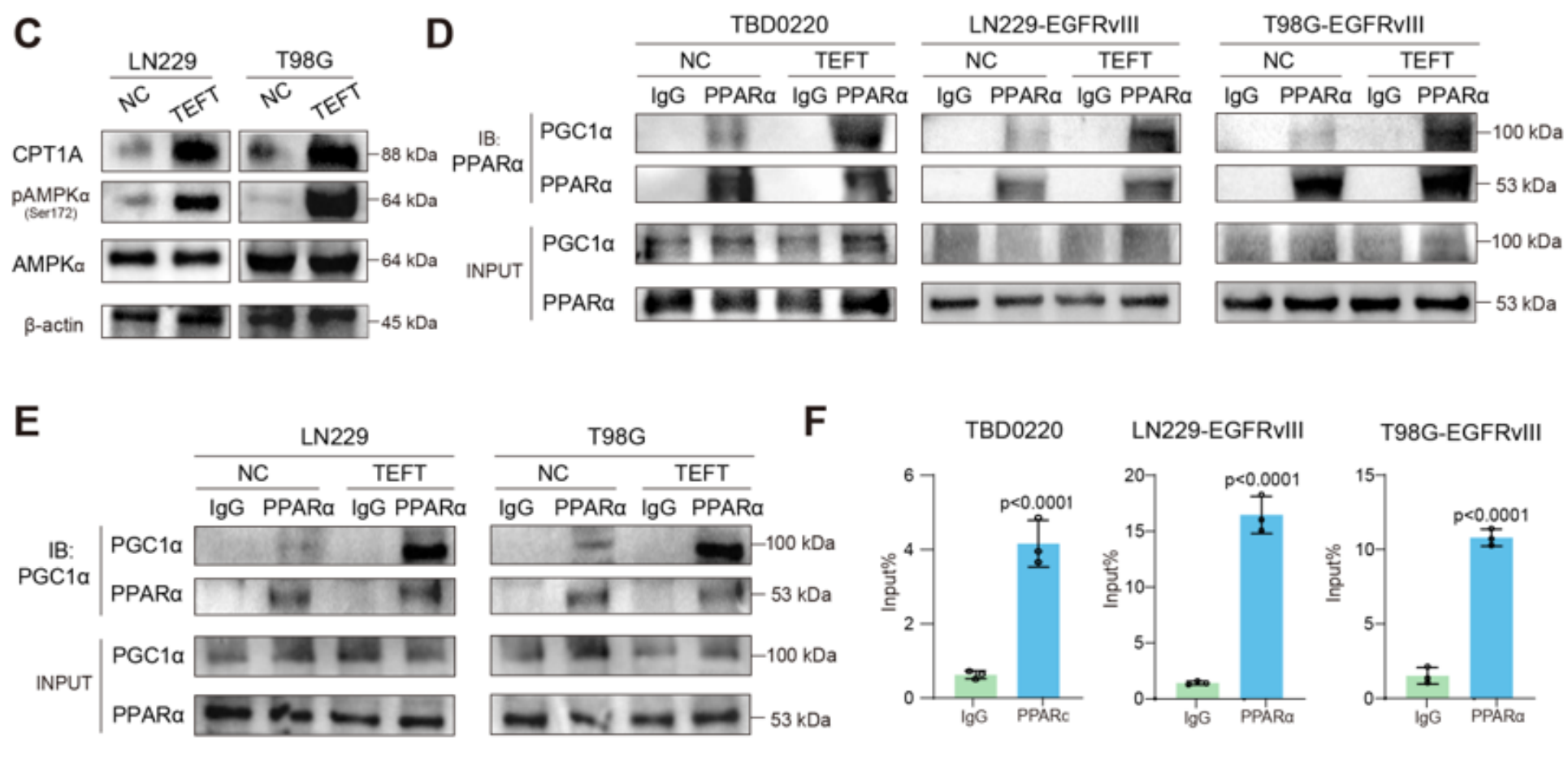


**fig. S6 (C)** Western blot analysis of CPT1A, phosphorylated AMPKα, and total AMPKα in parental LN229 and T98G cells treated with or without TEFT. Data show that TEFT-induced AMPK-CPT1A activation is also observed in parental GBM cells, **(D)** Co-immunoprecipitation analysis of the interaction between PPARα and its transcriptional co-activator PGC1α in TBD0220, LN229-EGFRvIII, and T98G-EGFRvIII cells treated with or without TEFT, **(E)** Co-immunoprecipitation analysis of PPARα–PGC1α interaction in parental LN229 and T98G cells treated with or without TEFT, **(F)** ChIP-qPCR analysis of PPARα occupancy at predicted PPRE motifs within the CPT1A promoter region in TBD0220, LN229-EGFRvIII, and T98G-EGFRvIII cells. Data are presented as percentage of input.

These data support the interpretation that TEFT-induced mitochondrial stress and AMPK–PPARα–CPT1A activation constitute a broadly inducible response across the GBM models examined. We have nevertheless avoided describing this response as universally established across all GBM molecular subtypes, because broader pan-genotypic patient-derived validation and longitudinal exposure models will be required.

Importantly, this general metabolic response is not by itself sufficient to establish the complete resistance program. EGFRvIII provides an additional genotype-specific transcriptional input through constitutive NF-κB/RelA signaling, thereby generating an elevated IDO1 pool that can subsequently be recruited to the CPT1A-associated mitochondrial interface and stabilized. Thus, EGFRvIII does not uniquely generate the initial TEFT-induced metabolic shift; instead, it enables tumor cells to exploit this broadly inducible response and convert it into an IDO1-dependent cytoprotective program.

**Other Changes in the revised manuscript are provided below.**

**Results, lines 219–241;**

To determine whether this stress response was restricted to EGFRvIII-mutant cells, we examined parental LN229 and T98G cells. Additional JC-1 analyses confirmed mitochondrial depolarization in EGFRvIII-driven models (fig. S5A, B). In parental LN229 and T98G cells, TEFT induced mitochondrial depolarization and mitochondrial calcium accumulation, as shown by JC-1, TMRM, and Rhod-2 staining (fig. S5C-G). These results indicate that TEFT-induced mitochondrial and redox stress is broadly

inducible across GBM cells rather than being restricted to EGFRvIII-driven models. GSEA analysis of the transcriptome provided mechanistic insight, showing negative enrichment of oxidative phosphorylation signatures but positive enrichment of the PPARα pathway (fig. S6A). Validating this cellular energy deficit, Western blotting showed activation of the energy sensor AMPK**,** evidenced by increased AMPKα phosphorylation in EGFRvIII-driven models (fig. S6B). TEFT also increased AMPKα phosphorylation and CPT1A expression in parental LN229 and T98G cells, further supporting a broadly induced metabolic-stress response (fig. S6C). Furthermore, Co-IP assays showed that TEFT enhanced the interaction between PPARα and its co-activator PGC1α in both EGFRvIII-driven and parental GBM cells (fig. S6D, E). ChIP-qPCR further showed increased PPARα occupancy at predicted PPRE motifs within the CPT1A promoter (fig. S6F). Together, these findings indicate that TEFT activates a broadly inducible AMPK-PPARα-CPT1A metabolic-stress program. However, this upstream response alone is insufficient to establish the complete IDO1-dependent resistance cascade, which also requires EGFRvIII-mediated IDO1 transcriptional priming. Consistent with this distinction, osimertinib did not abolish TEFT-induced AMPKα phosphorylation or CPT1A upregulation (fig. S6G), suggesting that EGFR inhibition primarily disrupts the IDO1 supply arm rather than the upstream metabolic-stress response.

**Discussion, lines 464–475 and 554–559;**

A pivotal discovery of our work is the counter-intuitive metabolic response triggered by TEFT. We observed that TEFT induces mitochondrial and bioenergetic stress characterized by mitochondrial depolarization, mitochondrial calcium accumulation, and lipid oxidative stress**.** Instead of succumbing to this stress, tumor cells initiate an active adaptation via the AMPK–PPARα axis to upregulate CPT1A. Crucially, although we initially delineated this pathway in EGFRvIII-mutant cells, the triggering bioenergetic stress is a broadly inducible biophysical consequence of electric-field exposure rather than a unique feature of the mutation. Parental LN229 and T98G cells also exhibited mitochondrial stress, AMPKα phosphorylation, PPARα–PGC1α engagement, and CPT1A induction following TEFT exposure. This suggests that upregulation of CPT1A-mediated fatty-acid metabolism may represent a common metabolic adaptation employed by diverse GBM cells under TEFT stress, although this upstream response alone is insufficient to establish the complete EGFRvIII–IDO1 resistance cascade. (Discussion, lines 464–475)

Second, although TEFT-induced mitochondrial stress and AMPK-PPARα-CPT1A activation were observed in both EGFRvIII-driven and parental GBM cells, it remains unknown whether this metabolic program becomes a general mechanism of acquired resistance across molecular subtypes. Longitudinal TEFT exposure models and pan-genotypic patient-derived systems will be needed to determine how broadly this adaptive response applies and whether CPT1A represents a shared therapeutic vulnerability. (Discussion, lines 555–560)

2. CPT1A is localized on the outer membrane of mitochondria, while Succinyl-CoA is generated in the matrix. Given the TEFT-induced mitochondrial dysfunction, the source of sufficient Succinyl-CoA is unclear. Furthermore, the topological mechanism is puzzling: does IDO1 translocate into the matrix, or is Succinyl-CoA transported out? Please clarify this spatial compartmentalization in the Discussion section and update the schematic model (Fig. 4M) accordingly.

**Response:** We agree that the submitochondrial topology of the proposed mechanism required clarification. We therefore added complementary biochemical and imaging analyses and revised the mechanistic model to distinguish experimentally supported localization from unresolved acyl-donor transport.

First, subcellular fractionation followed by IDO1 co-immunoprecipitation showed that TEFT enhanced CPT1A–IDO1 complex formation and that the complex was enriched in the mitochondrial fraction of TBD0220 and LN229-EGFRvIII cells. ATP5A was used as a mitochondrial marker, whereas Calnexin was used as an endoplasmic-reticulum contamination control. The TEFT-enhanced interaction was also reproduced in the patient-derived LG-260 model. The relevant changes made in the results have been added to Fig. 4B.

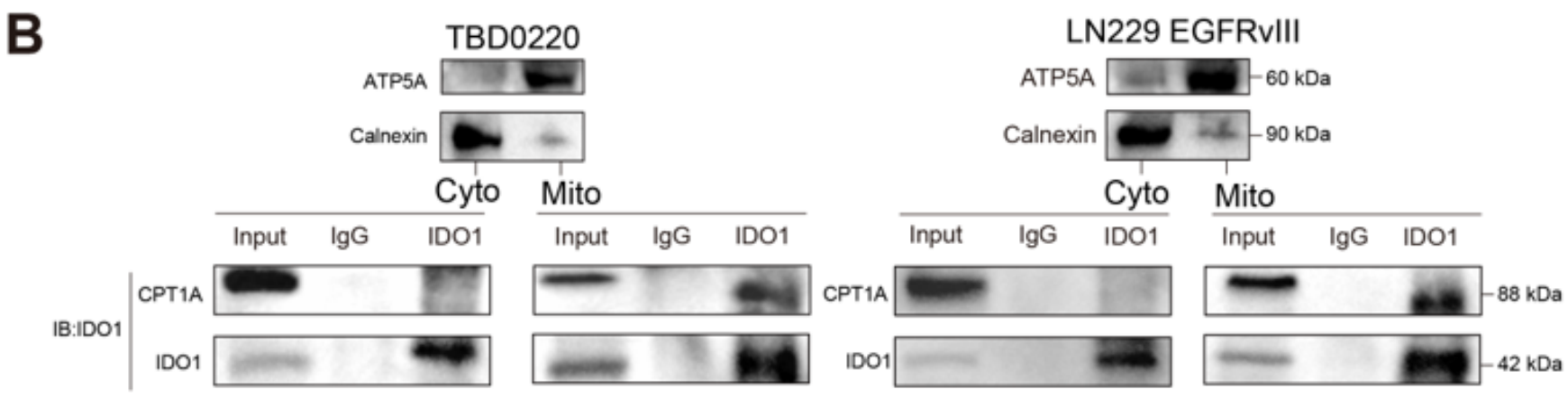


**Fig. 4 (B)** Subcellular fractionation followed by IDO1 co-immunoprecipitation in TBD0220 and LN229-EGFRvIII cells. CPT1A-IDO1 complex formation was enriched in the mitochondrial fraction. ATP5A and Calnexin were used to assess mitochondrial and non-mitochondrial fraction quality, respectively.

Second, high-resolution three-dimensional z-stack imaging showed increased spatial overlap between IDO1 and CPT1A-positive structures after TEFT. Conventional confocal imaging and line-scan analyses provided additional spatial support in TBD0220, LN229-EGFRvIII, and T98G-EGFRvIII cells. The relevant changes made in the results have been added to Fig. 4I.

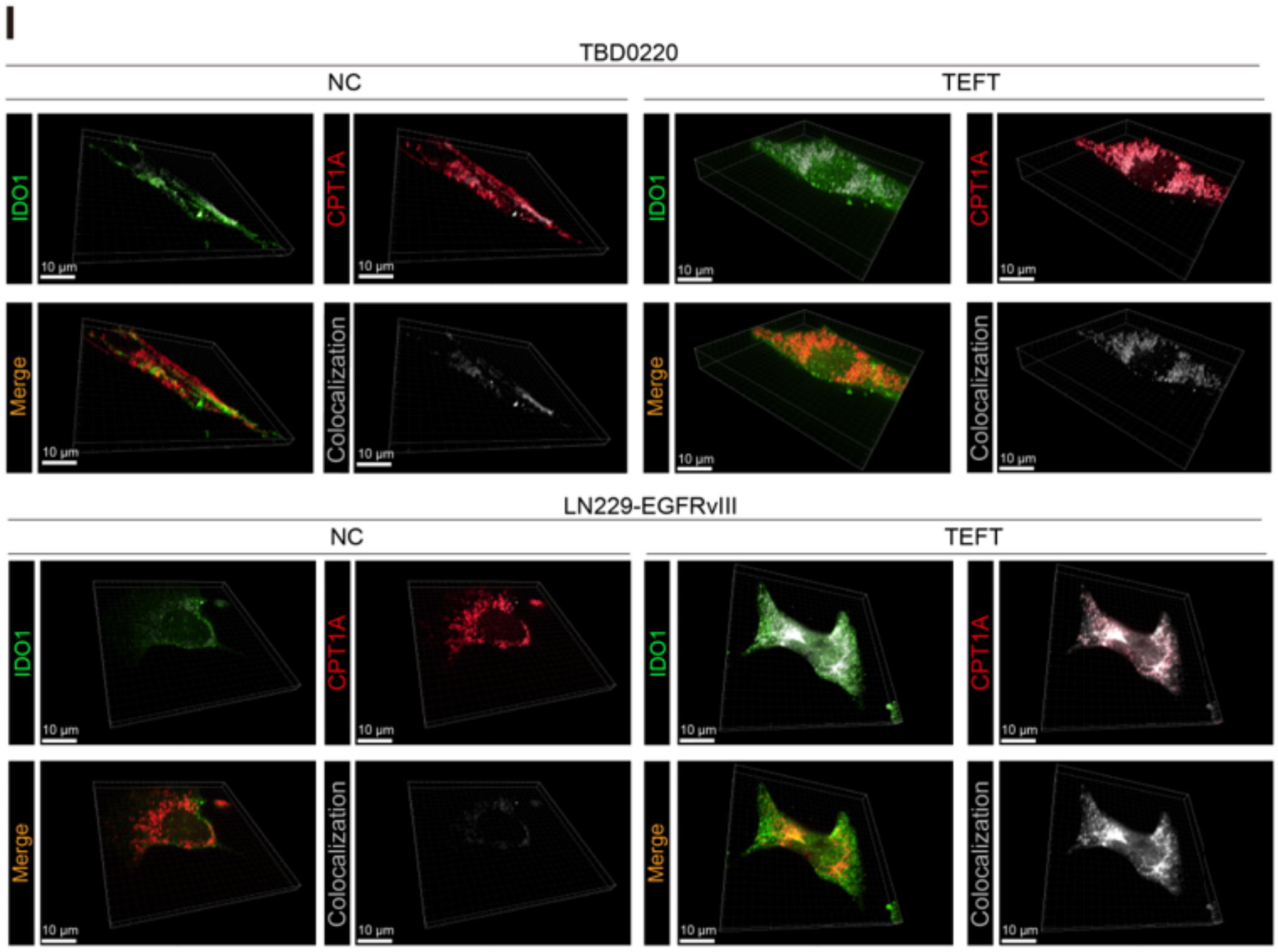


**Fig. 4 (I)** High-resolution 3D confocal imaging showing TEFT-enhanced colocalization between IDO1 and CPT1A-positive mitochondrial structures in TBD0220 and LN229-EGFRvIII cells. CPT1A was used as the OMM-resident mechanistic interactor defining the CPT1A-associated mitochondrial interface. Scale bars, 10 µm.

Because CPT1A is an outer mitochondrial membrane-resident protein, these data support recruitment of IDO1 to a CPT1A-associated outer mitochondrial membrane interface. They do not support, and we do not claim, import of IDO1 into the mitochondrial matrix.

We also measured total cellular succinyl-CoA and found that the pool was not depleted after TEFT. However, this measurement does not resolve submitochondrial succinyl-CoA distribution or demonstrate export of succinyl-CoA from the matrix. We therefore revised the Discussion and schematic to present a working CPT1A-associated mitochondrial microdomain model. In this model, local metabolic organization or acyl-donor accessibility at the outer mitochondrial membrane may support IDO1 succinylation, however the precise source and route of the donor remain unresolved. The relevant changes made in the results have been added to fig. S8F.

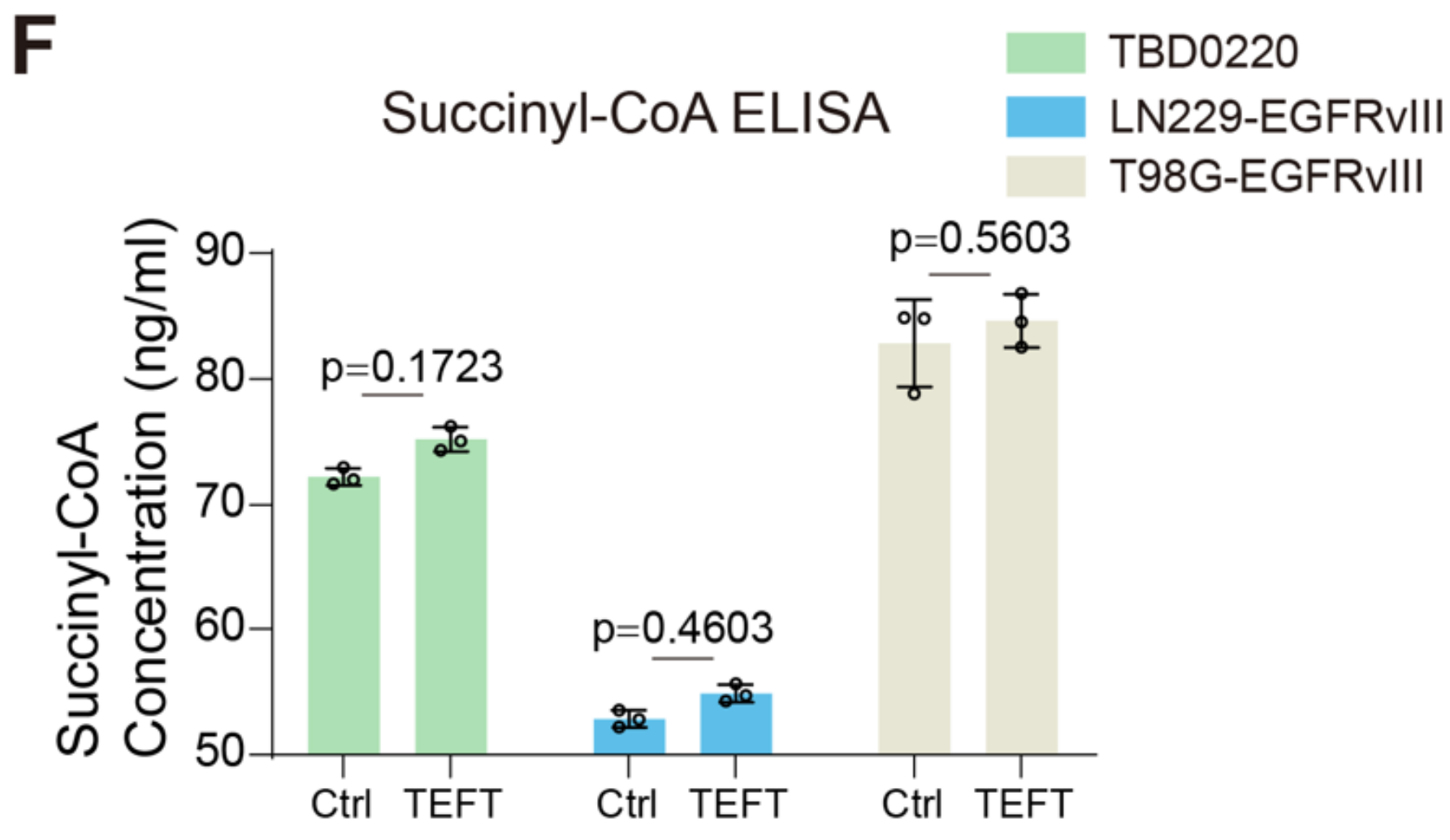


**fig. S8 (F)** Quantification of total cellular succinyl-CoA levels by ELISA in TBD0220, LN229-EGFRvIII, and T98G-EGFRvIII cells treated with or without TEFT. TEFT did not significantly deplete the total cellular succinyl-CoA pool.

The revised manuscript now explicitly states that direct submitochondrial metabolite tracing will be required to distinguish local succinyl-CoA accessibility, transport of an acyl donor or related equivalent, and other possible mechanisms. Fig. 4J has been revised accordingly.

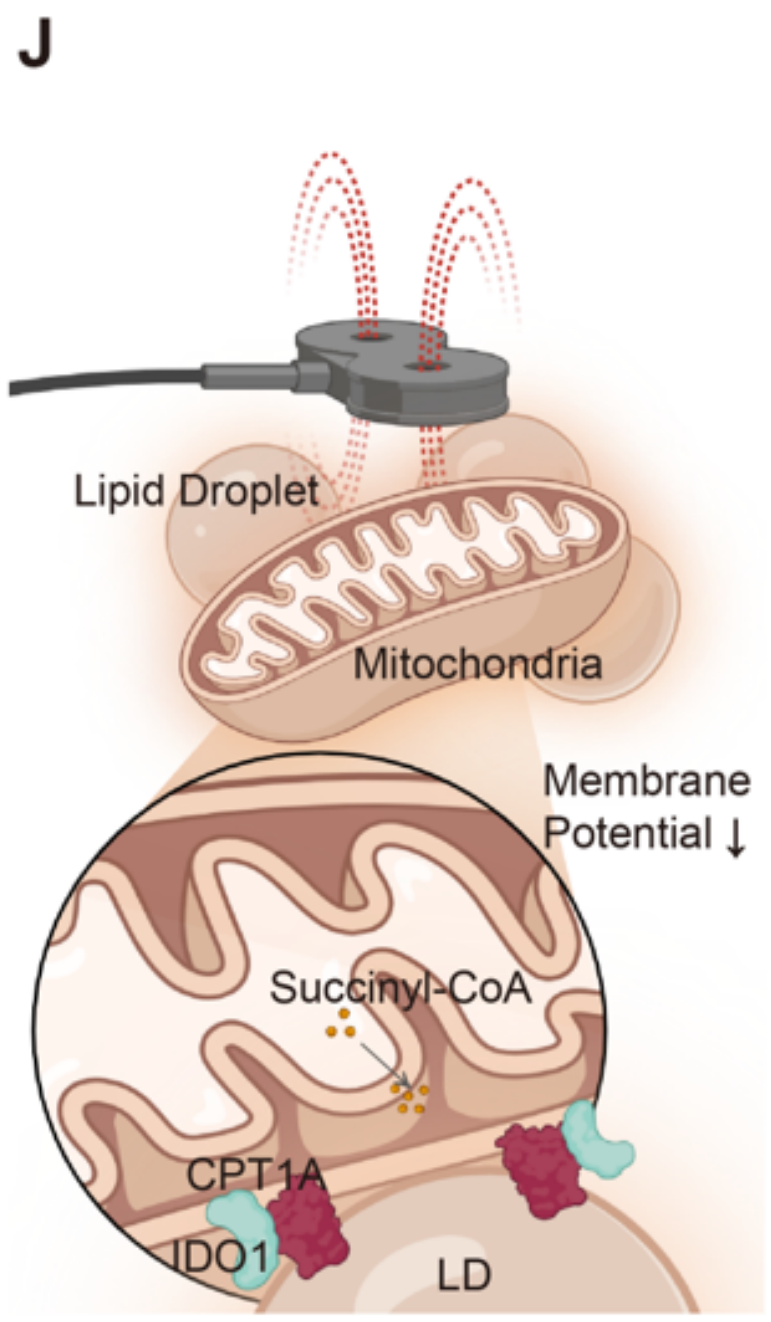


**Fig. 4 (J)** Proposed topological model of CPT1A-mediated IDO1 succinylation under TEFT-induced mitochondrial stress. TEFT induces mitochondrial depolarization and permeability stress, which may

provide local access to succinyl-CoA or succinyl-CoA-related acyl equivalents at the CPT1A-associated OMM interface. [Editorial note: Created in BioRender. Feng, B. (2026) https://BioRender.com/643b38a]

**Other Changes in the revised manuscript are provided below.**
**Results, lines 262–327;**

Motivated by the finding that TEFT drives mitochondrial-fraction enrichment of CPT1A and IDO1, we next investigated whether this spatial proximity facilitates a direct regulatory interaction. We hypothesized that CPT1A stabilizes IDO1 through physical binding and enzymatic modification. Surface plasmon resonance (SPR) assays supported a direct interaction between purified recombinant CPT1A and IDO1 proteins (Fig. 4A). To define the subcellular context of this interaction, we performed subcellular fractionation followed by IDO1 Co-IP in TBD0220 and LN229-EGFRvIII cells. TEFT enhanced the CPT1A-IDO1 interaction, and the complex was enriched in the mitochondrial fraction, with ATP5A and Calnexin used to assess fraction quality (Fig. 4B)**.** Given that CPT1A is an established outer mitochondrial membrane-resident enzyme, these findings support recruitment of IDO1 to a CPT1A-associated OMM interface rather than import of IDO1 into the mitochondrial matrix. Additional Co-IP assays in EGFRvIII-driven models, including patient-derived LG-260 cells, showed that TEFT increased IDO1-CPT1A association while reducing IDO1-TRIM21 association (fig. S8A, B). Structural modeling using AlphaFold3 predicted a CPT1A-IDO1 docking interface near the IDO1 regulatory region containing the K377 succinylation site and the adjacent K389 ubiquitination-associated degron (Fig. 4C).

CPT1A possesses carnitine palmitoyl transferase (CPTase) activity and has also been reported to exhibit non-canonical lysine succinyltransferase-related (LSTase) activity(*31*, *32*). We next investigated whether CPT1A exerts a post-translational modification on IDO1. Given CPT1A's reported moonlighting function as a lysine succinyltransferase, we performed LC-MS/MS analysis on IDO1. This identified lysine 377 (K377) as a candidate succinylation site and lysine 366/389 (K366/K389) as ubiquitination-associated sites (Fig. 4D and fig. S8C). Functional validation demonstrated that CPT1A overexpression increased IDO1 succinylation (Fig. 4E). Conversely, CPT1A depletion reduced IDO1 succinylation and increased IDO1 ubiquitination, whereas re-expression of wild-type CPT1A restored succinylation and reduced ubiquitination (Fig. 4F). To distinguish this effect of the LSTase-related activity of CPT1A from its canonical carnitine palmitoyl transferase (CPTase) function(*33*, *34*), we utilized specific point mutants: CPT1A-G710E, which retains LSTase activity but lacks CPTase activity, and CPT1A-H473A, a catalytically inactive mutant. In CPT1A-depleted cells, re-expression of wild-type CPT1A or the G710E mutant restored IDO1 succinylation and reduced IDO1 ubiquitination, whereas the H473A mutant failed to do so (Fig. 4F). These mutant-rescue data support the requirement of CPT1A LSTase-related activity, rather than classical CPTase activity alone, for the post-translational regulation of IDO1.

To obtain direct biochemical evidence for CPT1A-mediated IDO1 succinylation, we performed cell-free assays using purified recombinant proteins. CPT1A-WT promoted IDO1 succinylation in the presence of succinyl-CoA, whereas the H473A mutant showed reduced activity toward IDO1 (fig. S8D). IDO1 succinylation also increased with increasing succinyl-CoA concentrations in the presence of CPT1A (fig. S8E). Moreover, TEFT did not significantly increase total cellular succinyl-CoA concentrations in TBD0220, LN229-EGFRvIII, or T98G-EGFRvIII cells, arguing against a mechanism driven solely by a global increase in succinyl-CoA availability or nonspecific non-enzymatic succinylation (fig. S8F).

To determine the precise mechanism of stabilization, we generated IDO1 lysine mutants. Cycloheximide (CHX) chase assays revealed that the IDO1-K389R mutant exhibited a significantly extended half-life compared with wild-type IDO1, implicating K389 as an important ubiquitination-associated degron that contributes to IDO1 turnover (Fig. 4G). Consistent with this, the K389R mutation reduced IDO1 ubiquitination (fig. S8G). Evolutionary analysis showed that the K366, K377, and K389 sites are highly conserved across species, supporting the potential functional importance of this regulatory region (fig. S8H). Finally, we verified this axis in the context of TEFT. In TBD0220 and LN229-EGFRvIII cells, TEFT treatment increased IDO1 succinylation and reduced its ubiquitination; importantly, CPT1A knockdown attenuated this TEFT-induced post-translational switch (Fig. 4H). Consistent results were obtained in T98G-EGFRvIII and patient-derived EGFR-driven cells (fig. S8I-L). Thus, TEFT promotes a CPT1A-dependent shift in IDO1 post-translational regulation from ubiquitination toward succinylation, thereby supporting IDO1 stabilization.

To visually corroborate this interaction in situ, we performed immunofluorescence colocalization assays. High-resolution three-dimensional z-stack imaging revealed that TEFT increased the overlap between IDO1 and CPT1A-positive structures in TBD0220 and LN229-EGFRvIII cells (Fig. 4I and fig. S9A). Conventional confocal imaging and line-scan analysis in TBD0220, LN229-EGFRvIII, and T98G-EGFRvIII cells provided additional evidence of TEFT-enhanced spatial proximity between IDO1 and CPT1A-positive mitochondrial structures (fig. S9B-C). Together with the mitochondrial fractionation Co-IP data, these imaging results support recruitment of IDO1 to a CPT1A-associated OMM interface. Collectively, these data support a model in which TEFT-induced bioenergetic stress upregulates CPT1A and promotes its interaction with IDO1 at CPT1A-positive mitochondrial structures. CPT1A-dependent succinylation of IDO1 at K377 functionally antagonizes ubiquitination at the adjacent K389 degron, thereby preserving the IDO1 pool for downstream signaling (Fig. 4J).

### Discussion, lines 480–486 and 560–564;

CPT1A is an established outer mitochondrial membrane-resident enzyme, whereas succinyl-CoA is primarily generated within the mitochondrial matrix. Although TEFT did not globally increase total cellular succinyl-CoA, the metabolite pool was not depleted, indicating that donor availability was maintained. We therefore propose a CPT1A-associated mitochondrial microdomain model in which local metabolic organization may permit access to succinyl-CoA or related acyl-donor availability at the outer mitochondrial membrane interface(*52*, *53*). (Discussion, lines 480–486)

Third, although we mapped the CPT1A LSTase-related activity, the precise source and submitochondrial accessibility of the succinyl-CoA donor require further biochemical dissection. Although total cellular succinyl-CoA was not depleted under TEFT, direct submitochondrial metabolite tracing will be necessary to test the proposed CPT1A-associated outer mitochondrial membrane microdomain model. (Discussion, lines 560–564)

### Methods, lines 680-685;

Conventional confocal images were acquired using a Leica SP8 confocal laser-scanning microscope and analyzed using ImageJ software. For high-resolution IDO1-CPT1A spatial analysis, z-stack images were acquired using an Olympus SpinSR10 super-resolution spinning-disk confocal microscope and

reconstructed and analyzed using Imaris software. Colocalization was evaluated qualitatively by three-dimensional spatial overlap and line-scan fluorescence intensity profiles.

3. CPT1A succinylates IDO1 at K377 under TEFT-induced metabolic stress. However, TEFT can also reduce mitochondrial membrane potential and ATP level. Did authors measure the succinyl CoA levels under TEFT? How succinyl CoA pools are maintained? Whether TEFT increases CPT1A LSTase activity specifically.

**Response:** We thank the reviewer for raising these important questions regarding acyl-donor availability and CPT1A enzymatic output under TEFT-induced bioenergetic stress.

We first quantified total cellular succinyl-CoA in TBD0220, LN229-EGFRvIII, and T98G-EGFRvIII cells after TEFT exposure. Despite mitochondrial depolarization and energetic stress, TEFT did not significantly increase or deplete the total cellular succinyl-CoA pool. Thus, the increased IDO1 succinylation cannot be explained by a generalized increase in cellular succinyl-CoA, but sufficient donor availability was retained. The relevant changes made in the results have been added to fig. S8F.

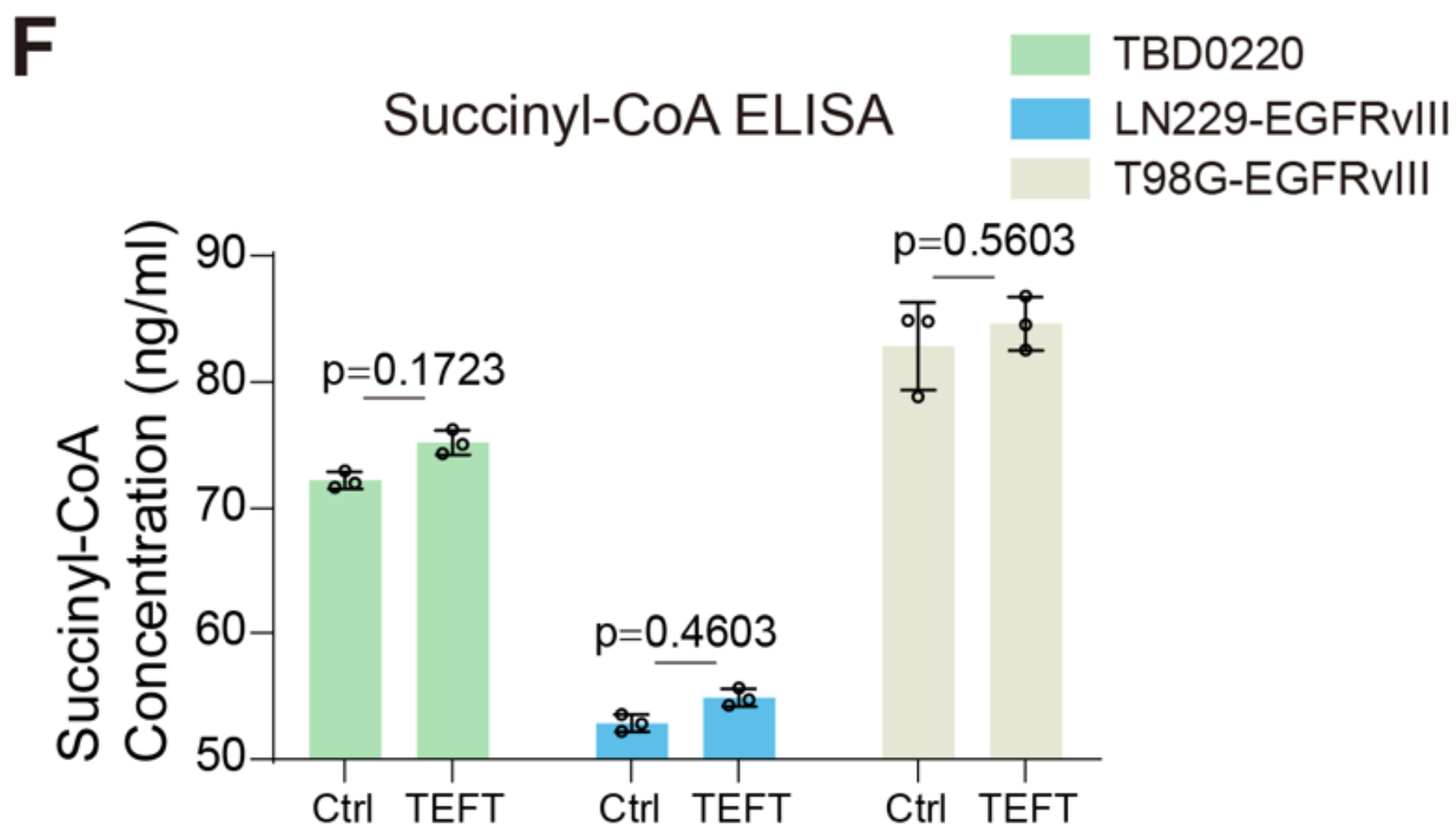


**fig. S8 (F)** Quantification of total cellular succinyl-CoA levels by ELISA in TBD0220, LN229-EGFRvIII, and T98G-EGFRvIII cells treated with or without TEFT. TEFT did not significantly deplete the total cellular succinyl-CoA pool.

We next strengthened the evidence that CPT1A LSTase-related activity contributes to IDO1 succinylation. CPT1A depletion reduced IDO1 succinylation and increased its ubiquitination. Re-expression of wild-type CPT1A or the LSTase-retaining/CPTase-deficient G710E mutant restored IDO1 succinylation and reduced ubiquitination, whereas the catalytically defective H473A mutant did not. The results are shown in Fig. 4F.

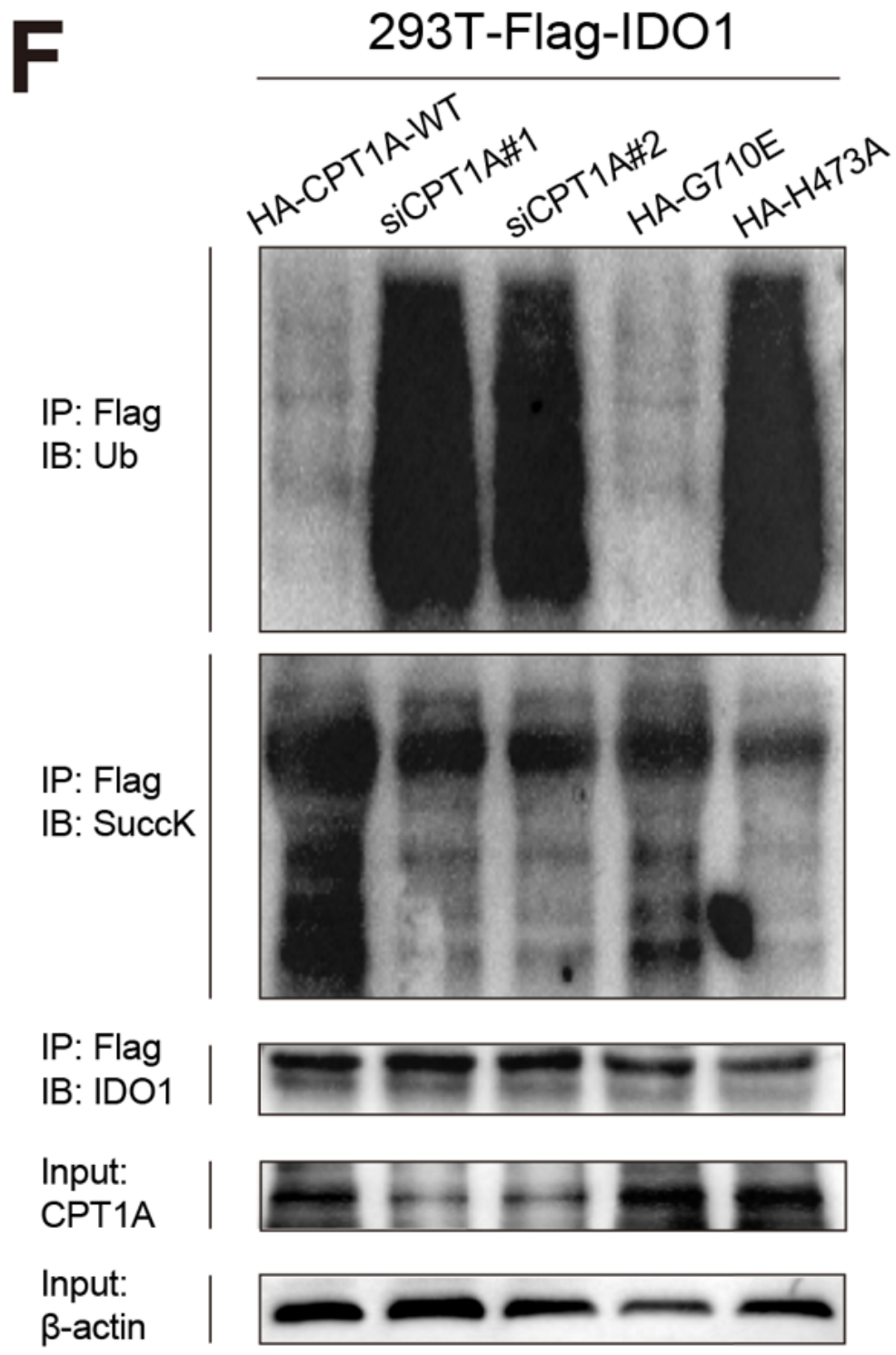


Fig. 4 (F) Rescue experiment in CPT1A-knockdown cells. Re-expression of wild-type (WT) CPT1A or the LSTase-active mutant (G710E) restores IDO1 succinylation and stability, whereas the LSTase-dead mutant (H473A) fails to do so.

In addition, cell-free assays were performed using purified recombinant proteins. CPT1A-WT promoted IDO1 succinylation in the presence of succinyl-CoA, whereas H473A exhibited reduced activity toward IDO1. IDO1 succinylation also increased with increasing succinyl-CoA concentrations in the presence of CPT1A. LC–MS/MS independently identified K377 as a candidate succinylation site. The relevant changes made in the results have been added to fig. S8C-E.

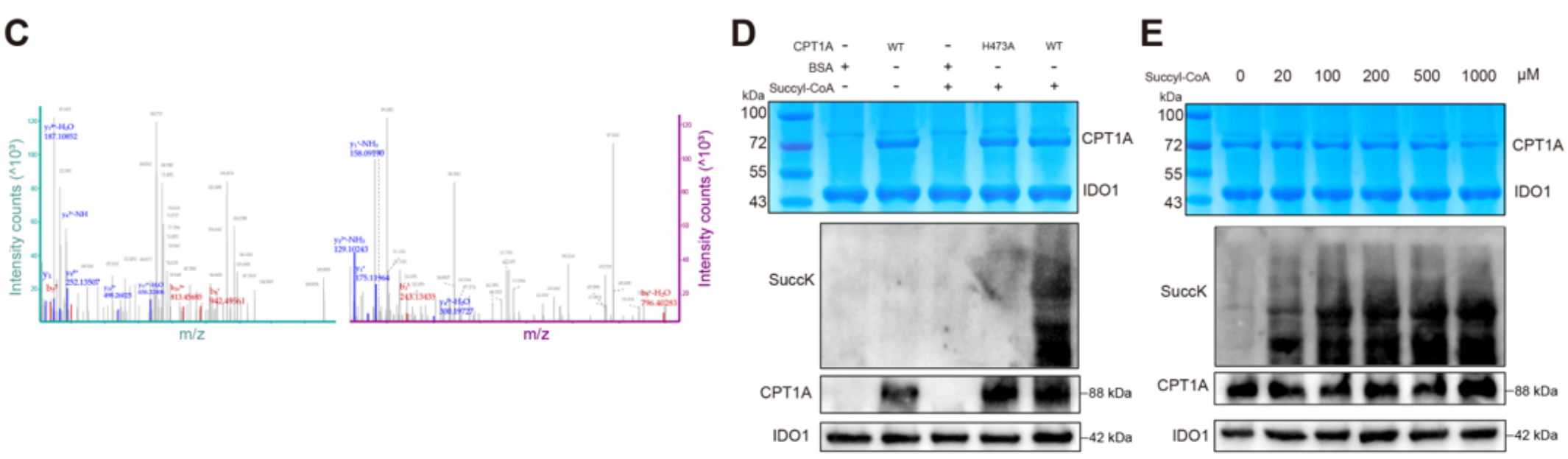


**fig. S8 (C)** Representative LC–MS/MS spectra identifying post-translational modification sites on IDO1, **(D)** In vitro recombinant protein succinylation assay using purified IDO1 and CPT1A proteins. Wild-type CPT1A directly induced IDO1 lysine succinylation in the presence of succinyl-CoA, whereas the catalytically defective H473A mutant showed reduced activity, **(E)** Dose-dependent in vitro

succinylation assay showing increased IDO1 lysine succinylation with increasing succinyl-CoA concentrations in the presence of recombinant CPT1A.

Together, these results support a requirement for CPT1A LSTase-related activity in the post-translational regulation of IDO1 and argue against nonspecific succinylation caused solely by a global substrate excess.

We have nevertheless refined the wording to avoid claiming that TEFT directly allosterically increases the intrinsic catalytic activity of each CPT1A molecule. The current data show that TEFT increases effective CPT1A-dependent succinylation output by increasing CPT1A abundance, promoting its association with IDO1, and maintaining sufficient donor availability. Direct kinetic comparison of purified CPT1A isolated from control and TEFT-treated cells would be required to establish whether TEFT additionally alters CPT1A-specific catalytic turnover.

The mechanism by which the total succinyl-CoA pool is maintained during TEFT has also not been assigned to a single metabolic source. The manuscript now states that energetic impairment does not represent complete exhaustion of TCA-cycle-associated metabolite pools and that the precise submitochondrial source and accessibility of the donor remain to be determined.

**Other Changes in the revised manuscript are provided below.**

**Results, lines 278–315;**

CPT1A possesses carnitine palmitoyl transferase (CPTase) activity and has also been reported to exhibit non-canonical lysine succinyltransferase-related (LSTase) activity(*31, 32*). We next investigated whether CPT1A exerts a post-translational modification on IDO1. Given CPT1A's reported moonlighting function as a lysine succinyltransferase, we performed LC-MS/MS analysis on IDO1. This identified lysine 377 (K377) as a candidate succinylation site and lysine 366/389 (K366/K389) as ubiquitination-associated sites (Fig. 4D and fig. S8C). Functional validation demonstrated that CPT1A overexpression increased IDO1 succinylation (Fig. 4E). Conversely, CPT1A depletion reduced IDO1 succinylation and increased IDO1 ubiquitination, whereas re-expression of wild-type CPT1A restored succinylation and reduced ubiquitination (Fig. 4F). To distinguish this effect of the LSTase-related activity of CPT1A from its canonical carnitine palmitoyl transferase (CPTase) function(*33, 34*), we utilized specific point mutants: CPT1A-G710E, which retains LSTase activity but lacks CPTase activity, and CPT1A-H473A, a catalytically inactive mutant. In CPT1A-depleted cells, re-expression of wild-type CPT1A or the G710E mutant restored IDO1 succinylation and reduced IDO1 ubiquitination, whereas the H473A mutant failed to do so (Fig. 4F). These mutant-rescue data support the requirement of CPT1A LSTase-related activity, rather than classical CPTase activity alone, for the post-translational regulation of IDO1.

To obtain direct biochemical evidence for CPT1A-mediated IDO1 succinylation, we performed cell-free assays using purified recombinant proteins. CPT1A-WT promoted IDO1 succinylation in the presence of succinyl-CoA, whereas the H473A mutant showed reduced activity toward IDO1 (fig. S8D). IDO1 succinylation also increased with increasing succinyl-CoA concentrations in the presence of CPT1A (fig.

S8E). Moreover, TEFT did not significantly increase total cellular succinyl-CoA concentrations in TBD0220, LN229-EGFRvIII, or T98G-EGFRvIII cells, arguing against a mechanism driven solely by a global increase in succinyl-CoA availability or nonspecific non-enzymatic succinylation (fig. S8F).

To determine the precise mechanism of stabilization, we generated IDO1 lysine mutants. Cycloheximide (CHX) chase assays revealed that the IDO1-K389R mutant exhibited a significantly extended half-life compared with wild-type IDO1, implicating K389 as an important ubiquitination-associated degron that contributes to IDO1 turnover (Fig. 4G). Consistent with this, the K389R mutation reduced IDO1 ubiquitination (fig. S8G). Evolutionary analysis showed that the K366, K377, and K389 sites are highly conserved across species, supporting the potential functional importance of this regulatory region (fig. S8H). Finally, we verified this axis in the context of TEFT. In TBD0220 and LN229-EGFRvIII cells, TEFT treatment increased IDO1 succinylation and reduced its ubiquitination; importantly, CPT1A knockdown attenuated this TEFT-induced post-translational switch (Fig. 4H). Consistent results were obtained in T98G-EGFRvIII and patient-derived EGFR-driven cells (fig. S8I-L). Thus, TEFT promotes a CPT1A-dependent shift in IDO1 post-translational regulation from ubiquitination toward succinylation, thereby supporting IDO1 stabilization.

### Discussion, lines 480–495 and 559–563;

CPT1A is an established outer mitochondrial membrane-resident enzyme, whereas succinyl-CoA is primarily generated within the mitochondrial matrix. Although TEFT did not globally increase total cellular succinyl-CoA, the metabolite pool was not depleted, indicating that donor availability was maintained. We therefore propose a CPT1A-associated mitochondrial microdomain model in which local metabolic organization may permit access to succinyl-CoA or related acyl-donor availability at the outer mitochondrial membrane interface(*52*, *53*). This topological model will require direct submitochondrial metabolite tracing. NAC increased TEFT-induced apoptosis and attenuated CPT1A accumulation, suggesting that redox stress contributes to the upstream metabolic adaptation. (*54*, *55*). Crucially, upregulated CPT1A also transcends its canonical metabolic role. Through its LSTase-related activity, CPT1A promotes IDO1 succinylation at K377, which functionally antagonizes ubiquitination at the adjacent K389 degron and limits TRIM21-mediated proteasomal degradation**.** This reveals a dual-input mechanism: TEFT-induced metabolic stress upregulates CPT1A, whereas EGFRvIII supplies the transcriptionally primed IDO1 substrate. When these inputs converge, the metabolic adaptation induced by TEFT stabilizes the protein that supports downstream resistance, thereby creating a compensatory mechanism that limits therapeutic efficacy. (Discussion, lines 480–495)

Third, although we mapped the CPT1A LSTase-related activity, the precise source and submitochondrial accessibility of the succinyl-CoA donor require further biochemical dissection. Although total cellular succinyl-CoA was not depleted under TEFT, direct submitochondrial metabolite tracing will be necessary to test the proposed CPT1A-associated outer mitochondrial membrane microdomain model. (Discussion, lines 560–567)

### Methods, lines 859–867;

Total cellular succinyl-CoA was quantified using an SCoA ELISA Kit (Fusheng, Shanghai) according to the manufacturer’s instructions. Cells were lysed using a non-denaturing lysis buffer to minimize

interference with the assay, and the lysates were clarified by centrifugation. Samples and standards were analyzed in duplicate. Absorbance was measured at 450 nm with 630 nm used as the reference wavelength on a TECAN microplate reader. The mean optical density of duplicate wells was corrected by subtracting the blank value, and succinyl-CoA concentrations were calculated using a four-parameter logistic standard curve. Samples with absorbance values above the calibration range were diluted and reanalyzed, with the corresponding dilution factor included in the final calculation.

4. While Osimertinib reduces IDO1 mRNA via NF-kB (supply side), the paper focuses on protein stabilization (stability side). Does Osimertinib also prevent the TEFT-induced metabolic adaptation (i.e., blocking AMPK/CPT1A upregulation)? Clarifying whether the drug targets both transcription and the metabolic stress response would refine the therapeutic logic.

**Response:** We agree that determining whether osimertinib acts on one or both inputs of the resistance mechanism is important for defining the therapeutic logic of the combination.

We therefore examined the AMPK–CPT1A pathway in TBD0220 and LN229-EGFRvIII cells treated with vehicle, osimertinib, TEFT, or the combination. TEFT increased AMPKα phosphorylation and CPT1A protein expression in both models. Importantly, osimertinib did not abolish TEFT-induced AMPKα activation or CPT1A upregulation. The relevant changes made in the results have been added to fig. S6G.

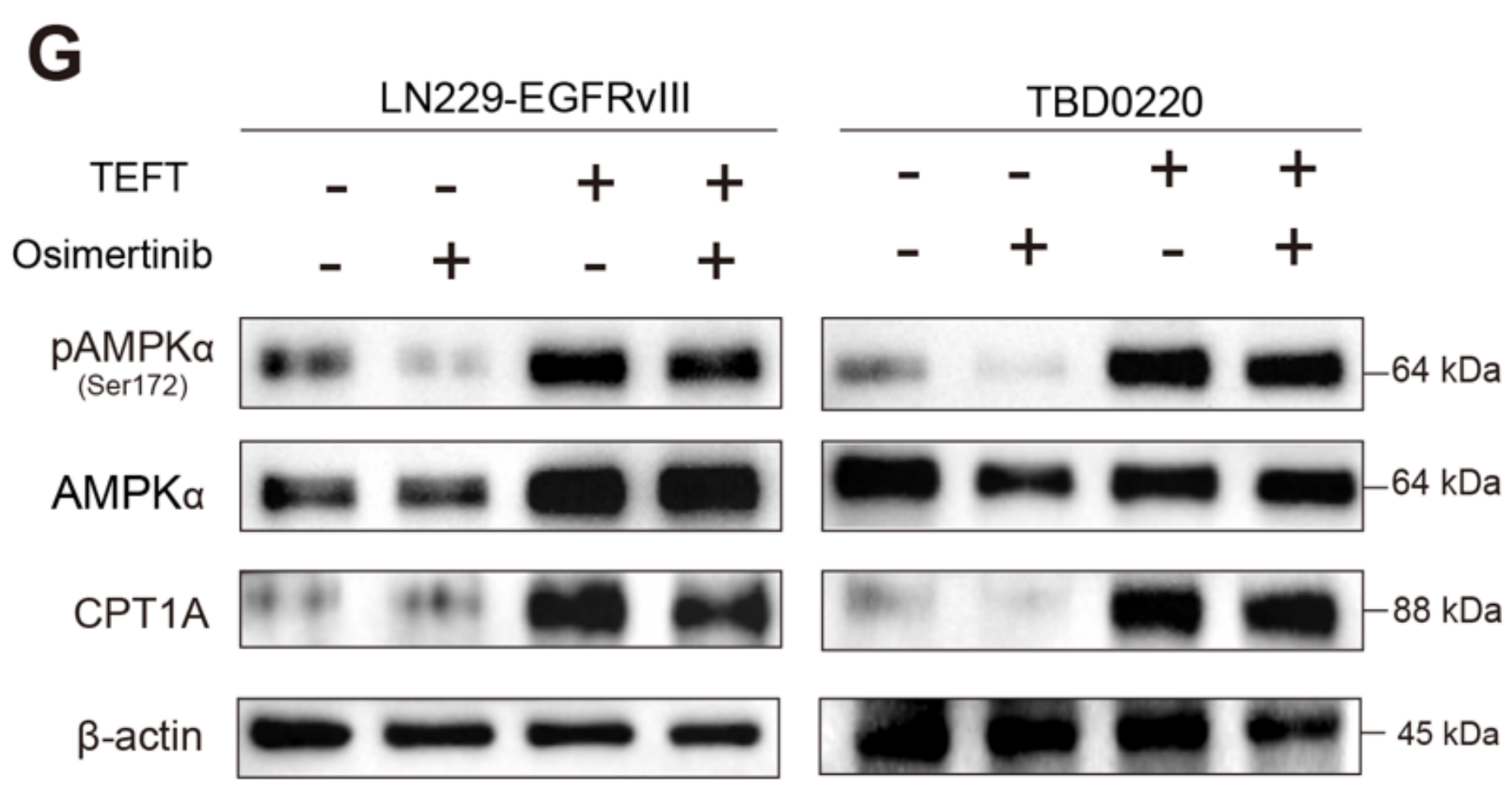


**fig. S6 (G)** Western blot analysis of the AMPK–CPT1A axis in LN229-EGFRvIII and TBD0220 cells treated with osimertinib, TEFT, or the combination. Osimertinib did not abolish TEFT-induced AMPKα phosphorylation or CPT1A upregulation.

These results indicate that osimertinib does not primarily restore TEFT sensitivity by preventing the upstream mitochondrial and metabolic-stress response. Instead, it predominantly suppresses the EGFRvIII–NF-κB-dependent transcriptional supply of

IDO1. Consequently, TEFT continues to impose metabolic stress and CPT1A remains inducible, but the IDO1 pool available for CPT1A-mediated stabilization is reduced.

This distinction supports a two-input model. TEFT provides the metabolic-stress input that activates the AMPK–PPARα–CPT1A program, whereas EGFRvIII provides the IDO1 transcriptional input. Osimertinib selectively disrupts the latter and therefore prevents the TEFT-induced CPT1A response from being efficiently coupled to IDO1 accumulation, kynurenine–AhR activation, and DCLK1/ARHGEF2-mediated cytoskeletal protection.

Thus, TEFT and osimertinib act through complementary rather than redundant mechanisms: TEFT maintains biophysical pressure on the mitotic apparatus, whereas osimertinib disables the genotype-dependent pathway that converts metabolic adaptation into structural resistance.

**Other Changes in the revised manuscript are provided below.**

**Results, lines 236–241;**

However, this upstream response alone is insufficient to establish the complete IDO1-dependent resistance cascade, which also requires EGFRvIII-mediated IDO1 transcriptional priming. Consistent with this distinction, osimertinib did not abolish TEFT-induced AMPKα phosphorylation or CPT1A upregulation (fig. S6G), suggesting that EGFR inhibition primarily disrupts the IDO1 supply arm rather than the upstream metabolic-stress response.

**Discussion, lines 535–539;**

By blocking EGFRvIII signaling, osimertinib suppresses the NF-κB-dependent IDO1 transcriptional supply, thereby reducing the IDO1 pool available for TEFT-induced CPT1A-mediated stabilization. Osimertinib did not abolish TEFT-induced AMPKα phosphorylation or CPT1A upregulation, indicating that it acts primarily on the EGFR-dependent IDO1 supply arm rather than eliminating the upstream metabolic-stress response.

5. In vitro TEFT can generate heat. Did the authors monitor culture temperature to rule out Heat Shock Protein (HSP) activation as a confounder for protein stabilization? A brief confirmation of temperature control in the Materials and Methods is recommended. accordingly.

**Response:** We agree that excluding thermal artifacts is essential because excessive heating could activate heat-shock responses and confound the interpretation of protein-stability experiments.

We therefore monitored the culture system during TEFT exposure using thermal infrared imaging. No appreciable heat accumulation was observed in the culture region, and the temperature remained within the physiological culture range during device operation.

In addition, the in vitro TEFT device contains an integrated active temperature-control module that continuously monitors and regulates culture temperature. All in vitro experiments were performed at 37°C under this active temperature-control condition.

The Methods section has been revised to specify the temperature-control procedure together with the field intensity, frequency, and exposure duration. These controls make a heat-induced protein-stabilization artifact unlikely under the experimental conditions used.

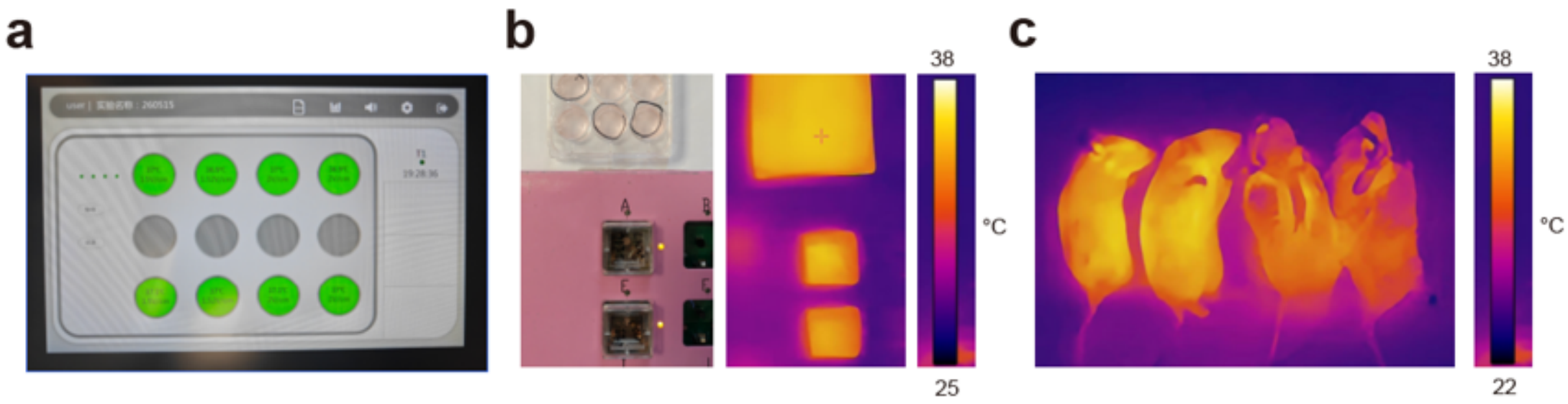


**Changes in the revised manuscript:**
**Methods, lines 627–640.**

**In vitro Tumor Electric Field Therapy**

The TEFT device used for in vitro experiments was provided by Antai Kangcheng Biotechnology Co., Ltd. GBM cells in the logarithmic growth phase were detached with trypsin, counted, and resuspended in 600 μL of high-glucose DMEM. A total of 6 × $10^4$ cells were seeded onto 20mm cell culture coverslips and allowed to adhere overnight before being transferred into TEFT culture dishes. For patient-derived glioblastoma organoids (GBOs), established organoids were transferred directly into TEFT culture dishes, which were placed on an orbital shaker at 120 rpm to maintain suspension culture during treatment. TEFT was applied at a frequency of 200 kHz and a field intensity of 1.5 V/cm. The culture temperature was maintained at 37°C throughout the exposure. TEFT was applied for 72 h unless otherwise indicated. The TEFT culture device contained an integrated active temperature-control module that continuously monitored and regulated the temperature during electric-field exposure. During representative treatment runs, infrared thermography was used to verify that TEFT exposure did not produce focal heat accumulation within the culture region.

6. To further corroborate the functional enzymatic activity of the stabilized IDO1, it would be strengthened to directly measure the concentration of Kynurenine in the culture supernatant (via ELISA or HPLC) in EGFRvIII cells treated with TEFT versus controls.

**Response:** We sincerely thank the reviewer for this constructive suggestion. We agree that measurement of kynurenine, the principal product of IDO1-mediated tryptophan catabolism, provides a functional readout of whether the stabilized IDO1 remains enzymatically active. Following the reviewer's recommendation, we quantified kynurenine concentrations in culture supernatants using a competitive ELISA. TEFT

significantly increased extracellular kynurenine levels in three EGFRvIII-driven GBM models—TBD0220, LN229-EGFRvIII, and T98G-EGFRvIII—compared with their corresponding untreated controls (fig. S10D).

D

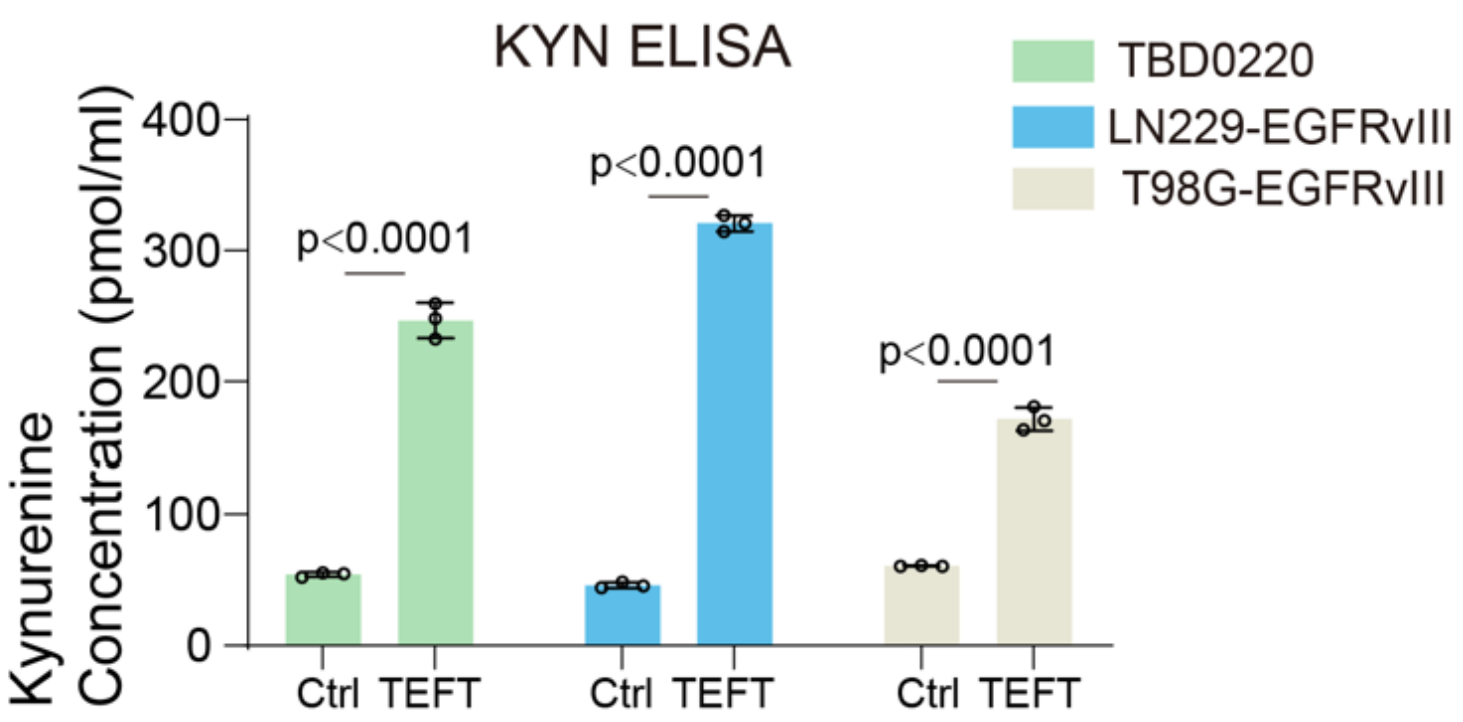


**fig. S10 (D)** Kynurenine ELISA showing increased extracellular kynurenine levels in the culture supernatants of TBD0220, LN229-EGFRvIII, and T98G-EGFRvIII cells after TEFT treatment.

We therefore quantified kynurenine concentrations in culture supernatants using a competitive ELISA. TEFT increased extracellular kynurenine levels in three EGFRvIII-driven GBM models: TBD0220, LN229-EGFRvIII, and T98G-EGFRvIII cells. Samples and standards were analyzed in duplicate, concentrations were calculated from a four-parameter logistic standard curve, and the results were normalized to the total protein content of the corresponding cell lysates.

These data demonstrate that TEFT-associated stabilization of IDO1 is accompanied by increased tryptophan-catabolic output. The increased kynurenine production also provides a biochemical basis for the observed AhR activation, including AhR nuclear accumulation and induction of the canonical AhR-responsive genes CYP1A1 and CYP1B1.

We have accordingly incorporated the kynurenine data into the Results and added the complete assay procedure to the Methods.

**Other Changes in the revised manuscript are provided below.**
**Results, lines 329–340;**

The stabilization of IDO1 initiates a crucial downstream signaling cascade by converting tryptophan into kynurenine, a high-affinity endogenous ligand for the aryl hydrocarbon receptor (AhR) (*35*, *36*). To determine whether TEFT-stabilized IDO1 remained enzymatically active, we measured kynurenine levels in culture supernatants. Kynurenine ELISA showed that TEFT increased extracellular kynurenine concentrations in EGFRvIII-driven GBM models, including TBD0220, LN229-EGFRvIII, and T98G-EGFRvIII cells (fig. S10D). We found that TEFT treatment in EGFRvIII cells significantly promoted AhR nuclear translocation (Fig. 5A, B), supporting its activation. Similar AhR nuclear accumulation was observed in T98G-EGFRvIII and patient-derived LG-260 cells (fig. S10A, B). Importantly, IDO1

knockdown or EGFR inhibition with osimertinib reduced TEFT-induced AhR nuclear accumulation, supporting placement of AhR activation downstream of EGFRvIII-dependent IDO1 signaling (Fig. 5B and fig. S10B).

**Methods, lines 851–867;**

**Kynurenine and Succinyl-CoA Quantification**

Kynurenine concentrations in culture supernatants were quantified using a competitive Kynurenine ELISA Kit (EU0188; FineTest) according to the manufacturer's instructions. Culture supernatants were collected after the indicated treatments and clarified by centrifugation to remove cells and debris. The clarified supernatants and standards were assayed in duplicate. Absorbance was measured at 450 nm using a TECAN microplate reader. Kynurenine concentrations were calculated from a four-parameter logistic standard curve.

7. In Figure 3E authors analyze protein levels in isolated mitochondria using flow cytometry. Please provide a control histogram using a known mitochondrial marker (e.g., ATP5A / Complex V) to confirm the identity of the gated population.

**Response:** We agree that validating the identity and purity of the gated particles is essential for interpreting flow-cytometric measurements in isolated mitochondrial fractions.

We therefore added the complete gating strategy together with an ATP5A control histogram. ATP5A, a component of mitochondrial complex V, showed a robust positive signal in the gated particle population. Calnexin was included as an endoplasmic-reticulum contamination control. The particles used for subsequent analysis were defined as ATP5A-positive and Calnexin-negative.

Unstained and single-stained controls were used to establish fluorescence gates and compensation. IDO1 and CPT1A signals were quantified only within the ATP5A-positive/Calnexin-negative mitochondrial gate. The relevant changes made in the results have been added to fig. S4K.

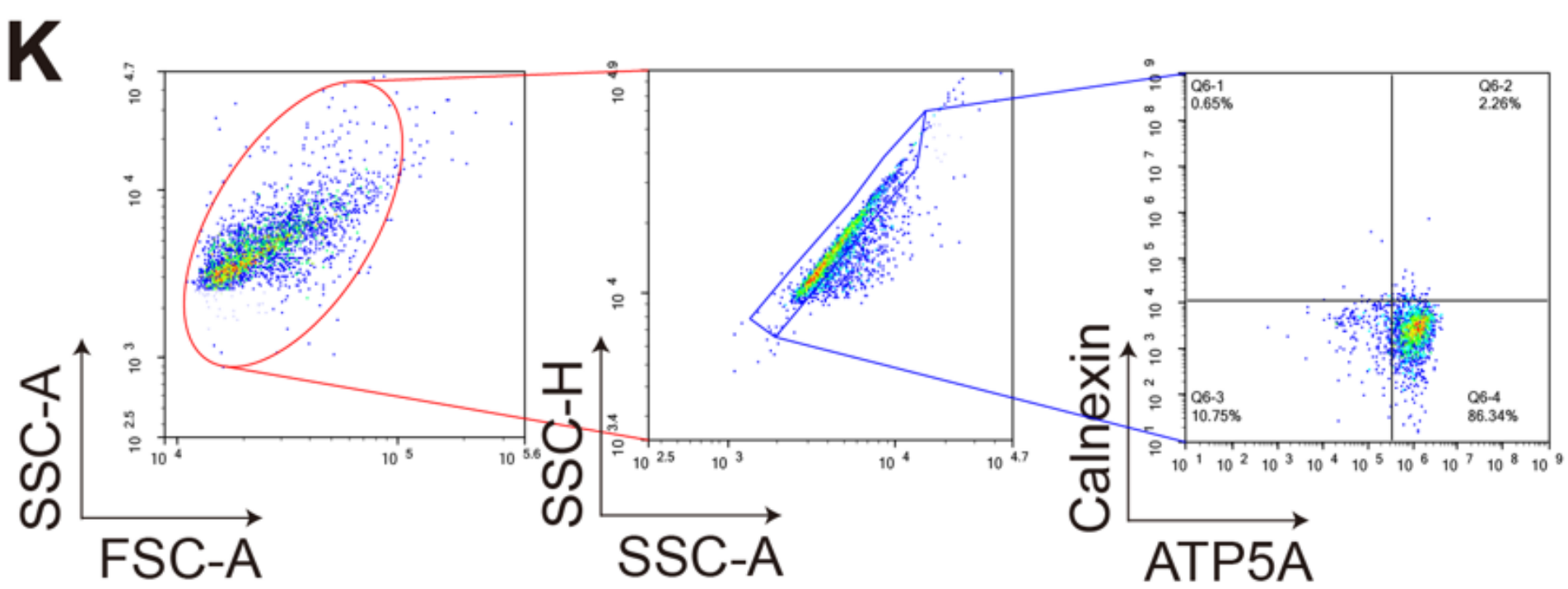

**fig. S4 (K)** Representative gating strategy for flow cytometric analysis of isolated mitochondrial particles. ATP5A was used as a mitochondrial marker and Calnexin as an endoplasmic reticulum contamination control, supporting enrichment of the gated mitochondrial population.

Independent immunoblotting of isolated fractions provided an additional quality-control approach. ATP5A was used as a mitochondrial marker, whereas Calnexin was used as an endoplasmic-reticulum contamination control, respectively. The relevant changes made in the results have been added to Fig. 4B.

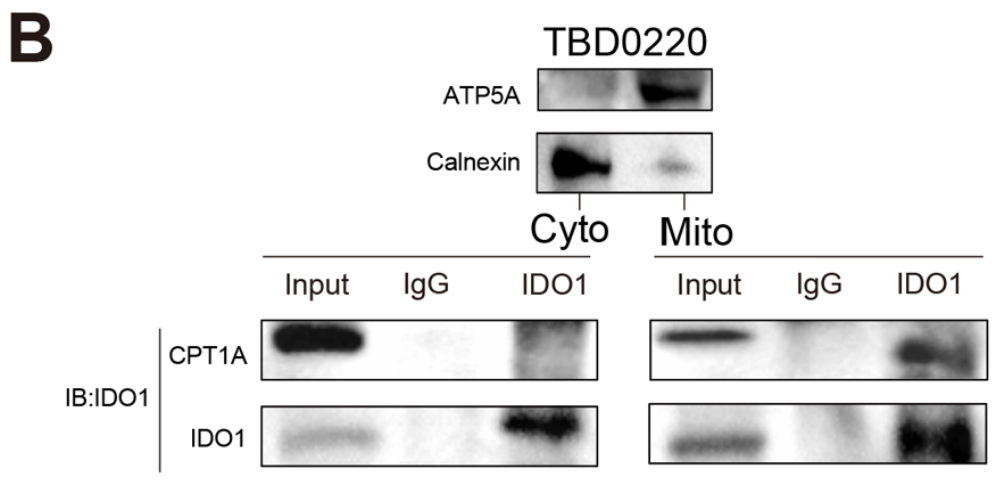


**Fig. 4 (B)** Subcellular fractionation followed by IDO1 co-immunoprecipitation in TBD0220 and LN229-EGFRvIII cells. CPT1A-IDO1 complex formation was enriched in the mitochondrial fraction. ATP5A and Calnexin were used to assess mitochondrial and non-mitochondrial fraction quality, respectively.

These controls support the interpretation that the signals shown in Fig. 3E represent mitochondrial-fraction-associated IDO1 and CPT1A rather than nonspecific cellular debris or contaminating membrane particles.

**Other Changes in the revised manuscript are provided below.**
**Results, lines 211–212;**

The gated mitochondrial population was supported by ATP5A-positive and Calnexin-negative controls (fig. S4K).

**Methods, lines 795–800;**

For flow cytometric analysis, intact mitochondrial pellets were resuspended in PBS containing 2% BSA, fixed and permeabilized, and subsequently stained with antibodies against ATP5A, Calnexin, IDO1, and CPT1A. The mitochondrial population was identified as ATP5A-positive and Calnexin-negative, with unstained and single-stained controls used to define gating and fluorescence compensation. IDO1 and CPT1A signals were quantified within the gated mitochondrial population.

8. Since TEFT induces mitochondrial stress and lipid peroxidation (Fig. 3L), treating cells with an ROS scavenger (like NAC) to see if it modulates IDO1 stabilization or cell survival would provide an insight into the redox-dependency of this mechanism.

**Response:** We agree that redox perturbation provides a useful means of determining whether oxidative stress contributes functionally to the TEFT-induced adaptation.

We therefore treated TBD0220 and LN229-EGFRvIII cells with the ROS scavenger N-acetylcysteine during TEFT exposure. NAC co-treatment increased TEFT-induced apoptosis in both models, indicating that redox buffering partially restored TEFT sensitivity. Western blot analysis showed that NAC attenuated TEFT-induced CPT1A accumulation. In contrast, total IDO1 abundance was only modestly affected and did not show a consistent reduction across the two models. The relevant changes made in the results have been added to fig. S7E–G.

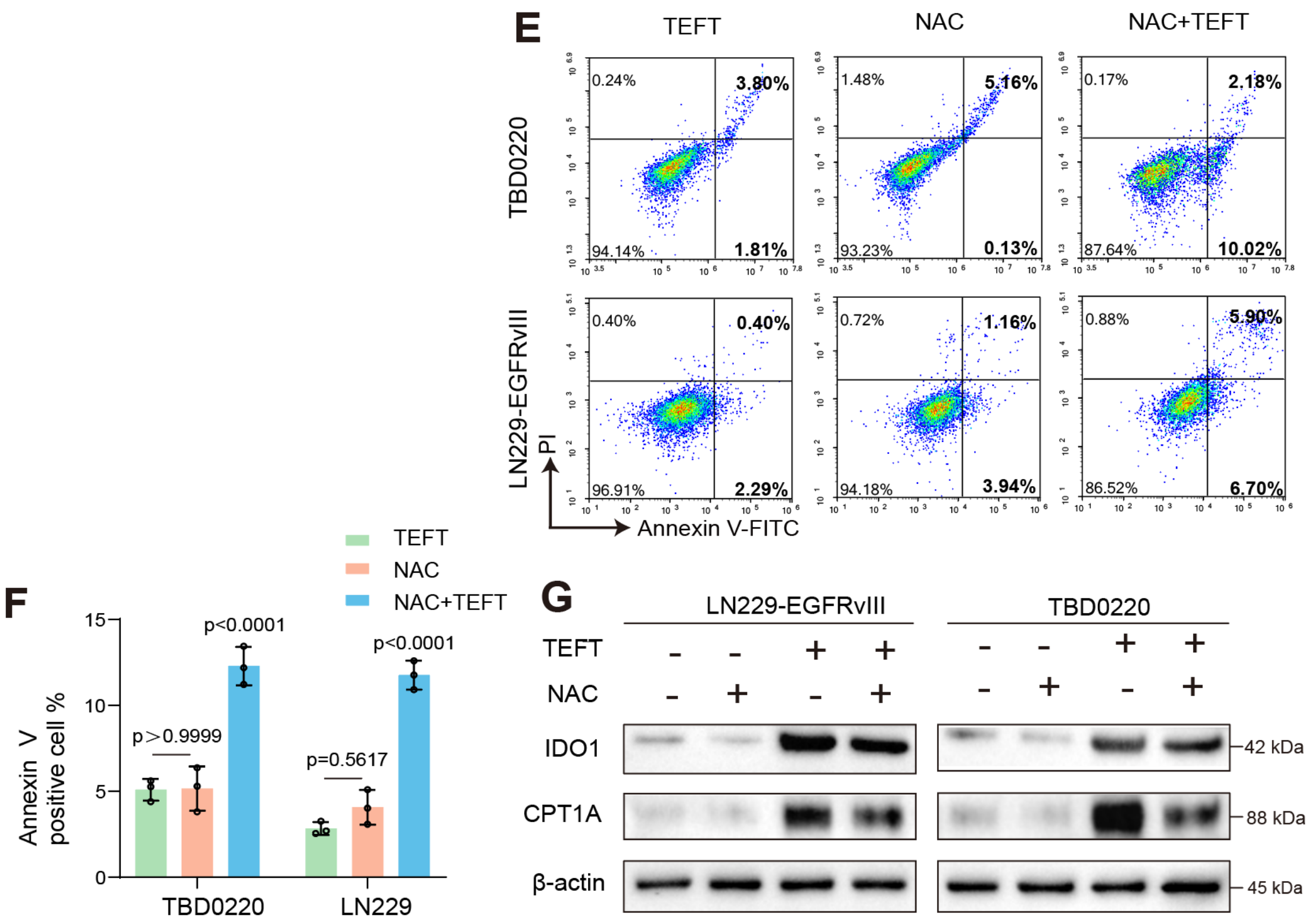


**Fig. S7 (E)** Representative Annexin V/PI flow cytometry plots of TBD0220 and LN229-EGFRvIII cells treated with TEFT, NAC, or TEFT plus NAC, **(F)** Quantification of Annexin V-positive cells from panel F. NAC co-treatment increased apoptosis under TEFT exposure, indicating partial restoration of TEFT sensitivity, **(G)** Western blot analysis of IDO1 and CPT1A protein levels in LN229-EGFRvIII and TBD0220 cells treated with TEFT, NAC, or TEFT plus NAC.

These results indicate that TEFT-induced redox stress contributes principally to the upstream CPT1A-associated metabolic-adaptation arm. However, redox stress is not the sole determinant of total IDO1 protein abundance or of the complete resistant phenotype. IDO1 is regulated at multiple levels, including EGFRvIII–NF-κB-dependent transcriptional supply and CPT1A-associated post-translational stabilization. Consequently, attenuation of CPT1A induction at a single experimental time point may not immediately produce a proportional change in total IDO1 protein.

We have therefore avoided stating that NAC completely reverses IDO1 stabilization. The revised interpretation is that the pathway is partially redox-dependent at the level

of upstream metabolic adaptation, while the full IDO1-dependent resistance program integrates both redox-sensitive and redox-independent regulatory inputs.

**Other Changes in the revised manuscript are provided below.**
**Results, lines 250–256;**

To assess whether redox stress contributes to this adaptive response, we treated cells with the ROS scavenger NAC. NAC co-treatment increased TEFT-induced apoptosis and attenuated CPT1A protein accumulation. In contrast, total IDO1 abundance was only modestly affected and did not show a consistent reduction across the tested models (fig. S7E-G). These findings suggest that redox stress contributes primarily to the upstream CPT1A-associated metabolic adaptation, but is not the sole determinant of IDO1 abundance or the TEFT-resistant phenotype.

**Discussion, lines 487–488;**

NAC increased TEFT-induced apoptosis and attenuated CPT1A accumulation, suggesting that redox stress contributes to the upstream metabolic adaptation.

# Response Letter to Reviewers

## Responses to the comments of Reviewer #2

Reviewer 2 Comments to the Author: The authors have substantially revised the manuscript and addressed the majority of my prior comments. Key findings are now validated in TBD0220 and 2 additional low-passage patient-derived models, with added genetic and pharmacologic IDO1 studies, mitochondrial-fraction interaction assays, higher-resolution microscopy, recombinant succinylation experiments, kynurenine measurements, and Bliss synergy analysis. Together, these data provide consistent support for the proposed mechanism.

Some mechanistic details, including precise mapping of the CPT1A–IDO1 interaction interface and its submitochondrial topology, remain unresolved, but these are appropriate for future studies rather than required for publication. The clinical claims are now appropriately restrained, with the cohort and compassionate-use cases presented as exploratory. Larger prospective studies will be needed to confirm these findings but are beyond the scope of the current work. The in vitro osimertinib dose-response studies address my prior comment, while in vivo dose optimization can be pursued in future preclinical work before clinical testing.

In my view, the authors also responded appropriately to the other reviewers' concerns. The distinction between a broadly induced TEFT metabolic-stress response and EGFRvIII-dependent IDO1 priming is now clearer. The recombinant succinylation assays, kynurenine measurements, mitochondrial gating controls, thermal controls, and integrated mechanistic model all strengthen the work.

Overall, the revision is materially stronger and has passed the bar for the major concerns raised. I therefore support publication. Congratulations.

**Response**: We are truly grateful for the reviewer's generous and encouraging assessment of our revised manuscript. The detailed comments provided throughout the review process helped us identify where the original manuscript needed stronger evidence, clearer reasoning, and more careful interpretation. We are pleased that the additional experiments and revisions have addressed the major concerns and made the overall mechanistic framework more convincing.

We fully agree that the precise CPT1A–IDO1 interaction interface, its submitochondrial organization, optimization of the in vivo osimertinib regimen, and prospective clinical validation remain important questions. We look forward to pursuing these directions in our future work. We sincerely appreciate the time and thought the reviewer has devoted to this study, as well as the kind words and support for publication.

## Responses to the comments of Reviewer #3

Reviewer 3 Comments to the Author: The authors have provided a comprehensive revision that completely answers all the concerns. I do not have any questions and prefer acceptance for publishing.

**Response**: We sincerely thank the reviewer for the positive and encouraging assessment. We are very pleased that the revised manuscript has addressed all the concerns raised. The reviewer's comments during the review process were valuable in helping us improve the rigor and clarity of the work. We greatly appreciate the time devoted to evaluating our manuscript and the support for its publication.
